# The onset of PI3K‐related vascular malformations occurs during angiogenesis and is prevented by the AKT inhibitor miransertib

**DOI:** 10.15252/emmm.202115619

**Published:** 2022-06-13

**Authors:** Piotr Kobialka, Helena Sabata, Odena Vilalta, Leonor Gouveia, Ana Angulo‐Urarte, Laia Muixí, Jasmina Zanoncello, Oscar Muñoz‐Aznar, Nagore G Olaciregui, Lucia Fanlo, Anna Esteve‐Codina, Cinzia Lavarino, Biola M Javierre, Veronica Celis, Carlota Rovira, Susana López‐Fernández, Eulàlia Baselga, Jaume Mora, Sandra D Castillo, Mariona Graupera

**Affiliations:** ^1^ Endothelial Pathobiology and Microenvironment Josep Carreras Leukaemia Research Institute Barcelona Spain; ^2^ Department of Immunology, Genetics, and Pathology Uppsala University Uppsala Sweden; ^3^ Developmental Tumor Biology Laboratory Institut de Recerca Sant Joan de Déu Barcelona Spain; ^4^ 3D Chromatin Organization Josep Carreras Leukaemia Research Institute Barcelona Spain; ^5^ CNAG‐CRG, Centre for Genomic Regulation Barcelona Institute of Science and Technology Barcelona Spain; ^6^ Universitat Pompeu Fabra (UPF) Barcelona Spain; ^7^ Pediatric Cancer Center Barcelona Hospital Sant Joan de Déu Barcelona Barcelona Spain; ^8^ Department of Pathology Hospital Sant Joan de Déu Barcelona Barcelona Spain; ^9^ Department of Plastic Surgery Hospital de la Santa Creu i de Sant Pau Barcelona Spain; ^10^ Department of Dermatology Hospital Sant Joan de Déu Barcelona Spain; ^11^ CIBERONC Instituto de Salud Carlos III Madrid Spain

**Keywords:** AKT, angiogenesis, endothelial cell, PI3K, vascular malformations, Cardiovascular System, Vascular Biology & Angiogenesis

## Abstract

Low‐flow vascular malformations are congenital overgrowths composed of abnormal blood vessels potentially causing pain, bleeding and obstruction of different organs. These diseases are caused by oncogenic mutations in the endothelium, which result in overactivation of the PI3K/AKT pathway. Lack of robust *in vivo* preclinical data has prevented the development and translation into clinical trials of specific molecular therapies for these diseases. Here, we demonstrate that the *Pik3ca^H1047R^* activating mutation in endothelial cells triggers a transcriptome rewiring that leads to enhanced cell proliferation. We describe a new reproducible preclinical *in vivo* model of PI3K‐driven vascular malformations using the postnatal mouse retina. We show that active angiogenesis is required for the pathogenesis of vascular malformations caused by activating *Pik3ca* mutations. Using this model, we demonstrate that the AKT inhibitor miransertib both prevents and induces the regression of PI3K‐driven vascular malformations. We confirmed the efficacy of miransertib in isolated human endothelial cells with genotypes spanning most of human low‐flow vascular malformations.

## Graphical abstract

This work describes a robust preclinical model of PI3K‐driven vascular malformations using the postnatal mouse retina. We show that AKT inhibition by miransertib is an effective therapeutic strategy for these diseases.

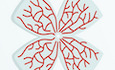

**The paper explained**

**Problem**

Low‐flow vascular malformations are congenital diseases caused by a focal overgrowth of vessels. They may cause pain, bleeding, infections and obstruction of organs. While their genetic causes, leading to hyperactivation of PI3K signalling, have been known for a few years, no targeted molecular therapy has been approved so far.

**Results**

*Pik3ca* mutation in ECs activates transcriptomic changes, leading to enhanced cell cycle progression in the presence of growth factors. We have generated a robust and fast preclinical *in vivo* model that allows for testing of targeted drugs. With this model, we demonstrated that PI3K‐driven vascular malformations rely on active angiogenesis. Our preclinical studies show that AKT inhibition using miransertib prevents the disease and fully regresses established vascular malformations.

**Impact**

Our new *in vivo* model of PI3K‐driven vascular malformations constitutes a reliable and fast preclinical setting to test new or repurposed targeted drugs. Our studies support that *Pik3ca*‐mutant ECs cause vascular malformations upon growth stimuli, highlighting the importance of preventive therapeutic approaches after invasive treatments. We provide proof of concept for the use of the AKT inhibitor miransertib in PI3K‐driven vascular malformations, which opens a new window for targeted therapeutic intervention for these diseases. We further demonstrate *in vitro* that this targeted therapy is similarly effective in both *PIK3CA* and *TEK*‐mutant vascular malformations.

## Introduction

Vascular malformations are a congenital group of diseases composed of abnormal vascular channels that can occur anywhere in the body and often have a major impact on the quality of life of patients. They tend to be painful and disfiguring and many leading to bleeding, recurrent infections, thrombosis, organ dysfunction and even death (Van Damme *et al*, 2020). Vascular malformations can be classified as low‐flow (venous, lymphatic and capillary) and fast‐flow (arteriovenous) lesions, the former being the most frequent subtype. These vascular lesions appear during embryonic development, when vascular growth factors are produced at high levels, and expand proportionally with the physiological growth of the patient (Pang *et al*, 2020). Of note, vascular malformations may occur in isolation or as part of a syndrome (Canaud *et al*, 2021). At present, there is no molecularly targeted therapy in the current management for these diseases. Instead, standard of care includes a broad spectra of mostly inefficient and invasive techniques including bandage compression, surgical excision and sclerosing approaches (Castillo *et al*, 2019).

Overactivation of phosphoinositide 3‐kinase (PI3K) signalling is a hallmark of most low‐flow vascular malformations (Castillo *et al*, 2016b, 2019; Canaud *et al*, 2021; Mäkinen *et al*, 2021). Sporadic venous malformations, the most common type of vascular malformations, are caused by gain‐of‐function mutations either in the endothelial tyrosine kinase receptor *TEK*/TIE2 or in the PI3K catalytic subunit alpha *PIK3CA* (Limaye *et al*, 2015; Castel *et al*, 2016; Castillo *et al*, 2016a); with *TEK* and *PIK3CA* mutations largely being mutually exclusive. Also, about 85% of lymphatic malformations are caused by activating *PIK3CA* mutations (Boscolo *et al*, 2015; Luks *et al*, 2015; Mäkinen *et al*, 2021). In addition, venous, lymphatic and/or capillary malformations are frequently present in overgrowth syndromes caused by *PIK3CA* mutations, the so‐called PROS (*PIK3CA*‐related overgrowth spectrum) (Keppler‐Noreuil *et al*, 2015). *PIK3CA* mutations in vascular malformations are similar to those found in epithelial cancer, being the missense mutations in the helical (*PIK3CA^E542K^* and *PIK3CA^E545K^*) and the kinase (*PIK3CA^H1047R^*) domains the most prevalent (Samuels *et al*, 2004).

*PIK3CA* encodes the p110α lipid kinase protein, which is a major signalling component downstream of growth factor receptor tyrosine kinases (RTKs) (Bilanges *et al*, 2019; Kobialka & Graupera, 2019). Specifically in endothelial cells (ECs), p110α is activated by the vascular endothelial growth factor receptors (VEGF‐R) and TIE tyrosine kinase receptors (Graupera & Potente, 2013). Hence, it is not surprising that p110α is the sole class I PI3K isoform required for blood and lymphatic vascular development (Graupera *et al*, 2008; Stanczuk *et al*, 2015). p110α catalyses the phosphorylation of the lipid second messenger phosphatidylinositol‐4,5‐triphosphate (PIP_2_) to phosphatidylinositol‐3,4,5‐triphosphate (PIP_3_) at the cell membrane (Bilanges *et al*, 2019). PIP_3_, in turn, contributes to the recruitment and activation of a wide range of downstream targets, the serine‐threonine protein kinase AKT (also known as protein kinase B, PKB) being critical in this cascade. The PI3K‐AKT signalling pathway regulates many cellular processes that are key for EC biology, including cell proliferation, survival and motility (Manning & Toker, 2017; Bilanges *et al*, 2019). There are three isoforms of AKT (AKT1, 2 and 3), showing high homology but being not redundant. AKT1 and AKT2 are broadly expressed, with AKT1 being the predominant isoform in ECs (Ackah *et al*, 2005; Chen *et al*, 2005).

The discovery that most low‐flow lesions are caused by overactivation of PI3K signalling has catalysed the repurpose of PI3K pathway inhibitors for the treatment of these diseases. Given that pathological mutant ECs primarily depend on AKT signalling, inhibition of AKT is a promising strategy for low‐flow vascular malformations. Among AKT inhibitors, miransertib (ARQ 092, MK‐7075) is a potent and selective allosteric AKT inhibitor showing higher specificity for the AKT1 isoform. Miransertib suppresses AKT activity by inhibiting membrane‐bound active form of AKT and preventing activation of the inactive form of AKT (Yu *et al*, 2015). This inhibitor has shown efficacy in preclinical studies for PI3K‐driven tumours (Yu *et al*, 2015, 2017) and Proteus syndrome (Lindhurst *et al*, 2015), which is caused by a somatic AKT gain‐of‐function mutation. Also, compassionate use of this inhibitor has shown therapeutic efficacy in patients with Proteus and PROS (Leoni *et al*, 2019; Biesecker *et al*, 2020; Forde *et al*, 2021).

Here, we show that *Pik3ca^H1047R^* expression in ECs triggers a transcriptional programme favouring cell cycle progression. Cell cycle progression in *Pik3ca^H1047R^* ECs is responsive to growth factors; thus, active angiogenesis is required for the formation of *Pik3ca*‐driven vascular malformations. We report a unique *in vivo* model of PI3K‐driven vascular malformations that allows a more accurate understanding of the dynamic pathogenesis of these diseases and thus a more efficient assessment of therapeutic strategies. In addition, we show how analysing patient‐derived ECs from vascular malformations allows for personalized medicine testing in these diseases. Using a spectra of preclinical models, we demonstrate the efficacy of the AKT inhibitor miransertib for PI3K‐driven vascular malformations both for prevention and treatment strategies.

## Results

### PI3K overactivation in ECs leads to a molecular signature for enhanced cell cycle progression

**Figure 1:**
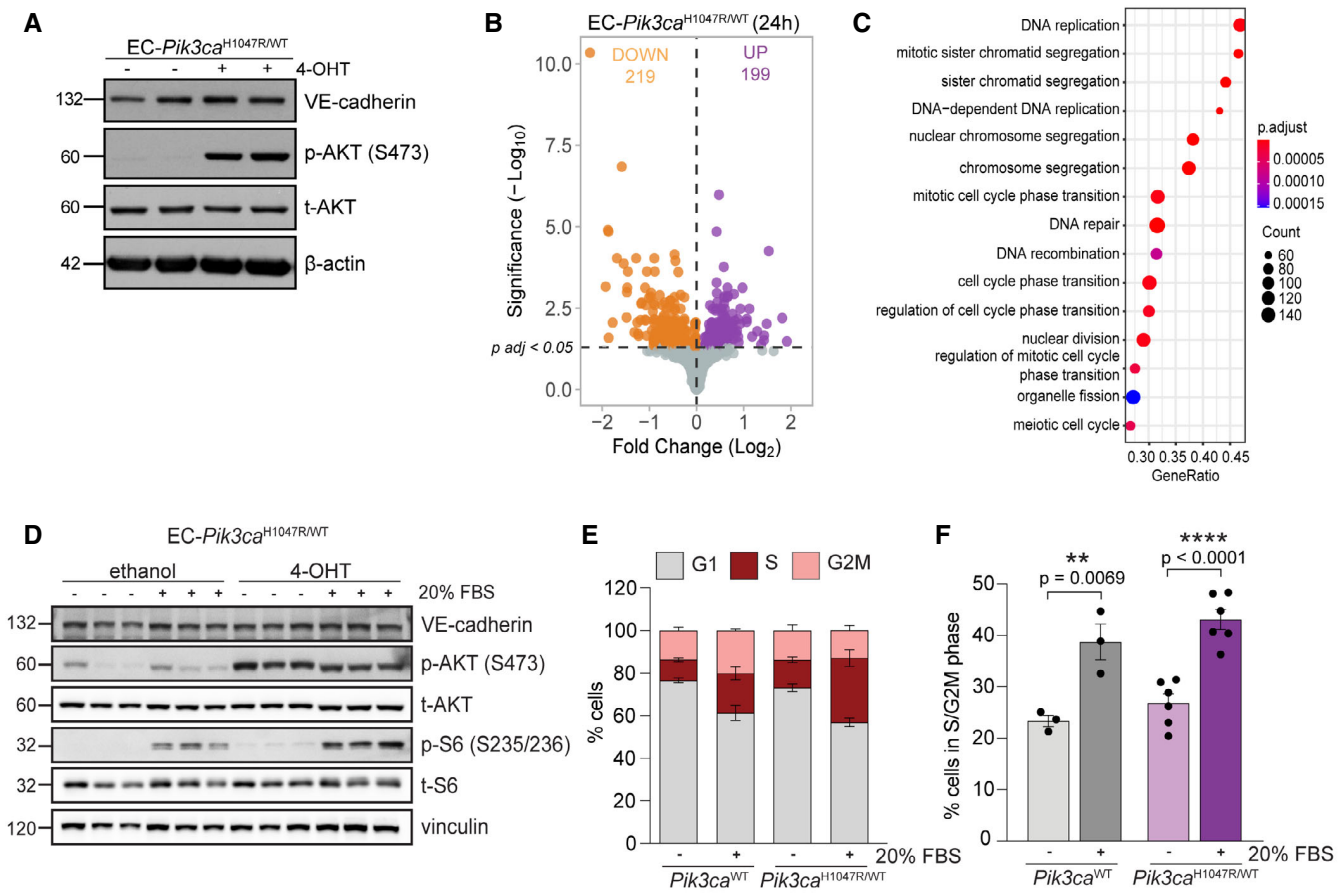
PI3K pathway activation in ECs leads to enhanced cell cycle progression AImmunoblot showing the activation of PI3K/AKT pathway (by assessing the levels of p‐AKT Ser473) of ECs 24 h after induction of *Pik3ca^H1047R^* expression.BVolcano plot of genes analysed by RNAseq in ECs upon *Pik3ca^H1047R^* expression. Differentially expressed genes (DEGs) (*P*_adj_ < 0.05) in *Pik3ca^H1047R^* over *Pik3ca^WT^* ECs at 24 h (grey, unchanged; orange, downregulated; purple, upregulated). *n* = 4 biological replicates per genotype.CPathway enrichment analysis showing most represented biological functions altered upon *Pik3ca^H1047R^* expression in ECs.DImmunoblot showing the activation of PI3K/AKT pathway of *Pik3ca^WT^* and *Pik3ca^H1047R^* ECs after 2 h starvation (−) and 30 min stimulation with 20% foetal bovine serum (FBS) (+).E, F(E) Cell cycle analysis by flow cytometry of *Pik3ca^WT^* and *Pik3ca^H1047R^* ECs after cultured either without serum (−) or 20% serum (+) for 24 h. (F) Percentage of proliferative ECs (in S and G2 M phases) from Fig 1E. Error bars are SEM. *n* ≥ 3 biological replicates per genotype. Statistical analysis was performed by nonparametric Mann–Whitney test. **P < 0.01 and ****P < 0.0001 were considered statistically significant. Source data are available online for this figure.

To understand the pathogenesis of PI3K‐driven blood vascular malformations, we analysed early changes in the transcriptome of ECs upon *Pik3ca^H1047R^* expression. For this, we isolated lung ECs from mice, which express the *Pik3ca^H1047R^* mutation under the endogenous promoter (Kinross *et al*, 2012) specifically in ECs (Pdgfb‐iCreER) upon 4‐hydroxytamoxifen (4‐OHT) treatment (Claxton *et al*, 2008). First, we aim to define a time point to assess for an early impact on the transcriptome driven by *Pik3ca^H1047R^*. To this end, we first analysed the expression of *Ang2* and *Pdgfb*, two bonafide transcriptomic readouts of PI3K activation in ECs (Potente *et al*, 2005; Castel *et al*, 2016; Castillo *et al*, 2016a) at 6, 24, 48, and 72 h after induction of *Pik3ca^H1047R^* expression (Appendix Fig S1A). We found that analysis at 24 h post‐4‐OHT was sufficient to induce PI3K‐related transcriptional changes (Appendix Fig S1A). We confirmed that PI3K signalling was overactivated at 24 h after 4‐OHT by showing enhanced phospho (p)‐Akt compared with non‐induced cells (Fig 1A). Next, we performed transcriptomic analysis by RNA sequencing of ECs at 24 h after 4‐OHT and their vehicle‐treated counterparts. In total, 14,226 genes were analysed and 418 genes resulted differentially expressed (*P*_adj_ < 0.05) (Fig 1B). Pathway enrichment analysis determined that the most affected biological processes upon *Pik3ca^H1047R^* expression in ECs included cell division, mitosis and cell cycle progression (Fig 1C). In line with this, gene set enrichment analysis (GSEA) confirmed that the top enriched hallmarks upon *Pik3ca^H1047R^* expression include E2F and MYC targets and G2 M checkpoints (Appendix Table S1, Appendix Fig S1B).

To assess the biological relevance of the proliferative molecular programme triggered by *Pik3ca^H1047R^* expression, we next studied the impact of PI3K overactivation in endothelial cell cycle upon both serum starvation and stimulation. Wild‐type ECs showed PI3K/AKT signalling activation 30 min after serum stimulation, while *Pik3ca^H1047R^* ECs showed stronger activation in both starved and serum‐stimulated culture conditions (Fig 1D). Cell cycle analysis revealed that, even though PI3K/AKT signalling was similarly overactivated in both starved and serum‐stimulated conditions of *Pik3ca^H1047R^* ECs, solely the presence of growth factors resulted in a significant overrepresentation of ECs in the S and G2/M phases (Fig 1E and F). The enhanced proliferative state upon growth factors correlated with pS6 levels (Fig 1D), thereby indicating that the AKT/mTOR axis is relevant for the pathological proliferation of *Pik3ca^H1047R^* ECs.

### Active angiogenesis is required for the formation of PI3K‐driven vascular malformations

**Figure 2:**
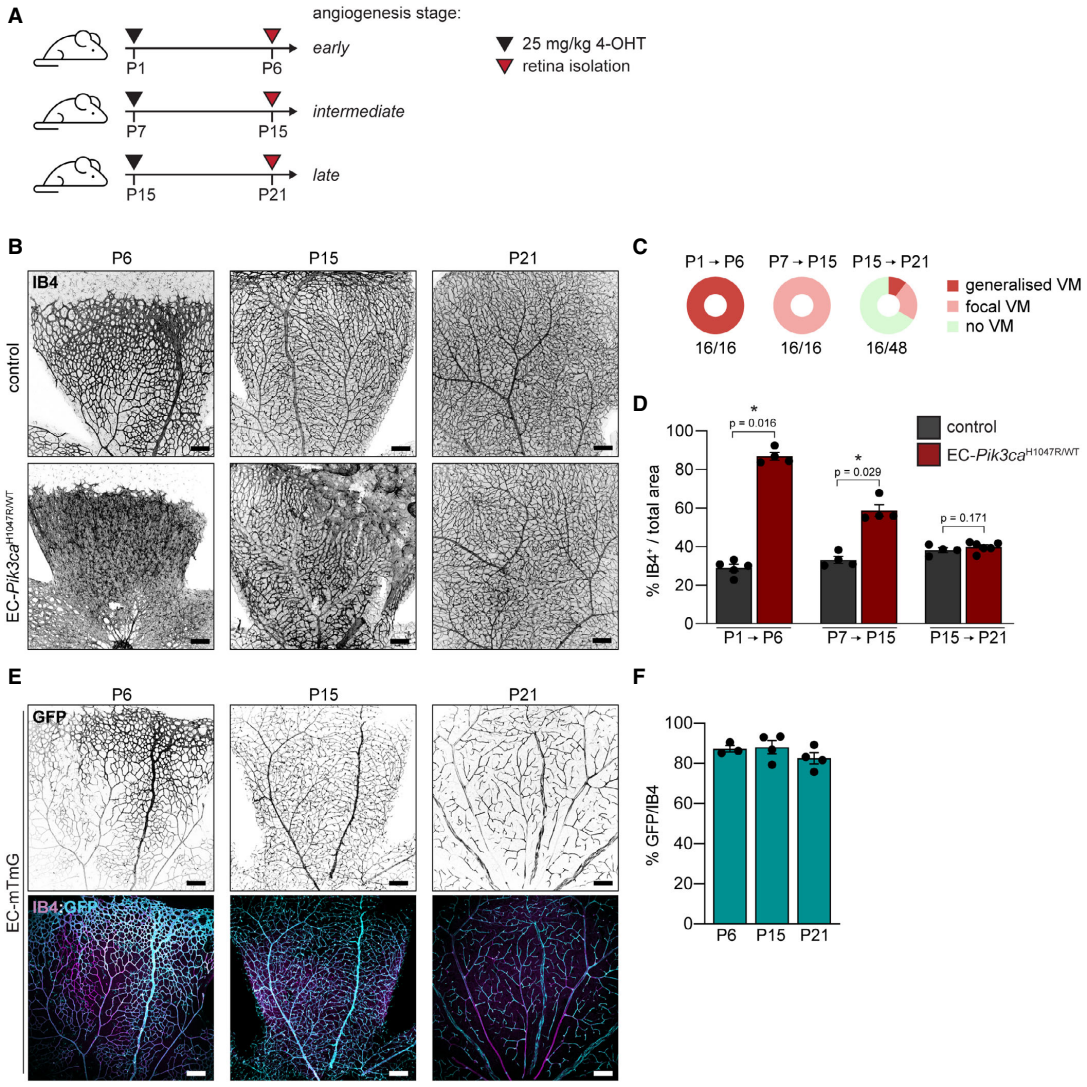
Pathogenesis of *Pik3ca*‐driven vascular malformations depends on active angiogenesis Experimental setup scheme showing analysed angiogenic stages in the retina models.Representative images of control and EC‐*Pik3ca^H1047R/WT^* mouse retinas isolated at indicated time points stained with IB4 for blood vessels. Scale bars: 150 μm.Pie charts showing the incidence of vascular lesions at different angiogenic stages. Lesions were categorized according to their expanse (generalized or focal). Quantification was performed per retina petal. Numbers below show the presence of any type of lesions per retina petal.Quantification of IB4‐positive areas per retina. Error bars are SEM. *n* ≥ 3 retinas per genotype.Representative images of EC‐mTmG mouse retinas isolated at indicated time points stained with IB4 and GFP. Scale bars: 150 μm.Quantification of GFP/IB4 ratio per retina. Error bars are SEM. *n* ≥ 3 retinas per genotype. Data information: Statistical analysis was performed by nonparametric Mann–Whitney test. *P < 0.05 was considered statistically significant. Source data are available online for this figure.

Our data suggest that *Pik3ca^H1047R^*‐driven EC proliferation requires the presence of growth factors to induce a pathological response. Given that vascular malformations appear during embryonic development when endothelial mitogenic signals are produced at high concentration, we hypothesized that the onset of these lesions depends on angiogenesis. To test this idea, we took advantage of the mouse retinal vasculature, which allows the study of the different phases of vascular development. We chose three developmental stages (early, intermediate and late) in which ECs exhibit different dependency on mitogenic signals (Ehling *et al*, 2013) to induce the endogenous expression of the *Pik3ca^H1047R^* mutation in ECs *in vivo*. 4‐OHT was administered at postnatal day (P)1, P7 or P15, and retinas were isolated 1 week later (Fig 2A). By analysing the extent of vascular overgrowth, we found that both early and intermediate stages showed full penetrance, with all retinas analysed developing vascular malformations (Fig 2B and C). However, the degree of enhanced vascularity was more prominent and generalized in the early developmental stage than in the intermediate period (Fig 2B–D). In contrast, only one‐third of the retinas showed malformed vascular areas when *Pik3ca^H1047R^* mutation was induced at P15 (Fig 2B–D). By taking advantage of ROSA^mTmG^; Pdgfb‐iCreER (later referred to as EC‐mTmG) reporter mice (Muzumdar *et al*, 2007), which expresses cell membrane‐localized EGFP following Cre recombination, we demonstrated that the Pdgfb‐iCreER line is similarly active at all time points tested (Fig 2E and F). Thus, penetrance and severity of vascular malformations in our model were independent of the number of ECs recombined at the different stages. These results indicated that the expression of mutant *Pik3ca* in ECs is not sufficient for the acquisition of a malformed vascular phenotype and that active angiogenesis is required for PI3K‐driven vascular malformations to occur.

### A new preclinical model of PI3K‐driven vascular malformations

**Figure 3:**
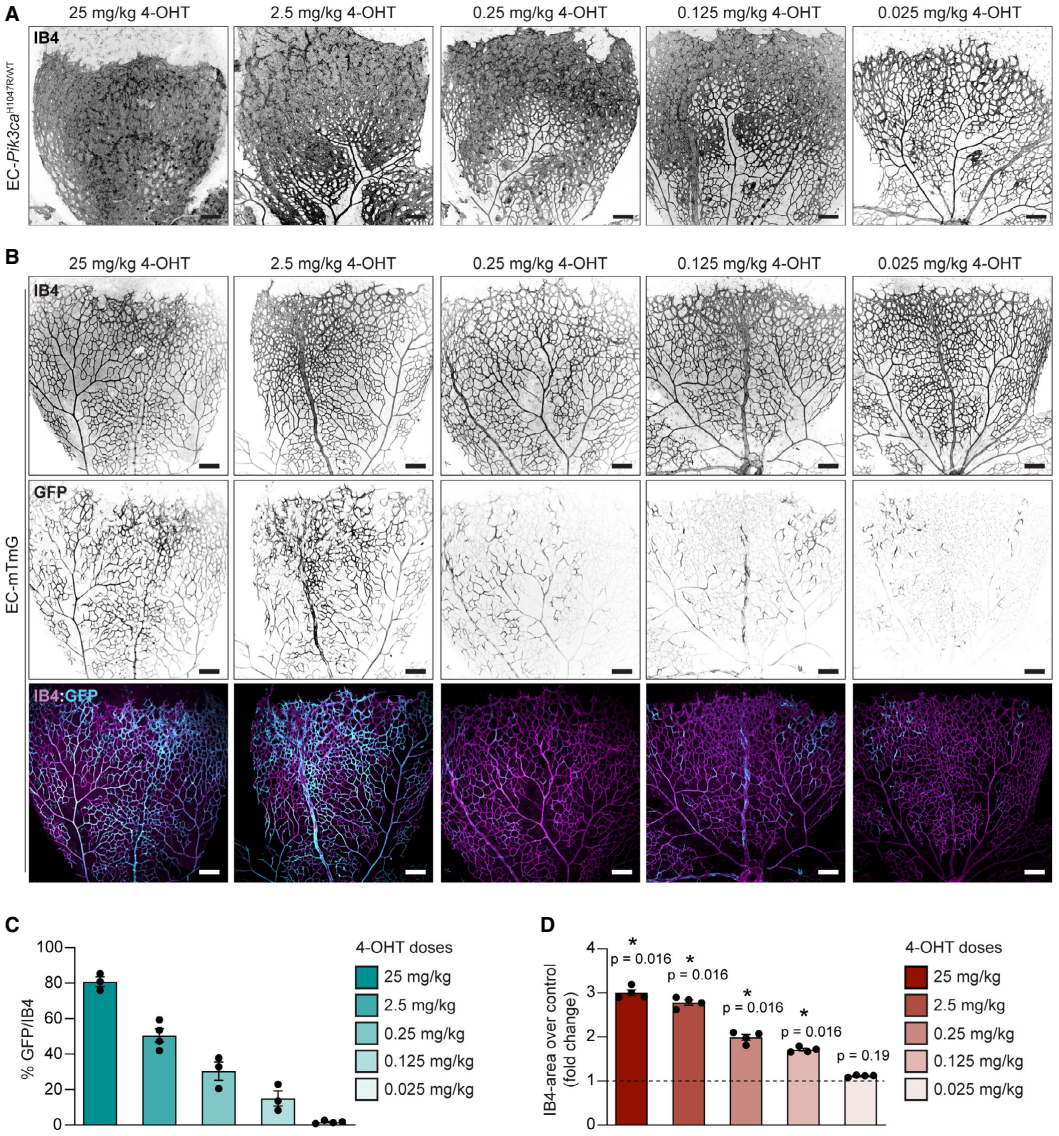
Modelling PI3K‐vascular malformations in murine retinas by mosaic expression of *Pik3ca^H1047R^* in ECs A, BRepresentative images of EC‐*Pik3ca^H1047R/WT^* (A) and EC‐mTmG (B) P6 retinas from mice treated with decreasing doses of 4‐OHT on P1. Retinas were stained for blood vessels (IB4) and GFP as indicated. Scale bars: 150 μm.CQuantification of GFP/IB4 ratio of EC‐mTmG retinas. Error bars are SEM. *n* ≥ 3 retinas per genotype.DQuantification of IB4‐positive area per retina in *Pik3ca^H1047R/WT^* retinas. Data presented as a percentage of the control for each 4‐OHT dose. Error bars are SEM. *n* = 4 retinas per genotype. Data information: Statistical analysis was performed by nonparametric Mann–Whitney test. *P < 0.05 was considered statistically significant. Source data are available online for this figure.

**Figure 4:**
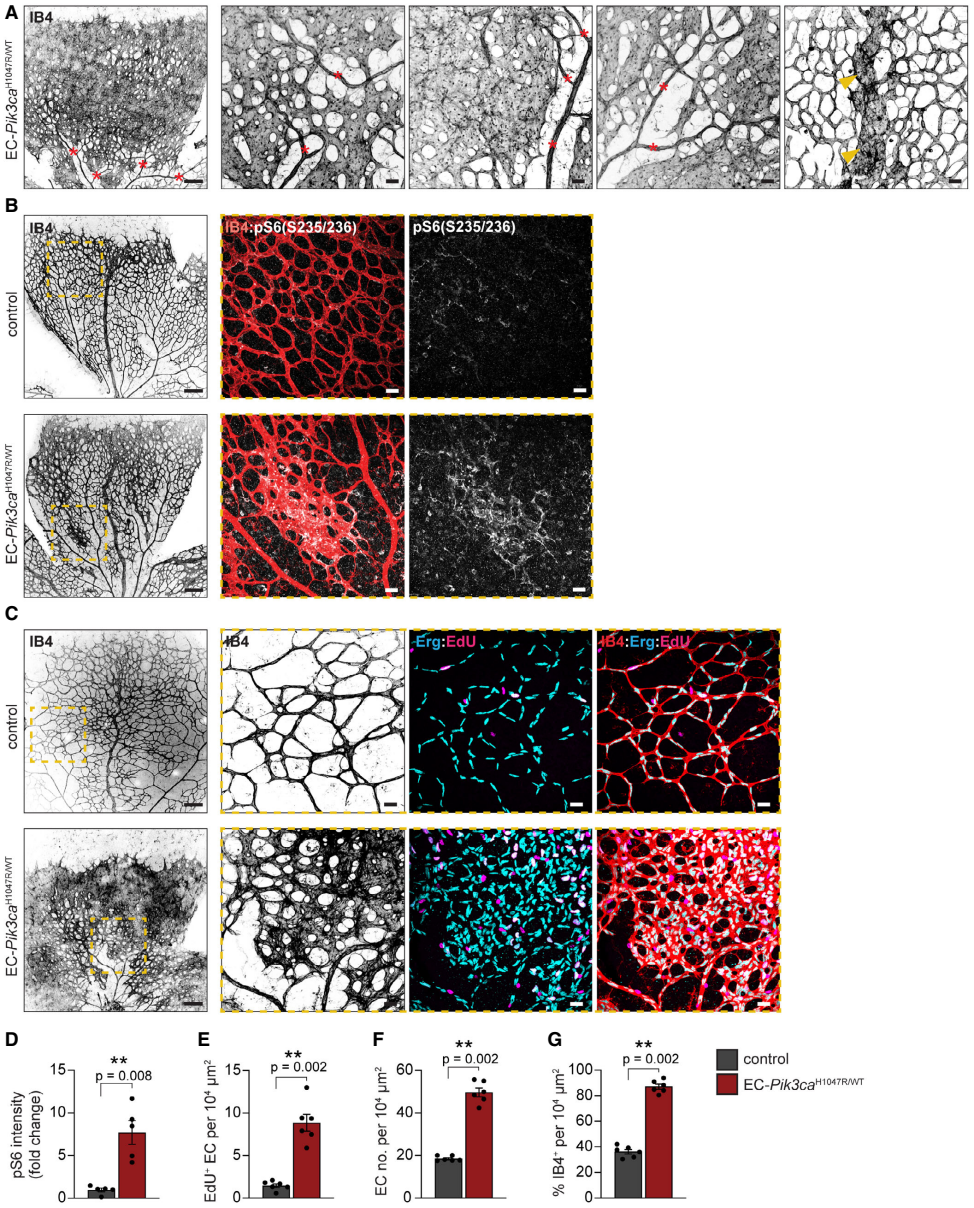
*Pik3ca*‐vascular malformations in murine retinas reproduce hallmarks of human disease ARepresentative images of EC‐*Pik3ca^H1047R/WT^* P6 retinas isolated from mice treated with 0.125 mg/kg of 4‐OHT on P1. Retinas were immunostained for blood vessels (IB4). Red asterisks show arteries and arterioles and yellow arrowheads veins. Scale bars: 150 μm (left panel) and 30 μm (right, high magnification panels).BRepresentative images of P6 retinas from control and EC‐*Pik3ca^H1047R/WT^* mice treated with 0.125 mg/kg 4‐OHT on P1, following immunostaining for p‐S6 (S235/236) and blood vessels (IB4). Scale bars: 150 μm (left panels) and 30 μm (right panels, high magnification).CRepresentative control and EC‐*Pik3ca^H1047R/WT^* P6 retinas immunostained for blood vessels (IB4), EC nuclei (Erg) and EdU. Scale bars: 150 μm (left panels) and 30 μm (right panels, high magnification).D–GQuantification of (D) p‐S6 (S235/236) intensity (presented as a fold change of vehicle‐treated control), (E) EC proliferation by EdU staining, (F) EC number by Erg‐positive cells and (G) retinal vascularity by IB4‐positive area in control and EC‐*Pik3ca^H1047R/WT^* retinas. Error bars are SEM. *n* > 5 retinas per genotype. Statistical analysis was performed by nonparametric Mann–Whitney test. **P < 0.01 was considered statistically significant. Source data are available online for this figure.

**Figure EV1:**
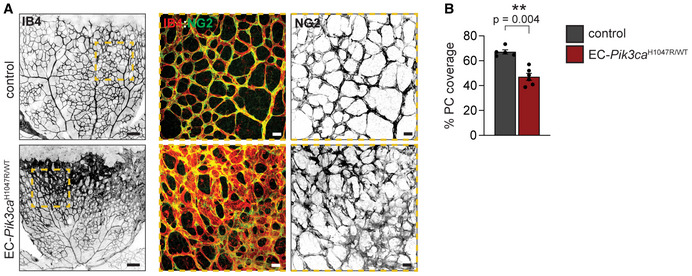
*Pik3ca*‐driven vascular malformations exhibit reduced coverage by pericytes Representative images of control and EC‐*Pik3ca* ^H1047R/WT^ P6 retinas immunostained for blood vessels (IB4) and pericyte marker (NG2). Dashed areas with high magnifications shown on the right. Scale bars: 150 μm (left panels) and 30 μm (right panels).Quantification of vessel coverage by pericytes. Data presented as a percentage of pericyte coverage over EC area (IB4 staining). Error bars are SEM. *n* ≥ 5 retinas per genotype. Statistical analysis was performed by nonparametric Mann–Whitney test. **P < 0.01 was considered statistically significant. Source data are available online for this figure.

**Figure EV2:**
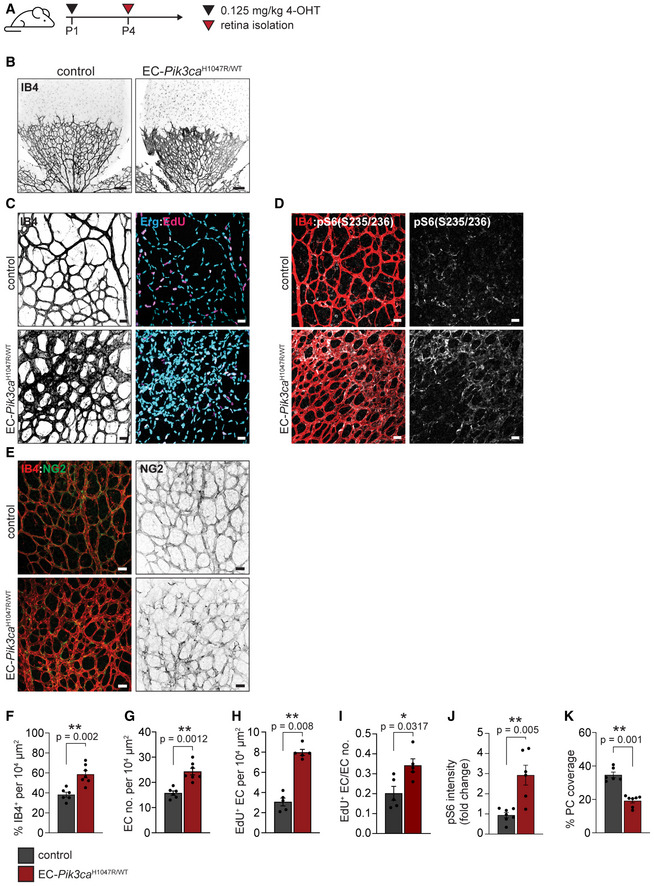
EC‐*Pik3ca* ^H1047R/WT^ P4 retinas exhibit vascular malformations AScheme showing 4‐OHT treatment regime.BRepresentative images of P4 retinas from control and EC‐*Pik3ca* ^H1047R/WT^ immunostained for blood vessels (IB4). Scale bars: 150 μm.CRepresentative high magnification images showing blood vessels (IB4), EC nuclei (Erg) and proliferative cells (EdU).DRepresentative high magnification images of retinas immunostained for blood vessels (IB4) and pS6 (S235/236).ERepresentative high magnification images showing blood vessels (IB4) and pericytes (NG2).F–KQuantification of (F) retina vascularity, (G) EC number, (H) EC proliferation by EdU staining, (I) mitotic index (EdU+ EC/total EC), (J) pS6 intensity and (K) pericyte coverage in control and EC‐*Pik3ca* ^H1047R/WT^ P4 retinas. Error bars are SEM. *n* ≥ 5 retinas per genotype. Statistical analysis was performed by nonparametric Mann–Whitney test. *P < 0.05 and **P < 0.01 were considered statistically significant. Data information: Scale bars (C, D and E) = 30 μm. Source data are available online for this figure.

Our data showed that expression of *Pik3ca^H1047R^* in ECs at an early stage of postnatal angiogenesis leads to generalized vascular malformations. However, sporadic vascular malformations appear as a mosaic disease, where malformed vascular lesions tend to be isolated and focal. Hence, to better reproduce the aetiology of human disease, we studied the impact of a decreasing range of 4‐OHT doses during the early developmental stage in EC‐Pik3ca^WT/H1047R^ mouse retinas (Fig 3A). To validate mosaicism and identify targeted ECs in our dosing strategy, we treated EC‐mTmG P1 mice in parallel with the same doses (Fig 3B and C). This approach allowed us to identify the lowest 4‐OHT dose (0.125 mg/kg) that led to distinguishable vascular malformations with a total vascular density significantly increased compared with wild‐type counterparts (Fig 3A and D). We noticed that low dose of 4‐OHT targeted all vessel subtypes shown by mTmG‐positive cells in arteries, veins and capillaries (Fig 3B). Instead, expression of *Pik3ca^H1047R^* upon low dose of 4‐OHT resulted in the formation of vascular malformations only in veins and capillaries (Fig 4A). *Pik3ca^H1047R^*‐vascular malformations in postnatal retinas showed enriched phospho (p)‐S6 (Ser235/236) levels, a read‐out for PI3K/AKT/mTORC1 signalling, compared with the surrounding normal vasculature and wild‐type retinas (Fig 4B and D). These lesions also exhibited loss of pericyte coverage, a hallmark of vascular malformations, in contrast to non‐malformed vasculature in the same retina and the control (Fig EV1). A 2‐h snapshot of the number of ECs in S‐phase unveiled that *Pik3ca^H1047R^* mutant retinas exhibited higher number of proliferative ECs (Fig 4C and E). Also, *Pik3ca^H1047R^* mutant retinas also showed that the malformed vasculature was composed of more ECs, which may result in no difference in the mitotic index between genotypes at this time point (Fig 4C and F). To further understand whether *Pik3ca*‐related vascular malformations emerge as a consequence of enhanced EC proliferation, we postulated that the analysis of ECs in S‐phase had to be done before the accumulation of too many ECs. To test this, we first induced the formation of vascular malformations by treating EC‐*Pik3ca^H1047R^* mice with 4‐OHT at P1 and retinas were analysed at P4 (Fig EV2A). At this time point, *Pik3ca^H1047R^* retinas showed vascular malformations (Fig EV2B and F), a modest increase in the number of ECs (Fig EV2C and G), as well as a substantial increase in EdU‐positive ECs (Fig EV2C and H). This allowed to ascertain that during the early onset of vascular malformations, *Pik3ca^H1047R^* leads to an increased mitotic index in ECs (Fig EV2I). In addition, P4 mutant retinas exhibited other hallmarks of PI3K‐driven vascular malformations such as enhanced pS6 levels (Fig EV2D and J) and impaired pericyte coverage (Fig EV2E and K). Collectively, these data show that EC‐specific mosaic induction of endogenous *Pik3ca^H1047R^* expression during active vascular growth faithfully recapitulates human low‐flow vascular malformations.

### AKT inhibitor miransertib prevents the formation of PI3K‐driven vascular malformations

**Figure 5:**
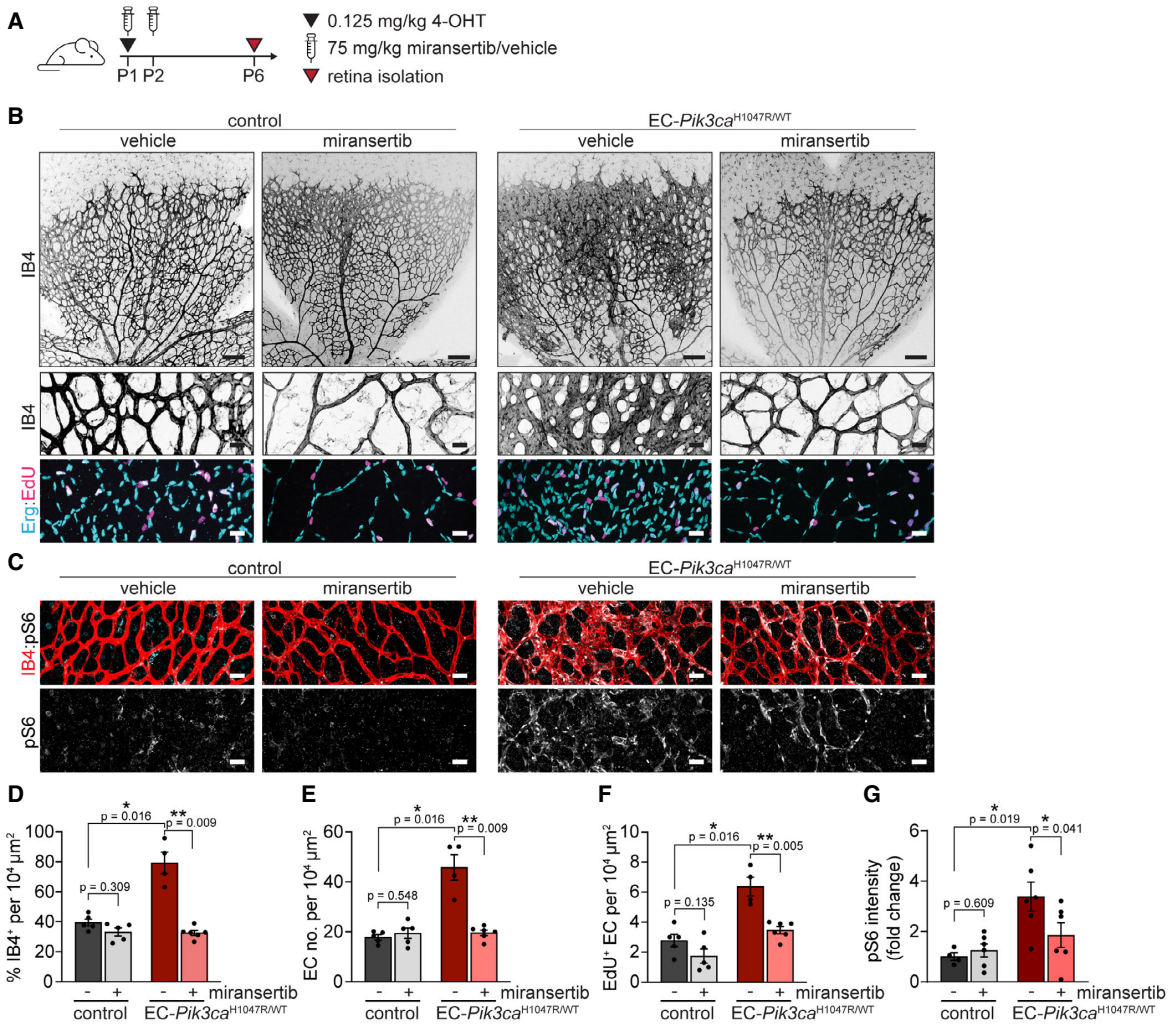
Miransertib prevents the formation of *Pik3ca*‐vascular malformations in mice A4‐OHT and miransertib dosing scheme used for a prevention therapeutic experimental setup.B, CRepresentative images of P6 retinas isolated from control and EC‐*Pik3ca^H1047R/WT^* mouse littermates. Blood vessels were stained with IB4. Lower panels showing high magnification images of the representative areas showing (B) blood vessels (IB4), EC nuclei (Erg), EdU incorporation and (C) pS6 (S235/236). Scale bars: 150 μm (upper panel) and 30 μm (lower panels).D–GQuantification of (D) retinal vascularity by IB4 staining, (E) EC number by Erg immunostaining, (F) EC proliferation by EdU staining, and (G) pS6 (S235/236) intensity (presented as a fold change of vehicle‐treated control). Bars represent the mean ± SEM. *n* ≥ 4 retinas per genotype. Statistical analysis was performed by nonparametric Mann–Whitney test. *P < 0.05 and **P < 0.01 were considered statistically significant. Source data are available online for this figure.

**Figure EV3:**
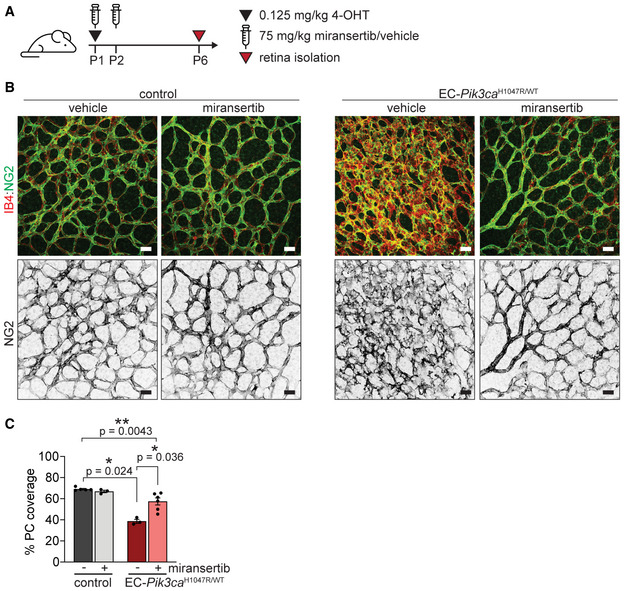
Miransertib prevents from loss of pericyte coverage 4‐OHT and miransertib dosing scheme used for a prevention therapeutic experimental setup.Representative images of P6 retinas isolated from control and EC‐*Pik3ca* ^H1047R/WT^ mouse littermates. Blood vessels were stained with IB4 and NG2 immunostaining was used to visualize pericytes. Scale bars: 30 μm.Quantification of vessel coverage by pericytes. Data presented as a percentage. Error bars are SEM. *n* ≥ 3 retinas per genotype. Statistical analysis was performed by nonparametric Mann–Whitney test. *P < 0.05 and **P < 0.01 was considered statistically significant. Source data are available online for this figure.

Targeting AKT, the main player of PI3K‐driven signalling in ECs has not yet been assessed *in vivo* in vascular malformations. First, we assessed PI3K/AKT signalling *in vitro* in *Pik3ca^H1047R^* ECs upon AKT inhibition with miransertib. P‐Akt and p‐Pras40 levels were reduced in a dose‐response manner (Appendix Fig S2A). In line with this, 24 h after miransertib treatment (2 µM), expression levels of Ang2 and specific cell cycle regulators found deregulated upon *Pik3ca^H1047R^* expression were normalized (Appendix Fig S2B–D). Next, we took advantage of our unique *in vivo* model to examine the impact of miransertib in the dynamic pathophysiology of PI3K‐driven vascular malformations. Previous preclinical studies of miransertib using tumour xenografts showed that the minimum dose that has an impact on tumour volume is 75 mg/kg (Yu *et al*, 2017); thus, we first evaluated this dose to assess miransertib for preventing the formation of *Pik3ca*‐driven vascular malformations. For this, we treated P1 EC‐*Pik3ca^H1047R^* mice with 4‐OHT and we dosed these mice with either 75 mg/kg of miransertib or vehicle at P1 and P2, followed by the analysis of P6 retinas (Fig 5A). Miransertib prevented the formation of vascular malformations, assessed by the vascular area and total EC number (Fig 5B, D and E) by inhibiting *Pik3ca^H1047R^*‐driven EC hyperproliferation assessed by EdU incorporation (Fig 5B and F). Also, the treatment partially prevented the loss of NG2‐positive mural cell coverage (Fig EV3). By pS6 immunostaining, we confirmed that miransertib treatment inhibited PI3K signalling in the vasculature (Fig 5C and G). Of note, wild‐type control retinas treated with miransertib showed a slight impact on vasculature density and EC proliferation (Fig 5B, D and F) confirming a key role of AKT in angiogenesis and vascular homeostasis (Ackah *et al*, 2005; Chen *et al*, 2005; Kerr *et al*, 2016).

**Figure 6:**
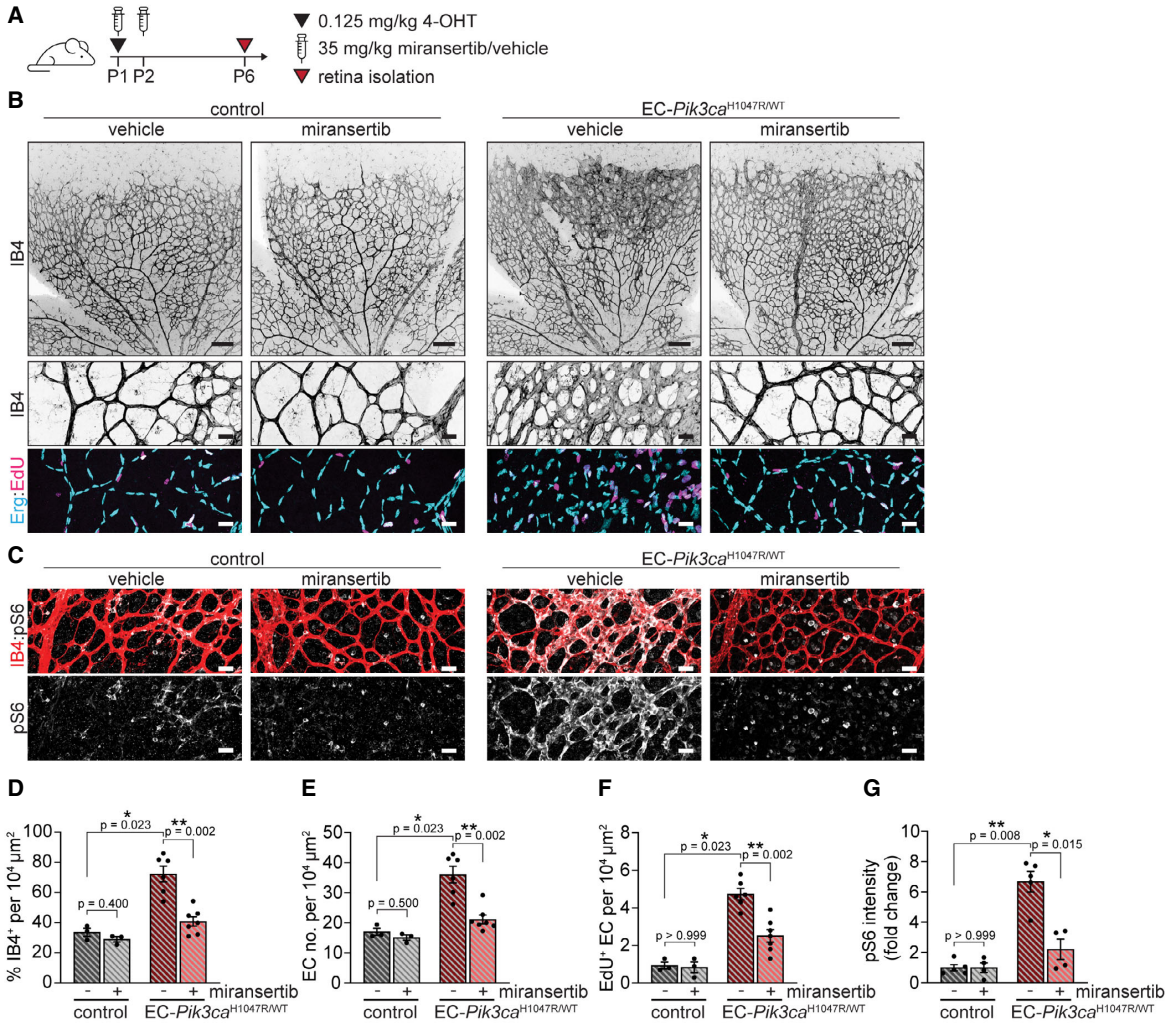
Low dose of miransertib prevents the growth of *Pik3ca*‐vascular malformations A4‐OHT and miransertib dosing scheme used for a prevention experimental setup.B, CRepresentative confocal images of P6 retinas isolated from control and EC‐*Pik3ca^H1047R/WT^* mouse littermates. Blood vessels were stained with IB4. Lower panels showing high magnification images of the representative areas showing (B) blood vessels (IB4), EC nuclei (Erg), EdU incorporation and (C) pS6 (S235/236). Scale bars: 150 μm (upper panels) and 30 μm (lower panels).D–GQuantification of (D) retinal vascularity by IB4 staining, (E) EC number by Erg immunostaining, (F) EC proliferation by EdU staining, and (G) pS6 (S235/236) intensity (presented as a fold change of vehicle‐treated control). Bars represent the mean ± SEM. *n* ≥ 3 retinas per genotype. Statistical analysis was performed by nonparametric Mann–Whitney test. *P < 0.05 and **P < 0.01 were considered statistically significant. Source data are available online for this figure.

Molecularly targeted treatment of vascular malformations may require long‐term, even chronic, therapeutic approaches in paediatric patients. This points to the importance of identifying the minimal effective dose of any candidate treatment. Thus, we next asked whether reducing the dose of miransertib would provide similar efficacy and reduce knock‐on effects on normal vasculature in our preclinical model. To test this, we reduced miransertib dose by half (35 mg/kg) and evaluated its efficacy on preventing *Pik3ca*‐driven vascular malformations (Fig 6A). We observed that the therapeutic efficacy of AKT inhibition by miransertib was maintained at the lower dose, with decreased EC proliferation leading to reduced number of total ECs and vascular density in EC‐*Pik3ca^H1047R^* miransertib‐treated retinas compared with vehicle‐treated counterparts (Fig 6B and D–F). This dose of miransertib efficiently inactivated PI3K signalling as shown by reduced pS6 levels (Fig 6C and G). Importantly, this dose had no impact on non‐mutant vasculature (Fig 6B–G). Altogether, our data demonstrate that miransertib efficiently inhibits the formation of PI3K‐driven vascular malformations and that lower doses than reported for oncological purposes of miransertib appear equally effective for preventive strategies.

### Treatment with miransertib induces the regression of PI3K‐driven vascular malformations

**Figure 7:**
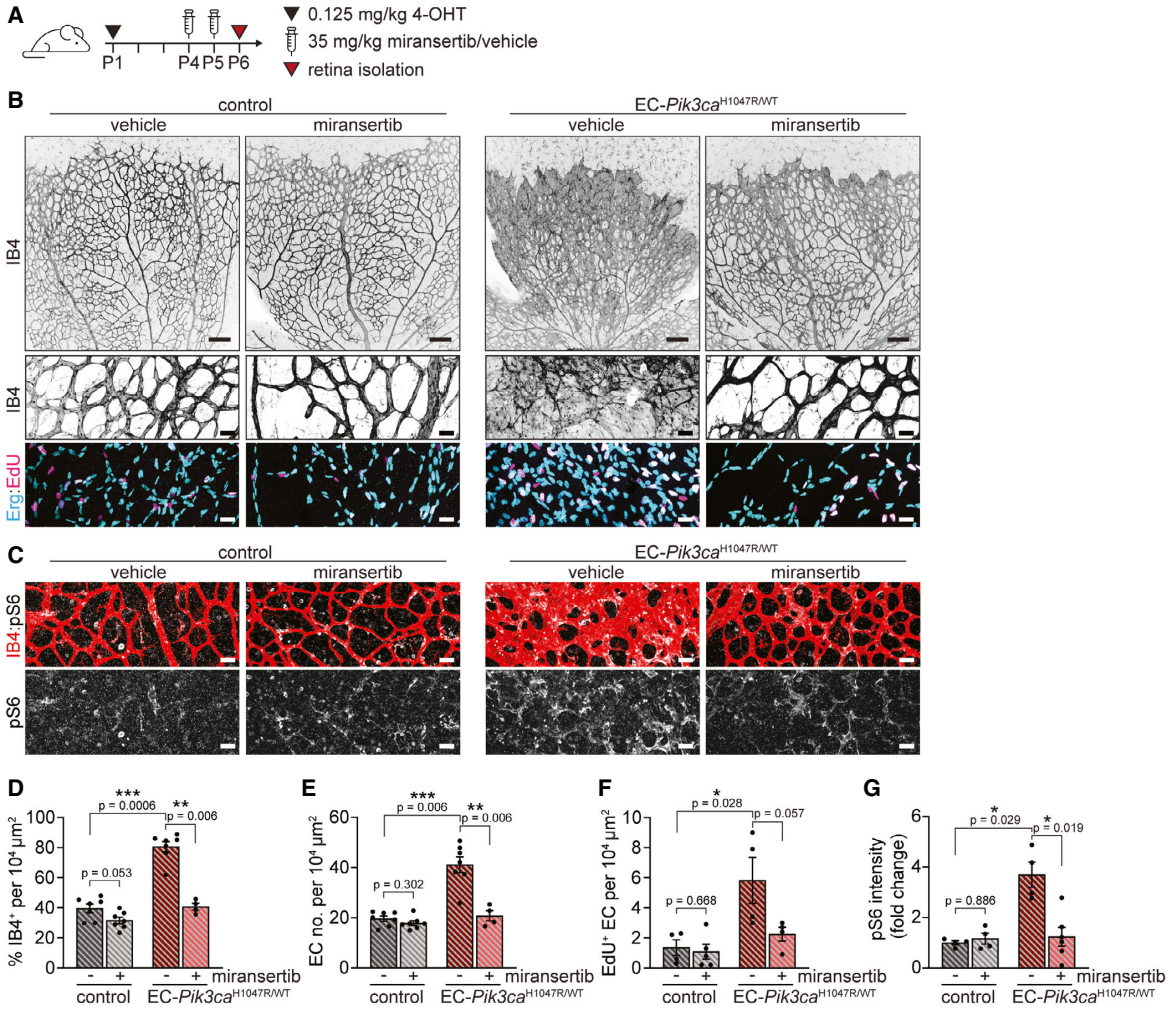
Low‐dose miransertib induces regression of *Pik3ca*‐vascular malformations *in vivo* A4‐OHT and miransertib dosing scheme used for a curative experimental setup.B, CRepresentative confocal images of P6 retinas isolated from control and EC‐*Pik3ca^H1047R/WT^* mouse littermates. Blood vessels were stained with IB4. Lower panels showing high magnification images of the representative areas showing (B) blood vessels (IB4), EC nuclei (Erg), EdU incorporation and (C) pS6 (S235/236). Scale bars: 150 μm (upper panels) and 30 μm (lower panels).D–GQuantification of (D) retinal vascularity by IB4 staining, (E) EC number by Erg immunostaining, (F) EC proliferation by EdU staining, and (G) pS6 (S235/236) intensity (presented as a fold change of vehicle‐treated control). Bars represent the mean ± SEM. *n* ≥ 4 retinas per genotype. Statistical analysis was performed by nonparametric Mann–Whitney test. *P < 0.05, **P < 0.01 and ***P < 0.001 were considered statistically significant. Source data are available online for this figure.

Since vascular malformations are congenital diseases, therapeutic interventions should aim for inducing the regression of mass lesions. Indeed, we assessed the therapeutic effect of miransertib on PI3K‐driven vascular malformations. For this, we first induced the formation of vascular malformations by treating EC‐*Pik3ca^WT/H1047R^* mice with 4‐OHT at P1 and then treated these mice with 35 mg/kg (low dose) of miransertib at P4 and P5 (Fig 7A). As shown above, in our *in vivo* preclinical model, vascular malformations are already present at P4, showing all hallmarks of PI3K‐driven vascular malformations (Fig EV2). In contrast to vehicle‐treated, miransertib‐treated EC‐*Pik3ca^H1047R^* retinas effectively regressed vascular malformations, confirmed by the normalization of vascular density, EC number and proliferation rate (Fig 7B and D–F). Importantly, elevated PI3K signalling induced by *Pik3ca^H1047R^* was blunted by miransertib (Fig 7C and G). These data demonstrate that miransertib is effective in inducing regression of PI3K‐driven vascular malformations, thereby setting the grounds for a promising clinical strategy for these diseases.

### Miransertib inhibits PI3K/AKT signalling and reduces cell viability in patient‐derived PIK3CA‐ and TEK‐mutant ECs

**Figure 8:**
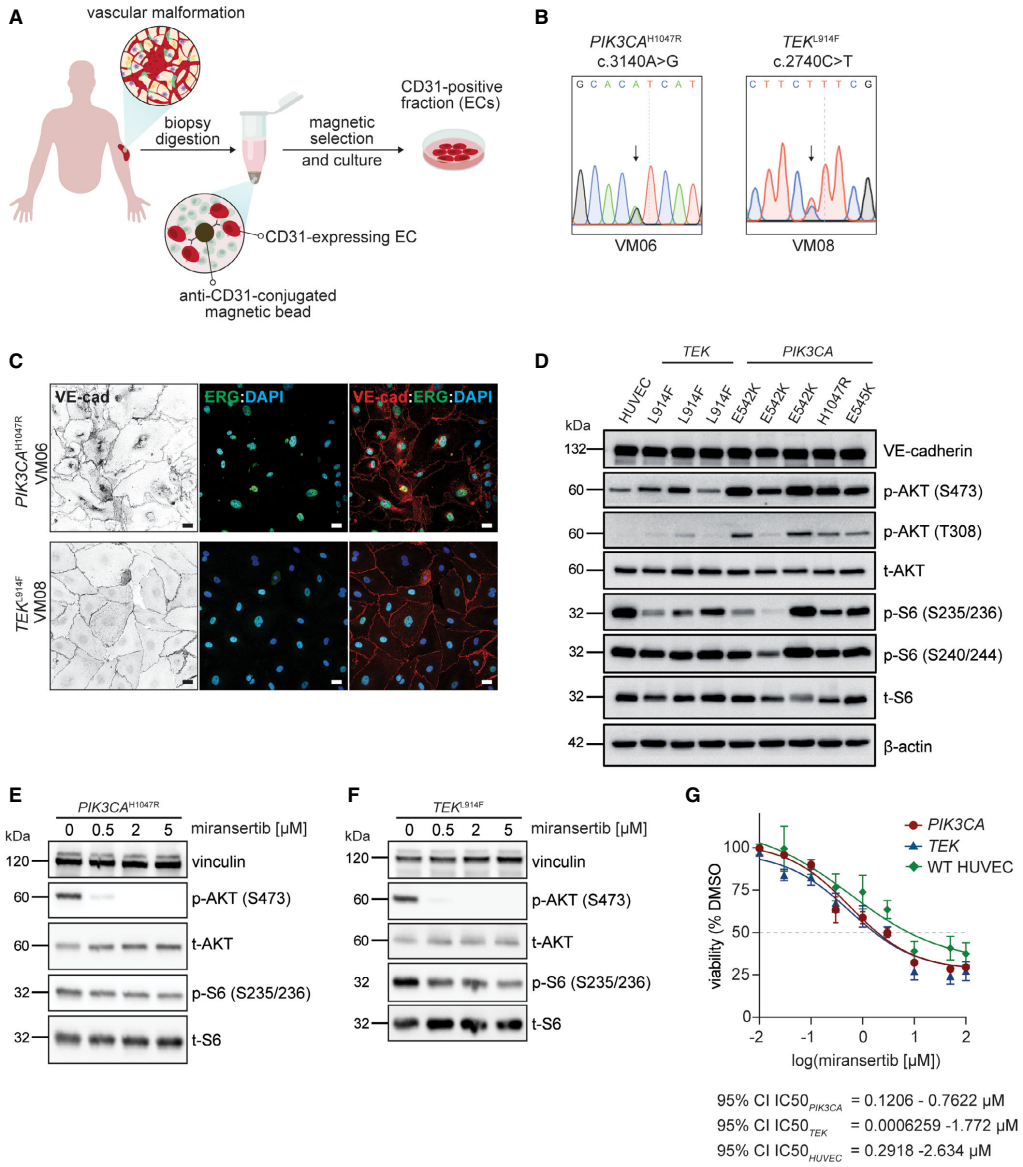
Miransertib impairs cell viability of *PIK3CA*‐ and *TEK*‐mutant patient‐derived ECs AIllustration showing the strategy of EC isolation from patient‐derived biopsies.BRepresentative sequencing chromatograms of *PIK3CA* and *TEK*‐mutant VM‐derived ECs. Arrows show the detected point mutations.CRepresentative confocal images of *PIK3CA* and *TEK* patient‐derived ECs immunostained for VE‐cadherin (EC‐specific junctional protein) and ERG (EC‐specific transcription factor). Cell nuclei were visualized with DAPI. Scale bars: 30 μm.DImmunoblot showing the activation of PI3K/AKT/mTORC1 pathway (by assessing the levels of p‐AKT and p‐S6) among different *PIK3CA* and *TEK* patient‐derived ECs. Primary HUVEC were used as wild‐type control.E, FImmunoblot showing the impact of miransertib at increasing doses on PI3K/AKT/mTORC1 pathway (by assessing p‐AKT and p‐S6 levels) in (E) *PIK3CA* and (F) *TEK*‐mutant patient‐derived ECs.GWild‐type (HUVECs), *PIK3CA* and *TEK*‐mutant EC viability upon the treatment with miransertib for 72 h at different doses assessed by MTS assay. Fitting curves and 95% CI IC50 values for wild‐type (HUVECs), *PIK3CA* and *TEK* ECs are shown. For *PIK3CA* and *TEK*‐mutant ECs *n* = 3 biological replicates (patients); for wild‐type ECs (HUVECs) *n* = 4 technical replicates. *PIK3CA*‐mutant versus *TEK*‐mutant *P* = 0.501; *PIK3CA*‐mutant versus HUVECs *P* = 0.0815; *TEK*‐mutant versus HUVECs: *P* = 0.4085. Statistical analysis was performed by comparison of best‐fit values using the extra sum‐of‐squares *F* test. Source data are available online for this figure.

Next, we assessed the therapeutic potential of miransertib in a preclinical human setting. For this, we isolated and cultured ECs derived from human vascular malformations carrying mutations in *PIK3CA* and *TEK/TIE2* (Appendix Table S2), together spanning the genetic causes of more than 80% of low‐flow lesions in patients (Limaye *et al*, 2009, 2015; Castel *et al*, 2016; Castillo *et al*, 2016a). To isolate and culture these cells, fresh surgical resections of low‐flow vascular malformations were subjected to tissue digestion and EC‐positive selection (Fig 8A). Sanger sequencing and droplet digital PCR revealed that causing mutations were present in the EC culture with variant allelic frequencies (VAFs) of around 50% (Fig 8B; Appendix Fig S3A and B), demonstrating that these cell cultures were enriched in mutant cells. Characterization of these cells validated their specific EC properties—cobblestone morphology and expression of the EC‐specific markers VE‐cadherin and ERG (Fig 8C; Appendix Fig S3C). Next, we analysed the impact of these mutations in PI3K signalling by assessing AKT and S6 phosphorylation levels. Indeed, *PIK3CA*‐ and *TIE2*‐mutant ECs exhibited constitutive activation of the pathway compared with wild‐type HUVECs (Fig 8D). To evaluate the therapeutic efficacy of miransertib in patient‐derived *PIK3CA* and *TEK*‐mutant ECs, we first assessed its impact on PI3K/AKT signalling. At very low doses, miransertib strongly inhibited AKT signalling in wild‐type and mutant ECs, shown by reduced levels of AKT and S6 phosphorylation in a dose‐dependent manner (Fig 8E and F; Appendix Fig S3D). This effect was similar in both *PIK3CA* and *TEK*‐mutant ECs (Fig 8E and F). We then studied the functional impact of miransertib‐mediated AKT signalling inhibition by determining the dose‐response effect on cell viability in ECs with either *PIK3CA*‐ or *TEK*‐mutant genotype and wild‐type HUVECs. Miransertib robustly decreased EC viability in all genotypes showing low IC_50_ with overlapping (not significantly different) confident intervals (Fig 8G). Interestingly, cell viability of wild‐type ECs was slightly less sensitive to miransertib treatment. To assess whether this *in vitro* effect is reproduced by other AKT inhibitor, we assessed cell viability after treating wild‐type and *PIK3CA* and *TEK*‐mutant ECs with the AKT inhibitor MK2206 (Appendix Fig S3E). The impact of MK2206 on EC viability is significantly reduced in both *PIK3CA* and *TEK*‐mutant genotypes compared with miransertib treatment. Moreover, wild‐type ECs are slightly more sensitive to MK2206 treatment than mutant ECs.

These data show that miransertib impacts the viability of patient‐derived ECs at low concentrations and that it might constitute a promising therapeutic strategy for both *PIK3CA* and *TEK*‐mutant vascular malformations.

## Discussion

Activation of the PI3K/AKT signalling pathway by genetic mutations in the endothelium is the primary etiological cause of most, if not all, low‐flow vascular malformations (Boscolo *et al*, 2015; Limaye *et al*, 2015; Luks *et al*, 2015; Castel *et al*, 2016; Castillo *et al*, 2016a). Despite of this, there is no molecular targeted therapy approved for their clinical management today. This is partially due to poor biological understanding of PI3K overactivation in ECs. Here, we provide the first evidence that endothelial expression of a hotspot *Pik3ca* mutation in these diseases triggers the activation of early molecular programmes involved in cell cycle progression and cell proliferation; thus, shedding light into how these lesions form. The precise mechanism by which *Pik3ca^H1047R^* leads to enhanced cell cycle progression still remains unclear. Nevertheless, our results show that pathological expansion of *Pik3ca^H1047R^* ECs primary occurs in the presence of growth factors. Given that pS6, a downstream effector of AKT/mTOR, is selectively activated in the presence of growth factors in mutant cells, it suggests that the AKT/mTOR axis orchestrates the onset of *Pik3ca^H1047R^*‐vascular malformations.

Using the postnatal mouse retina as a model of vascular malformations in combination with a tamoxifen‐induced strategy, which allows to mosaically express *Pik3ca^H1047R^* at different developmental times, we demonstrate that active angiogenesis is required for *Pik3ca* mutants to generate vascular malformations. In line with this, cultured patient‐derived *TEK* or *PIK3CA*‐mutant ECs lead to pathological proliferative response only upon stimulation of growth signals (Cai *et al*, 2019; Le Cras *et al*, 2020). Also, *in vivo*, other types of PI3K‐related vascular malformations such as *Pik3ca*‐driven lymphatic malformations and cerebral cavernous malformations, and hereditary haemorrhagic telangiectasia‐like arteriovenous malformations rely on growth factor signals to be induced and expand (Garrido‐Martin *et al*, 2014; Martinez‐Corral *et al*, 2020; Mäkinen *et al*, 2021; Ren *et al*, 2021). The growth‐push concept fits with clinical observations where low‐flow vascular malformations appear during embryonic development, expand proportionally with the physiological growth of the patient and are largely quiescent during adulthood, when the production of growth factors is residual (Pang *et al*, 2020). This also explains why low‐flow vascular malformations are histopathologically characterized by a low rate of EC proliferation. However, acute production of extrinsic inputs such as hormonal changes, injury and wound healing can reactivate proliferation in the malformed vasculature (Pang *et al*, 2020); being a key aspect to mind for preventive approaches.

Another important observation of our model is that *Pik3ca*‐related blood vascular malformations only occur in veins and in the capillary bed, excluding arteries which remain unaltered. This resembles the human spectrum of vascular malformations where *PIK3CA* mutations have not been reported in arterial malformations (Peyre *et al*, 2021; Ren *et al*, 2021). Why arteries are unaffected by *Pik3ca* mutation remains unclear. This could be related to the refractory behaviour that arterial cells exhibit upon reaching their definitive maturation state (Orsenigo *et al*, 2020; Luo *et al*, 2021), and the cell cycle arrested state which fluid shear stress imposes on arterial ECs (Fang *et al*, 2017). While this would indicate that flow overrules high PI3K signalling, further experiments are required to address this.

Inhibition of the PI3K/AKT signalling pathway has been prioritized in targeted therapy strategies in medical oncology (Castel *et al*, 2021; Vanhaesebroeck *et al*, 2021). At least in part, the lack of robust and rapid preclinical models for drug evaluation has hampered the field. In this study, we provide evidence that the mouse retina model offers an opportunity for preclinical drug testing of blood vascular disorders. The key aspects of this model are as follows: (i) it develops in a short time frame (1 week); (ii) it allows for a dynamic preclinical testing, which covers prevention and curative strategies; (iii) it grants the quantification of disease hallmarks including vascularity, EC proliferation and PI3K signalling, thus providing a robust and non‐biased setting to evaluate treatment efficacy; (iv) it recapitulates relevant features of vascular malformations microenvironment including the impact of blood flow, extracellular matrix, surrounding pericytes and smooth muscle cells, which may influence drug response. Of relevance, this experimental system has been previously used for targeted therapy validation in other vascular disorders (Ola *et al*, 2016, 2018; Alsina‐Sanchis *et al*, 2018). However, this is not a systemic model, and thus, it lacks some important aspects of the disease such as thrombosis, haemorrhages, phlebectasia or infection (Pang *et al*, 2020). We have previously modelled systemic vascular malformations by embryonic expression of *Pik3ca^H1047R^* mutation in mice recapitulating some of these knock‐on effects (Castillo *et al*, 2016a). This preclinical model showed that therapeutic reduction of vascular malformation may also eliminate and relieve some of these clinical manifestations such as haemorrhages or phlebectasia of main internal veins (Castillo *et al*, 2016a). Nevertheless, the high heterogeneity of this model and the long‐time frame needed to assess therapeutic effectivity are important drawbacks for preclinical studies of molecular therapies.

Blocking AKT signalling has been a promising therapy for cancer; however, rewiring PI3K signalling due to the complex genetic landscape and instability of malignant cells have dampened these expectations (Jansen *et al*, 2016). In contrast, vascular malformations are considered largely monogenic diseases caused by activating point mutations in an otherwise stable genetic context (Castel *et al*, 2020). Our data indicate that, similar to alpelisib, a selective p110α inhibitor, low‐dose miransertib may be sufficient to prevent and induce regression of these diseases (Venot *et al*, 2018). This is in line with previous studies reporting the effect of miransertib in *PIK3CA*‐mutant ECs (Boscolo *et al*, 2019). Our results might indicate that this therapy is also effective for *TEK*‐mutant vascular malformations; however, the lack of *TEK*‐mutant mouse lines precludes the assessment *in vivo*. In addition, comparison of different PI3K signalling pathway inhibitors may contribute to the refinement of therapeutic strategies for these diseases. Since these are early days for patient stratification for PI3K‐related disorders, preclinical assessment of new molecular drugs is critical to maximize therapeutic opportunities for these patients. Indeed, these patients may need long‐term or even chronic treatment; thus alternation therapies over time using effective low dose of inhibitor may be required to avoid side effects and the development of drug resistance. We anticipate that these results will have a direct impact on future clinical strategies.

Collectively, our data suggest that blocking EC growth should be considered during potential reactivation scenarios. Furthermore, our study calls for revisiting current classification of low‐flow vascular malformation, which should be considered proliferative vascular disorders.

## Materials and Methods

### Reagents

All chemical reagents were purchased from Sigma‐Aldrich, unless stated otherwise. Cell culture media and buffers were purchased from Lonza and Gibco. Primers were obtained from Invitrogen. Normal Primary Umbilical Vein Endothelial Cells (HUVECs) were purchased from ATCC (PCS‐100‐010).

### Mice

The *in vivo* experiments were performed in agreement with the guidelines and legislations of the Catalan Ministry of Agriculture, Livestock, Fisheries and Food (Catalonia, Spain), following protocols approved by the local Ethics Committees of IDIBELL‐CEEA (animal Use Protocol number 6809). Mice were kept in individually ventilated cages under specific pathogen‐free conditions (IDIBELL Animal Facility). All mice were crossed onto the C57BL/6J genetic background. *Pik3ca* ^WT/H1047R^ mice carry a single, germline Cre‐inducible point mutation in *Pik3ca* allele (H1047R) (Kinross *et al*, 2012). This mice are crossed onto *Pdgfb*‐iCreER mice (Claxton *et al*, 2008) that express an inducible iCreER recombinase from the endogenous *Pdgfb* locus (EC‐specific). Control mice were CreiER‐negative littermates injected with 4‐hydroxytamoxifen. iCreER‐mediated recombination in *Pik3ca* ^WT/H1047R^ mice was induced by intraperitoneal injection of 4‐hydroxytamoxifen (doses indicated in the figure legends). ROSA‐mTmG double fluorescent reporter mouse (Muzumdar *et al*, 2007) was crossed to *Pdgfb*‐iCreER mice. The ROSA‐mTmG allele was kept heterozygous. 4‐Hydroxytamoxifen was injected intraperitoneally in indicated doses (see the figure legends) and mouse retinas isolated at indicated time points. Cre‐mediated recombination was assessed by the expression of membrane‐bound GFP. For the analysis of mouse postnatal retinas, male and female mice were analysed; the gender of mice was not assessed given the difficulty to determine this by anatomical differences at postnatal age. For the RNAseq analysis, primary endothelial cells were isolated from two female and two male mice. For the rest of experiments, primary endothelial cells were isolated from male mice.

### Mouse ECs isolation and culture

Mouse lung ECs were isolated from adult mice between 3 and 6 weeks old. Briefly, lungs were homogenized with a scalpel blade and incubated in dispase II in Hank's Balanced Salt Solution at 4 U/ml concentration for 1 h at 37°C. The digested tissue was disintegrated by pipetting into a single‐cell solution, following enzyme inactivation with DMEM supplemented with 10% FBS and 1% penicillin/streptomycin. Cells were resuspended in 100 µl of PBS and incubated with rat anti‐CD‐144 (BD Biosciences, 555289) antibody‐coated magnetic beads for 30 min at room temperature. CD‐144‐positive fraction was washed with PBS with 0.5% BSA and sorted using a magnet. Cells were resuspended and cultured in 0.5% gelatine‐coated culture well (12‐well format) in F12/DMEM medium supplemented with 20% FBS, 1% penicillin/streptomycin and 4 ml EC growth factors, later referred to as F12 complete until they reached 80–90% confluency. Cells were subjected for a second selection with the CD‐144 antibody‐coated magnetic beads for 1 h at room temperature, then trypsinized, magnetic‐sorted and resuspended in F12 complete medium and further cultured (6‐well format). Cells were cultured at 37°C in 5% CO_2_ atmosphere until passage 5. Primary cells were not tested for mycoplasma contamination. To induce *Pik3ca^H1047R^* mutation, ECs were treated with 2 µM 4‐hydroxytamoxifen for 6 h. Ethanol‐treated cells were used as a control. Recombined cells were re‐seeded for an experiment and cultured at 37°C in 5% CO_2_ atmosphere.

### Protein extraction and immunoblotting

Cells were lysed in ice‐cold lysis buffer (50 mM Tris–HCl pH 7.4, 5 mM EDTA, 150 mM NaCl and 1% Triton X‐100) containing protease (Roche, 11836153001) and phosphatase inhibitors (Sigma‐Aldrich, 4906837001). Total cell lysates were resolved on 10% polyacrylamide gels, transferred onto nitrocellulose membranes and incubated with appropriate primary and secondary antibodies. The following primary antibodies were used: p‐AKT S473 (CST, 4060, diluted 1:1,000), p‐AKT T308 (CST, 4056S, diluted 1:500), AKT (CST, 9272, diluted 1:2,000), p‐PRAS40 T246 (CST, 2640, diluted 1:1,000), p‐S6 S235/236 (CST, 4857, diluted 1:1,000), p‐S6 S240/244 (CST, 2215S, diluted 1:1,000), S6 (CST, 2212, diluted 1:1,000), VE‐cadherin (Santa Cruz Biotechnology, sc‐6458, diluted 1:500) and vinculin (Abcam, ab49900, diluted 1:10,000). The following secondary antibodies from DAKO were used (all diluted 1:5,000): swine anti‐rabbit (P0399), rabbit anti‐goat (P0449), rabbit anti‐mouse (P0260) and rabbit anti‐sheep (P0163).

### cDNA synthesis and quantitative polymerase chain reaction

RNA was extracted using RNeasy Qiagen kit following manufacturer instructions. Reverse transcription was performed from 250 ng of RNA for cell lysate samples by using High‐Capacity cDNA Reverse Transcription Kit. For quantitative polymerase chain reaction, a LightCycler 480 System was used with LightCycler 480 SYBR Green I Master kit and specific primers detailed in Appendix Table S3. *Rpl32* gene (coding for the 60S ribosomal protein L32) was used for gene expression normalization.

### RNA sequencing and analysis

The quantity and quality of the total RNA sample were determined by Qubit RNA BR Assay kit (Thermo Fisher Scientific) and RNA 6000 Nano Bioanalyser 2100 Assay (Agilent). The RNASeq libraries were prepared following the TruSeq Stranded mRNA Sample Prep Kit protocol. Briefly, total RNA (500ng) was enriched for the polyA mRNA fraction and fragmented by divalent metal cations at high temperature. In order to achieve the directionality, the second strand cDNA synthesis was performed in the presence of dUTP. The blunt‐ended double‐stranded cDNA was 3′adenylated and Illumina Truseq adaptors with single indexes were ligated. The ligation product was enriched with 15 PCR cycles, and the final library was validated on an Agilent 2100 Bioanalyser with the DNA 7500 assay (Agilent). For the analysis of RNAseq data, generated fastq files were first quality controlled using FASTQC software (version 0.11.9). Then, these were aligned to the UCSC mm10 reference genome using STAR (version 2.7.2b) (Dobin *et al*, 2013). Qualimap (version 2.2) (Okonechnikov *et al*, 2016) was used for mapping quality control and IGV (version 2.3.86) (Thorvaldsdóttir *et al*, 2013) for visualization of the aligned reads. Mapped counts were quantified with featureCounts (Liao *et al*, 2014) from the SubRead R package (Liao *et al*, 2019) using the UCSC mm10 gene annotation, and normalization was performed with the DESeq2 R package (version 3.10) (Love *et al*, 2014). Differential genes expression was also analysed using DESeq2, with 0.05 as the significance cut‐off, and the obtained *P*‐values were corrected for multiple testing using the Benjamini and Hochberg method. Gene set enrichment analysis (GSEA, Subramanian *et al*, 2005) was performed on the log 2 fold change ranked list of genes in our dataset using the fgsea R package (preprint: Korotkevich *et al*, 2021) with Msigdb (Liberzon *et al*, 2011) and clusterProfiler (Yu *et al*, 2012) with BP Gene Ontology terms (Ashburner *et al*, 2000). Volcano plot in Fig 1B was created with VolcaNoseR (Goedhart & Luijsterburg, 2020).

### Cell cycle analysis by flow cytometry

For cell cycle analysis, 10^6^ cells per condition were harvested, washed in PBS and fixed by adding dropwise 0.9 ml ice‐cold 70% ethanol while vortexing and stored 24 h at −20°C. On the day of analysis, cells were spun down at 300g 5 min and washed with PBS. Cell pellet was stained 30 min at RT using FxCycle PI/RNase Staining solution (Life Technologies F10797) (0.5 ml per sample). Cell cycle was assessed using BD FACSCanto^TM^ II flow cytometer, and data were analysed using the FlowJo^TM^ software v10.8.0 (BD Life Sciences).

### Pharmacological *in vivo* treatment

Miransertib (ARQ 092·2MSA salt) (ArQule, Inc., a wholly owned subsidiary of Merck & Co., Inc., Kenilworth, NJ, USA) was prepared at a stock concentration of 10 mgA/ml in 20% Captisol (m/v) in 0.02 M citrate/saline buffer. 20% Captisol (m/v) in 0.02 M citrate/saline buffer was used as a vehicle. Mice were injected intraperitoneally with either 75 mg/kg or 35 mg/kg dose of miransertib.

### Mouse retina isolation and immunostaining

Mice were sacrificed by decapitation and eyes were isolated, followed by an hour incubation on ice in 4% PFA in PBS. Isolated retinas were fixed for additional hour, permeabilized overnight at 4°C in permabilization/blocking buffer (1% BSA, 0.3% Triton X‐100 in PBS). Afterwards, the retinas were incubated overnight at 4°C with specific primary antibodies, diluted in permabilization/blocking buffer (ERG (Abcam, AB92513, diluted 1:400), NG2 (Milipore, AB5320, diluted 1:200), p‐S6 235/236 (Cell Signalling Technology, 4857, diluted 1:100). Samples were washed three times in PBS containing 1% Tween‐20 (PBST), following incubation with PBlec buffer (1% Triton X‐ 100, 1 mM CaCl_2_, 1 mM MgCl_2_ and 1 mM MnCl_2_ in PBS, pH 6.8) for 30 min at RT. Secondary AlexaFluor‐conjugated antibodies, diluted in PBlec, were added to the retinas and incubated for another 2 h (Invitrogen, A11001, A11011, A11008 and A31573). Blood vessels were visualized with AlexaFluor‐conjugated Isolectin GS‐B4 (Molecular Probes, I21411, I21412). Following three washes with PBST, the tissues were flat‐mounted on a microscope slide.

### *In vivo* proliferation assay

5‐ethynyl‐2′‐deoxyuridine (EdU)‐incorporation assay has been performed using a commercially available kit (Invitrogen, C10340). Animals were injected intraperitoneally with 60 µl of EdU (0.5 mg/ml in 50% DMSO and 50% PBS solution), and after 2 h, the animals were sacrificed and retinas isolated. EdU incorporation was detected with Click‐iT EdU Alexa Fluor‐647 Imaging Kit, following manufactures instructions. Afterwards, standard protocol for retina immunostaining was applied.

### Confocal imaging and image quantification

Microscopy imaging was done with Leica TCS SP5 confocal microscope. Volocity, Adobe Photoshop 2021 and ImageJ softwares were used for image editing and quantification, respectively. Images were taken from at least 4 retina areas in each genotype. At least three biological replicates per genotype were performed. To quantify the vascular lesion area, an IB4‐positive area was manually selected and the percentage of IB4 area per retina area was quantified. To determine the recombination efficiency of mTmG allele in ECs, the ratio of GFP‐positive area to IB4‐positive area was calculated and presented as percentage. Retina vascularity was measured using IB4 channel by adjusting the threshold to select the IB4‐positive area, followed by quantification of the percentage of IB4‐positive area in the total image area (10^4^ µm^2^). EC number was determined manually based on EC‐specific nuclei staining (Erg) in 10^4^ µm^2^ image area. Quantification of EC proliferation was done using EdU and Erg co‐immunostaining—both EdU‐ and Erg‐positive ECs were quantified in the 10^4^ µm^2^ image area. The coverage of vessels by NG2‐positive pericytes was quantified from both NG2 and IB4 channels by adjusting the threshold and selecting the positive NG2 and IB4 areas, respectively. Then, the percentage of NG2 to IB4 ratio was calculated. The vascular‐specific p‐S6 intensity was measured using both p‐S6 and IB4 channels. First, a manual threshold was set to obtain the IB4‐positive area and define the region of interest (ROI). Then, the integrated density of p‐S6 was measured in IB4‐positive areas. The background measurements (mean grey values) were taken from areas in close proximity to the vasculature, but negative for IB4. The corrected total fluorescence (CTF) was calculated based on the following equation: CTF = integrated density—(vascular area × mean grey background value). Veins, arteries and capillaries in the mouse postnatal retina are identified by morphology as previously described (Pitulescu *et al*, 2010).

### Isolation, culture and sequencing of ECs from patient‐derived vascular malformations

Patient tissue samples were obtained under therapeutic surgical resection. Informed consent was obtained from all subjects with the approval of the Committees on Biomedical Investigation at Hospital Sant Joan de Deu, Hospital Santa Creu i Sant Pau and Hospital Universitari Bellvitge (Barcelona, Spain) under the codes PR264/16 and PIC‐96‐16. Experiments conformed to the principles set out in the WMA Declaration of Helsinki and the Department of Health and Human Services Belmont Report. Collected data were stored in a secure database maintained by Hospital Sant Joan de Deu. Human ECs were isolated from patient‐derived biopsies of vascular malformations. Briefly, the biopsy was homogenized with a scalpel blade and digested in dispase II (4 U/ml) and collagenase A (0.9 mg/ml) in Hank's Balanced Salt Solution for maximum 1.5 h at 37°C, vortexing the sample every 30 min. The digested tissue was disintegrated by pipetting into a single‐cell solution, following enzyme inactivation with DMEM supplemented with 10% FBS and 1% penicillin/streptomycin. Cells were resuspended in 100 µl of 0.5% BSA in PBS and incubated with mouse anti‐human CD31 (Agilent Dako, M0823, clone JC70A) antibody‐coated magnetic beads (ThermoFisher Scientific, 11041) for 1 h at room temperature. CD31‐positive fraction was washed with 0.5% BSA in PBS and sorted with a magnet. Cells were resuspended and cultured in 0.5% gelatine‐coated culture well (12‐well format) in EGM2 medium (PromoCell, C30140) supplemented with 10% FBS, 1% penicillin/streptomycin (later referred to as EGM2 complete) at 37°C and 5% CO_2_ until they reach confluency. Cells were subjected for a second selection. Primary cells were not tested for mycoplasma contamination. Genomic DNA was isolated according to the manufacturer’s protocol (Thermo Fisher Scientific, K182001). The regions of interest in the genomic DNA were amplified by PCR using Platinum™ Taq DNA Polymerase High Fidelity (Thermo Fisher Scientific, 11304011). Exons 10 and 21 of *PIK3CA*, and exon 17 of *TEK* were amplified. PCR products were purified according to the manufacturer’s protocol (GE Healthcare, 28‐9034‐70) followed by Sanger sequencing. Sequences of the primers used: *PIK3CA* exon 10: (forward) 5′‐TGGTTCTTTCCTGTCTCTGAAAA‐3′ and (reverse) 5′‐CCATTTTAGCACTTACCTGTGAC‐3′. *PIK3CA* exon 21: (forward) 5′‐CATTTGCTCCAAACTGACCA‐3′ and (reverse) 5′‐TGTGTGGAAGATCCAATCCA‐3′. *TEK* exon 17: (forward) 5′‐TAGGCAATTTCCACAGCACA‐3′ and (reverse) 5′‐GGCAAACCAGGCTAAGAGAG‐3′. Droplet digital PCR was done on genomic DNA extracted from cell cultures. *PIK3CA* genotyping assays from Bio‐Rad were used to specifically detect the *PIK3CA^E542K^* mutation on DNA samples. The Bio‐Rad QX200 ddPCR system was used, and allelic frequencies were calculated using Quantasoft Analysis Pro (BioRad) software.

### Cell immunofluorescence

Human VM‐derived ECs were seeded on gelatine‐coated coverslips in a way to reach confluency the next day and incubated overnight at 37°C in 5% CO_2_. Then, cells were washed with warm PBS with Mg^2+^ and Ca^2+^ and then fixed with 4% PFA for 15 min at room temperature, followed by triple wash with PBS with Mg^2+^ and Ca^2+^. Cells were permeabilized with PBS containing 0.4% Triton X‐100 for 5 min and blocked with 2% BSA in PBS for 1 h at RT. The following primary antibodies were used for 1 h at RT: VE‐cadherin F8 (Santa Cruz, SC‐9989, 1:100), ERG (Abcam, ab92513, 1:400). Then, coverslips were washed three times with PBS for 5 min, followed by 45 min incubation at RT with appropriate secondary antibody in PBS: goat anti‐mouse Alexa Fluor‐488 (Invitrogen A11001, diluted 1:300) and goat anti‐rabbit Alexa Fluor‐568 (Invitrogen A11011, diluted 1:300). Then, coverslips were washed with PBS three times for 5 min, and in the last wash DAPI (Invitrogen, D1306, diluted 1:10 000) was added to visualize cell nuclei. Coverslips were mounted on a microscope slide in a mounting medium (ThermoFisher Scientific, 9990402).

### MTS viability assay and calculation of IC50

MTS assay was used in order to determine cell viability. Briefly, 2·10^3^ cells were seeded on gelatine‐coated 96‐well plates (5 technical replicates per condition) and incubated overnight at 37°C in 5% CO_2_ atmosphere. The next day, cells were treated for 3 days with miransertib. MTS assay (Abcam, ab197010) was performed for 2.5 h, and the absorbance was measured at 490 nm. Data (the percentage of a vehicle) were plotted against the logarithm of inhibitor concentration. IC50 and 95% CI values were calculated by non‐linear regression (variable slope) using GraphPad Prism software.

### Statistics

Statistical analysis was performed using Prism 8 (GraphPad Software Inc.). All figures are displayed with individual data points that indicate biological replicates and with the standard error of the mean (SEM) as error bars. At least three biological replicates were used. *P*‐values considered as statistically significant were as follows: *P < 0.05; **P < 0.01 and ***P < 0.0001.

## Author contributions

**Piotr Kobialka:** Conceptualization; Data curation; Formal analysis; Investigation; Methodology; Writing—original draft; Writing—review & editing. **Helena Sabata:** Conceptualization; Data curation; Formal analysis; Investigation; Methodology; Writing—original draft; Writing—review & editing. **Odena Vilalta:** Methodology. **Leonor Gouveia:** Data curation; Formal analysis. **Ana Angulo‐Urarte:** Methodology. **Laia Muixi:** Methodology. **Jasmina Zanoncello:** Methodology. **Oscar Muñoz‐Aznar:** Methodology. **Nagore G Olaciregui:** Methodology. **Lucia Fanlo:** Methodology. **Anna Esteve‐Codina:** Data curation; Formal analysis. **Cinzia Lavarino:** Resources; Supervision. **Biola M Javierre:** Supervision; Methodology. **Veronica Celis:** Resources; Supervision. **Carlota Rovira:** Resources; Supervision. **Susana López‐Fernández:** Resources; Supervision. **Eulàlia Baselga:** Resources; Supervision. **Jaume Mora:** Resources; Supervision. **Sandra D Castillo:** Conceptualization; Data curation; Formal analysis; Supervision; Funding acquisition; Investigation; Methodology; Writing—original draft; Writing—review & editing. **Mariona Graupera:** Conceptualization; Resources; Data curation; Formal analysis; Supervision; Funding acquisition; Investigation; Writing—original draft; Writing—review & editing.

In addition to the CRediT author contributions listed above, the contributions in detail are:

MG, SDC, PK and HS were the main contributors in the conception, design, acquisition and interpretation of the data and in writing the article. PK HS, OV, AA‐U, LM, JZ, LF. NGO, CL, BMJ and SDC performed experiments and data analysis with input from SDC and MG. RNAseq analysis was performed by LG and AE‐C. CR and OM‐A interpreted histopathology. VC, SL‐F, EB and JM liaised with human subjects and provided access to human tissue samples and clinical input in the study.

## Disclosure and competing interests statement

M.G. has a research agreement with ArQule, Inc., a wholly owned subsidiary of Merck & Co., Inc., Kenilworth, NJ, USA, and Venthera. E.B. is founder and CAB of Venthera; PI and Advisor for Pierre Fabre; PI of the clinical trial NCT04589650 (Novartis).

## Supporting information



Appendix

Expanded View Figures PDF

Source Data for Expanded View and Appendix

Source Data for Figure 1

Source Data for Figure 2

Source Data for Figure 3

Source Data for Figure 4

Source Data for Figure 5

Source Data for Figure 6

Source Data for Figure 7

Source Data for Figure 8

## Data Availability

RNAseq data are deposited in the BioProject repository (NCBI) under the accession ID PRJNA780473 (https://www.ncbi.nlm.nih.gov/bioproject/PRJNA780473).
