# Supplementary material for: The onset of PI3K‐related vascular malformations occurs during angiogenesis and is prevented by the AKT inhibitor miransertib
Source: EMBO Mol Med. 2022 Jun 13;14(7):e15619. doi: 10.15252/emmm.202115619 (PMC9260211; doi:10.15252/emmm.202115619)

APPENDIX

Table of Content

Appendix Tables	
Appendix Table S1	GSEA analysis showing enriched hallmarks in Pik3ca^{H1047R} ECs.
Appendix Table S2	Clinical data from patients
Appendix Table S3	List of primers used for gene expression analysis
**Appendix Figures**	
Appendix Figure S1	Gene expression changes upon Pik3ca^{H1047R} expression in ECs.
Appendix Figure S2	Molecular rescue of Pik3ca^{H1047R} ECs upon miransertib treatment
Appendix Figure S3	Characterization of primary ECs isolated from patient-derived vascular malformations

Appendix Tables

Appendix Table S1. GSEA analysis showing enriched hallmarks in Pik3ca^{H1047R} ECs.

GS	SIZE	ES	NES	NOM p-val	FDR q-val	FWER p-val	RANK AT MAX	LEADING EDGE
HALLMARK_E2F_TARGETS	197	0.7654796	3.09031	0.0	0.0	0.0	2923	tags=75%, list=17%, signal=89%
HALLMARK_G2M_CHECKPOINT	191	0.6743256	2.724793	0.0	0.0	0.0	2465	tags=53%, list=14%, signal=62%
HALLMARK_MYC_TARGETS_V1	199	0.5443253	2.222949	0.0	0.0	0.0	5482	tags=59%, list=32%, signal=86%
HALLMARK_MTORC1_SIGNALING	198	0.52600056	2.1334949	0.0	0.0	0.0	3476	tags=40%, list=20%, signal=50%
HALLMARK_MITOTIC_SPINDLE	197	0.5062556	2.0471668	0.0	0.0	0.0	1987	tags=31%, list=11%, signal=35%
HALLMARK_MYC_TARGETS_V2	57	0.5541562	1.8464261	0.0	5.226724E-4	0.003	5702	tags=63%, list=33%, signal=94%
HALLMARK_CHOLESTEROL_HOMEOSTASIS	73	0.50320303	1.7783641	0.0	9.1641356E-4	0.006	2070	tags=40%, list=12%, signal=45%
HALLMARK_NOTCH_SIGNALING	32	0.5165779	1.5285118	0.019189766	0.013299267	0.086	3004	tags=34%, list=17%, signal=41%
HALLMARK_HEDGEHOG_SIGNALING	36	0.46456835	1.4196477	0.037861917	0.040052503	0.26	3122	tags=42%, list=18%, signal=51%

Appendix Table S2. Clinical data from patients indicating genotype of vascular malformation, sex of patient, age at surgery, type of vascular malformation and location of vascular malformation. nd, not determined.

ID	Genotype		Sex	Age at surgery	Type	Location
VM01	PIK3CA	E542K	female	10	Venous malformation	hand
VM02	PIK3CA	E542K	female	56	Venous malformation	hand
VM03	PIK3CA	E542K	male	2	Venous malformation	nd
VM04	PIK3CA	E542K	male	5	Venous - Lymphatic malformation	leg
VM05	PIK3CA	E545K	male	15	Venous - Lymphatic malformation	nd
VM06	PIK3CA	H1047R	female	62	Venous malformation	finger
VM07	TEK	L914F	female	38	Venous malformation	lip
VM08	TEK	L914F	male	50	Venous malformation	finger
VM09	TEK	L914F	female	10	Venous malformation	lip
VM10	TEK	L914F	nd	1	Venous malformation	face and neck
VM11	TEK	L914F	female	3	Venous Malformation	face

Appendix Table S3. List of primers used for gene expression analysis.

Gene	Primer forward	Primer reverse
Ang2	CCTCGACTACGACGACTCAGT	TCTGCACCACATTCTGTTGGA
Ccnd1	AGCAGAGAGCTACAGACTCCG	TCTGCTCCTCACAGACCTCC
Ccnd2	AAGGAGGTAAGGGAAGCACT	GTTCCTCACAGACCTCTAGCA
Ccnf	GCGGCGGTGTGATCCAT	GCATGGTTGTCCACCAGGTA
Ccng2	AGGCTACCCCGGAGAATGAT	CGCGTGGTACAAGTGCAAAA
Cdc25b	CAGAGCAAGCGCAGGAAAAG	CCTGATGAAGCGGCACATTC
Cdk5rap2	AGAGGCGAAGTCAAGACAGC	GCACGGATGAAGAACCCTTTG
Cdkn1c	CGGCCAATGCGAACGACTTC	TACGCAACCATCTCCGGTTC

Cenpf	GAGCCAGGTTCTGTGAGGAG	TTCCGTCTTCAACCTTCTGC
Mki67	ACCATCATTGACCGCTCCTT	TTGACCTTCCCCATCAGGGT
Pdgfb	CATCCGCTCCTTTGATGATCTT	GTGCTCGGGTCATGTTCAAGT
Rad9a	AAGGCTGTCCATTCGCTATCC	CCTCTGCCAGAACCCGTG
Rbl1	CACAAGAGTTTCGTGAGCGG	ATACGGAAATTCCCCTTGGTG
Rpl32	ACCCCAGAGGCATTGACAAC	ATTGTGGACCAGGAACTTGC
Top2a	GAAGCTCCATGTCGGTAGTTG	CCACAGGGAAGTCCTGTTCC

Appendix Figure Legends

Appendix Figure S1. Gene expression changes upon Pik3ca^{H1047R} expression in ECs.

(**A**) Gene expression analysis by quantitative PCR of Ang2 and Pdgfb in lung mouse ECs after 6h, 24h, 48h and 72h of Pik3ca^{H1047R} expression. Error bars are s.e.m. n = 3 biological replicates per genotype.

(**B**) GSEA enrichment plots of the two most significantly enriched hallmarks upon Pik3ca^{H1047R} expression.

Data information: Statistical analysis was performed by nonparametric Mann–Whitney test. ns (not statistically significant) $p > 0.05$; *$p < 0.05$ and ***$p < 0.001$ were considered statistically significant.

Appendix Figure S2. Molecular rescue of Pik3ca^{H1047R} ECs upon miransertib treatment.

(**A**) Immunoblot showing PI3K/AKT/mTOR pathway by assessing the levels of p-AKT, pPras40, and p-S6 of Pik3ca^{WT} and Pik3ca^{H1047R} mouse ECs after 2h of miransertib treatment.

(**B**) Gene expression analysis by quantitative PCR of Ang2 in lung mouse ECs 24h after treatment with vehicle or 2µM of miransertib. To induce Pik3ca^{H1047R} expression, ECs were pretreated with 4-OHT for 24h prior to miransertib treatment. Error bars are s.e.m. n = 3 biological replicates per genotype.

(**C**) Heatmap of representative DEGs from the DNA replication and cell cycle processes identified in the RNAseq (Fig 1B, C).

(**D**) Gene expression analysis by quantitative PCR of DNA replication and cell cycle regulators in lung mouse ECs 24 h after treatment with vehicle or 2µM of miransertib. To induce $Pik3ca^{H1047R}$ expression, ECs were pretreated with 4-OHT for 24h prior to miransertib treatment. Error bars are s.e.m. n = 3 biological replicates per genotype.

Data information: Statistical analysis was performed by one-tail t test (hypothesis-driven). ns (not statistically significant) $p > 0.05$, *$p < 0.05$ and **$p < 0.01$ were considered statistically significant.

Appendix Figure S3. Characterization of primary ECs isolated from patient-derived vascular malformations.

(**A**) Digital Droplet PCR-based detection of the $PIK3CA^{WT}$ (green) and $PIK3CA^{E542K}$ (blue) alleles in cultured ECs (CD31-positive) from the vascular malformation of patient VM01.

(**B**) Sequencing chromatograms for *PIK3CA* and *TEK* mutant VM-derived ECs. Arrows show the detected point mutations.

(**C**) Representative confocal images of *PIK3CA* and *TEK* patient-derived ECs immunostained for VE-cadherin (EC-specific junctional protein) and ERG (EC-specific transcription factor). Cell nuclei were visualised with DAPI. Scale bars: 30 µm.

(**D**) Immunoblot showing the impact of miransertib at increasing doses on PI3K/AKT/mTORC1 pathway (by assessing p-AKT and p-S6 levels) in wild type ECs (HUVECs).

(**E**) Wild type (HUVECs), *PIK3CA* and *TEK* mutant EC viability upon the treatment with the MK-2206 and miransertib inhibitors for 72h at different doses assessed by MTS assay. Miransertib vs. MK-2206: *PIK3CA*-mutant: $p < 0.0001$; *TEK*-mutant: $p = 0.0017$; wild type (HUVECs): $p = 0.0654$. Statistical analysis was performed by comparison of best-fit values using the extra sum-of-squares F test.

Appendix Figure S1

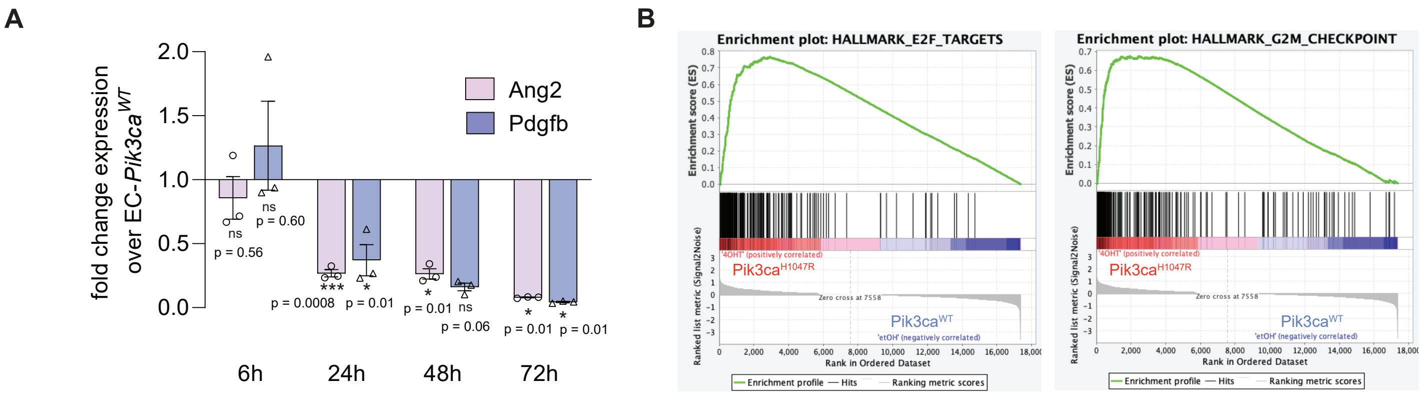

Appendix Figure S2

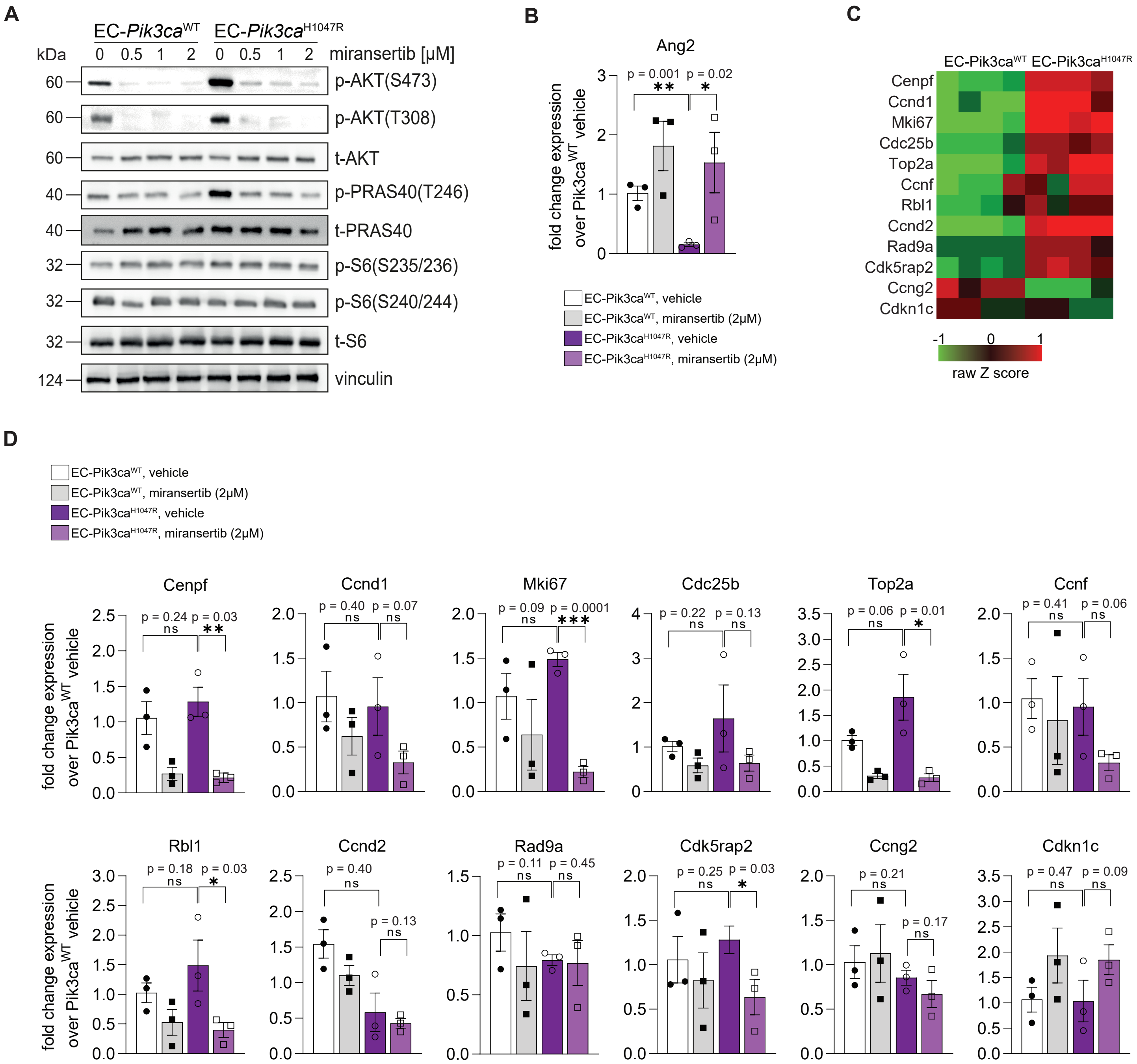

Appendix Figure S3

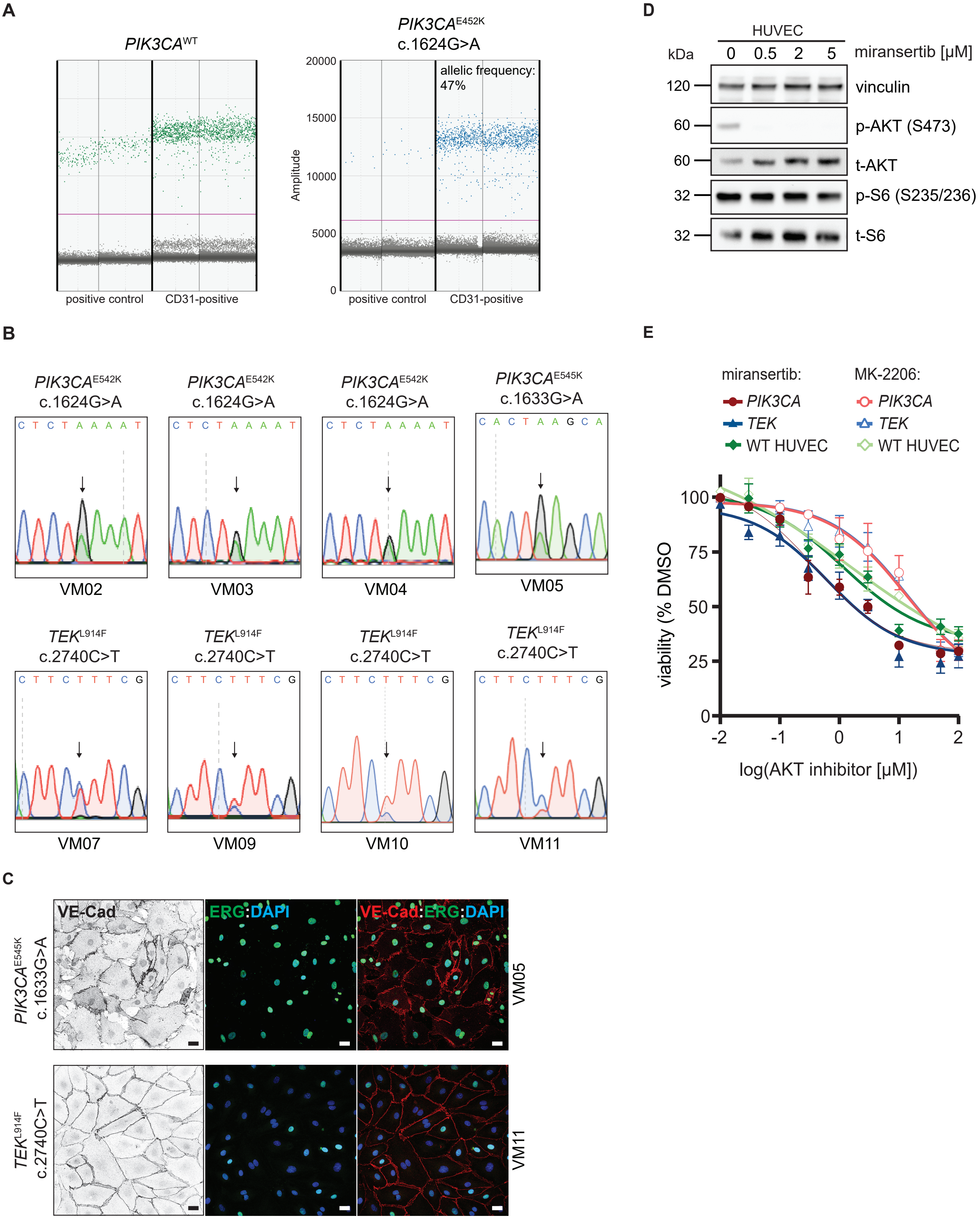

Supplement: Supplementary file 1 — Appendix [file EMMM-14-e15619-s005.pdf]
