# Supplementary material for: The onset of PI3K‐related vascular malformations occurs during angiogenesis and is prevented by the AKT inhibitor miransertib
Source: EMBO Mol Med. 2022 Jun 13;14(7):e15619. doi: 10.15252/emmm.202115619 (PMC9260211; doi:10.15252/emmm.202115619)
Supplement: Supplementary file 2 — Expanded View Figures PDF [file EMMM-14-e15619-s006.pdf]

Expanded View Figures

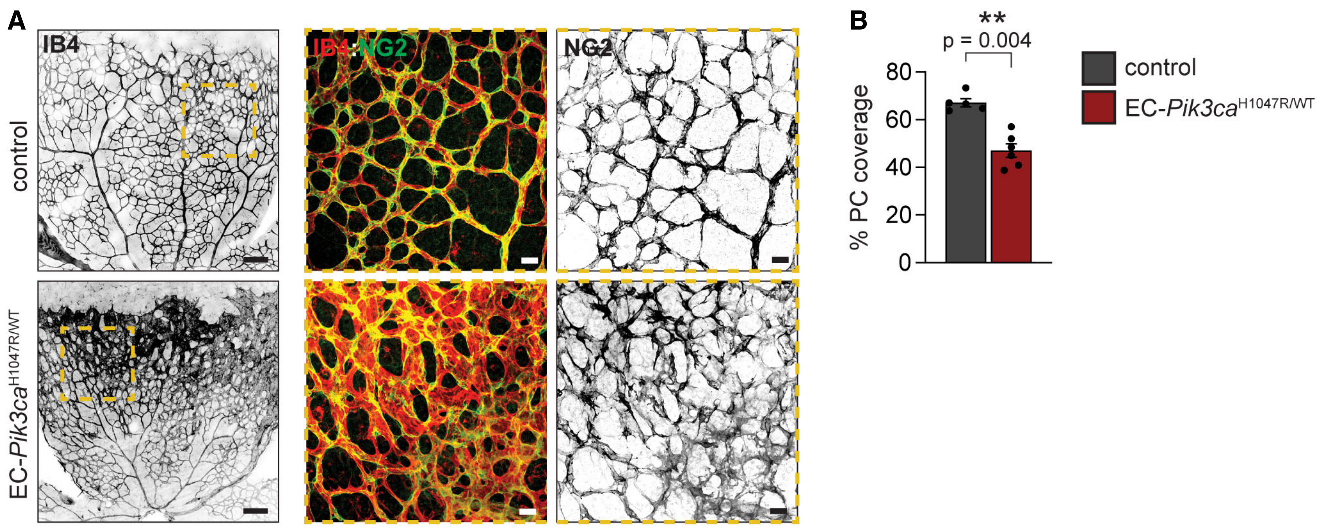

Figure EV1. *Pik3ca*-driven vascular malformations exhibit reduced coverage by pericytes.

A Representative images of control and EC-*Pik3ca*$^{H1047R/WT}$ P6 retinas immunostained for blood vessels (IB4) and pericyte marker (NG2). Dashed areas with high magnifications shown on the right. Scale bars: 150 µm (left panels) and 30 µm (right panels).

B Quantification of vessel coverage by pericytes. Data presented as a percentage of pericyte coverage over EC area (IB4 staining). Error bars are SEM. $n \geq 5$ retinas per genotype. Statistical analysis was performed by nonparametric Mann–Whitney test. $^{**}P < 0.01$ was considered statistically significant.

Source data are available online for this figure.

Figure EV2. EC-*Pik3ca*$^{H1047R/WT}$ P4 retinas exhibit vascular malformations.

A Scheme showing 4-OHT treatment regime.

B Representative images of P4 retinas from control and EC-*Pik3ca*$^{H1047R/WT}$ immunostained for blood vessels (IB4). Scale bars: 150 µm.

C Representative high magnification images showing blood vessels (IB4), EC nuclei (Erg) and proliferative cells (EdU).

D Representative high magnification images of retinas immunostained for blood vessels (IB4) and pS6 (S235/236).

E Representative high magnification images showing blood vessels (IB4) and pericytes (NG2).

F–K Quantification of (F) retina vascularity, (G) EC number, (H) EC proliferation by EdU staining, (I) mitotic index (EdU+ EC/total EC), (J) pS6 intensity and (K) pericyte coverage in control and EC-*Pik3ca*$^{H1047R/WT}$ P4 retinas. Error bars are SEM. $n \geq 5$ retinas per genotype. Statistical analysis was performed by nonparametric Mann–Whitney test. $^{*}P < 0.05$ and $^{**}P < 0.01$ were considered statistically significant.

Data information: Scale bars (C, D and E) = 30 µm.
Source data are available online for this figure.

© 2022 The Authors

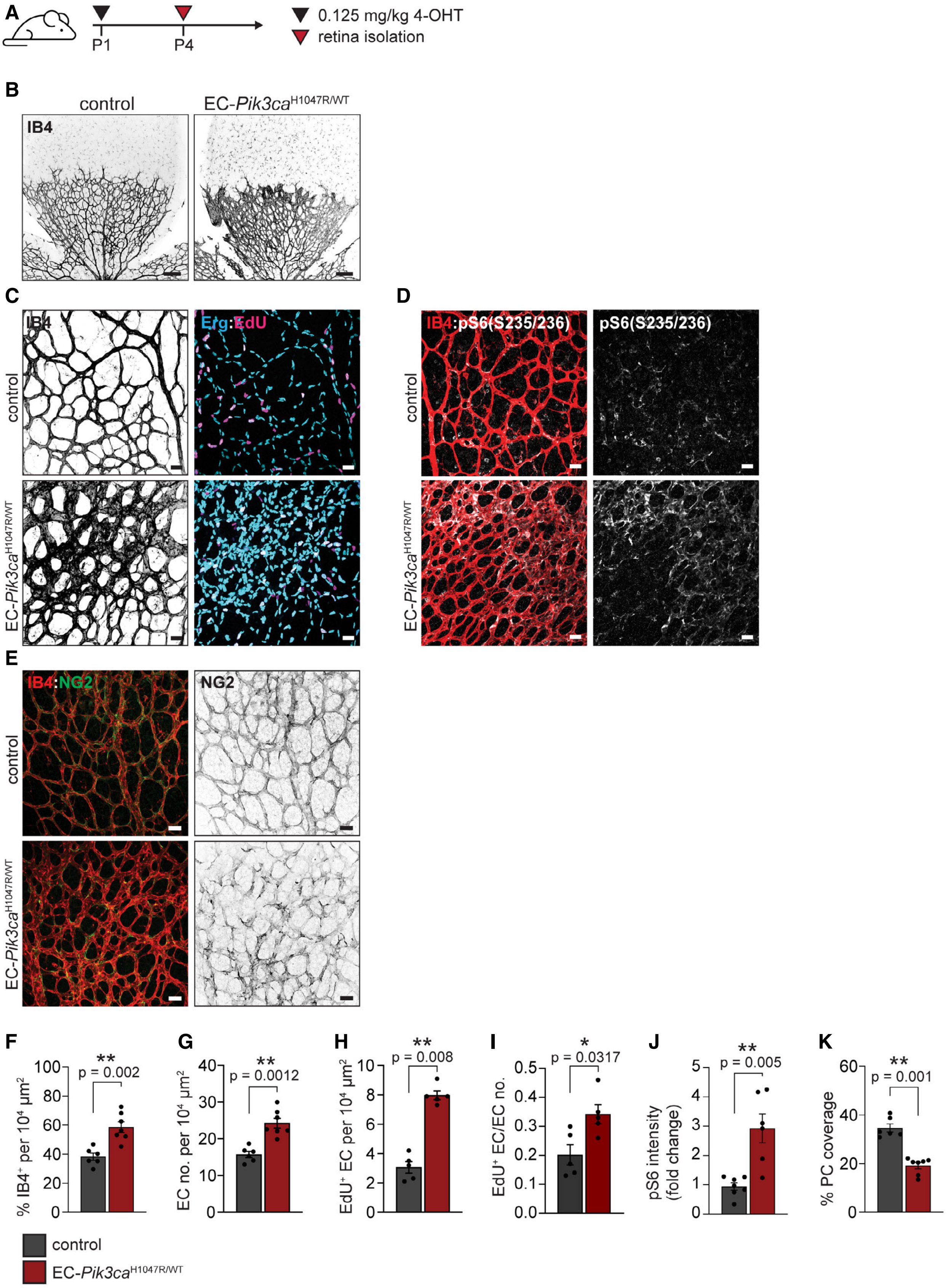
A
0.125 mg/kg 4-OHT
retina isolation
P1
P4
B
control
EC-Pik3caH1047R/WT
IB4
C
IB4
Erg:EdU
control
EC-Pik3caH1047R/WT
D
IB4:pS6(S235/236)
pS6(S235/236)
control
EC-Pik3caH1047R/WT
E
IB4:NG2
NG2
control
EC-Pik3caH1047R/WT
F
% IB4+ per 10⁴ µm²
** p = 0.002
G
EC no. per 10⁴ µm²
** p = 0.0012
H
EdU+ EC per 10⁴ µm²
** p = 0.008
I
EdU+ EC/EC no.
* p = 0.0317
J
pS6 intensity (fold change)
** p = 0.005
K
% PC coverage
** p = 0.001
control
EC-Pik3caH1047R/WT

Figure EV2.

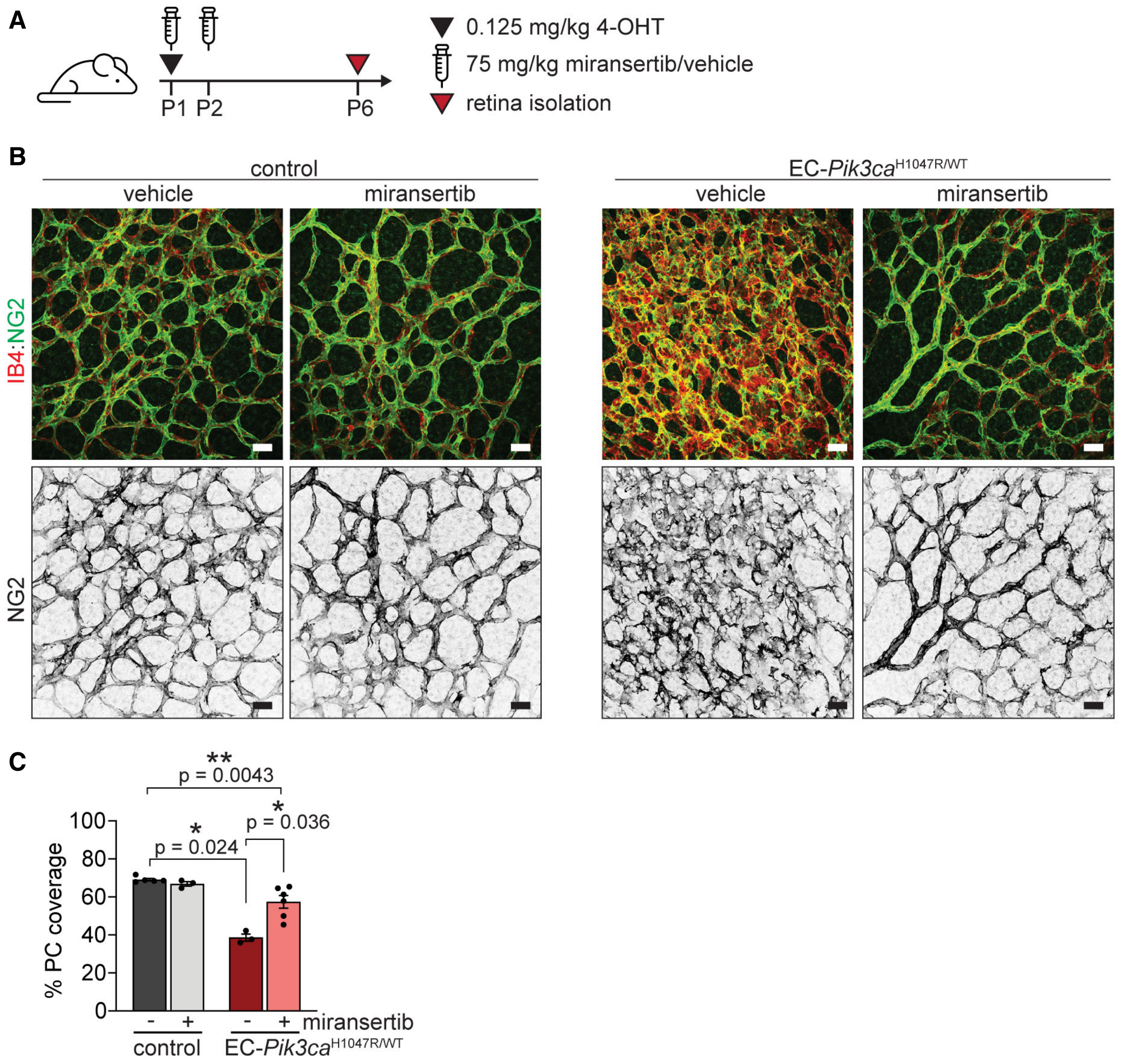

Figure EV3. Miransertib prevents from loss of pericyte coverage.

A 4-OHT and miransertib dosing scheme used for a prevention therapeutic experimental setup.

B Representative images of P6 retinas isolated from control and EC-*Pik3ca*$^{H1047R/WT}$ mouse littermates. Blood vessels were stained with IB4 and NG2 immunostaining was used to visualize pericytes. Scale bars: 30 μm.

C Quantification of vessel coverage by pericytes. Data presented as a percentage. Error bars are SEM. $n \geq 3$ retinas per genotype. Statistical analysis was performed by nonparametric Mann–Whitney test. $^*P < 0.05$ and $^{**}P < 0.01$ was considered statistically significant.

Source data are available online for this figure.

 © 2022 The Authors
