# Supplementary figures and images for: The onset of PI3K‐related vascular malformations occurs during angiogenesis and is prevented by the AKT inhibitor miransertib
Source: EMBO Mol Med. 2022 Jun 13;14(7):e15619. doi: 10.15252/emmm.202115619 (PMC9260211; doi:10.15252/emmm.202115619)

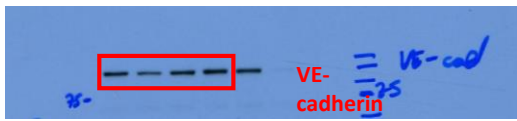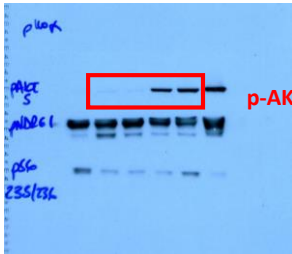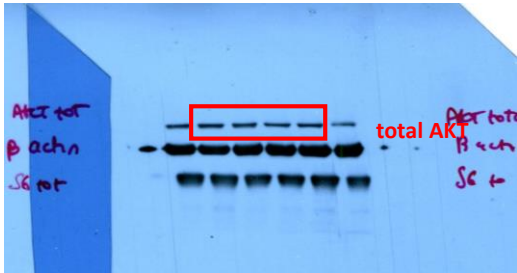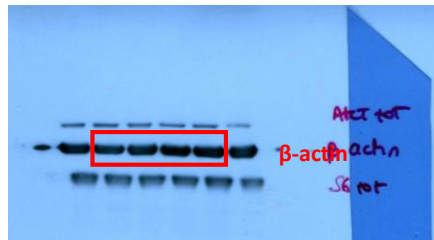

Supplement: Supplementary file 4 — Source Data for Figure 1 [file EMMM-14-e15619-s001.zip › Fig 1/WB Fig 1A.pdf]

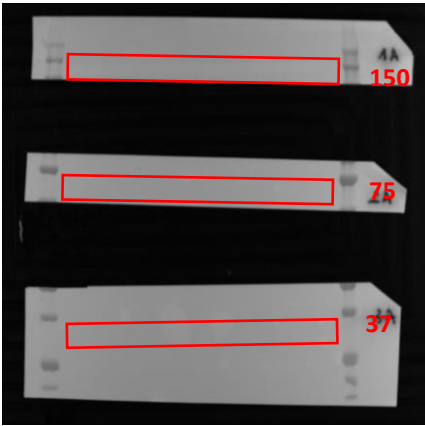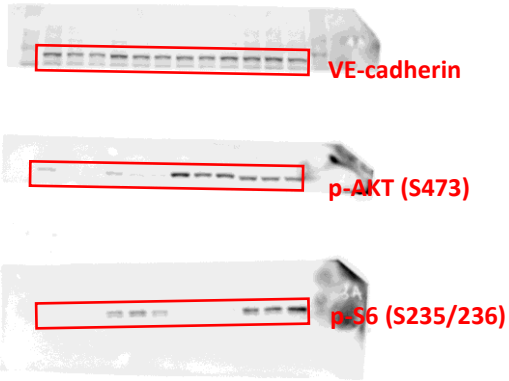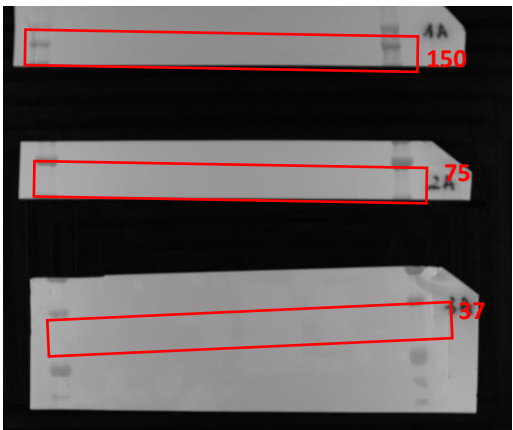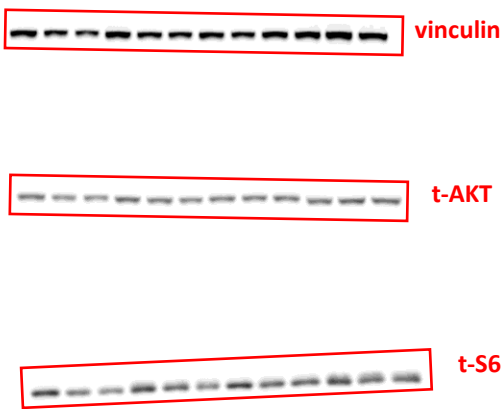

Supplement: Supplementary file 4 — Source Data for Figure 1 [file EMMM-14-e15619-s001.zip › Fig 1/WB Fig 1D.pdf]

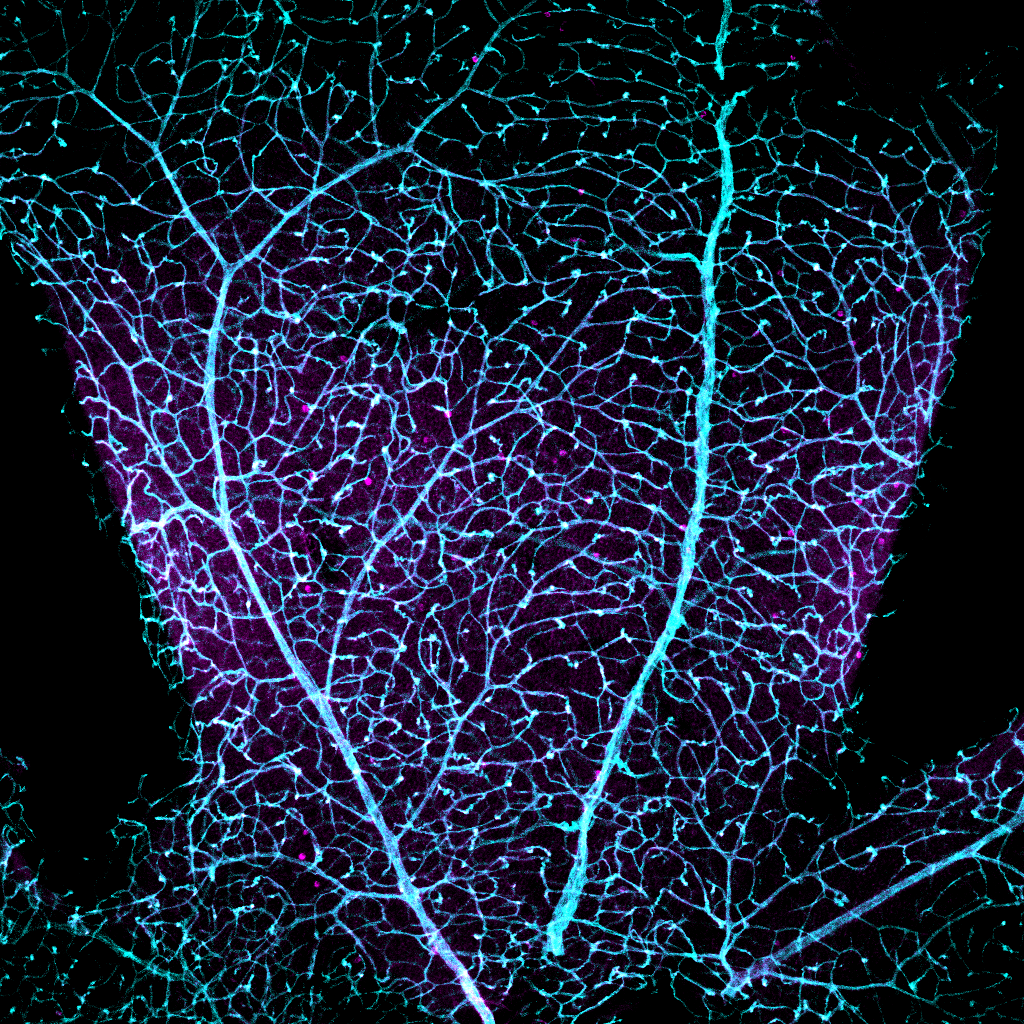

Supplement: Supplementary file 5 — Source Data for Figure 2 [file EMMM-14-e15619-s011.zip › EMM-2021-15619-V3-Figure_2_Source_Data/Fig. 2/2E/EC-mtmG IB4Blue GFPGreen P15 NEW.tif]

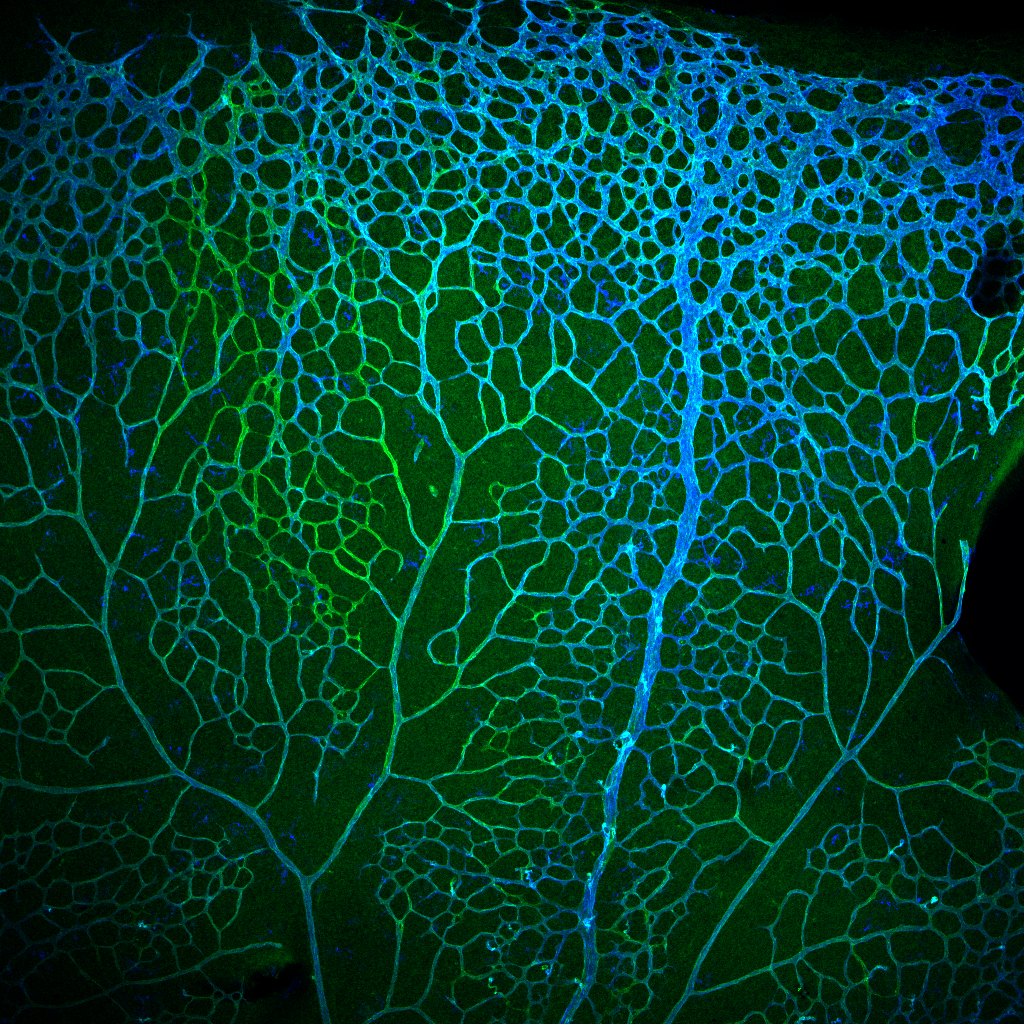

Supplement: Supplementary file 5 — Source Data for Figure 2 [file EMMM-14-e15619-s011.zip › EMM-2021-15619-V3-Figure_2_Source_Data/Fig. 2/2E/EC-mTmG IB4Blue GFPGreen P6.tif]

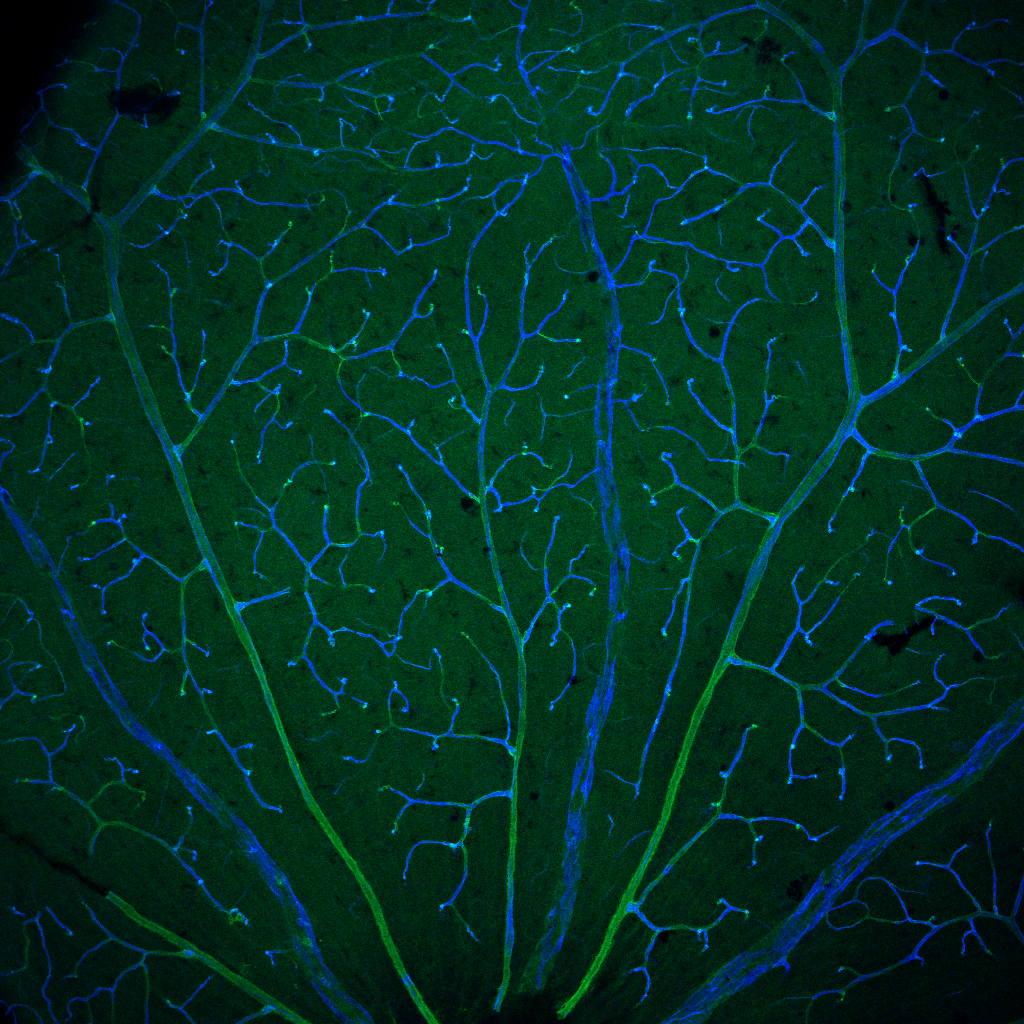

Supplement: Supplementary file 5 — Source Data for Figure 2 [file EMMM-14-e15619-s011.zip › EMM-2021-15619-V3-Figure_2_Source_Data/Fig. 2/2E/EC-mTmG IB4Blue GFPGreen P21.tif]

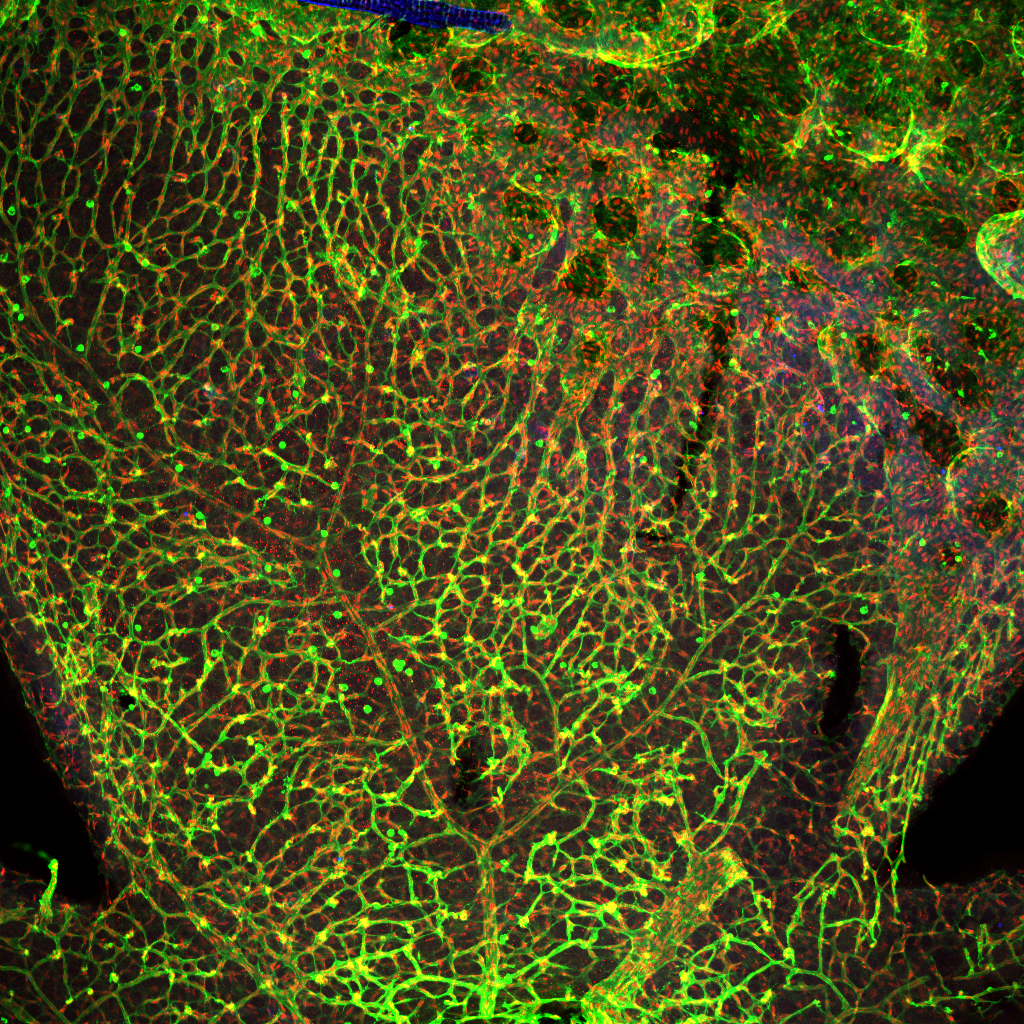

Supplement: Supplementary file 5 — Source Data for Figure 2 [file EMMM-14-e15619-s011.zip › EMM-2021-15619-V3-Figure_2_Source_Data/Fig. 2/2B/Pik3caH1047R P15.tif]

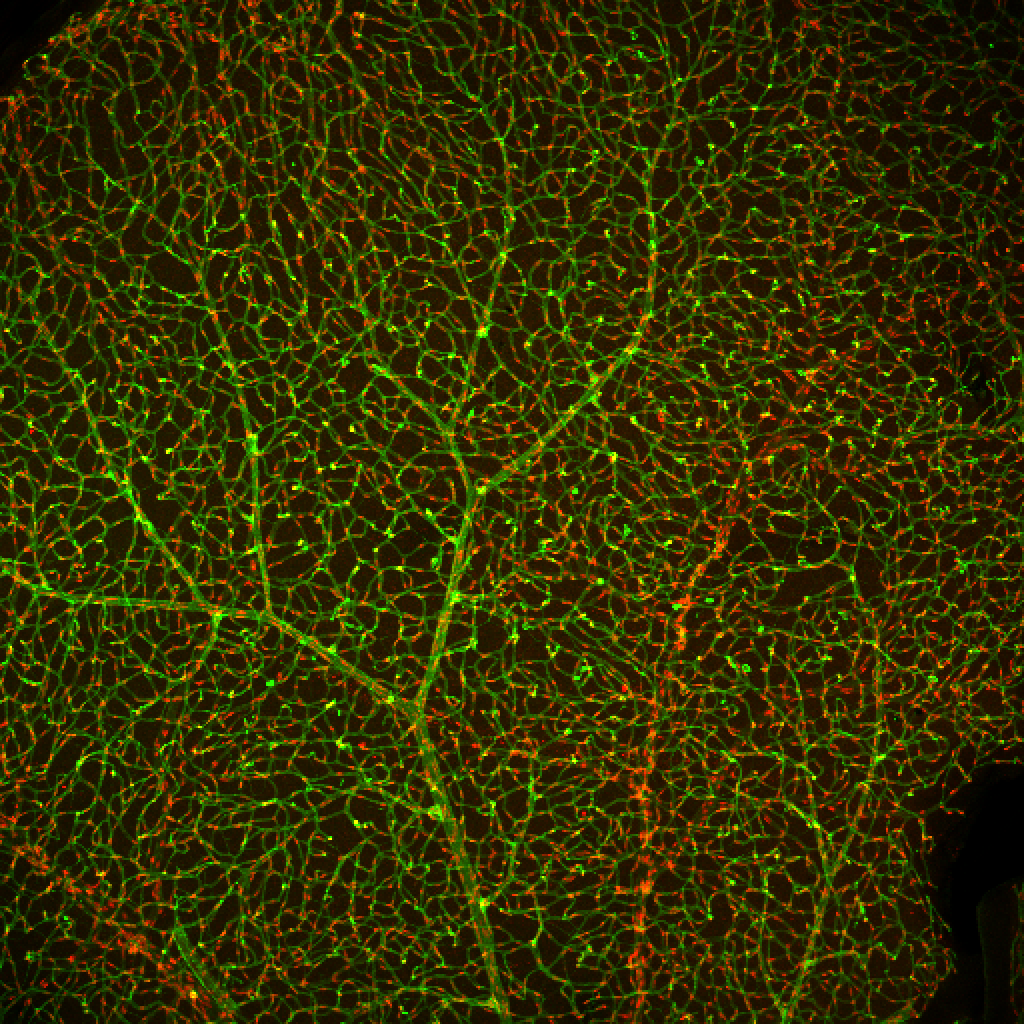

Supplement: Supplementary file 5 — Source Data for Figure 2 [file EMMM-14-e15619-s011.zip › EMM-2021-15619-V3-Figure_2_Source_Data/Fig. 2/2B/control P21.tif]

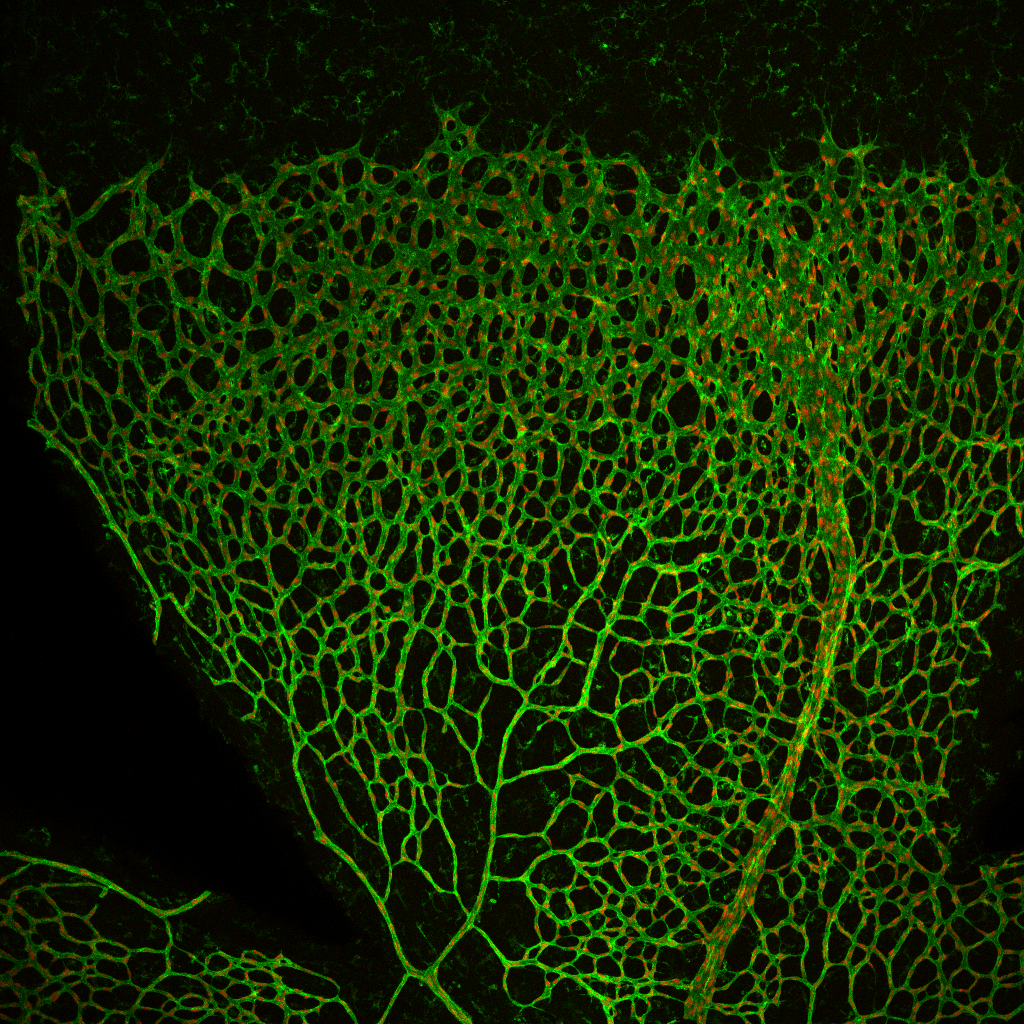

Supplement: Supplementary file 5 — Source Data for Figure 2 [file EMMM-14-e15619-s011.zip › EMM-2021-15619-V3-Figure_2_Source_Data/Fig. 2/2B/control P6.tif]

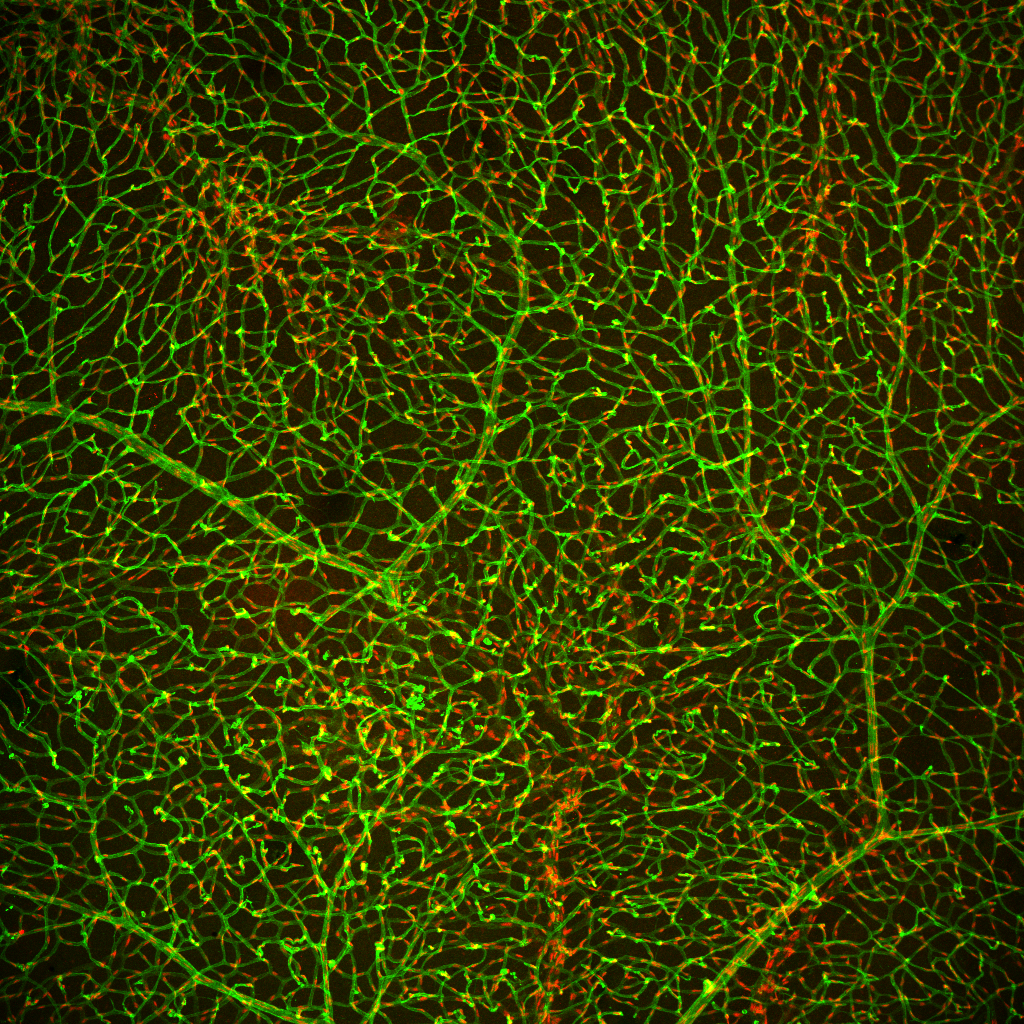

Supplement: Supplementary file 5 — Source Data for Figure 2 [file EMMM-14-e15619-s011.zip › EMM-2021-15619-V3-Figure_2_Source_Data/Fig. 2/2B/Pik3caH1047R P21.tif]

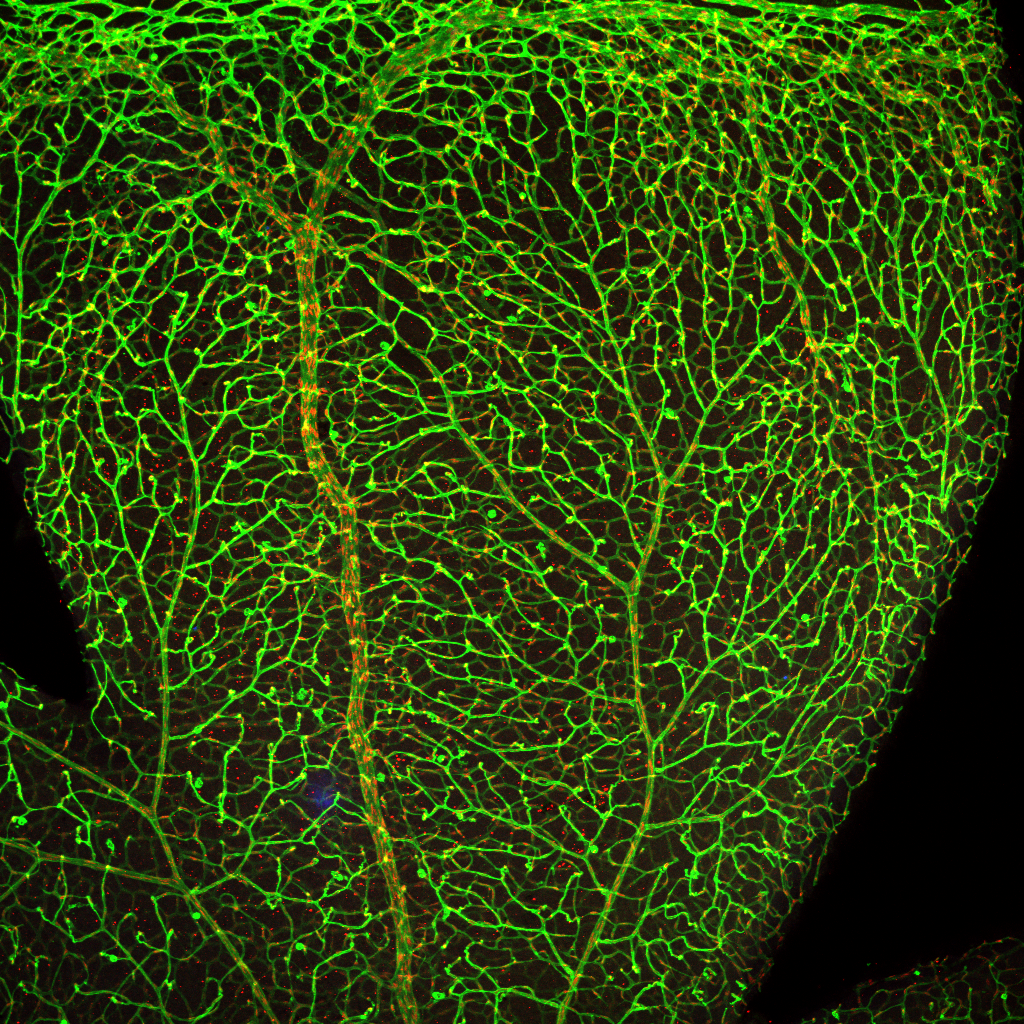

Supplement: Supplementary file 5 — Source Data for Figure 2 [file EMMM-14-e15619-s011.zip › EMM-2021-15619-V3-Figure_2_Source_Data/Fig. 2/2B/control P15.tif]

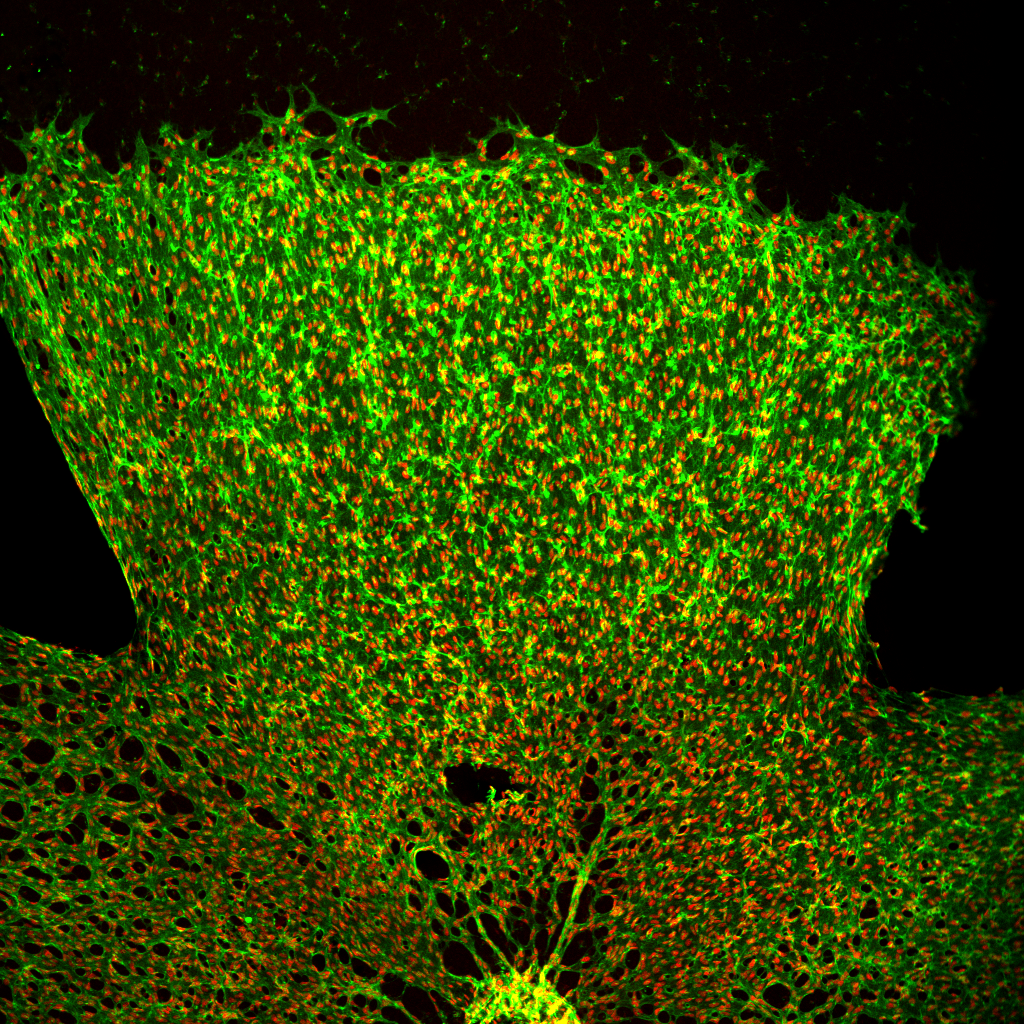

Supplement: Supplementary file 5 — Source Data for Figure 2 [file EMMM-14-e15619-s011.zip › EMM-2021-15619-V3-Figure_2_Source_Data/Fig. 2/2B/Pik3caH1047R P6.tif]

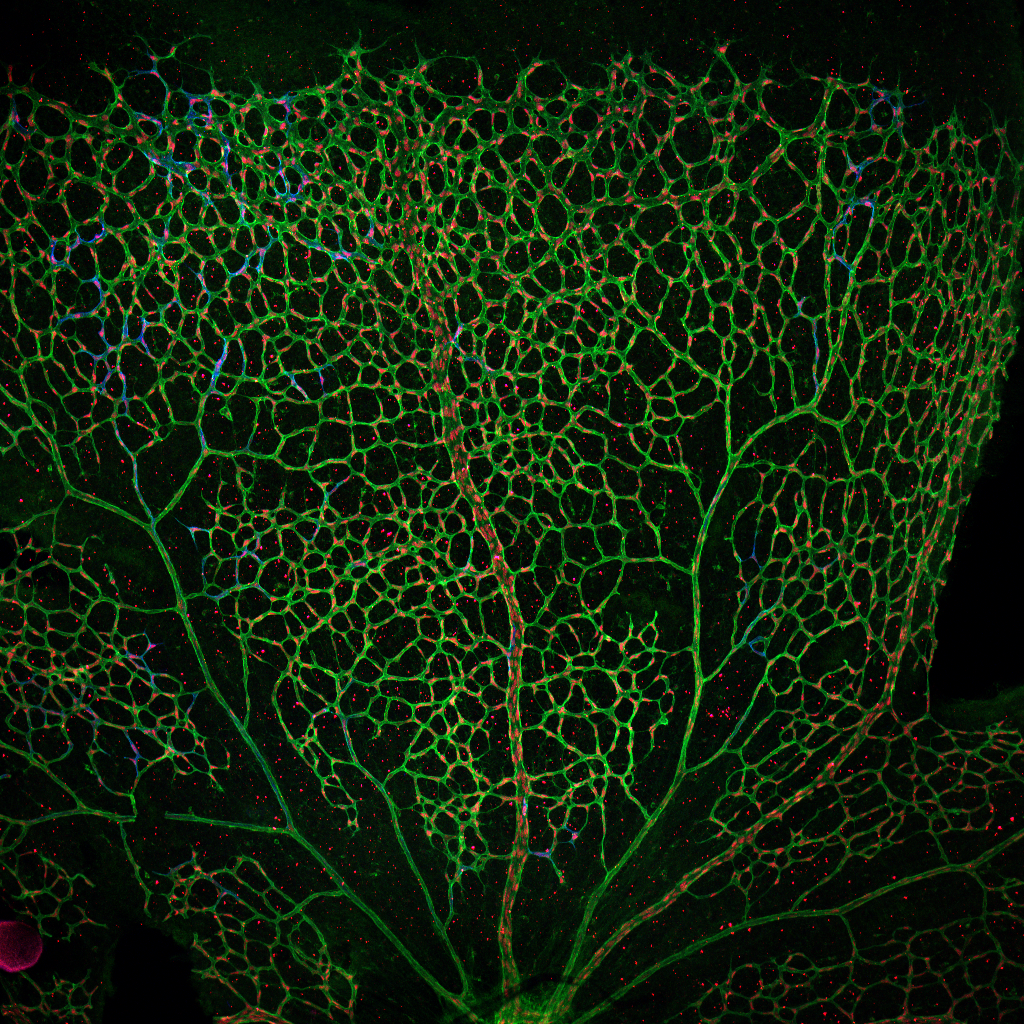

Supplement: Supplementary file 6 — Source Data for Figure 3 [file EMMM-14-e15619-s012.zip › EMM-2021-15619-V3-Figure_3_Source_Data/Fig. 3/3B/EC-mTmG 0.025 mgkg.tif]

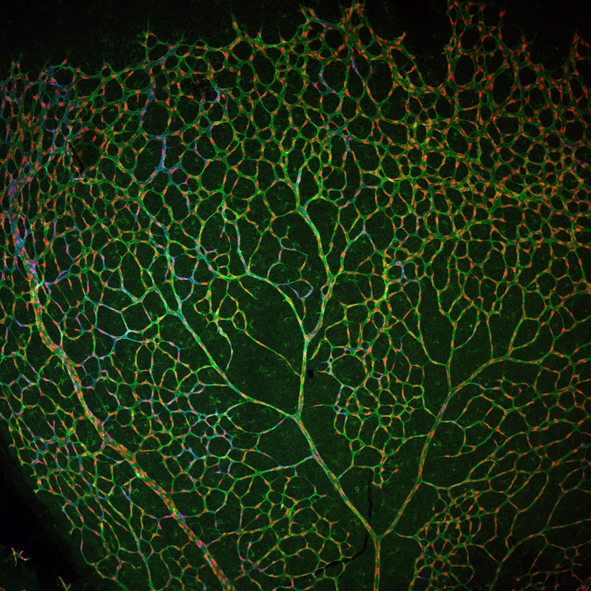

Supplement: Supplementary file 6 — Source Data for Figure 3 [file EMMM-14-e15619-s012.zip › EMM-2021-15619-V3-Figure_3_Source_Data/Fig. 3/3B/EC-mTmG 0.25 mgkg.tif]

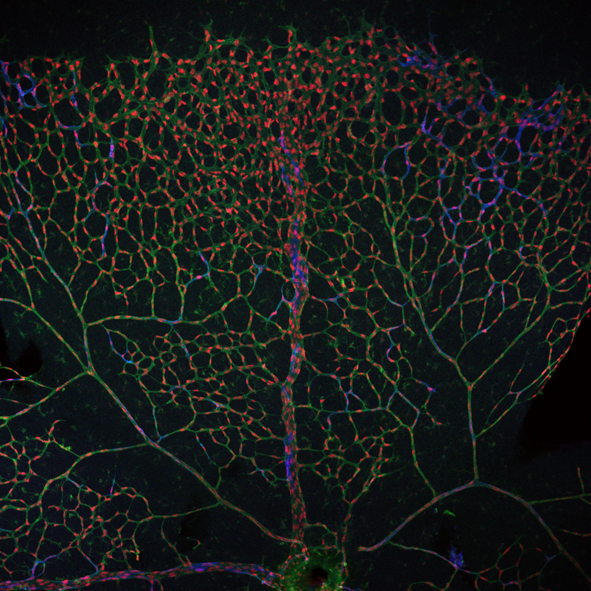

Supplement: Supplementary file 6 — Source Data for Figure 3 [file EMMM-14-e15619-s012.zip › EMM-2021-15619-V3-Figure_3_Source_Data/Fig. 3/3B/EC-mTmG 0.125 mgkg.tif]

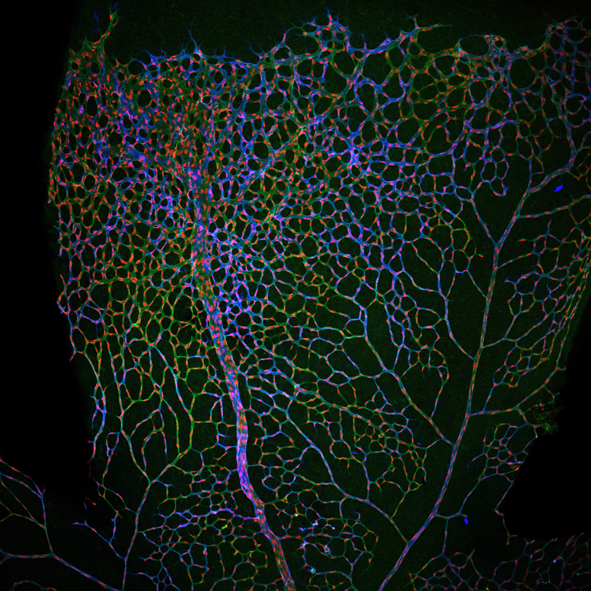

Supplement: Supplementary file 6 — Source Data for Figure 3 [file EMMM-14-e15619-s012.zip › EMM-2021-15619-V3-Figure_3_Source_Data/Fig. 3/3B/EC-mTmG 2.5 mgkg.tif]

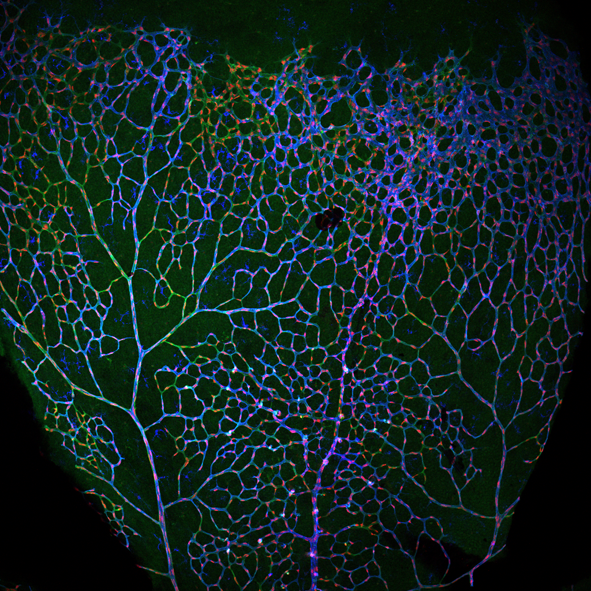

Supplement: Supplementary file 6 — Source Data for Figure 3 [file EMMM-14-e15619-s012.zip › EMM-2021-15619-V3-Figure_3_Source_Data/Fig. 3/3B/EC-mTmG 25 mgkg.tif]

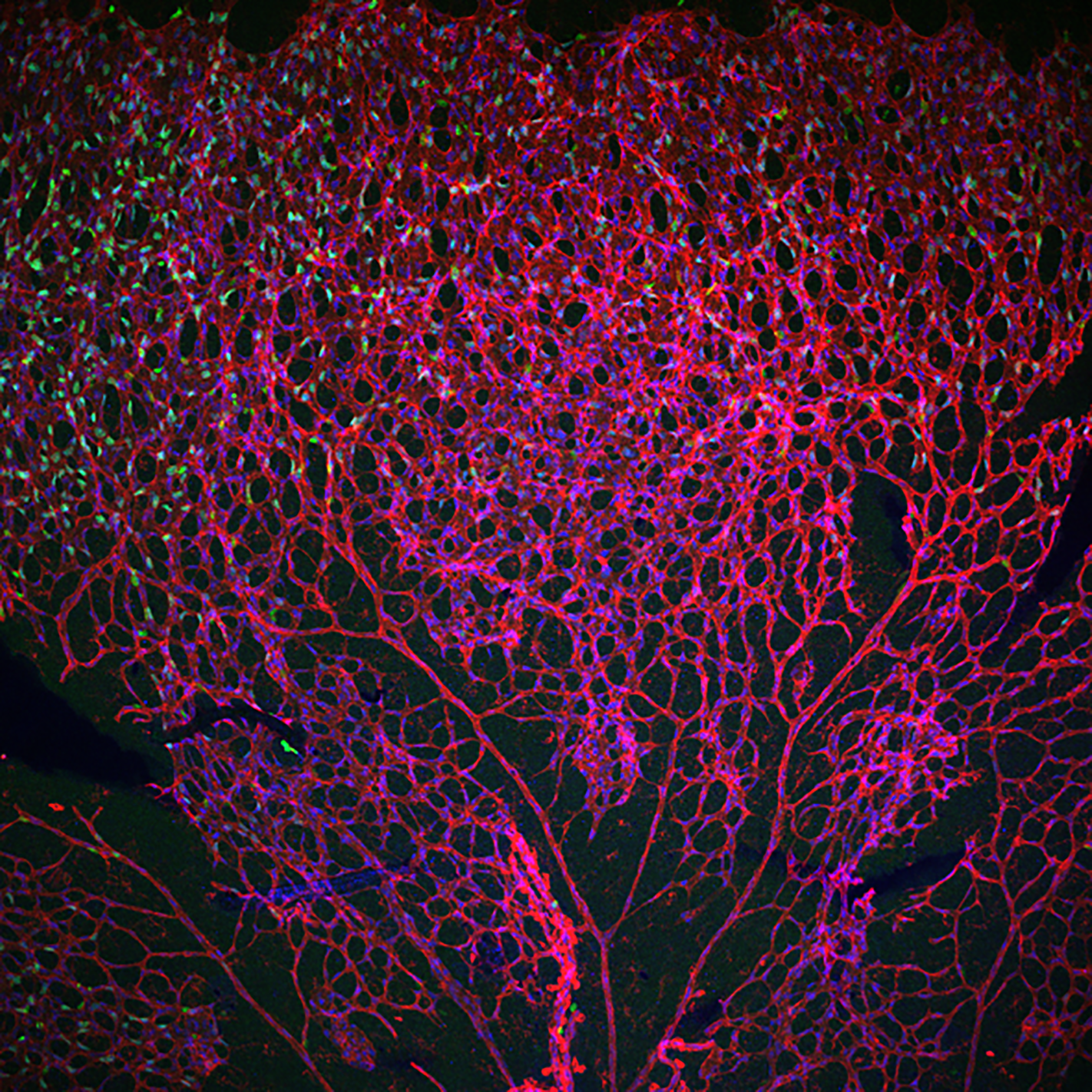

Supplement: Supplementary file 6 — Source Data for Figure 3 [file EMMM-14-e15619-s012.zip › EMM-2021-15619-V3-Figure_3_Source_Data/Fig. 3/3A/EC-Pik3caH1047R 10X 0.125 mgkg.tif]

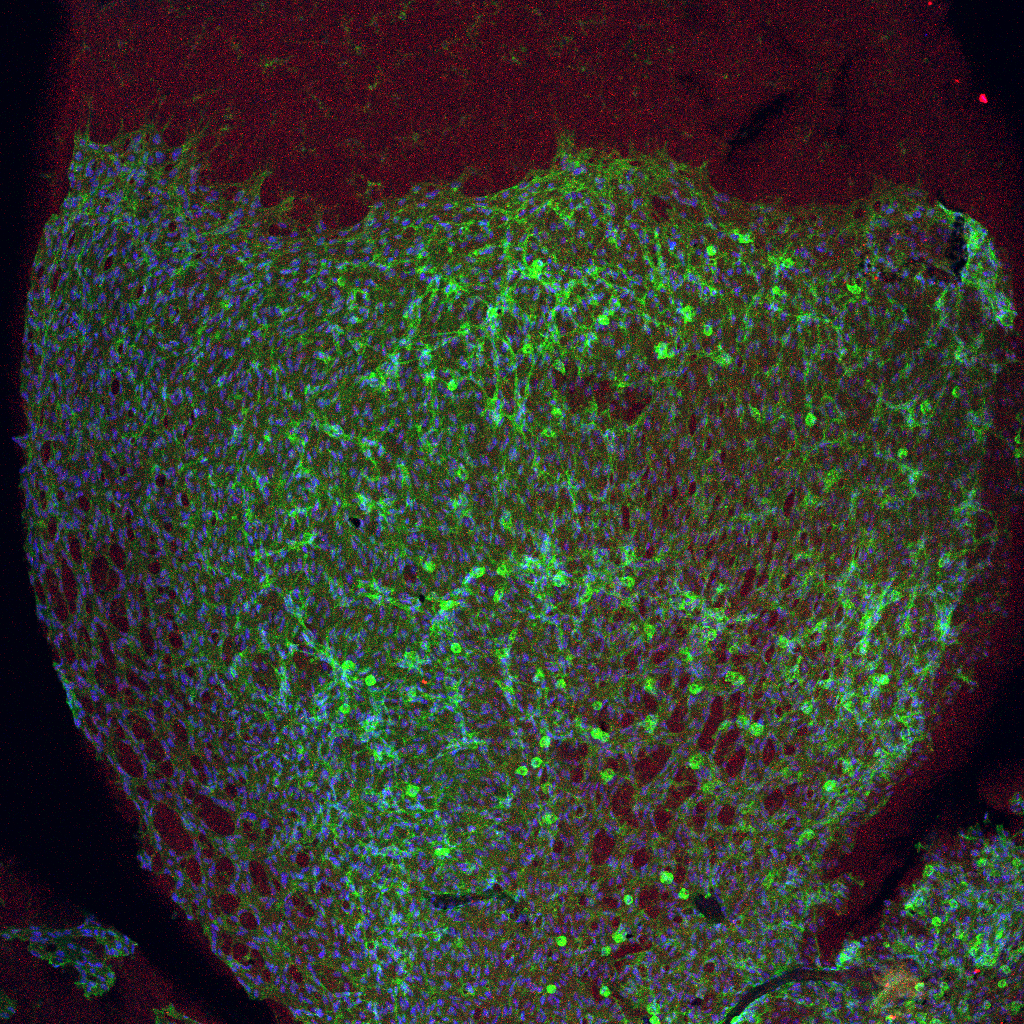

Supplement: Supplementary file 6 — Source Data for Figure 3 [file EMMM-14-e15619-s012.zip › EMM-2021-15619-V3-Figure_3_Source_Data/Fig. 3/3A/EC-Pik3caH1047R 10X 25 mgkg.tif]

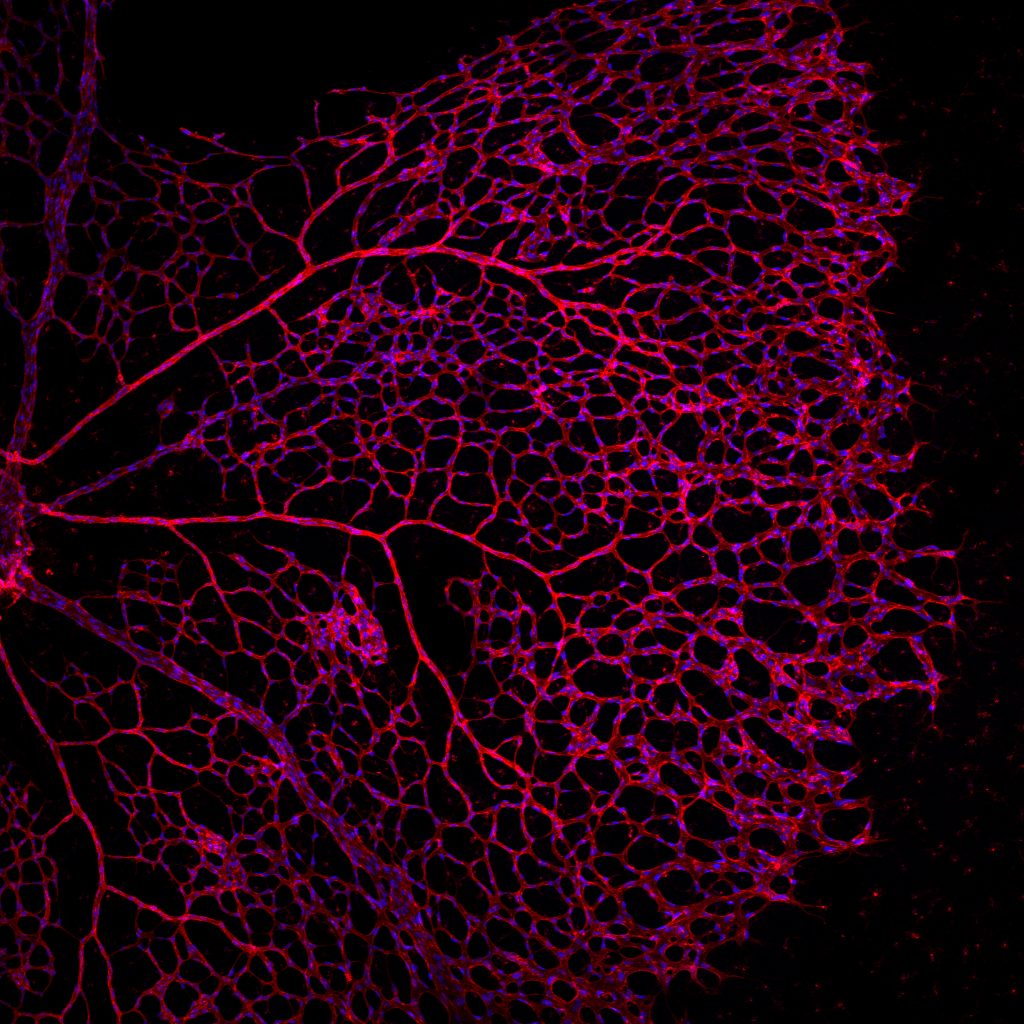

Supplement: Supplementary file 6 — Source Data for Figure 3 [file EMMM-14-e15619-s012.zip › EMM-2021-15619-V3-Figure_3_Source_Data/Fig. 3/3A/EC-Pik3caH1047R 10X 0.025 mgkg.tif]

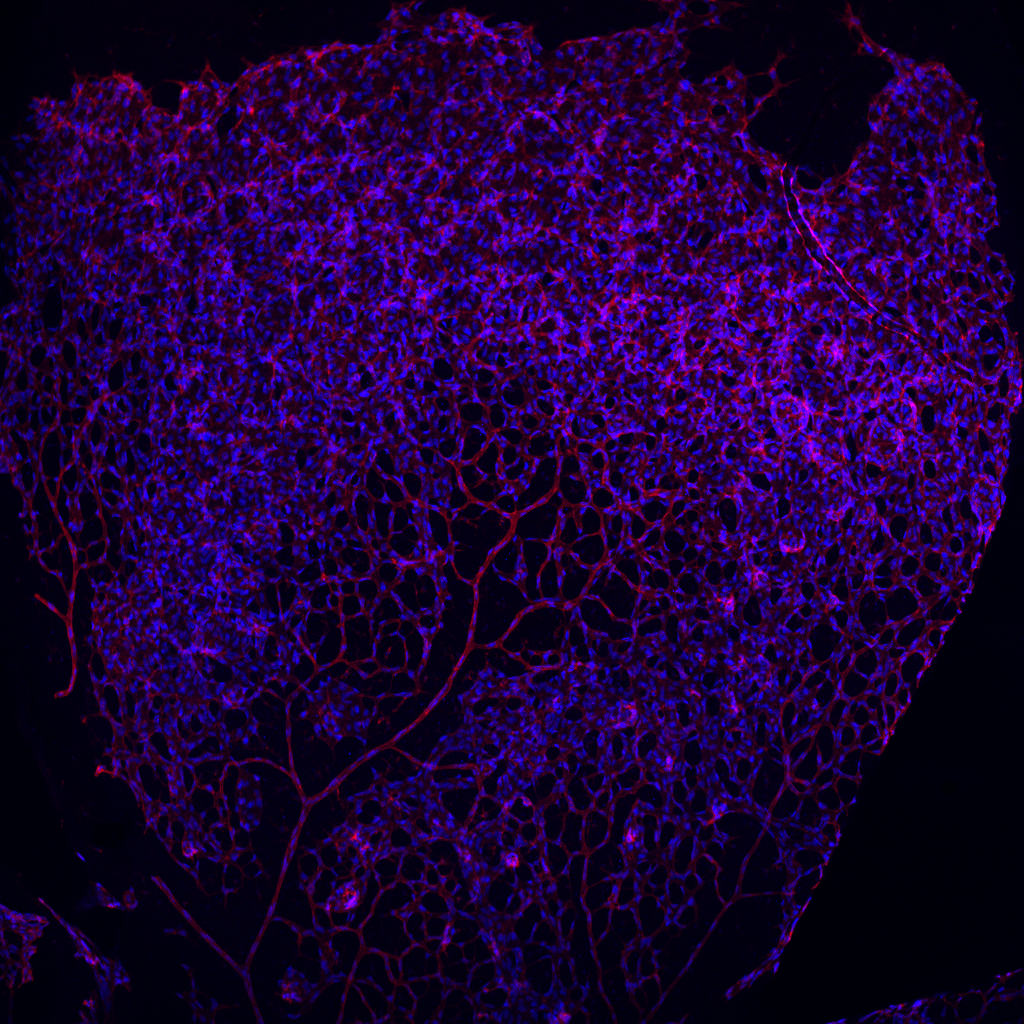

Supplement: Supplementary file 6 — Source Data for Figure 3 [file EMMM-14-e15619-s012.zip › EMM-2021-15619-V3-Figure_3_Source_Data/Fig. 3/3A/EC-Pik3caH1047R 10X 0.25 mgkg.tif]

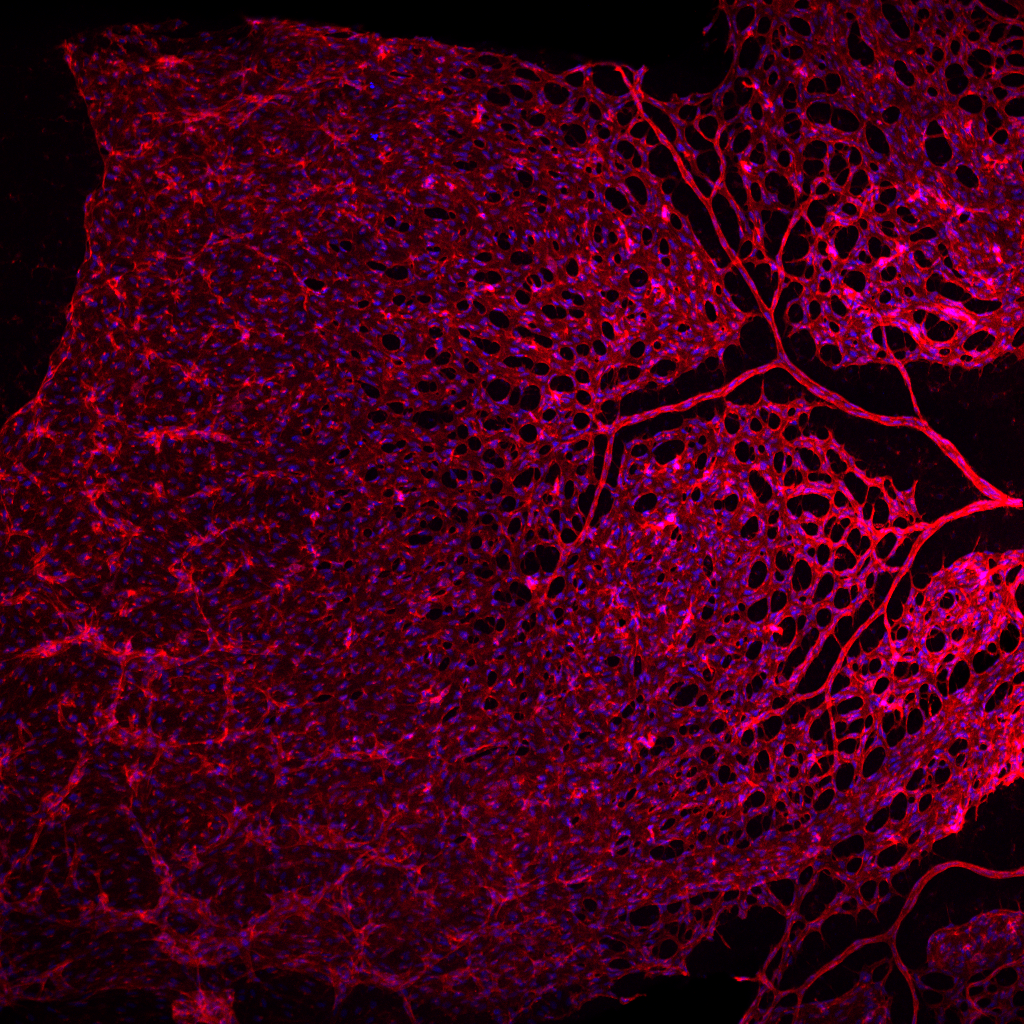

Supplement: Supplementary file 6 — Source Data for Figure 3 [file EMMM-14-e15619-s012.zip › EMM-2021-15619-V3-Figure_3_Source_Data/Fig. 3/3A/EC-Pik3caH1047R 10X 2.5 mgkg.tif]

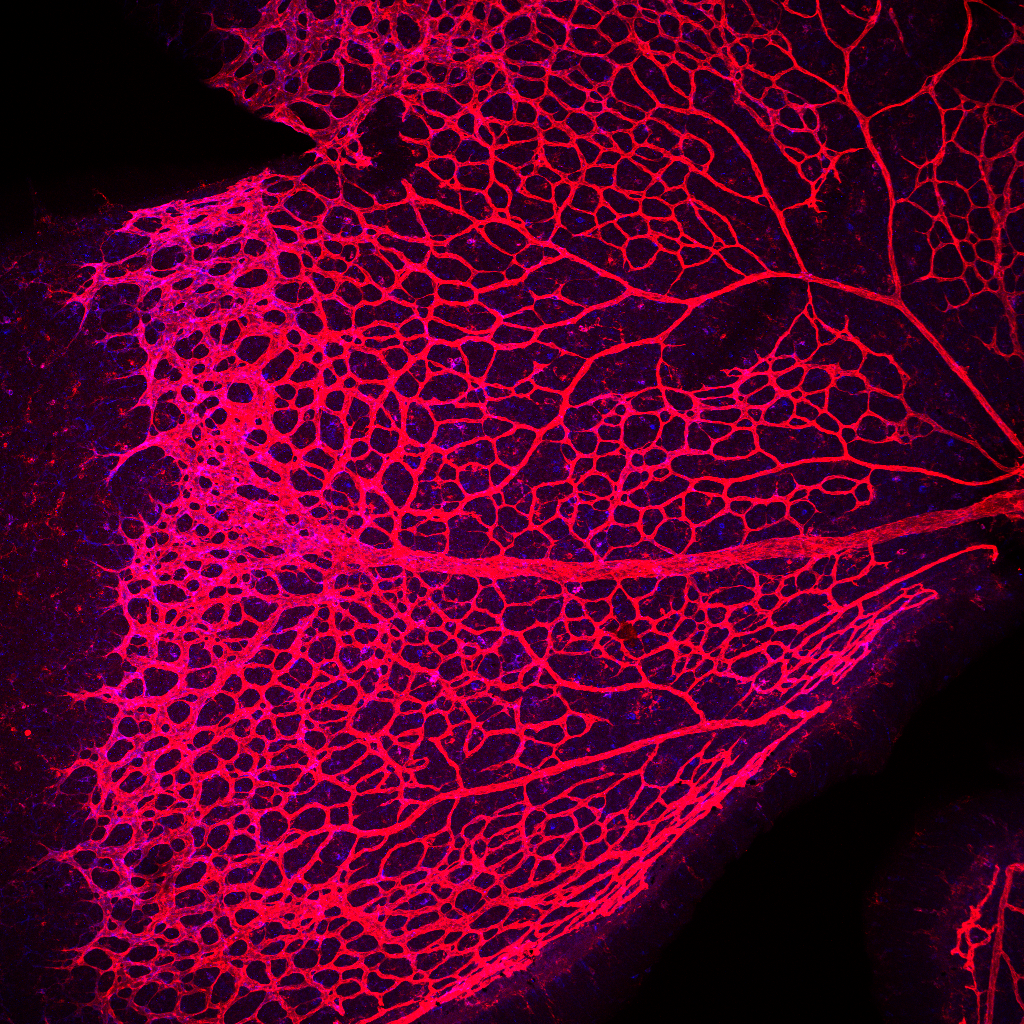

Supplement: Supplementary file 7 — Source Data for Figure 4 [file EMMM-14-e15619-s010.zip › EMM-2021-15619-V3-Figure_4_Source_Data/Fig. 4/4B/Control 10X.tif]

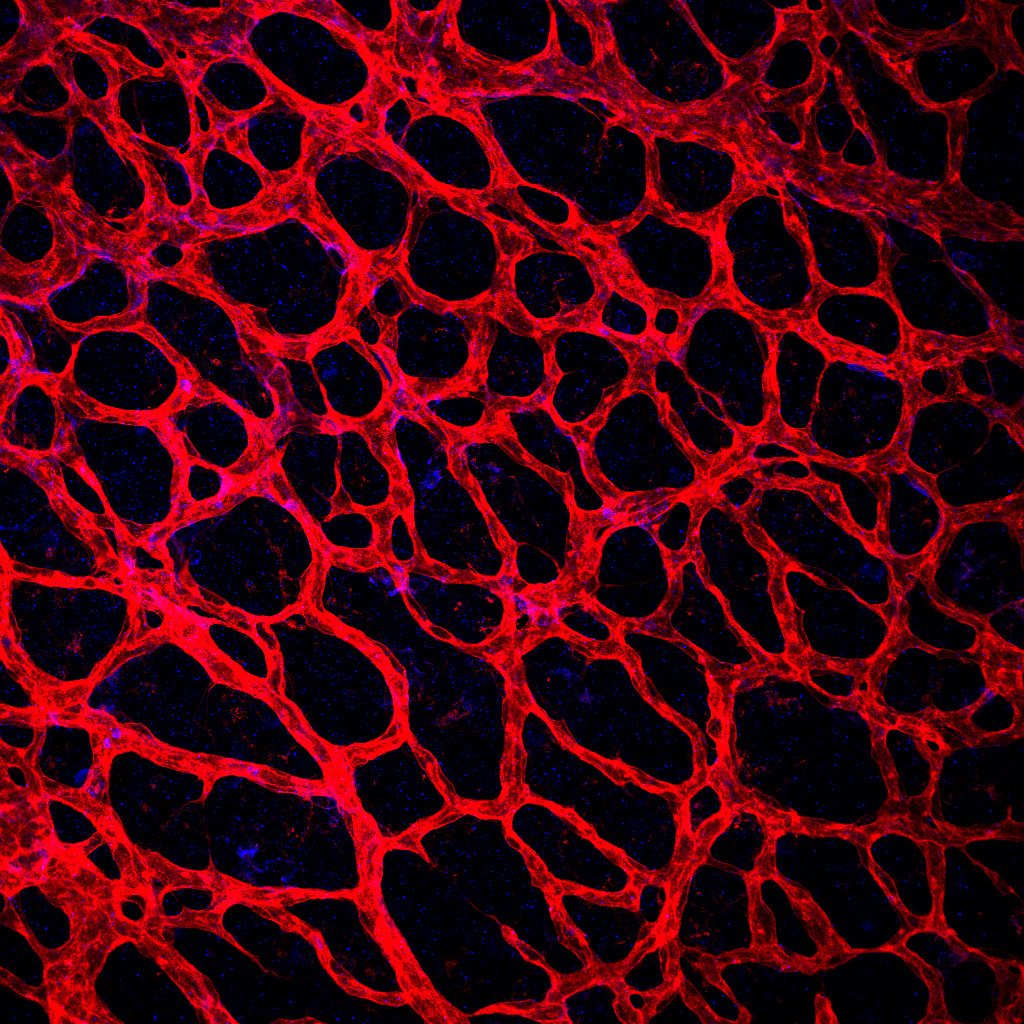

Supplement: Supplementary file 7 — Source Data for Figure 4 [file EMMM-14-e15619-s010.zip › EMM-2021-15619-V3-Figure_4_Source_Data/Fig. 4/4B/Control 40X.tif]

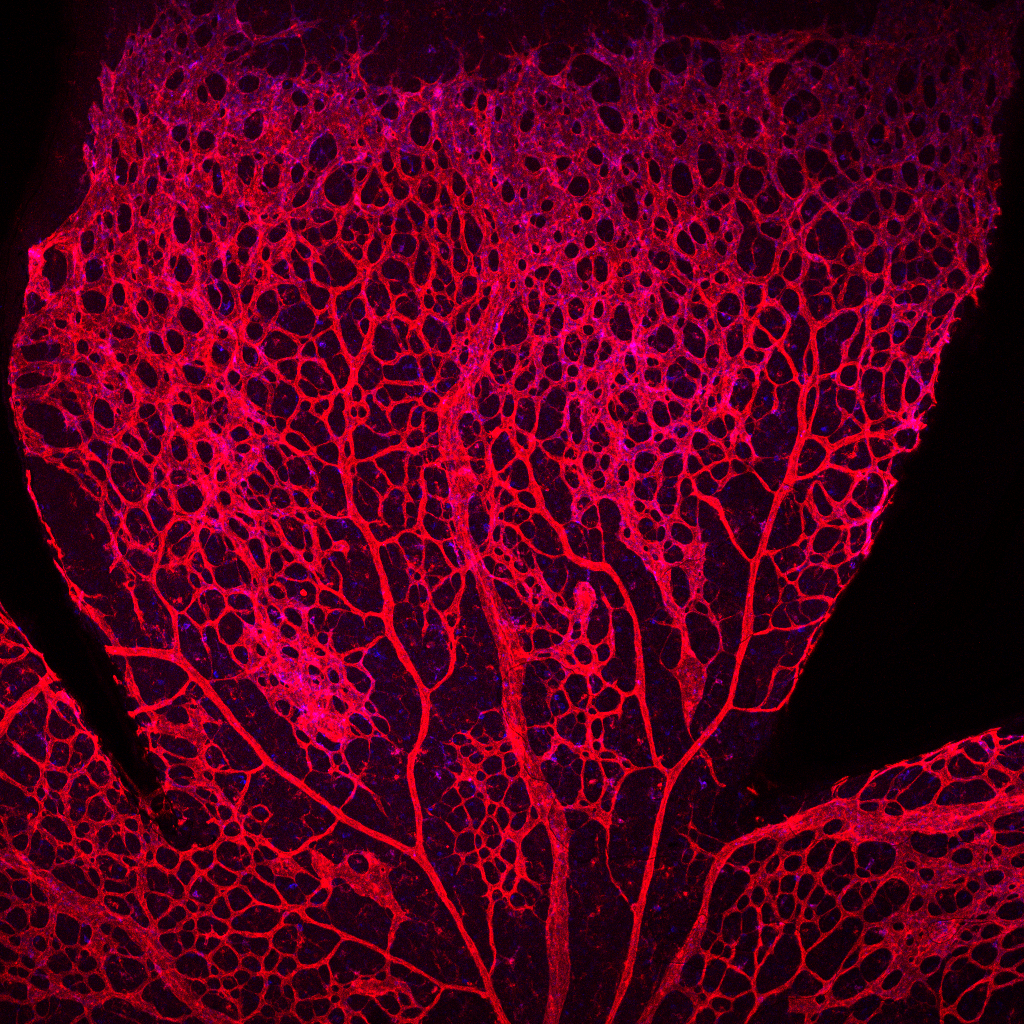

Supplement: Supplementary file 7 — Source Data for Figure 4 [file EMMM-14-e15619-s010.zip › EMM-2021-15619-V3-Figure_4_Source_Data/Fig. 4/4B/EC-Pik3caH1047R 10X.tif]

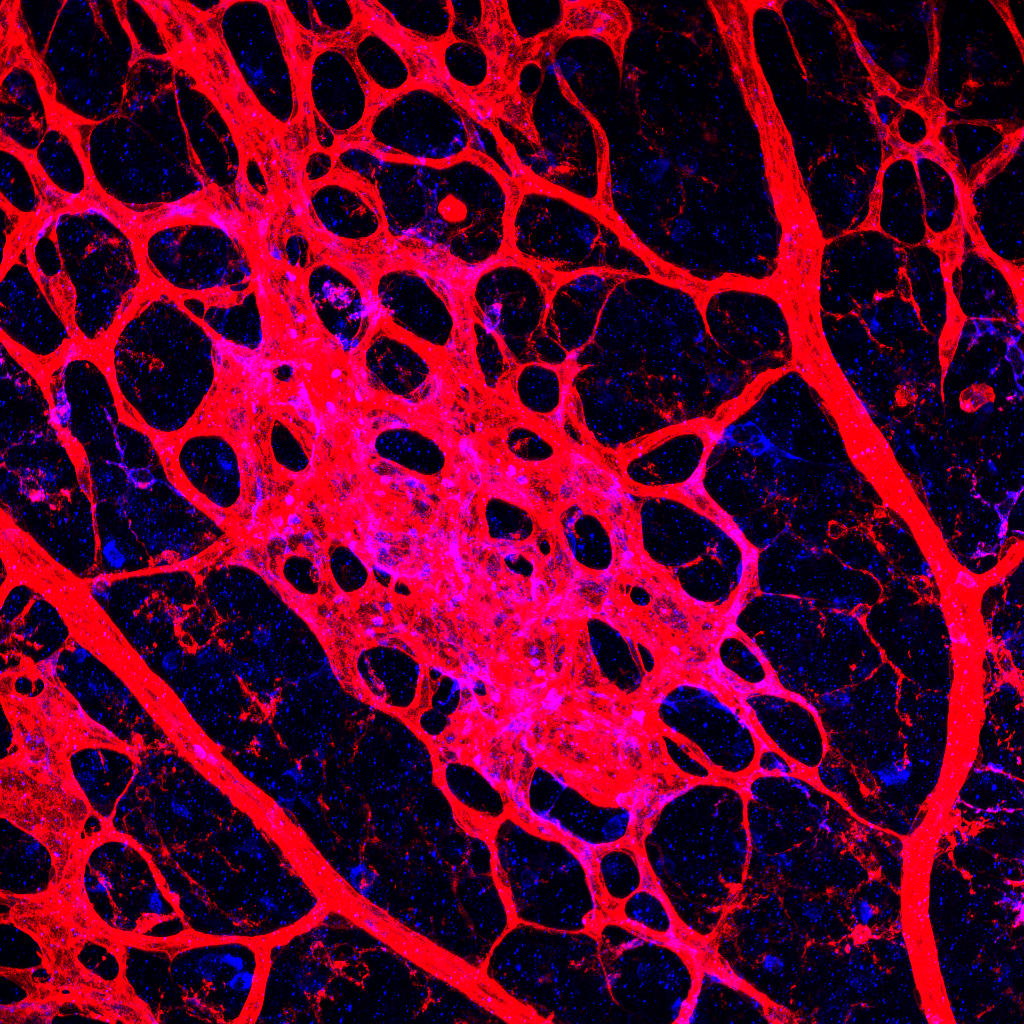

Supplement: Supplementary file 7 — Source Data for Figure 4 [file EMMM-14-e15619-s010.zip › EMM-2021-15619-V3-Figure_4_Source_Data/Fig. 4/4B/EC-Pik3caH1047R 40X.tif]

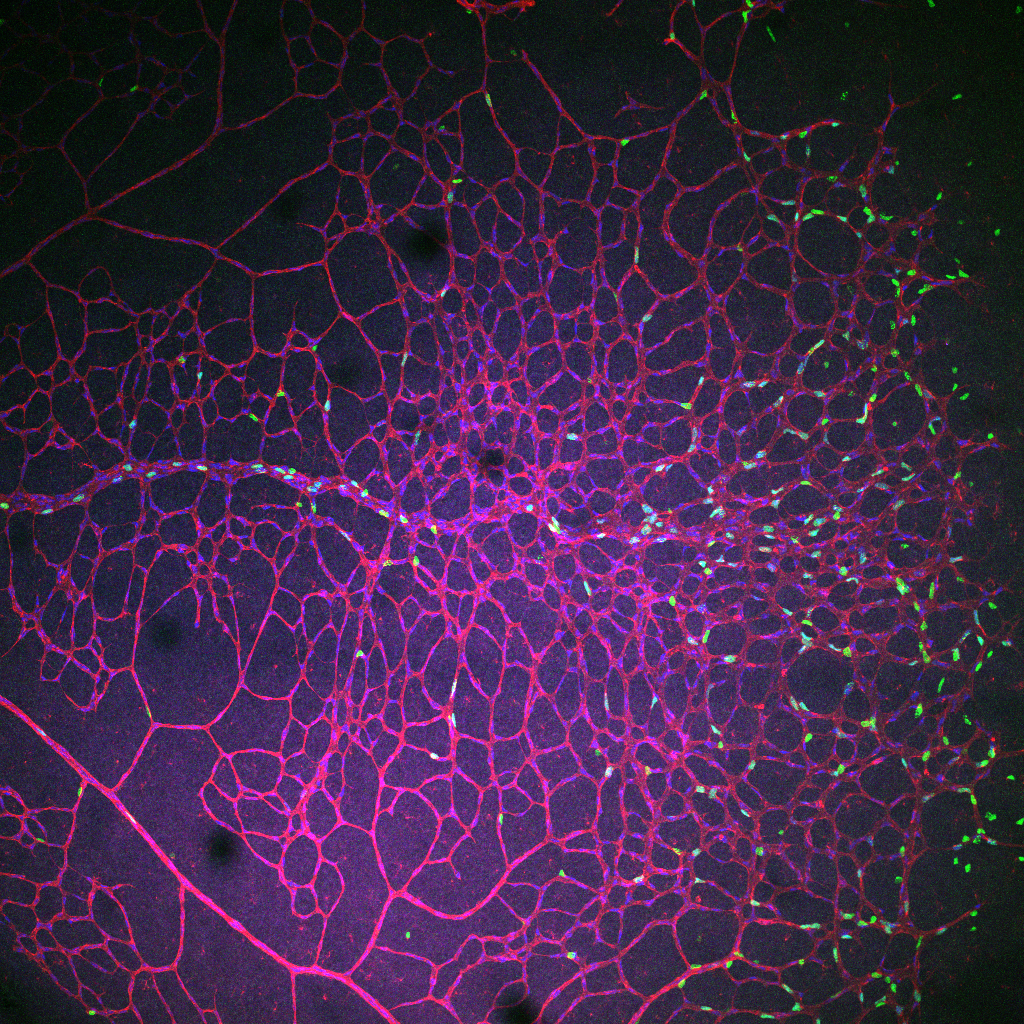

Supplement: Supplementary file 7 — Source Data for Figure 4 [file EMMM-14-e15619-s010.zip › EMM-2021-15619-V3-Figure_4_Source_Data/Fig. 4/4C/Control 10X.tif]

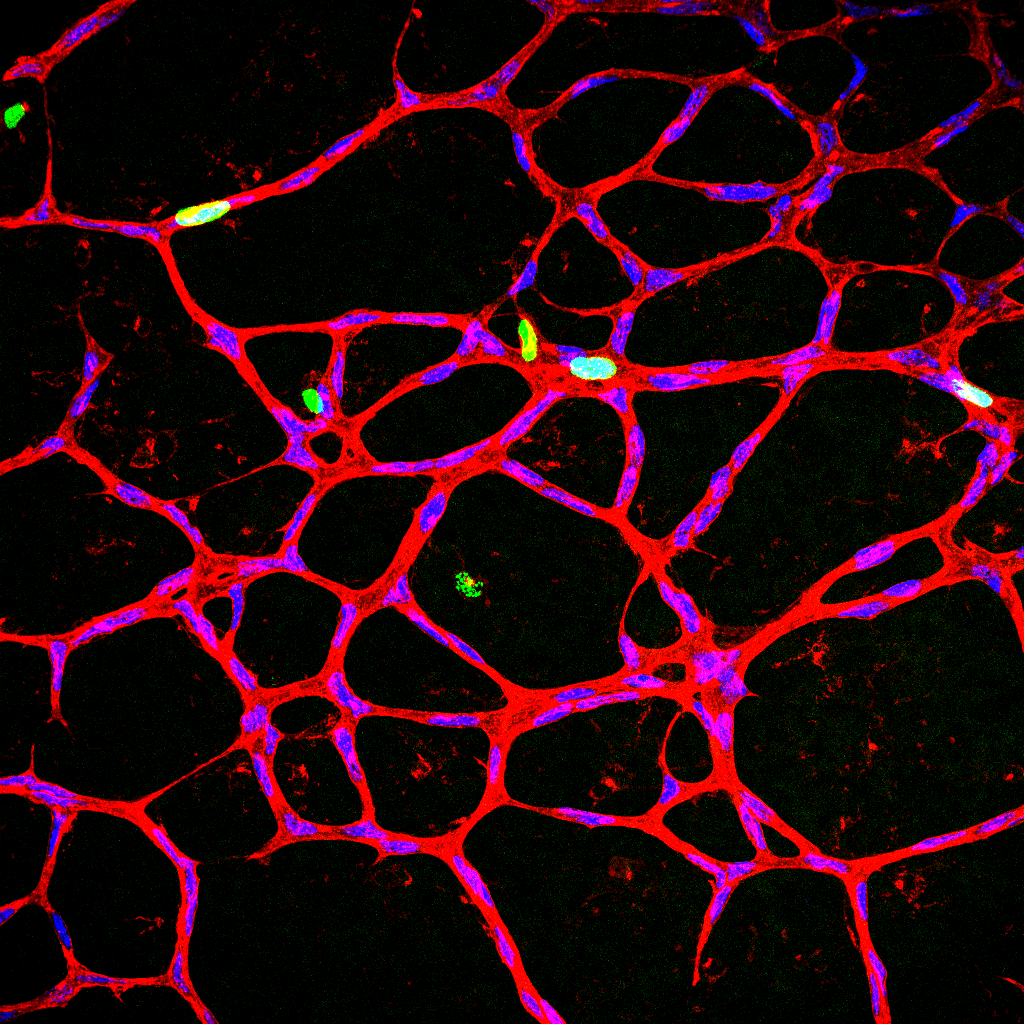

Supplement: Supplementary file 7 — Source Data for Figure 4 [file EMMM-14-e15619-s010.zip › EMM-2021-15619-V3-Figure_4_Source_Data/Fig. 4/4C/Control 40X.tif]

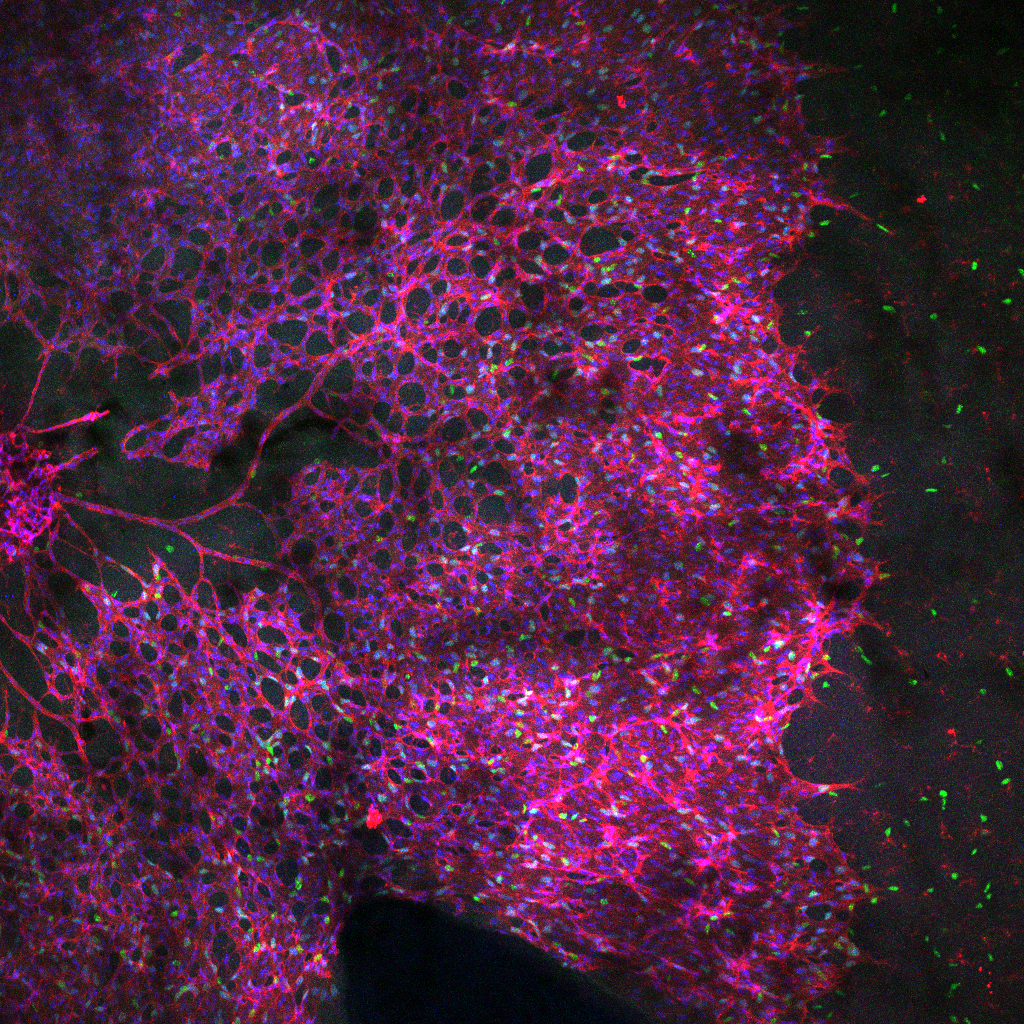

Supplement: Supplementary file 7 — Source Data for Figure 4 [file EMMM-14-e15619-s010.zip › EMM-2021-15619-V3-Figure_4_Source_Data/Fig. 4/4C/EC-Pik3caH1047R 10X.tif]

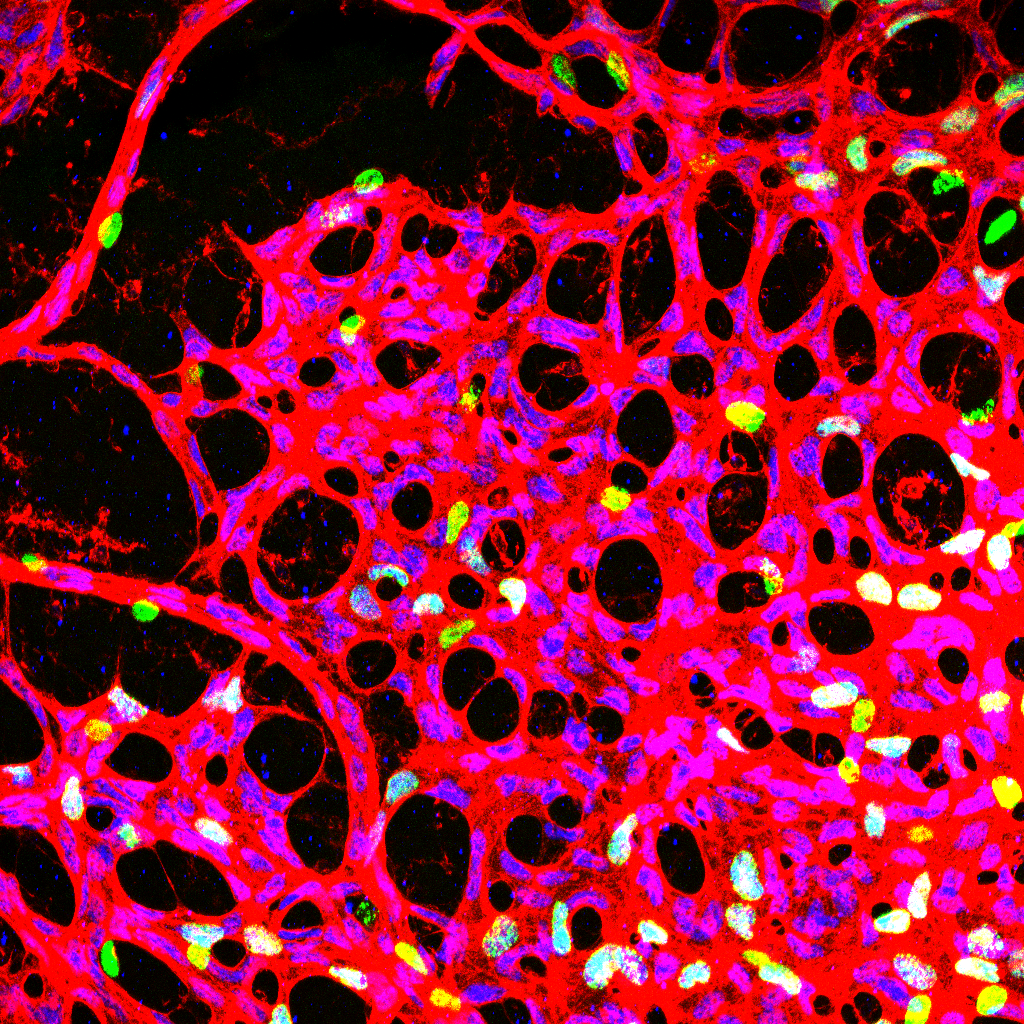

Supplement: Supplementary file 7 — Source Data for Figure 4 [file EMMM-14-e15619-s010.zip › EMM-2021-15619-V3-Figure_4_Source_Data/Fig. 4/4C/EC-Pik3caH1047R 40X.tif]

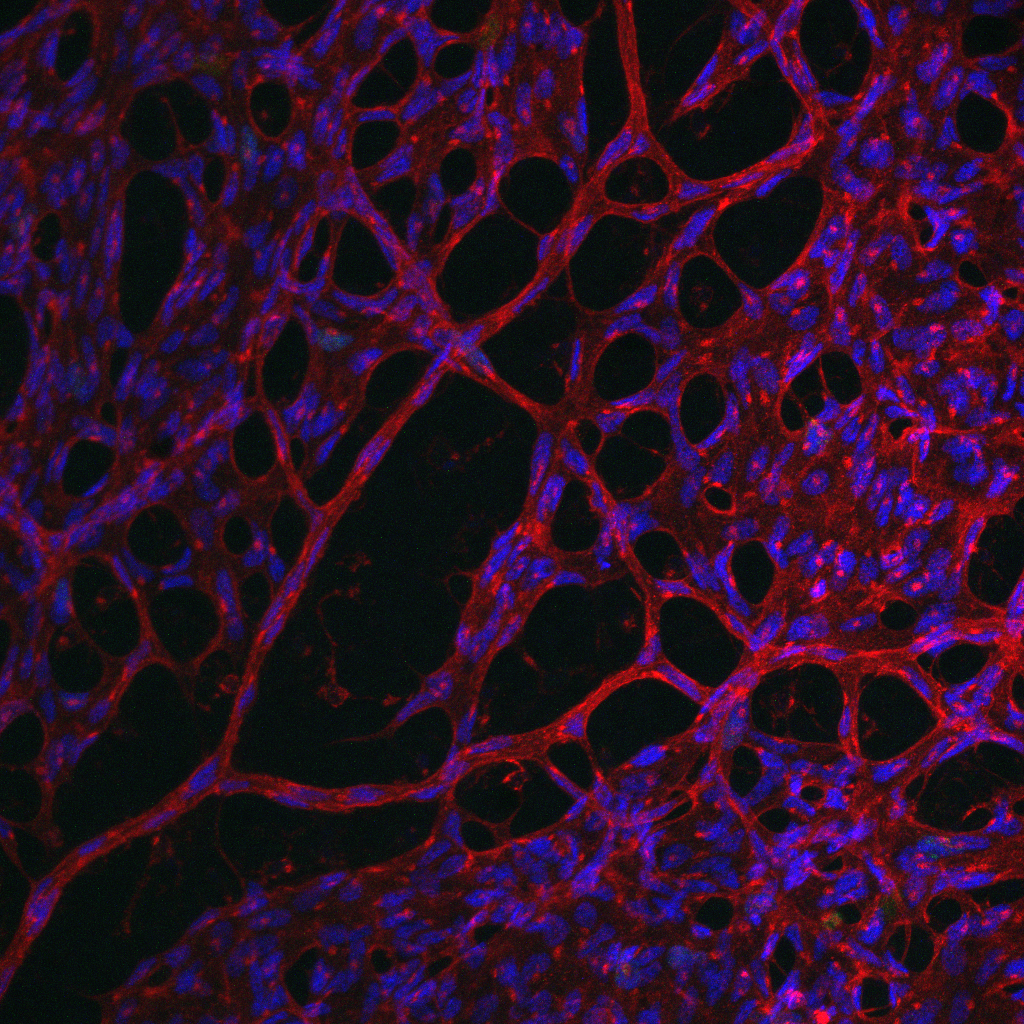

Supplement: Supplementary file 7 — Source Data for Figure 4 [file EMMM-14-e15619-s010.zip › EMM-2021-15619-V3-Figure_4_Source_Data/Fig. 4/4A/EC-Pik3caH1047R 40X 3.tif]

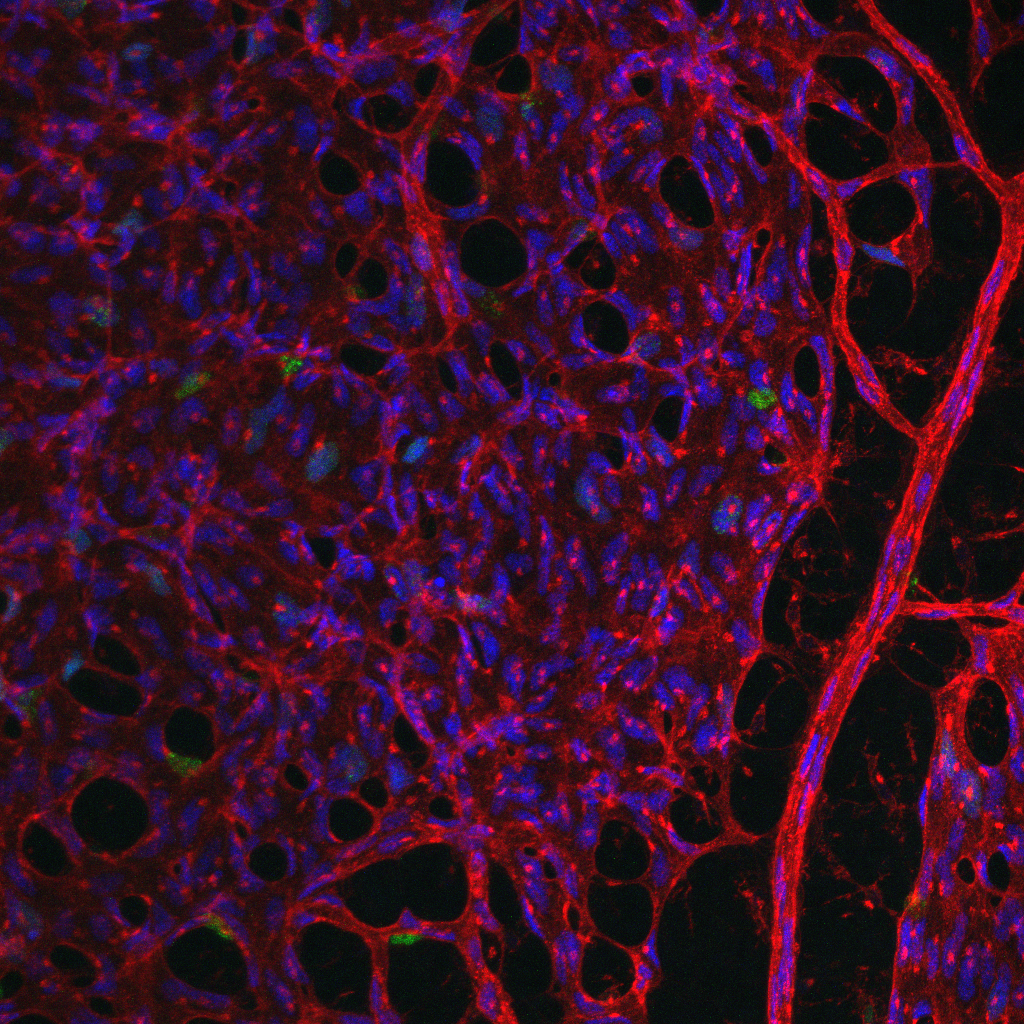

Supplement: Supplementary file 7 — Source Data for Figure 4 [file EMMM-14-e15619-s010.zip › EMM-2021-15619-V3-Figure_4_Source_Data/Fig. 4/4A/EC-Pik3caH1047R 40X 2.tif]

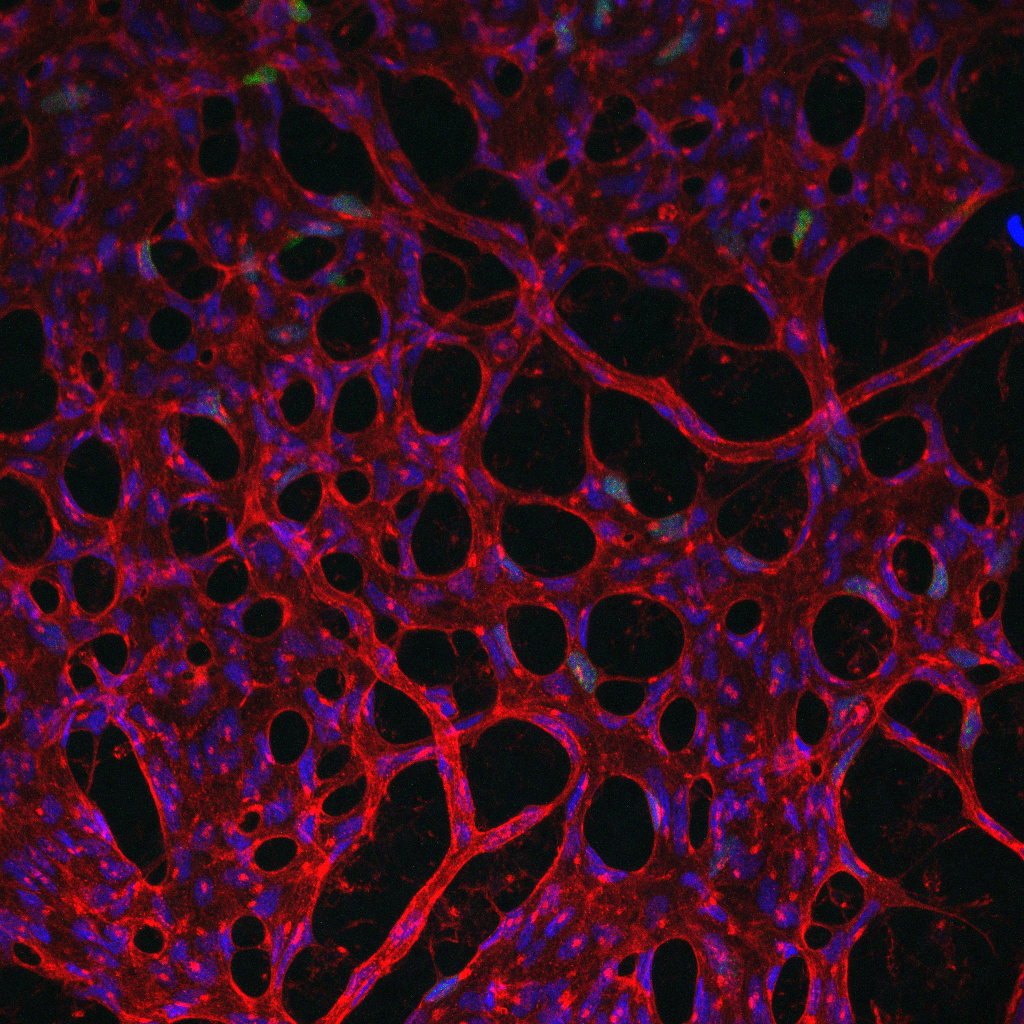

Supplement: Supplementary file 7 — Source Data for Figure 4 [file EMMM-14-e15619-s010.zip › EMM-2021-15619-V3-Figure_4_Source_Data/Fig. 4/4A/EC-Pik3caH1047R 40X 1.tif]

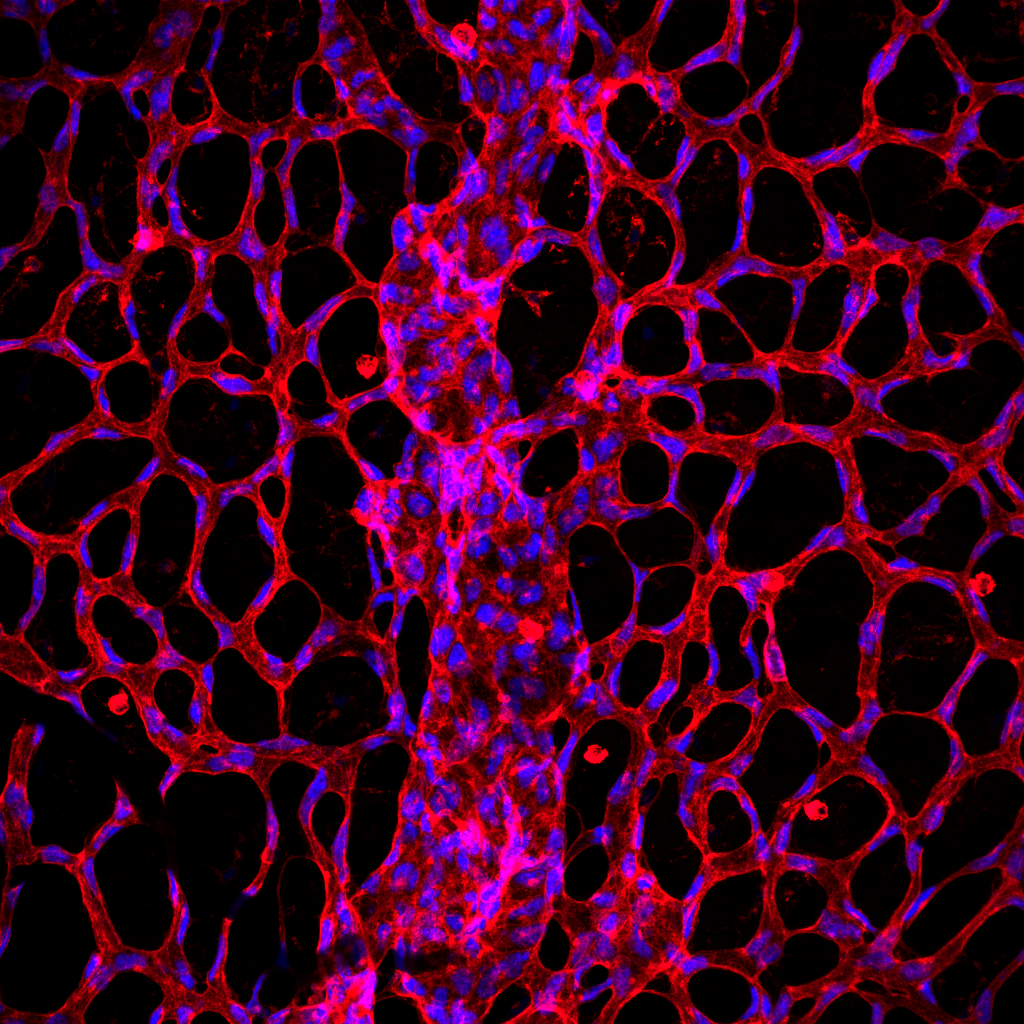

Supplement: Supplementary file 7 — Source Data for Figure 4 [file EMMM-14-e15619-s010.zip › EMM-2021-15619-V3-Figure_4_Source_Data/Fig. 4/4A/EC-Pik3caH1047R 40X 4.tif]

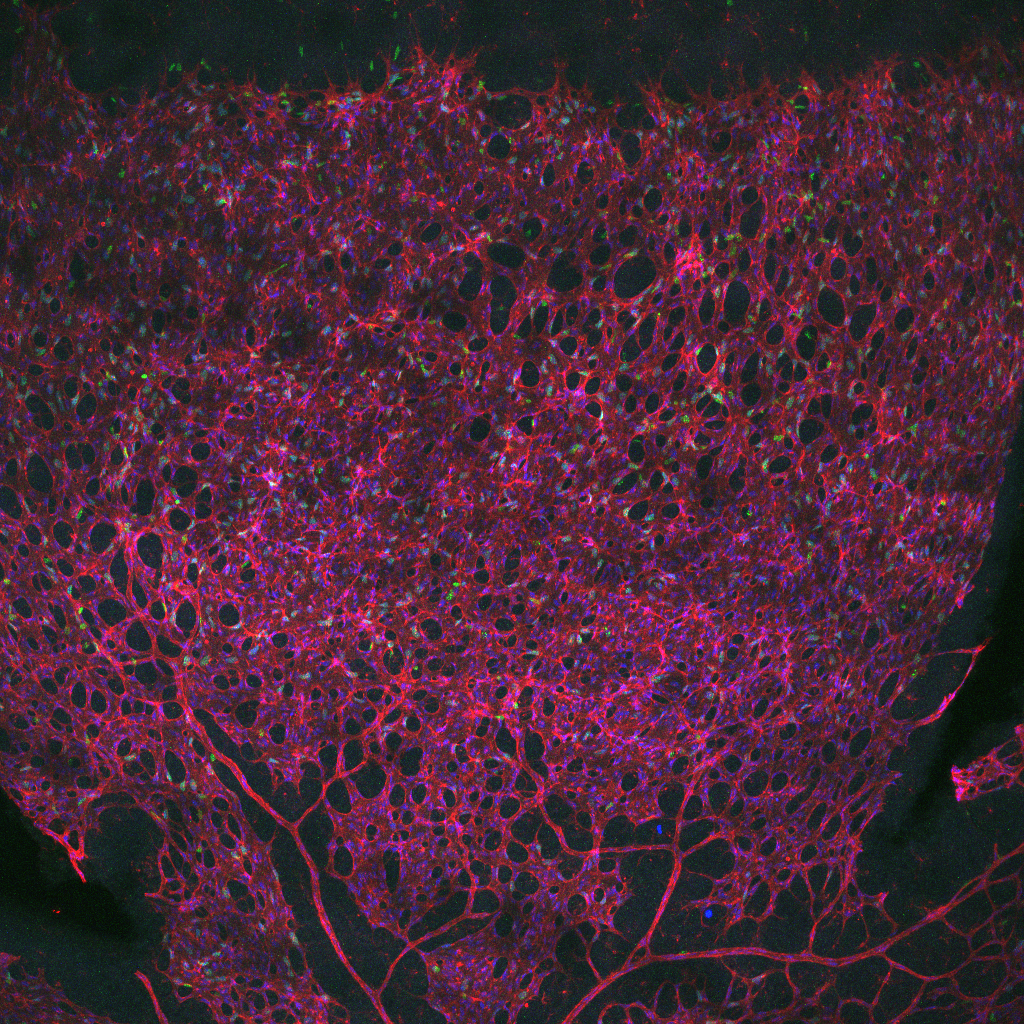

Supplement: Supplementary file 7 — Source Data for Figure 4 [file EMMM-14-e15619-s010.zip › EMM-2021-15619-V3-Figure_4_Source_Data/Fig. 4/4A/EC-Pik3caH1047R 10X.tif]

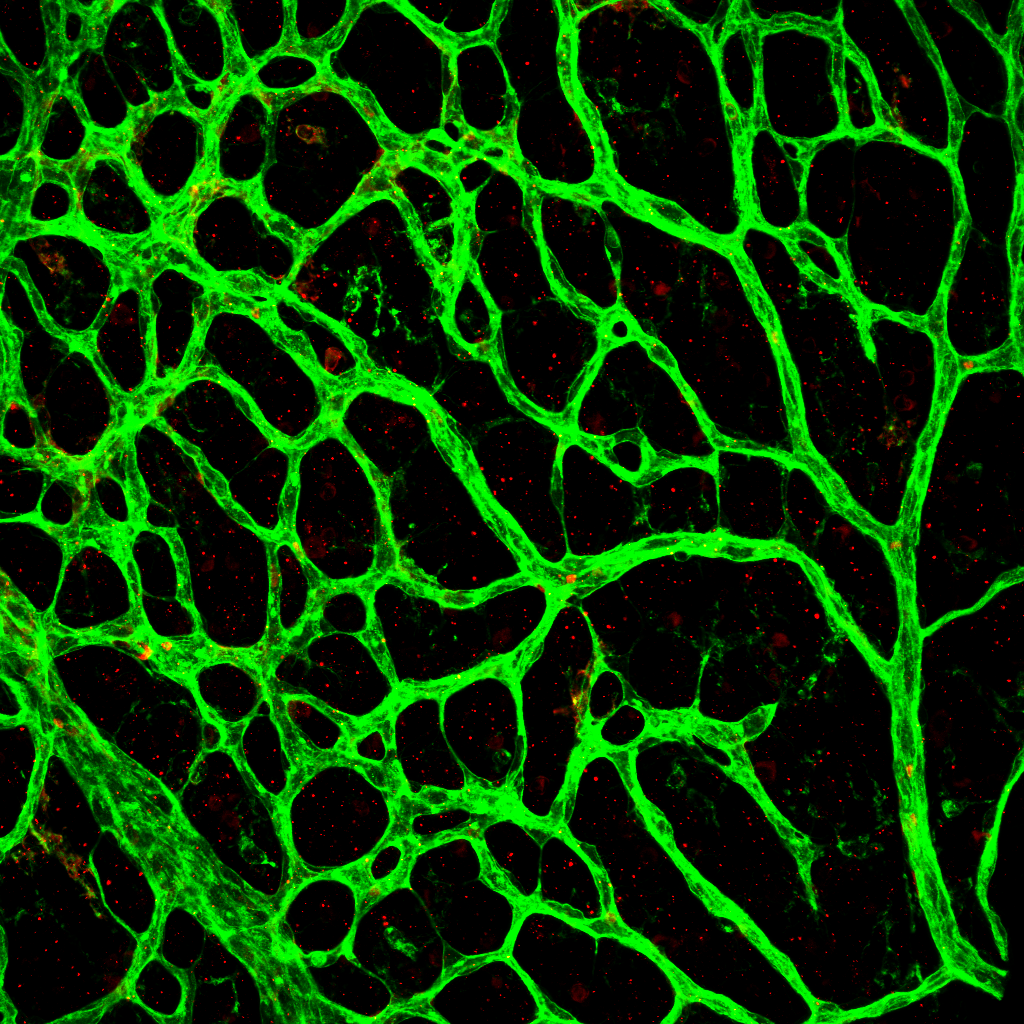

Supplement: Supplementary file 8 — Source Data for Figure 5 [file EMMM-14-e15619-s004.zip › EMM-2021-15619-V3-Figure_5_Source_Data/Fig. 5/5C/Control prevention 75mg ARQ 092 40X.tif]

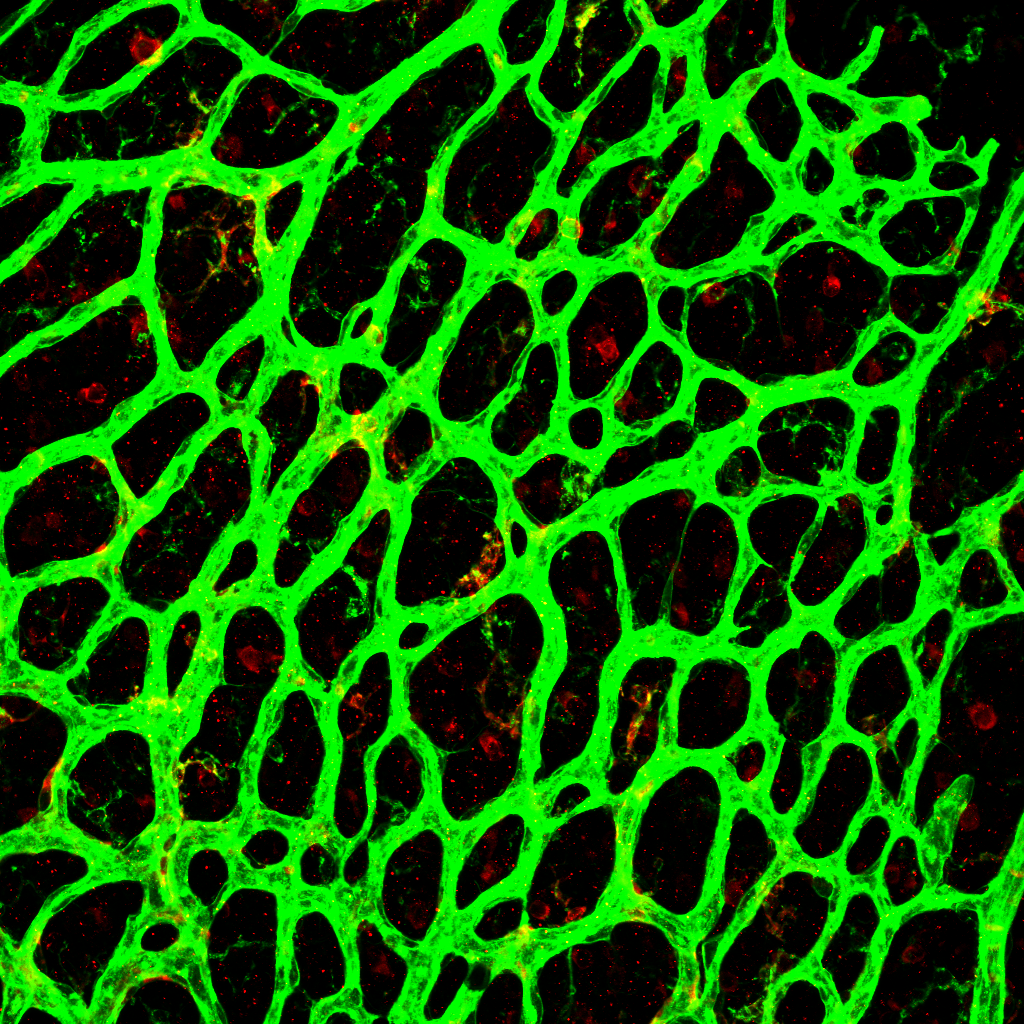

Supplement: Supplementary file 8 — Source Data for Figure 5 [file EMMM-14-e15619-s004.zip › EMM-2021-15619-V3-Figure_5_Source_Data/Fig. 5/5C/Control prevention vehicle 40X.tif]

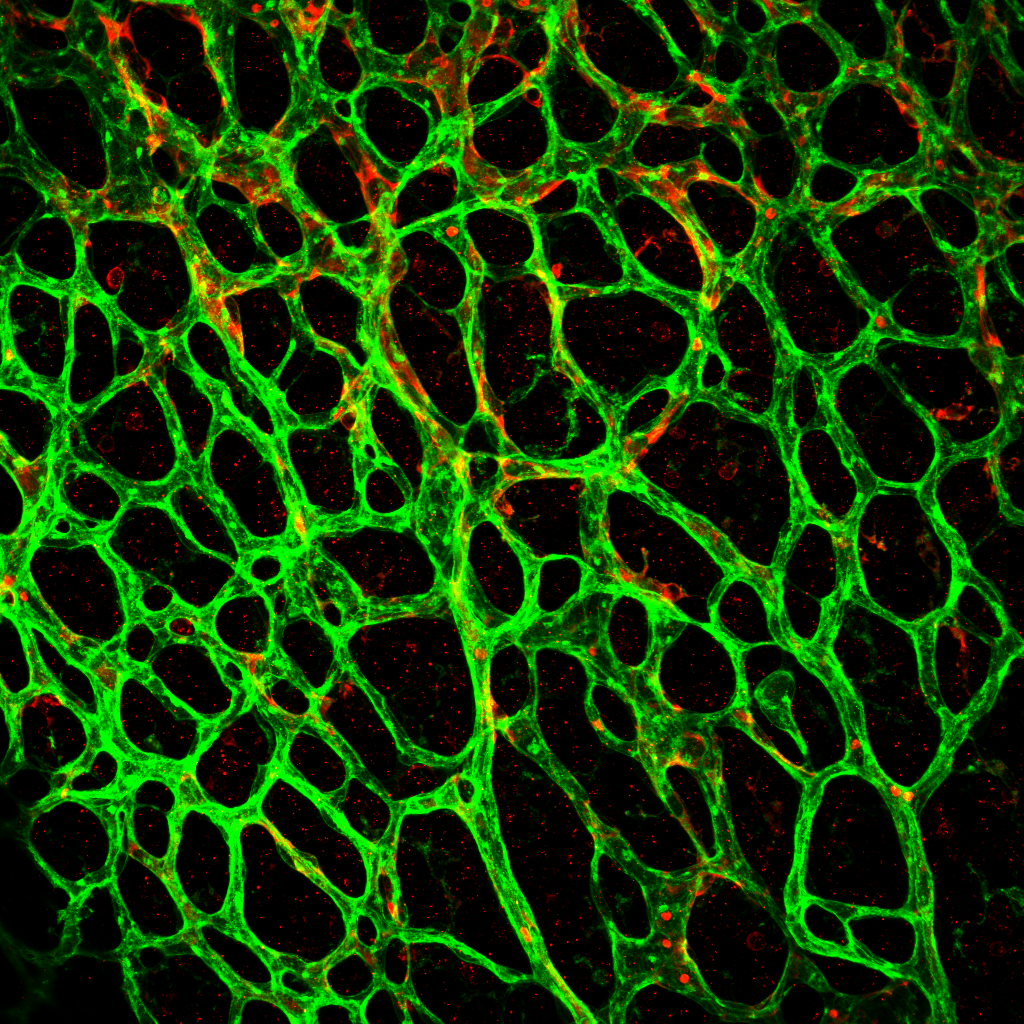

Supplement: Supplementary file 8 — Source Data for Figure 5 [file EMMM-14-e15619-s004.zip › EMM-2021-15619-V3-Figure_5_Source_Data/Fig. 5/5C/EC-Pik3caH1047R prevention 75mg ARQ 092 40X.tif]

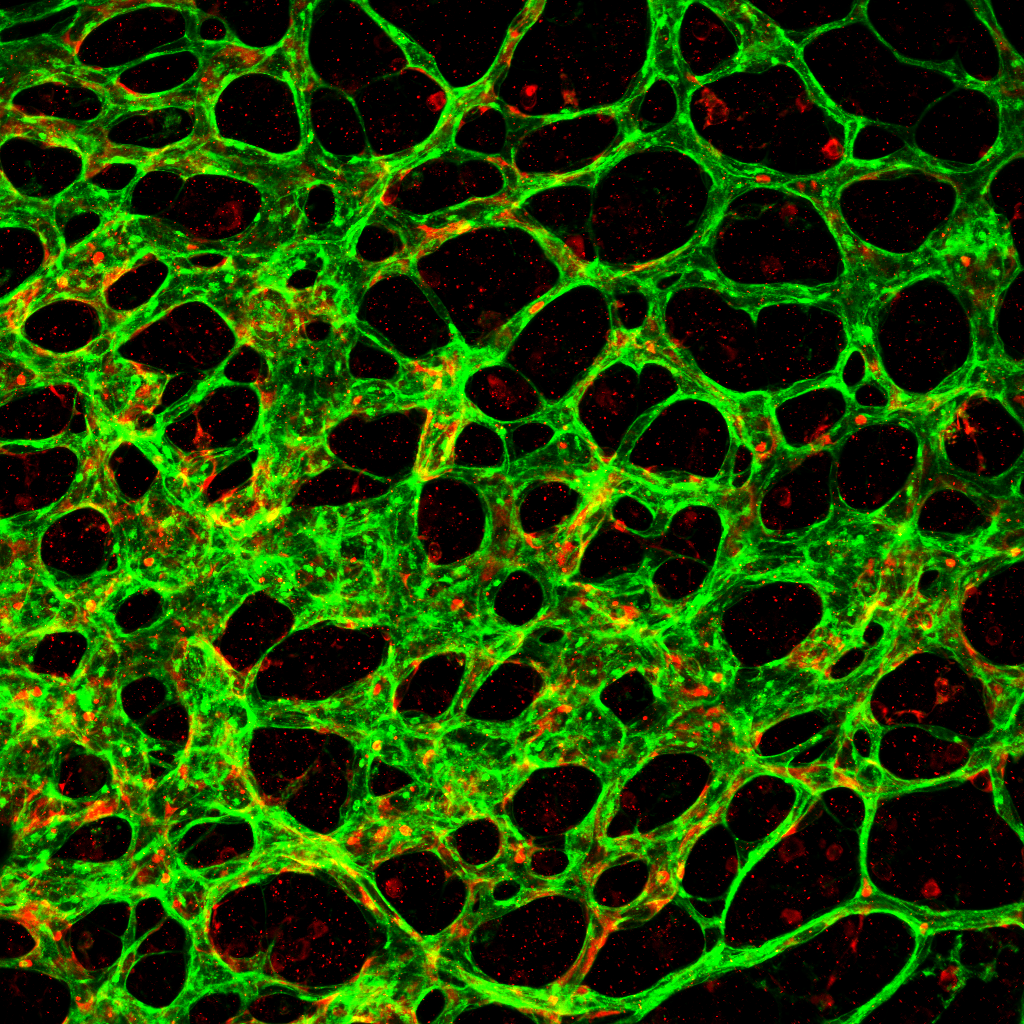

Supplement: Supplementary file 8 — Source Data for Figure 5 [file EMMM-14-e15619-s004.zip › EMM-2021-15619-V3-Figure_5_Source_Data/Fig. 5/5C/EC-Pik3caH1047R prevention vehicle 40X.tif]

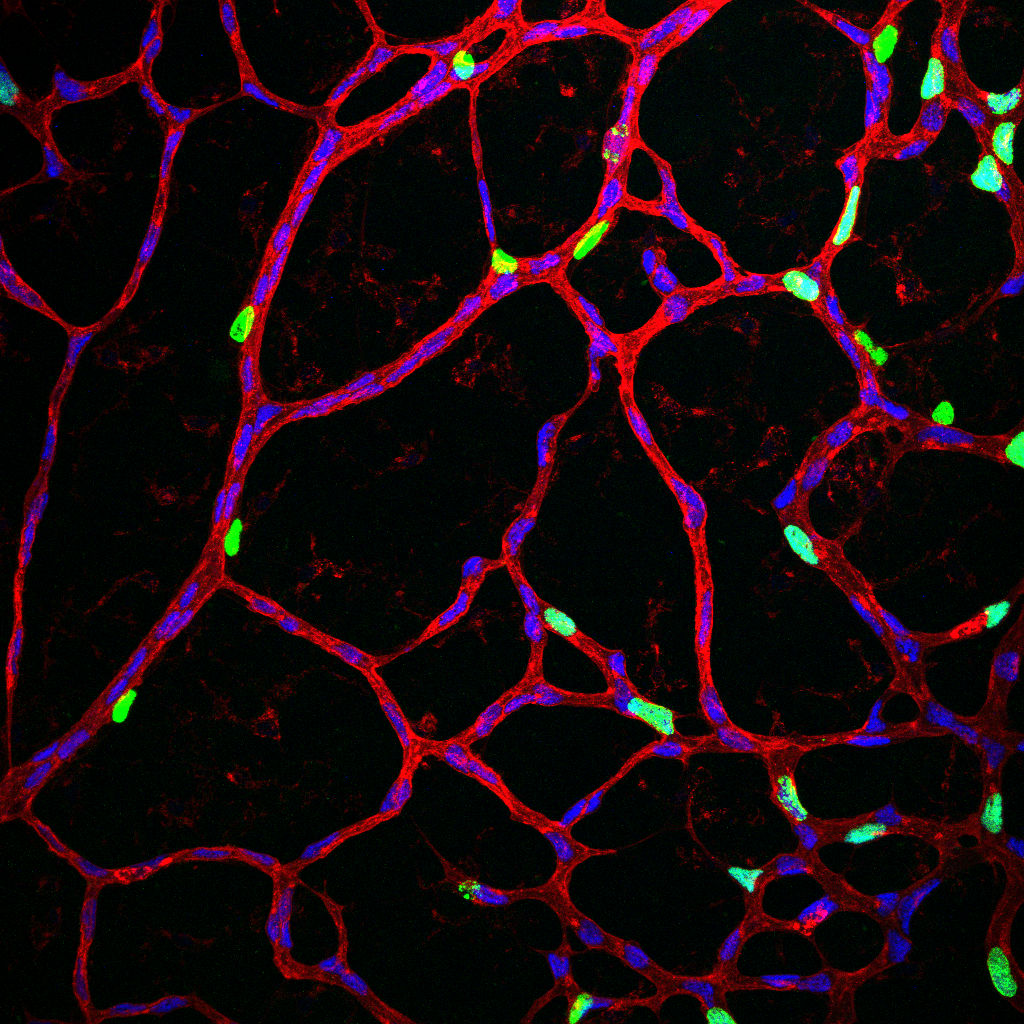

Supplement: Supplementary file 8 — Source Data for Figure 5 [file EMMM-14-e15619-s004.zip › EMM-2021-15619-V3-Figure_5_Source_Data/Fig. 5/5B/Control prevention 75mg ARQ 092 40X.tif]

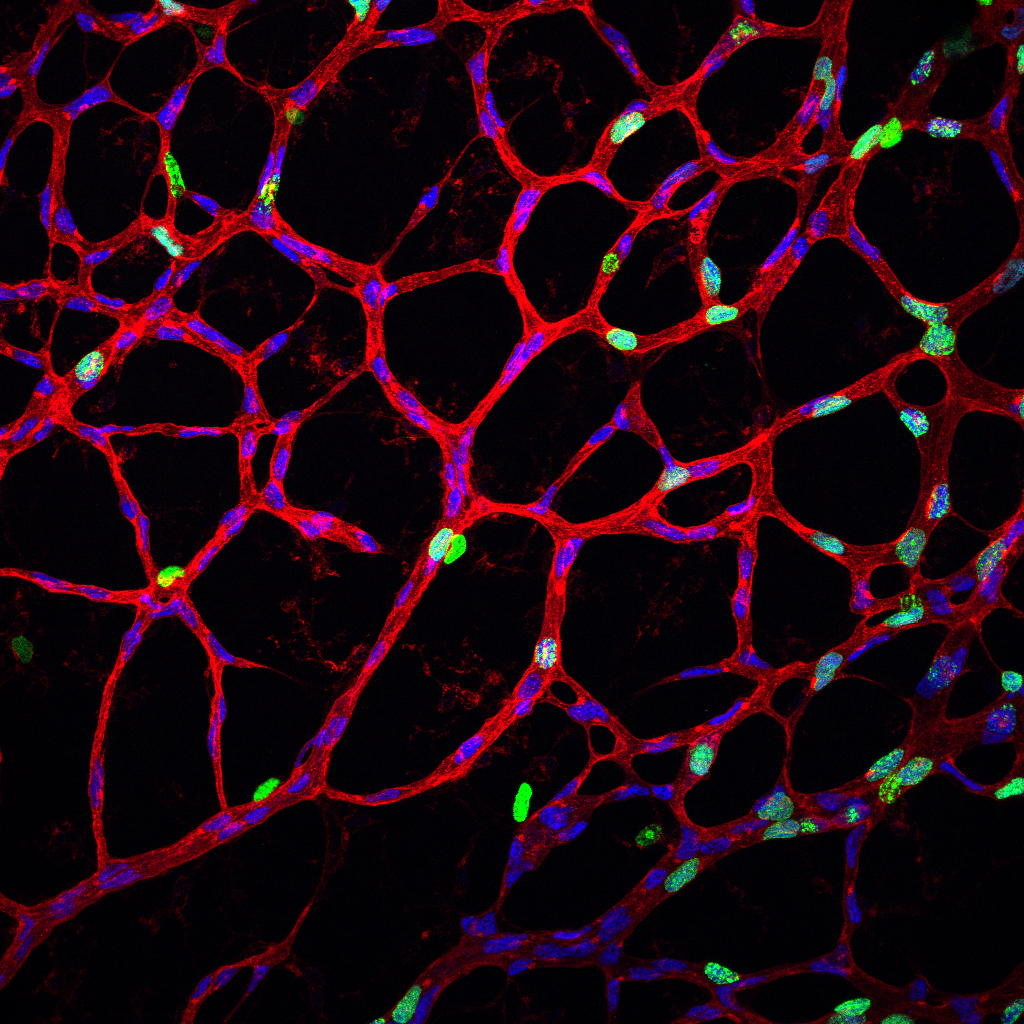

Supplement: Supplementary file 8 — Source Data for Figure 5 [file EMMM-14-e15619-s004.zip › EMM-2021-15619-V3-Figure_5_Source_Data/Fig. 5/5B/EC-Pik3caH1047R prevention 75 mg ARQ 092 40X.tif]

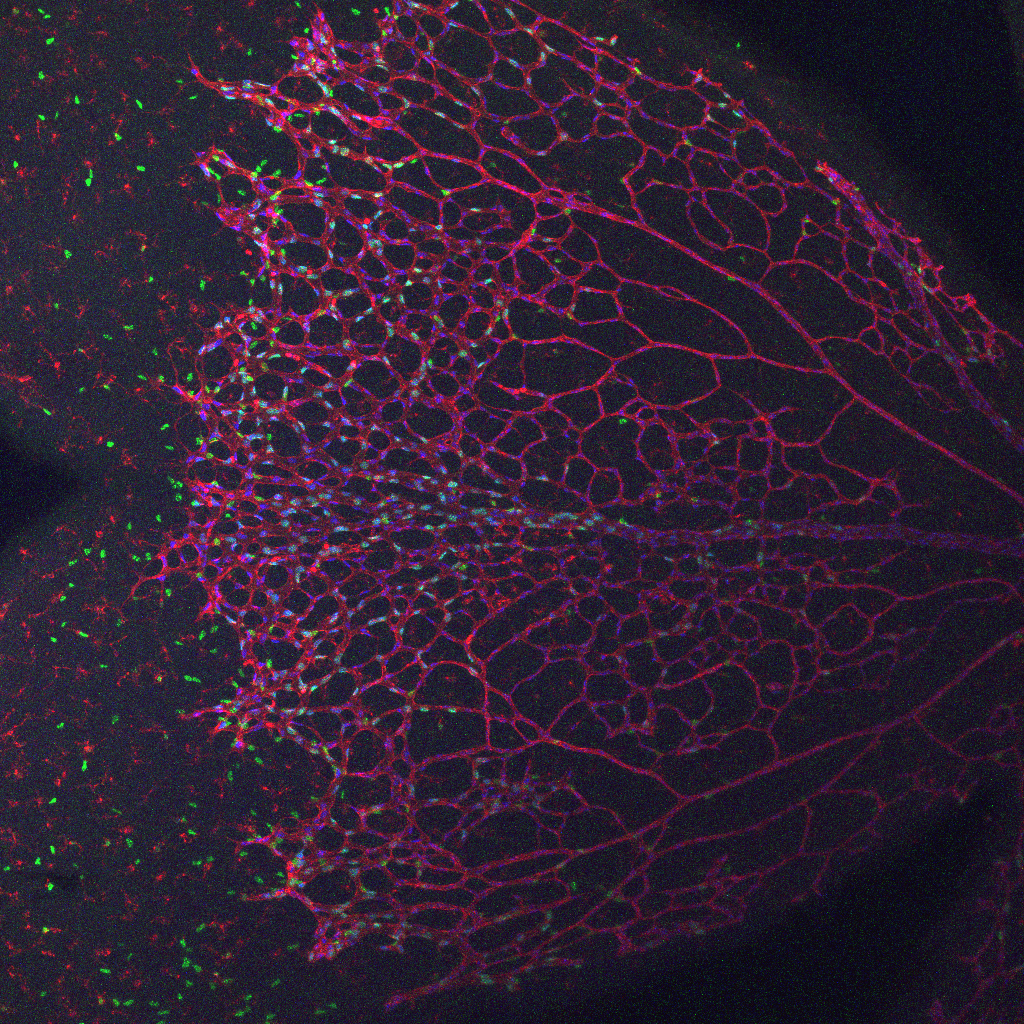

Supplement: Supplementary file 8 — Source Data for Figure 5 [file EMMM-14-e15619-s004.zip › EMM-2021-15619-V3-Figure_5_Source_Data/Fig. 5/5B/EC-Pik3caH1047R prevention 75 mg ARQ 092 10X.tif]

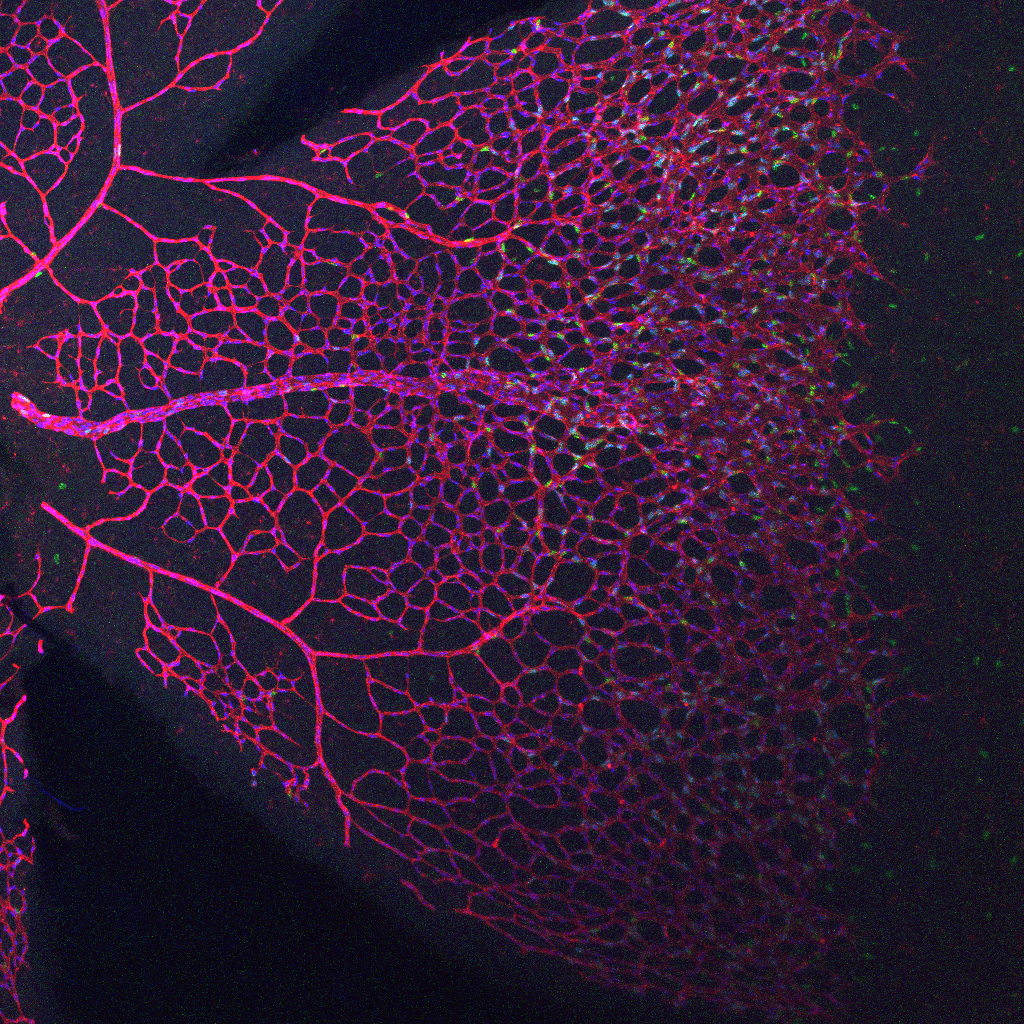

Supplement: Supplementary file 8 — Source Data for Figure 5 [file EMMM-14-e15619-s004.zip › EMM-2021-15619-V3-Figure_5_Source_Data/Fig. 5/5B/Control prevention 75mg ARQ 092 10X.tif]

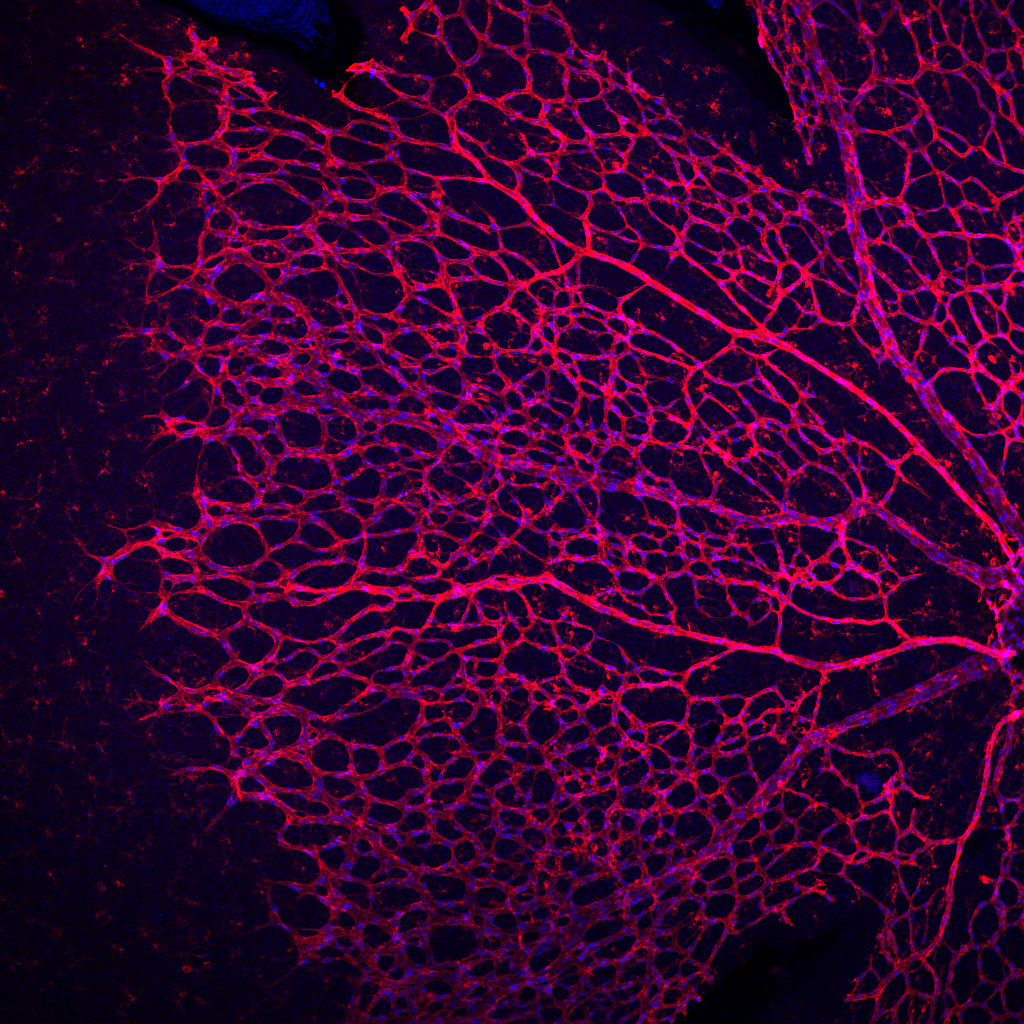

Supplement: Supplementary file 8 — Source Data for Figure 5 [file EMMM-14-e15619-s004.zip › EMM-2021-15619-V3-Figure_5_Source_Data/Fig. 5/5B/Control prevention vehicle 10X.tif]

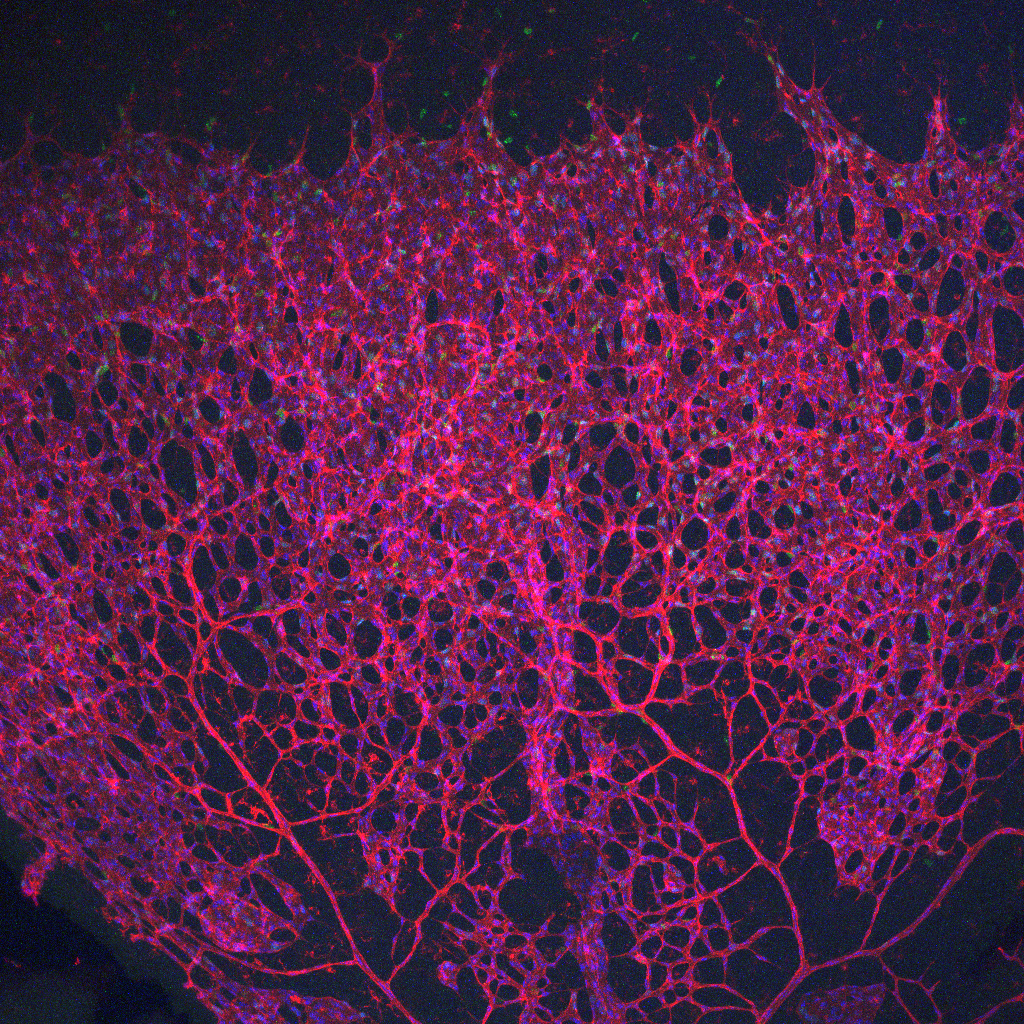

Supplement: Supplementary file 8 — Source Data for Figure 5 [file EMMM-14-e15619-s004.zip › EMM-2021-15619-V3-Figure_5_Source_Data/Fig. 5/5B/EC-Pik3caH1047R prevention vehicle 10X.tif]

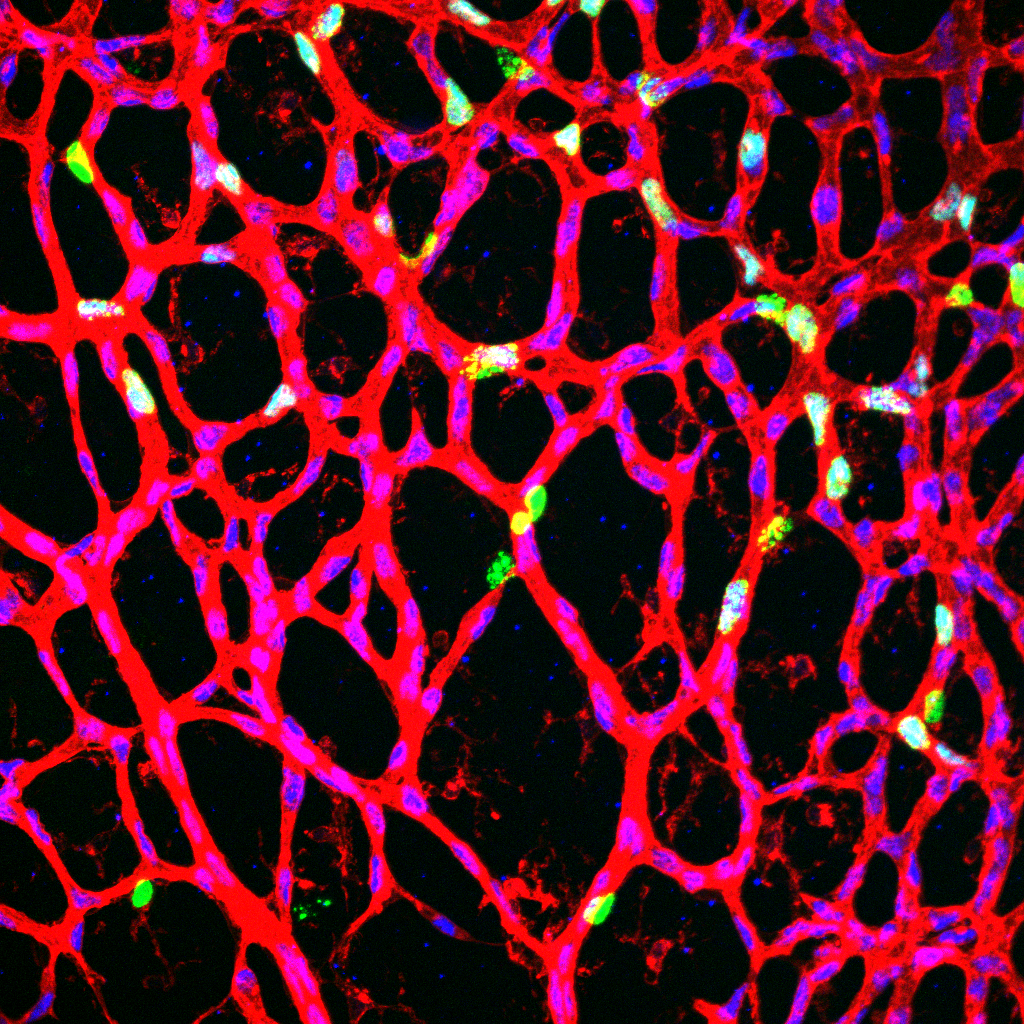

Supplement: Supplementary file 8 — Source Data for Figure 5 [file EMMM-14-e15619-s004.zip › EMM-2021-15619-V3-Figure_5_Source_Data/Fig. 5/5B/Control prevention vehicle 40X.tif]

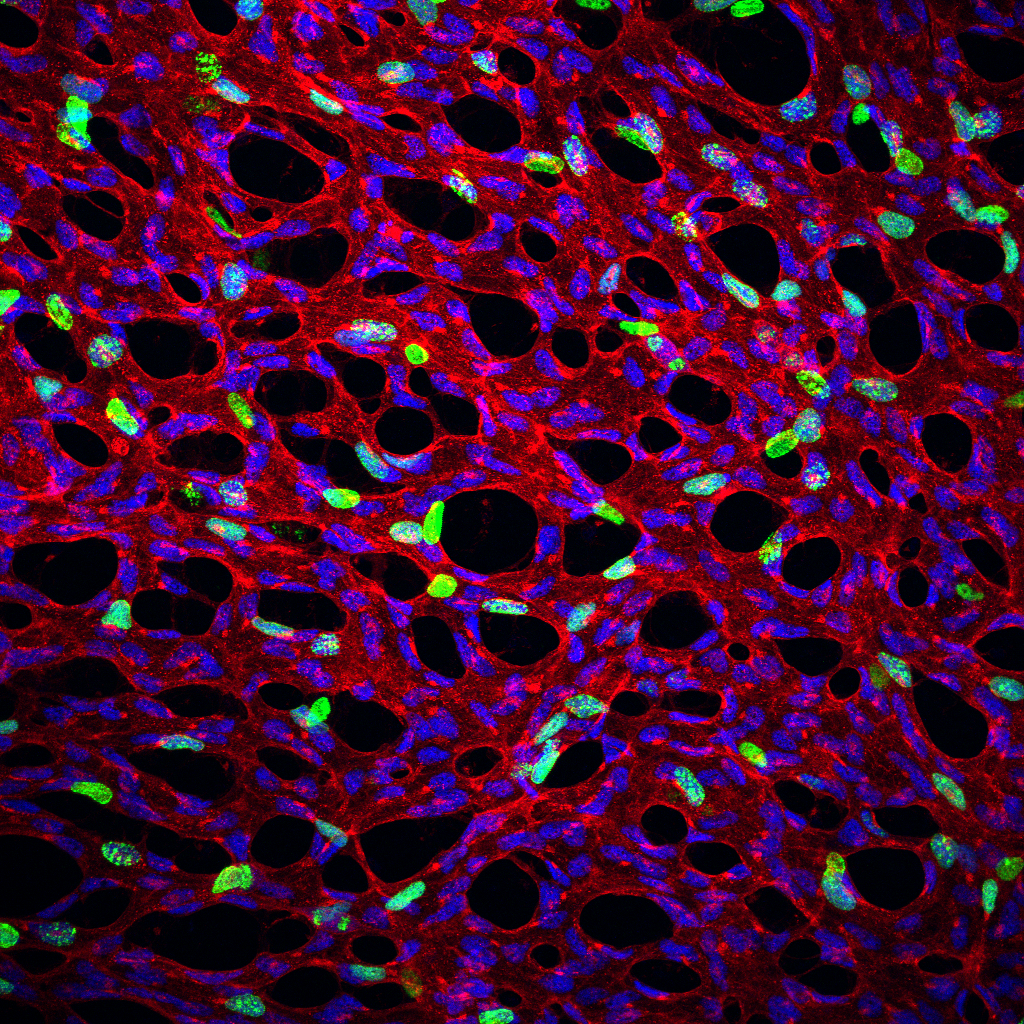

Supplement: Supplementary file 8 — Source Data for Figure 5 [file EMMM-14-e15619-s004.zip › EMM-2021-15619-V3-Figure_5_Source_Data/Fig. 5/5B/EC-Pik3caH1047R prevention vehicle 40X.tif]

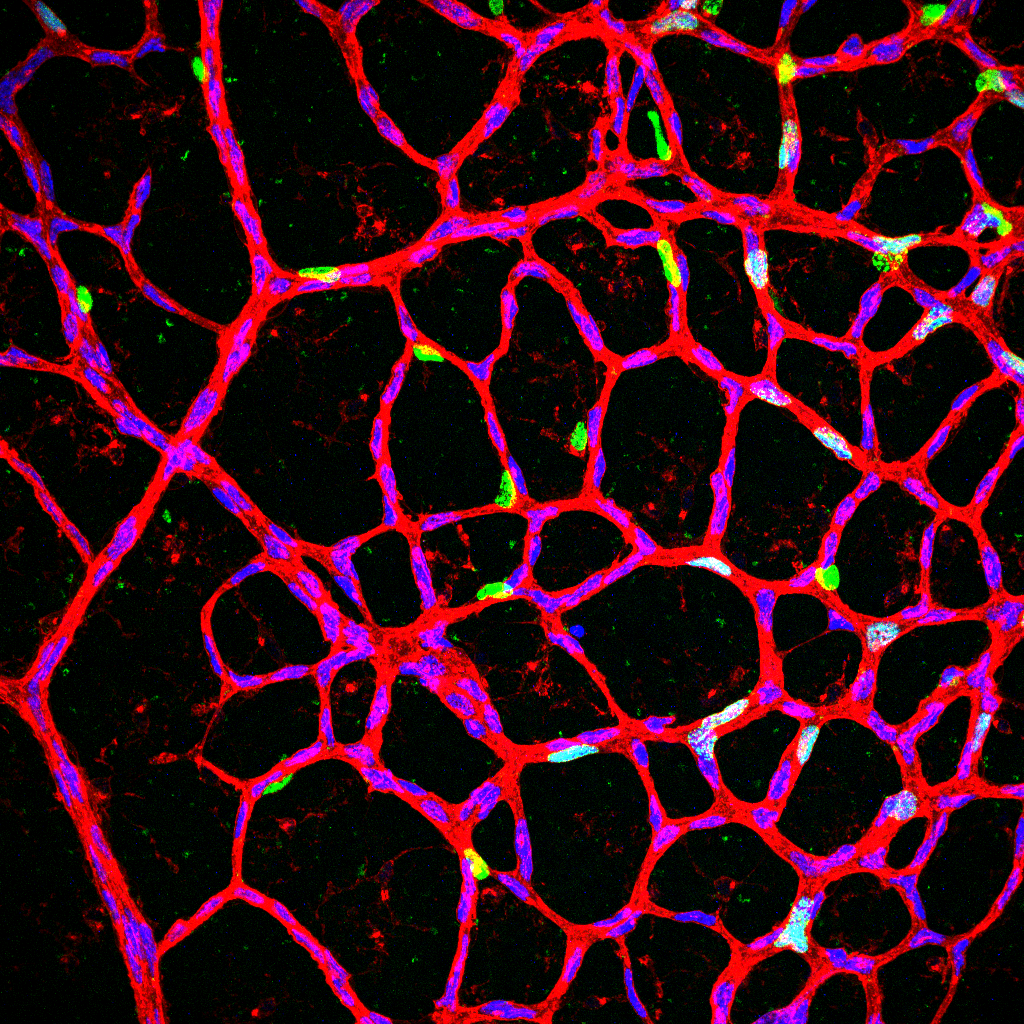

Supplement: Supplementary file 9 — Source Data for Figure 6 [file EMMM-14-e15619-s002.zip › EMM-2021-15619-V3-Figure_6_Source_Data/Fig. 6/6B/EC-Pik3caH1047R prevention 35 mg ARQ 092 40X.tif]

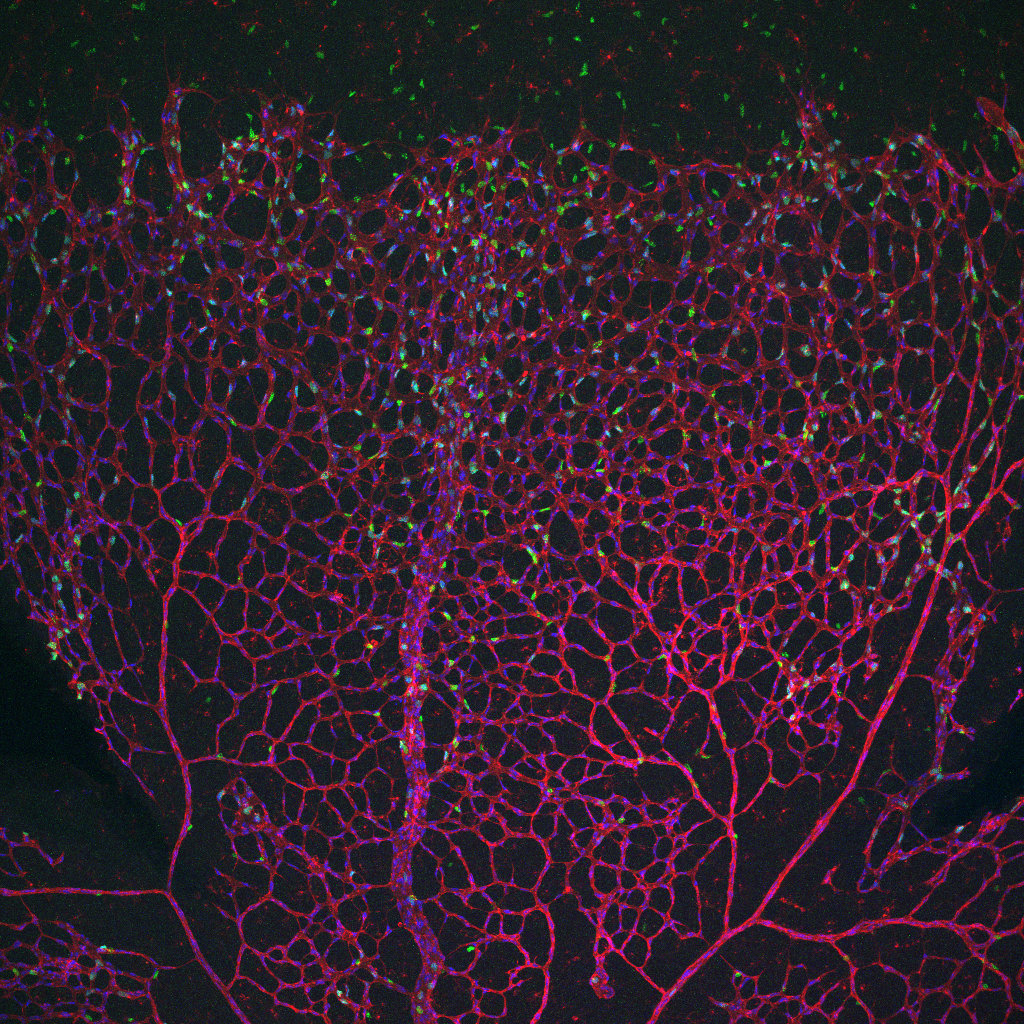

Supplement: Supplementary file 9 — Source Data for Figure 6 [file EMMM-14-e15619-s002.zip › EMM-2021-15619-V3-Figure_6_Source_Data/Fig. 6/6B/EC-Pik3caH1047R prevention 35 mg ARQ 092 10X.tif]

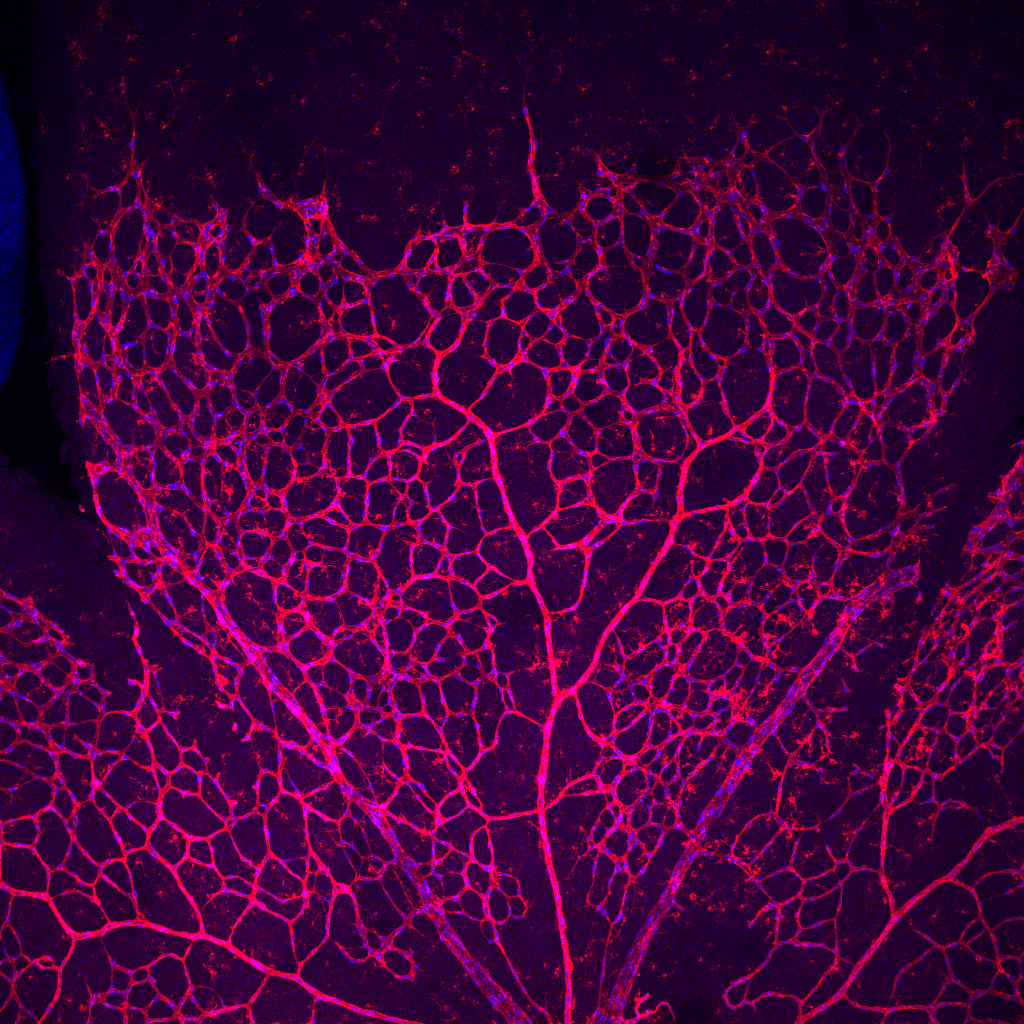

Supplement: Supplementary file 9 — Source Data for Figure 6 [file EMMM-14-e15619-s002.zip › EMM-2021-15619-V3-Figure_6_Source_Data/Fig. 6/6B/Control prevention 35 mg ARQ 092 10X.tif]

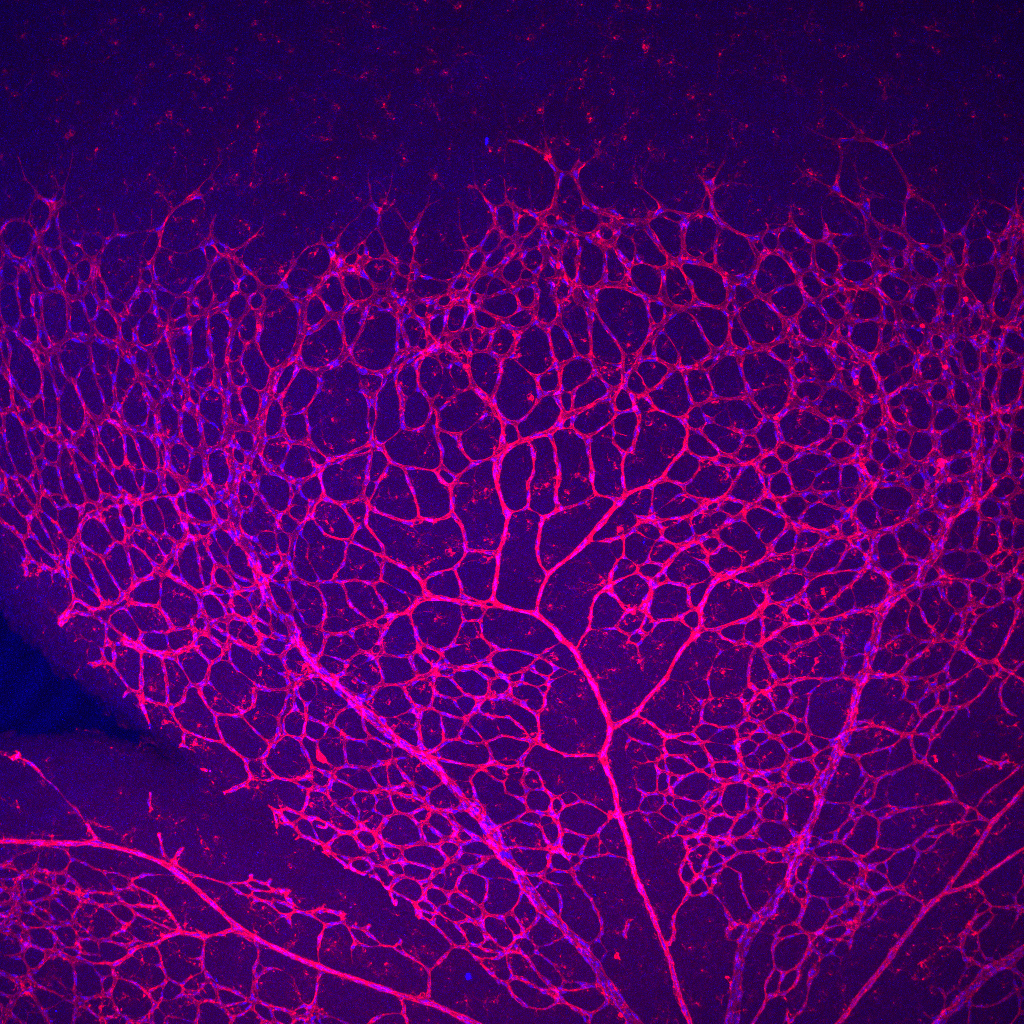

Supplement: Supplementary file 9 — Source Data for Figure 6 [file EMMM-14-e15619-s002.zip › EMM-2021-15619-V3-Figure_6_Source_Data/Fig. 6/6B/Control prevention vehicle 10X.tif]

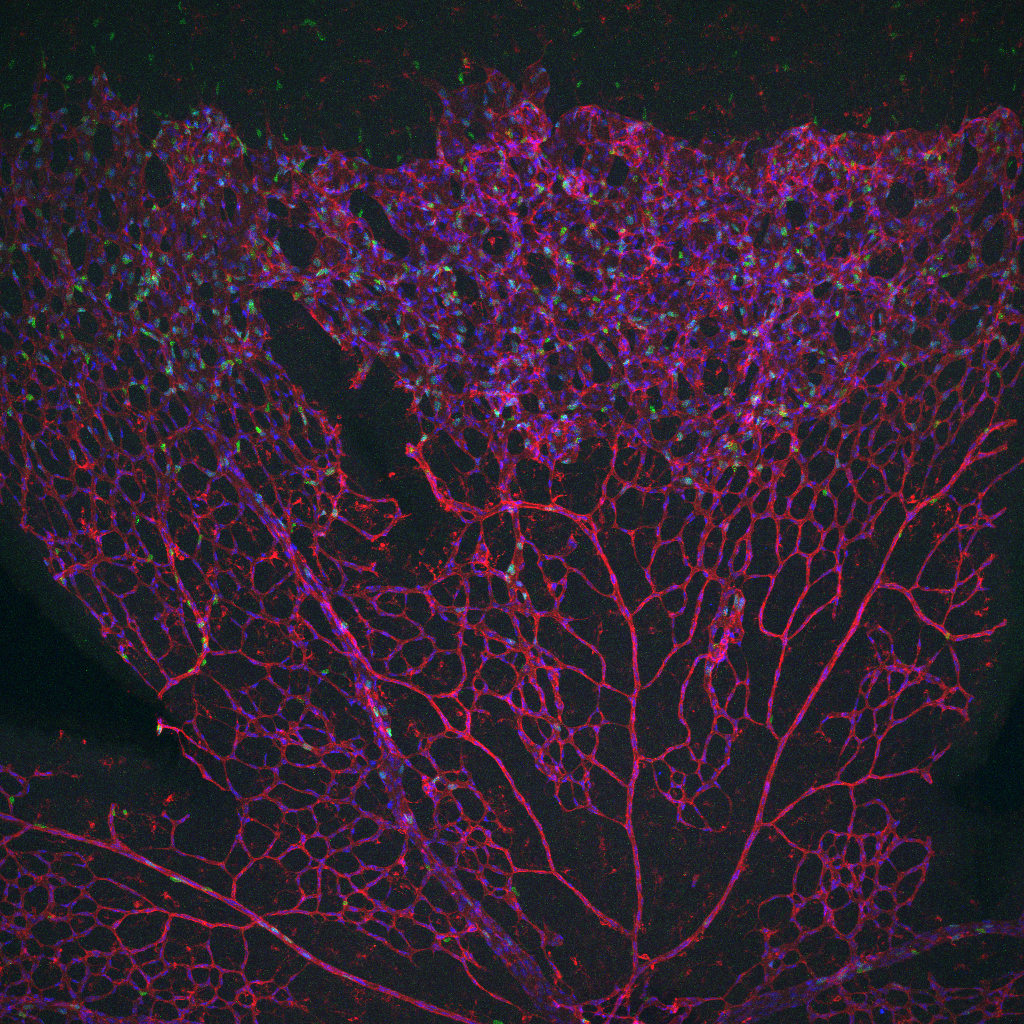

Supplement: Supplementary file 9 — Source Data for Figure 6 [file EMMM-14-e15619-s002.zip › EMM-2021-15619-V3-Figure_6_Source_Data/Fig. 6/6B/EC-Pik3caH1047R prevention vehicle 10X.tif]

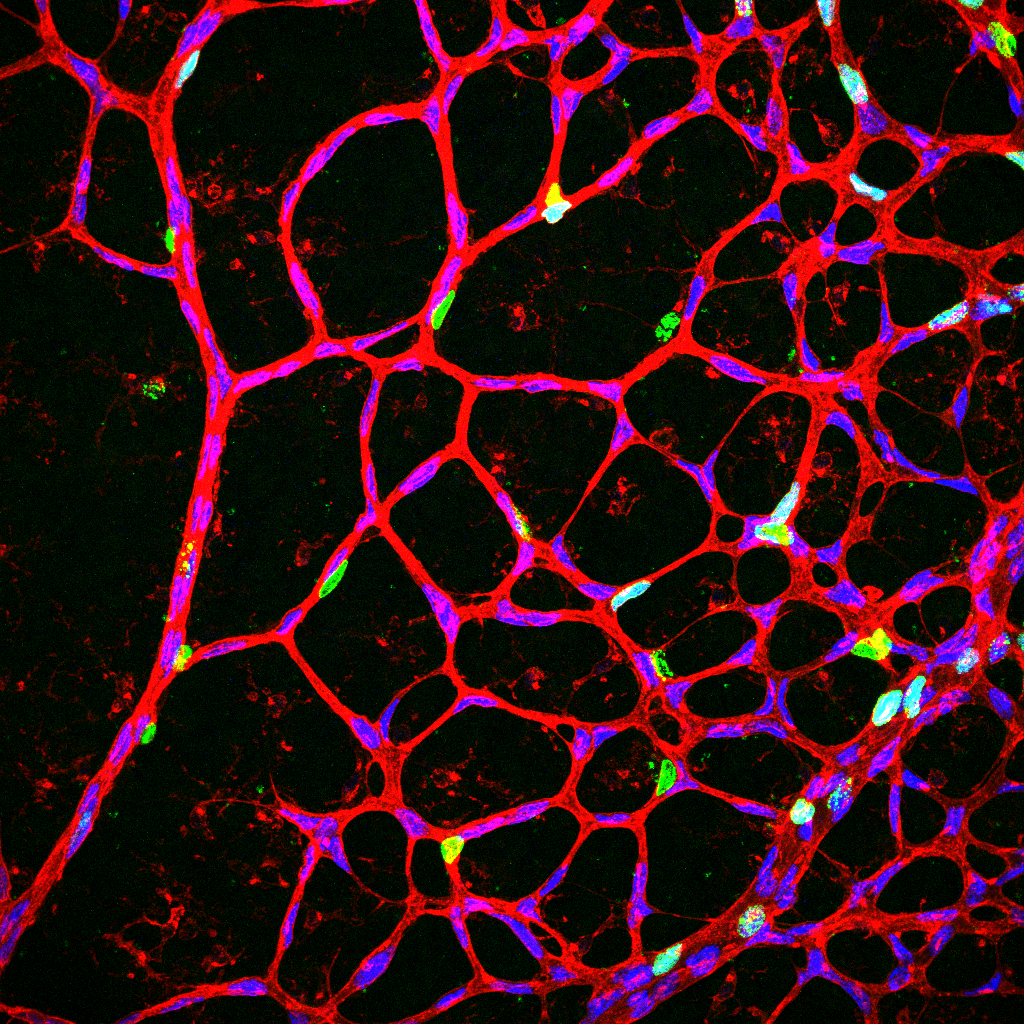

Supplement: Supplementary file 9 — Source Data for Figure 6 [file EMMM-14-e15619-s002.zip › EMM-2021-15619-V3-Figure_6_Source_Data/Fig. 6/6B/Control prevention vehicle 40X.tif]

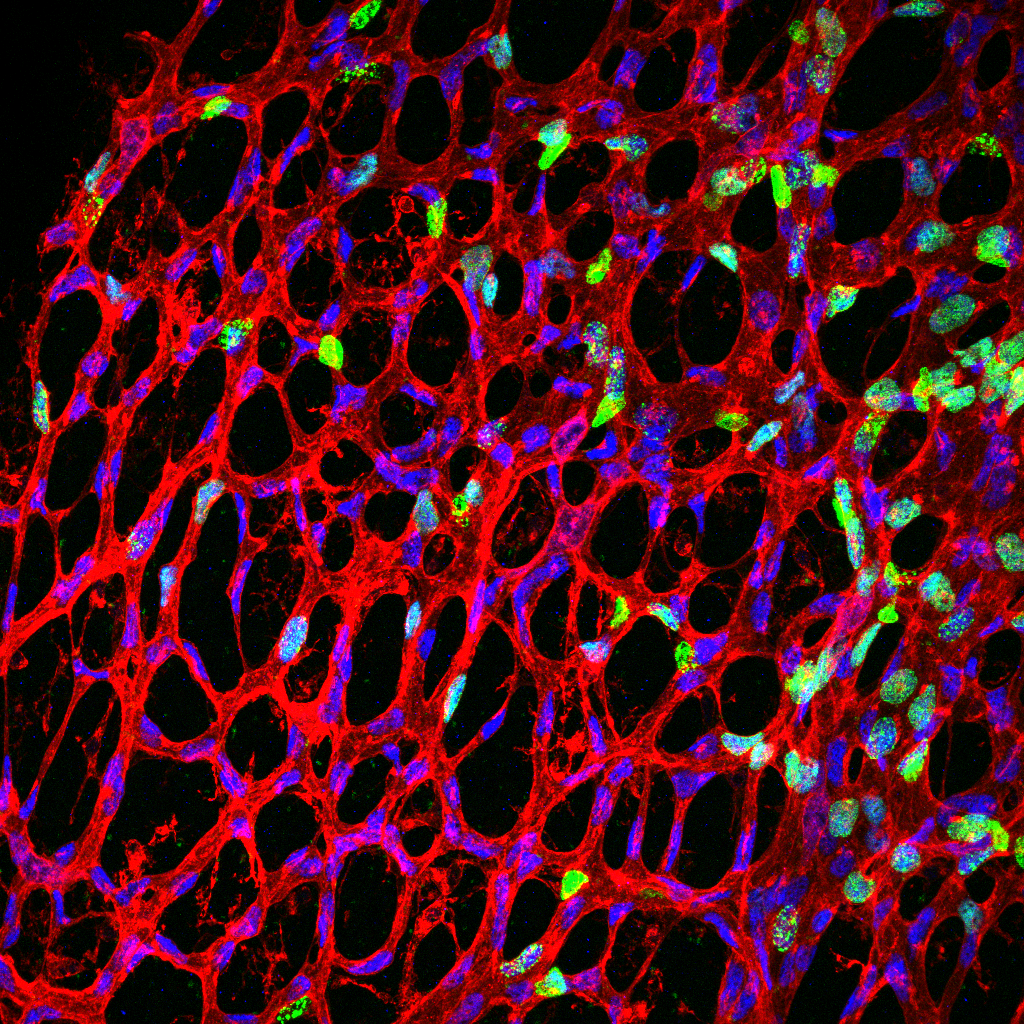

Supplement: Supplementary file 9 — Source Data for Figure 6 [file EMMM-14-e15619-s002.zip › EMM-2021-15619-V3-Figure_6_Source_Data/Fig. 6/6B/EC-Pik3caH1047R prevention vehicle 40X.tif]

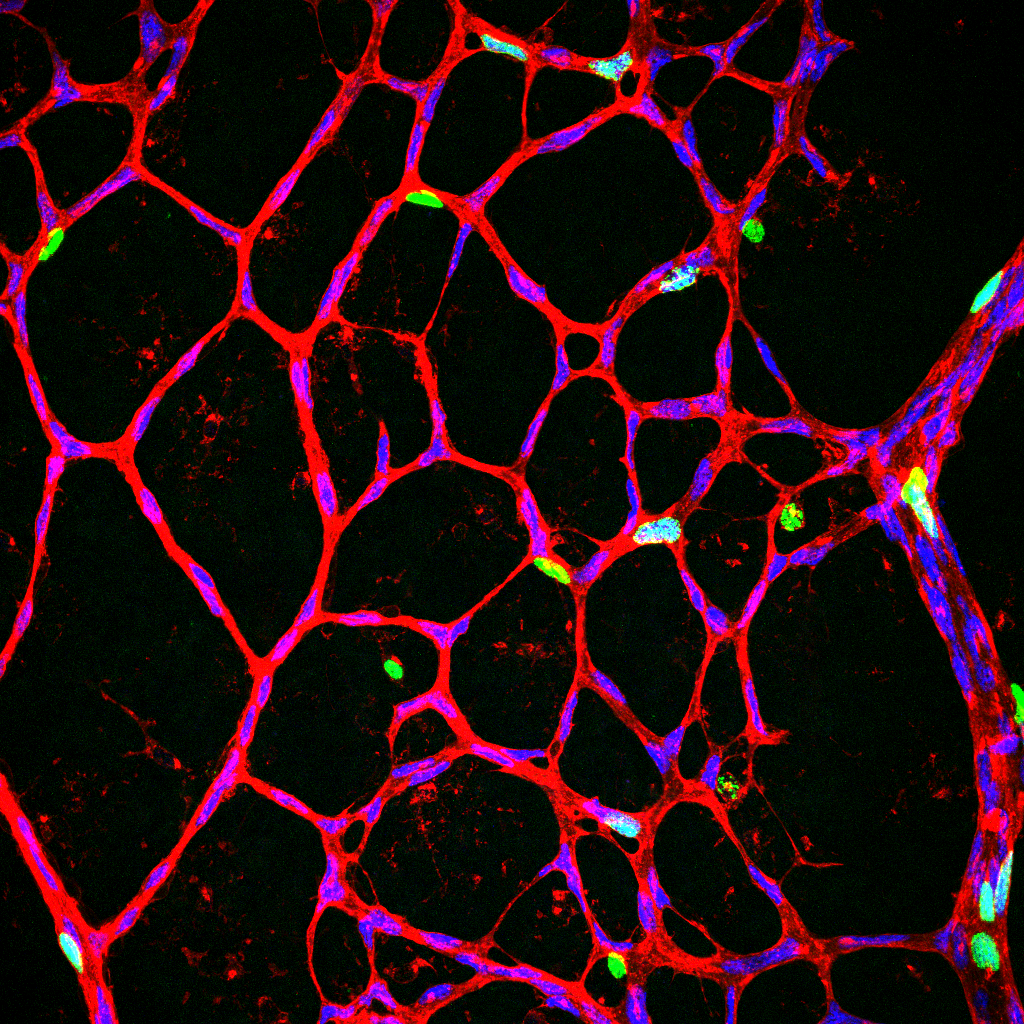

Supplement: Supplementary file 9 — Source Data for Figure 6 [file EMMM-14-e15619-s002.zip › EMM-2021-15619-V3-Figure_6_Source_Data/Fig. 6/6B/Control prevention 35 mg ARQ 092 40X.tif]

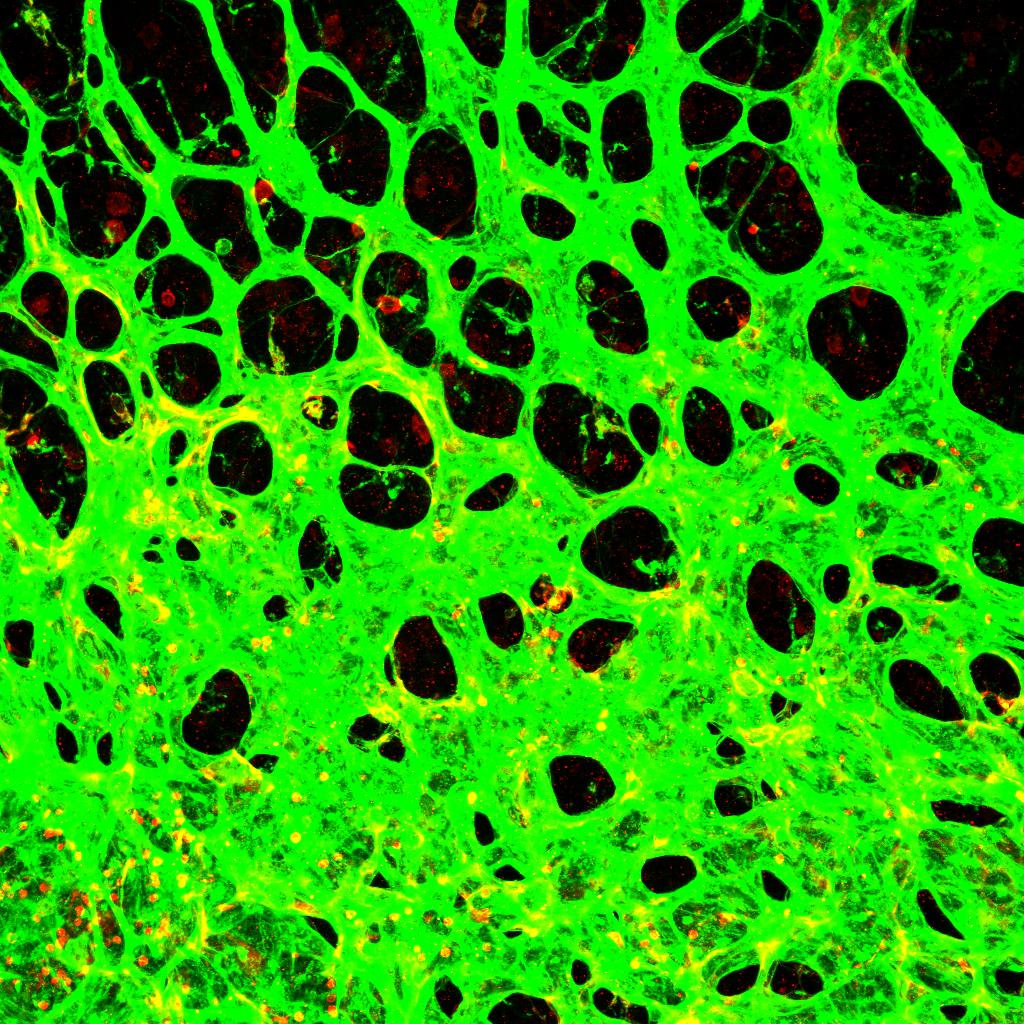

Supplement: Supplementary file 10 — Source Data for Figure 7 [file EMMM-14-e15619-s009.zip › EMM-2021-15619-V3-Figure_7_Source_Data/Fig. 7/7C/EC-Pik3caH1047R regression vehicle 40X.tif]

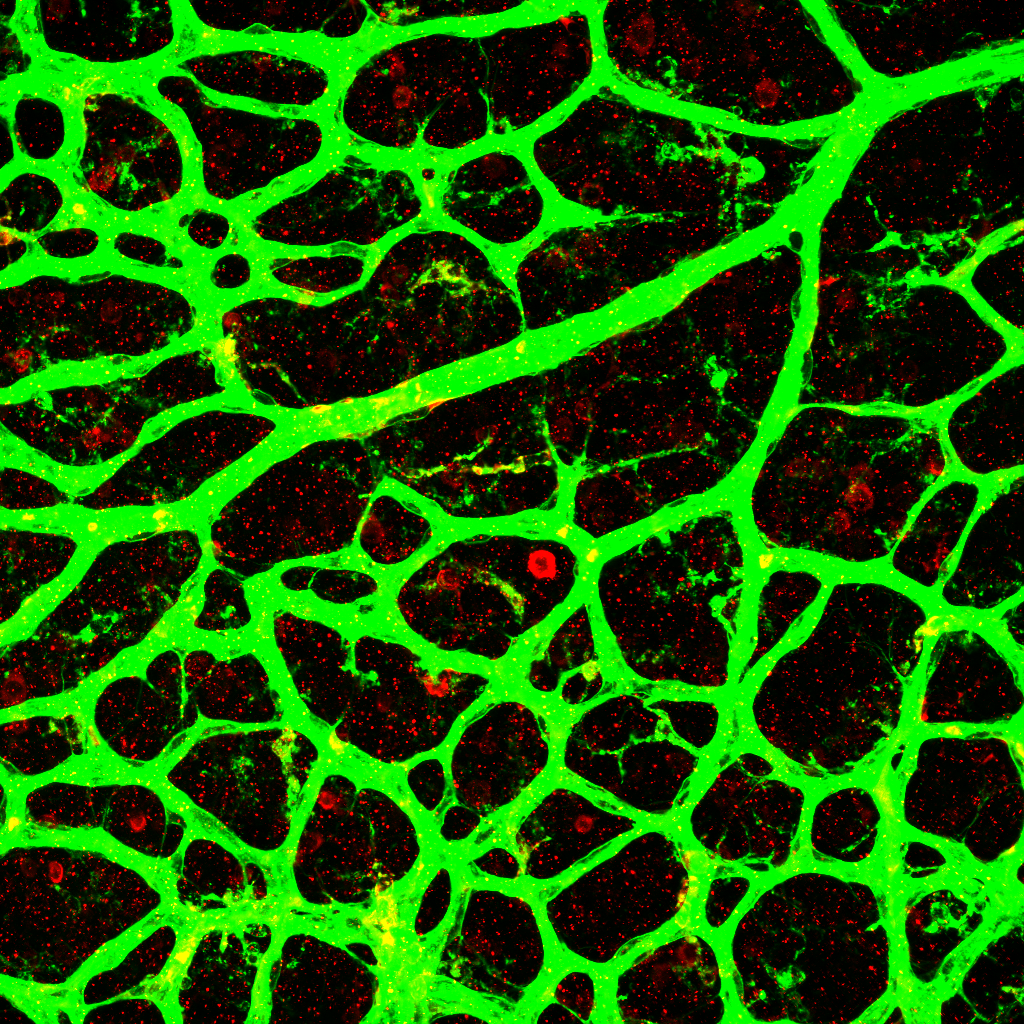

Supplement: Supplementary file 10 — Source Data for Figure 7 [file EMMM-14-e15619-s009.zip › EMM-2021-15619-V3-Figure_7_Source_Data/Fig. 7/7C/Control regression vehicle 40X.tif]

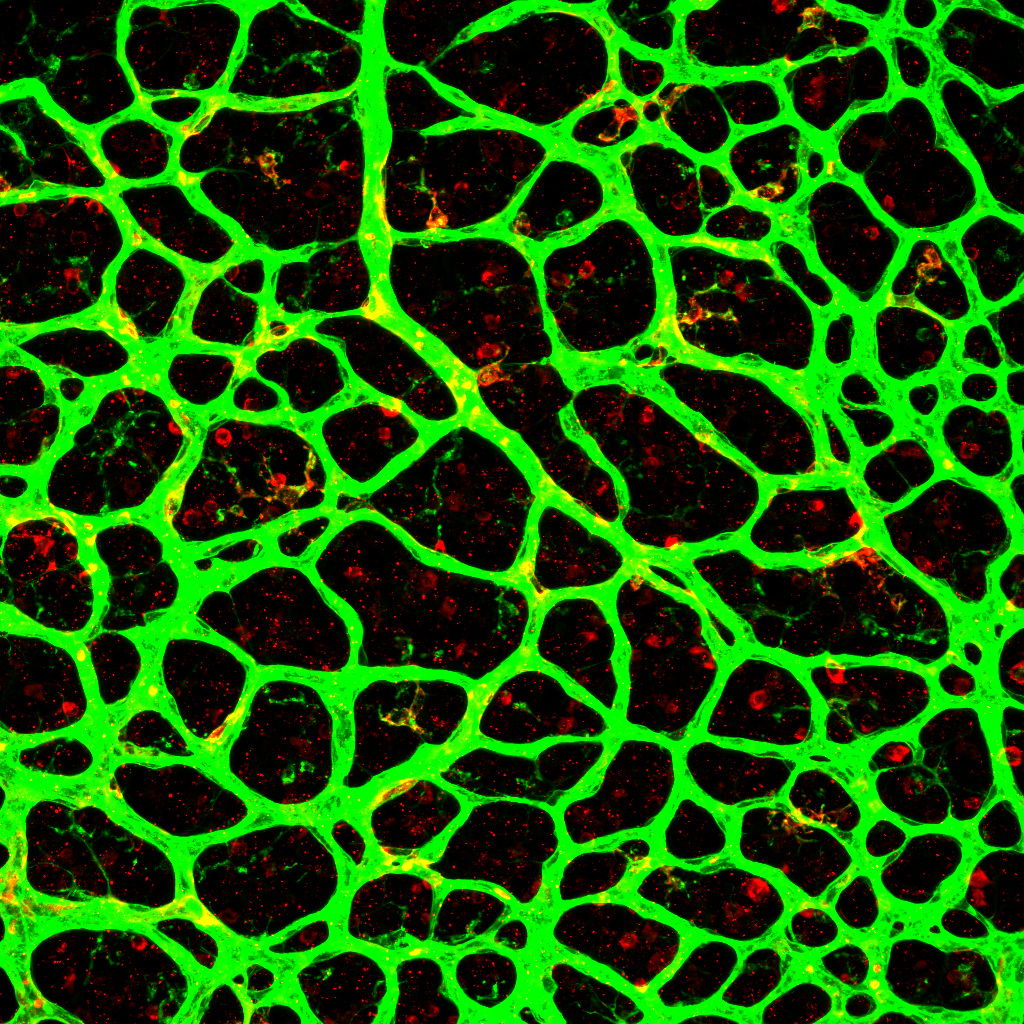

Supplement: Supplementary file 10 — Source Data for Figure 7 [file EMMM-14-e15619-s009.zip › EMM-2021-15619-V3-Figure_7_Source_Data/Fig. 7/7C/Control regression 35mg ARQ 092 40X.tif]

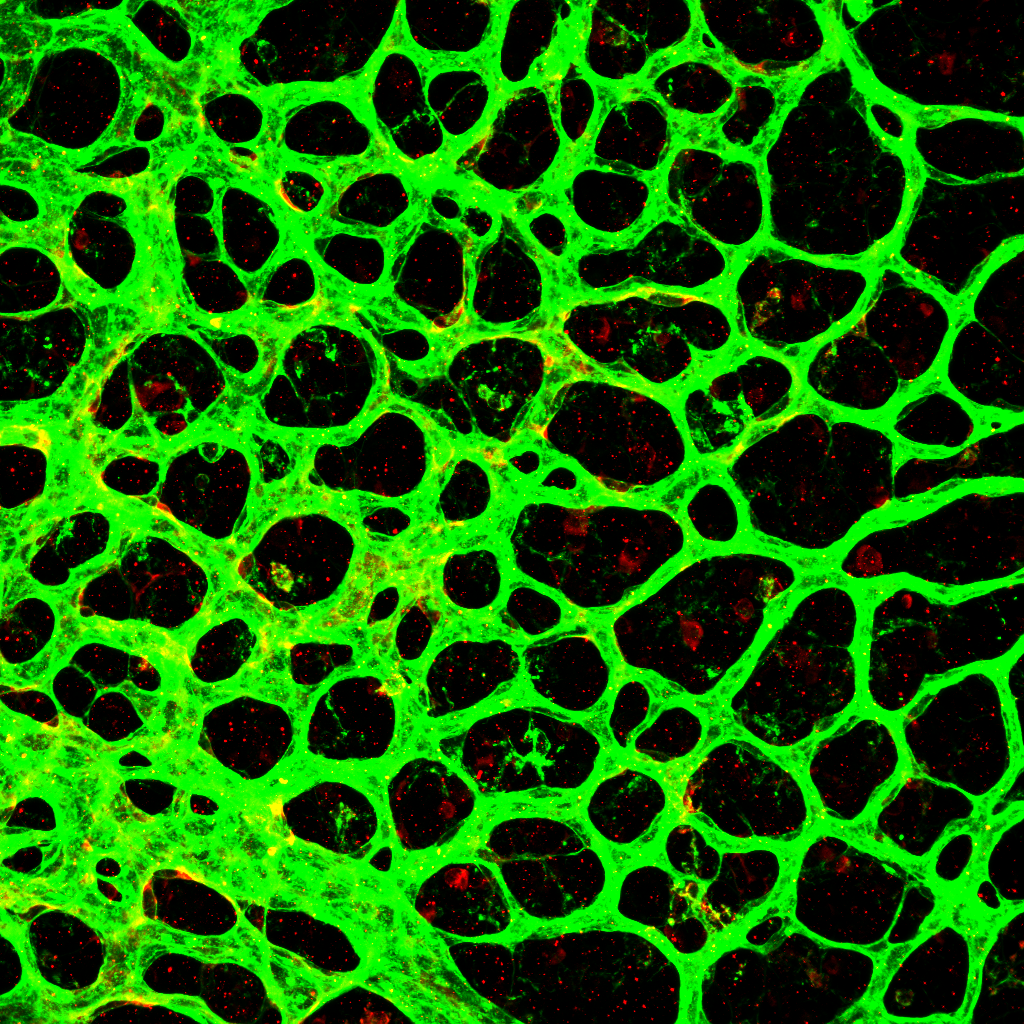

Supplement: Supplementary file 10 — Source Data for Figure 7 [file EMMM-14-e15619-s009.zip › EMM-2021-15619-V3-Figure_7_Source_Data/Fig. 7/7C/EC-Pik3caH1047R regression 35mg ARQ 092 40X.tif]

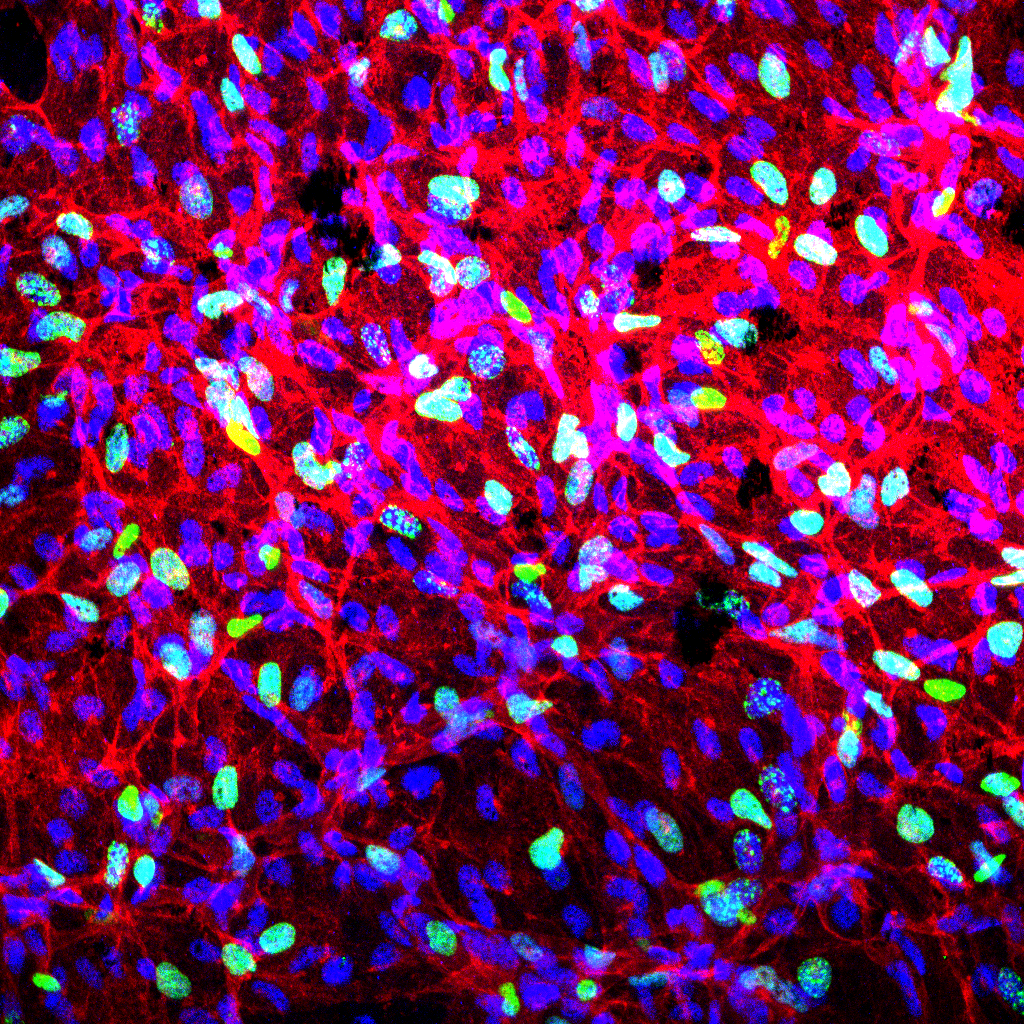

Supplement: Supplementary file 10 — Source Data for Figure 7 [file EMMM-14-e15619-s009.zip › EMM-2021-15619-V3-Figure_7_Source_Data/Fig. 7/7B/EC-Pik3caH1047R regression vehicle 40X.tif]

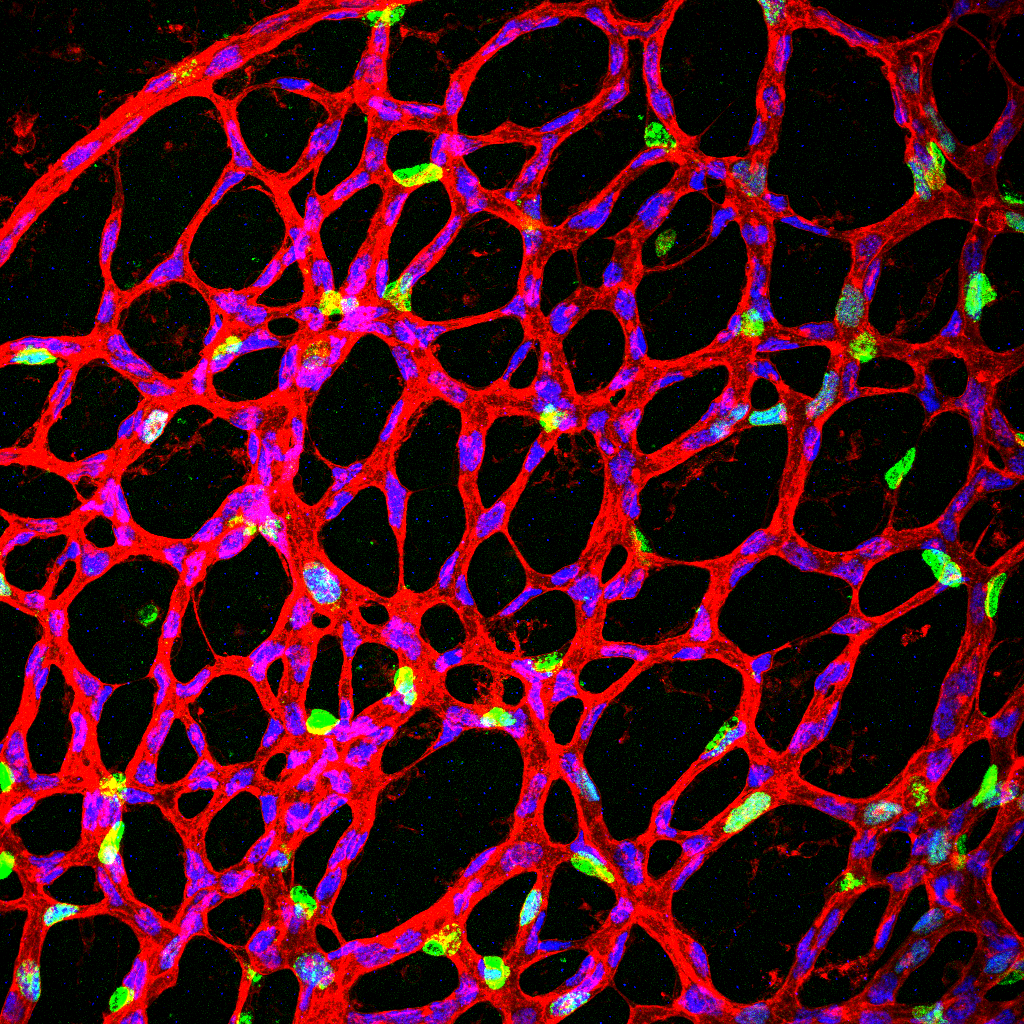

Supplement: Supplementary file 10 — Source Data for Figure 7 [file EMMM-14-e15619-s009.zip › EMM-2021-15619-V3-Figure_7_Source_Data/Fig. 7/7B/Control regression vehicle 40X.tif]

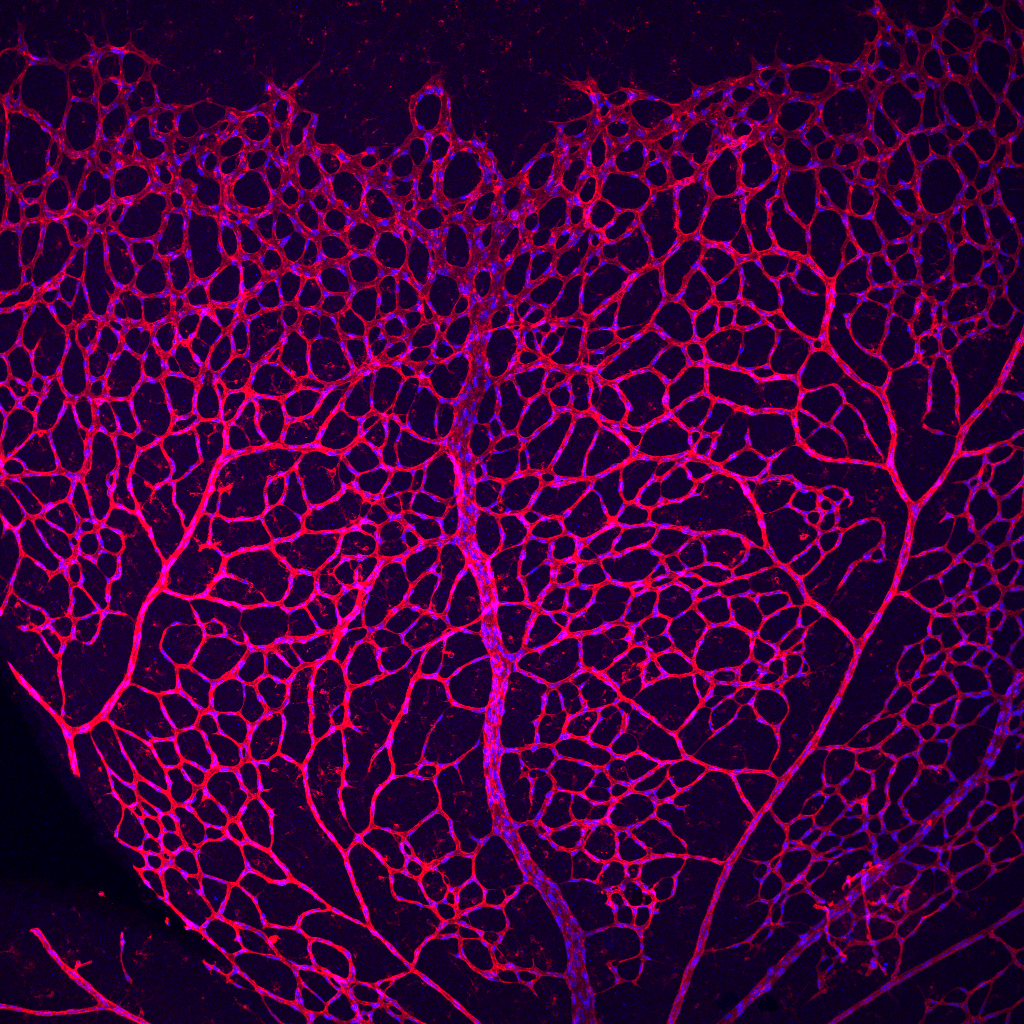

Supplement: Supplementary file 10 — Source Data for Figure 7 [file EMMM-14-e15619-s009.zip › EMM-2021-15619-V3-Figure_7_Source_Data/Fig. 7/7B/Control regression 35 mg ARQ 092 10X.tif]

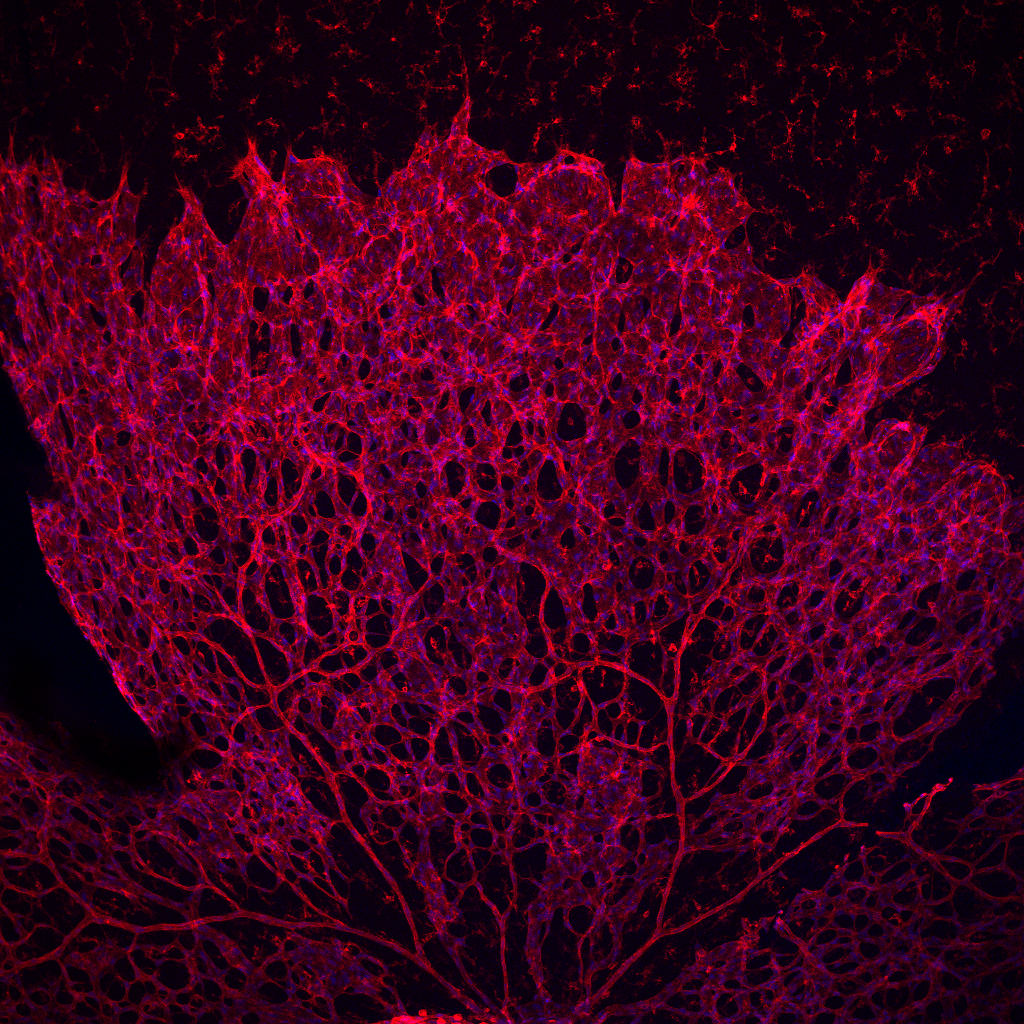

Supplement: Supplementary file 10 — Source Data for Figure 7 [file EMMM-14-e15619-s009.zip › EMM-2021-15619-V3-Figure_7_Source_Data/Fig. 7/7B/EC-Pik3caH1047R regression vehicle 10X.tif]

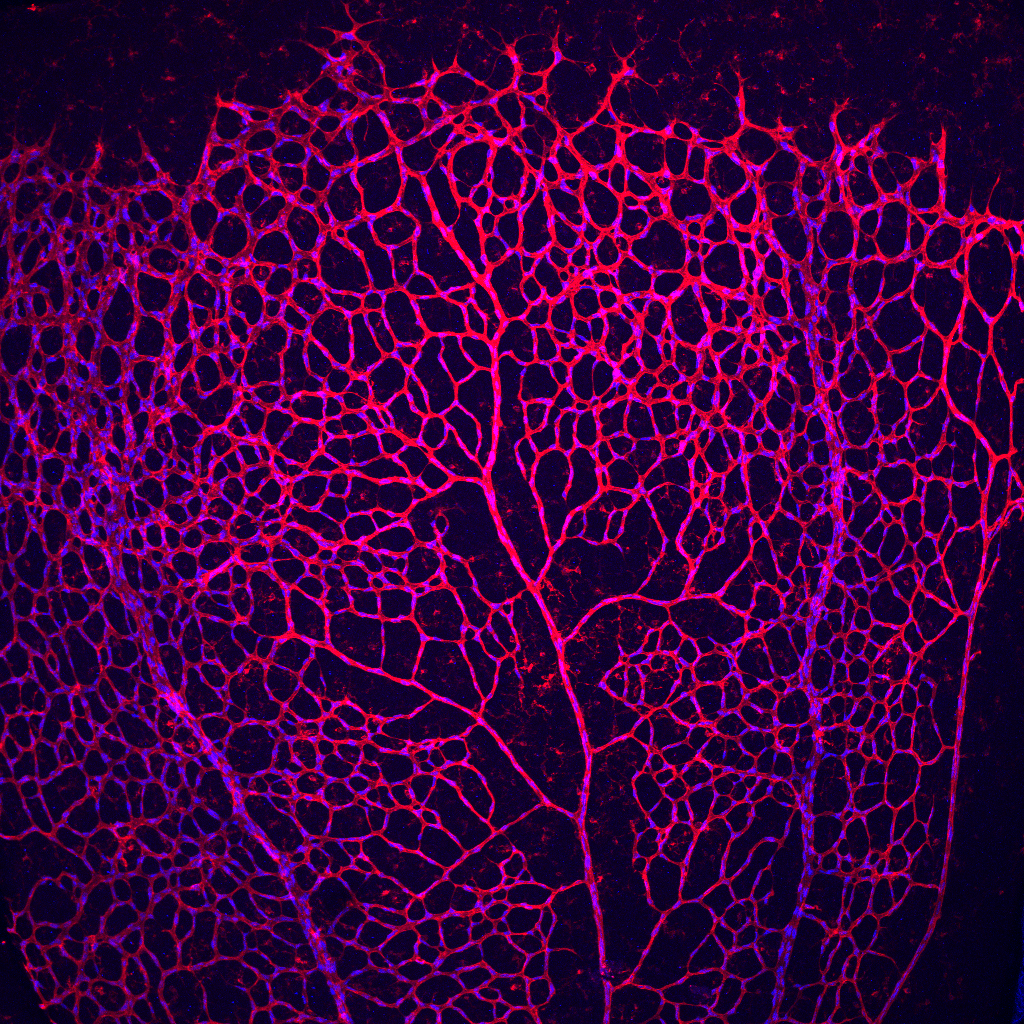

Supplement: Supplementary file 10 — Source Data for Figure 7 [file EMMM-14-e15619-s009.zip › EMM-2021-15619-V3-Figure_7_Source_Data/Fig. 7/7B/Control regression vehicle 10X.tif]

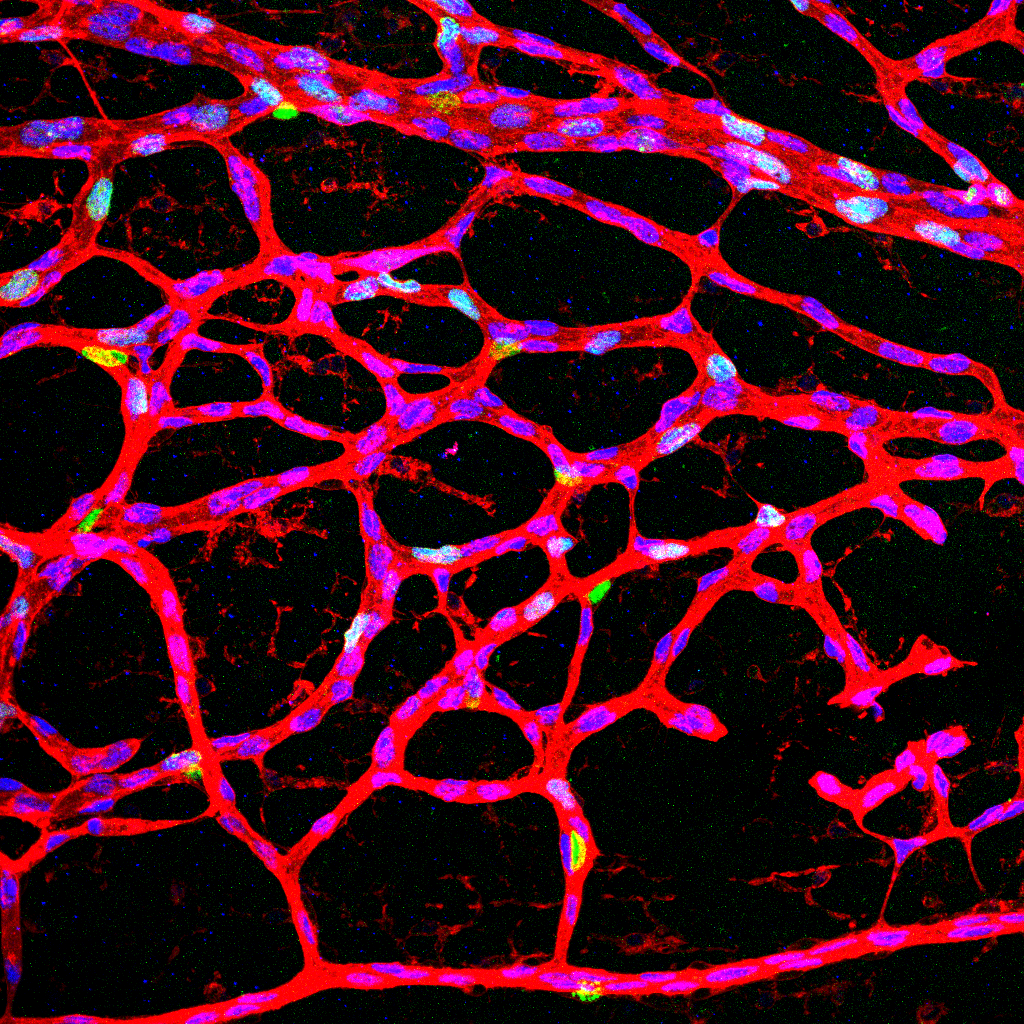

Supplement: Supplementary file 10 — Source Data for Figure 7 [file EMMM-14-e15619-s009.zip › EMM-2021-15619-V3-Figure_7_Source_Data/Fig. 7/7B/Control regression 35 mg ARQ 092 40X.tif]

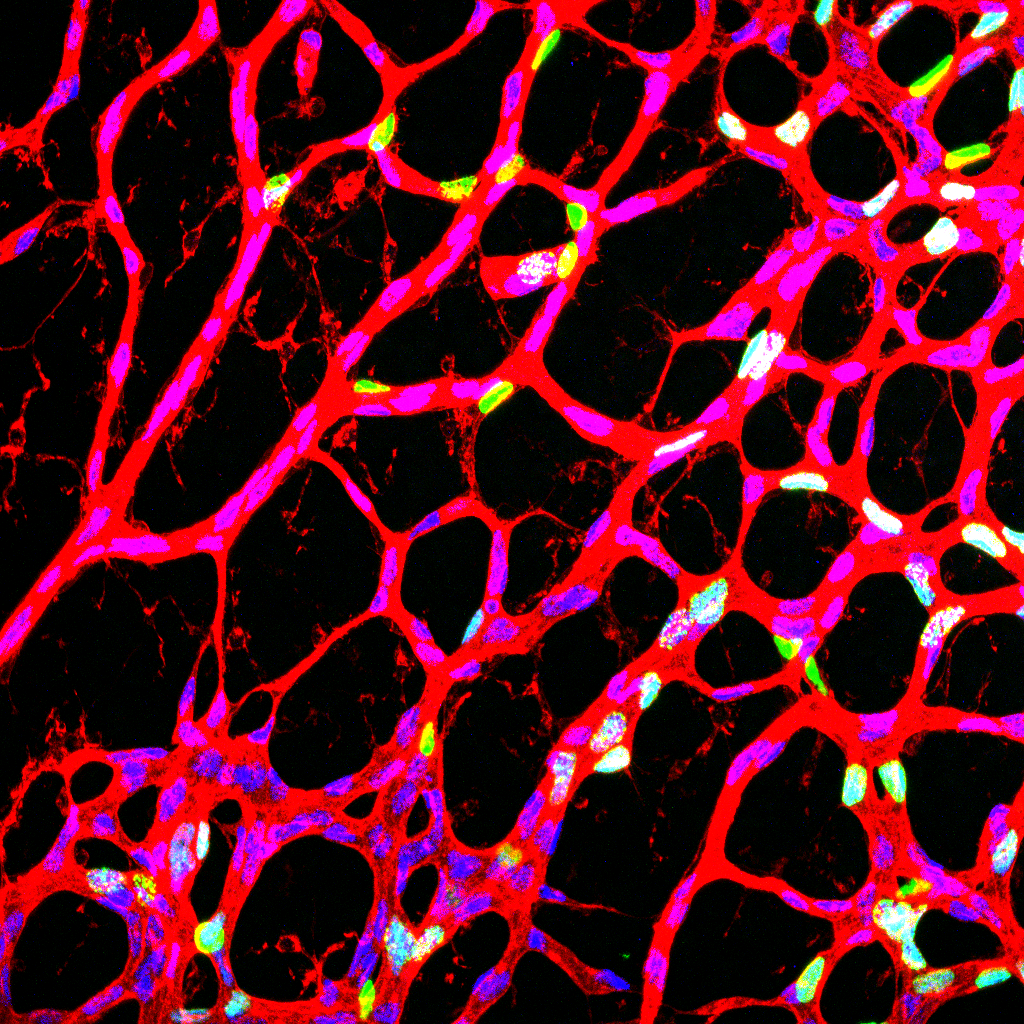

Supplement: Supplementary file 10 — Source Data for Figure 7 [file EMMM-14-e15619-s009.zip › EMM-2021-15619-V3-Figure_7_Source_Data/Fig. 7/7B/EC-Pik3caH1047R regression 35 mg ARQ 092 40X.tif]

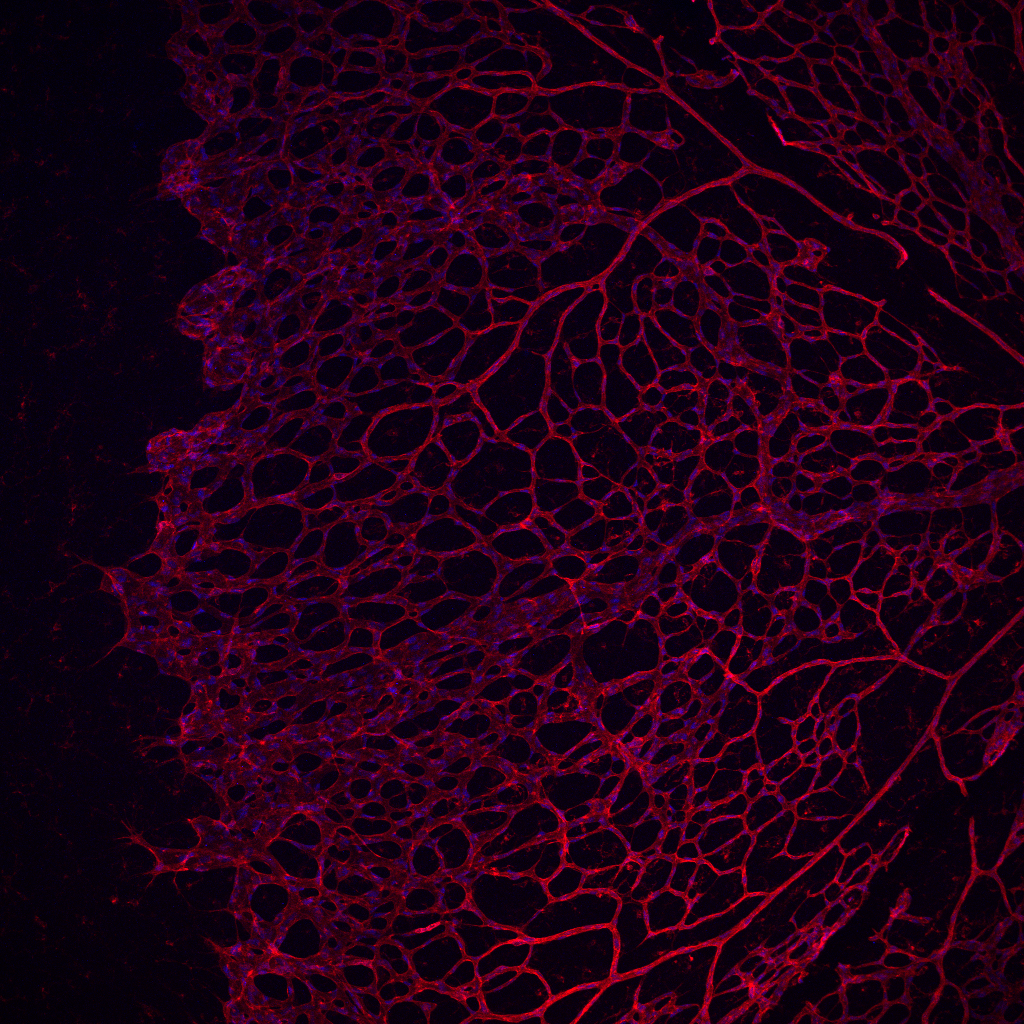

Supplement: Supplementary file 10 — Source Data for Figure 7 [file EMMM-14-e15619-s009.zip › EMM-2021-15619-V3-Figure_7_Source_Data/Fig. 7/7B/EC-Pik3caH1047R regression 35 mg ARQ 092 10X.tif]

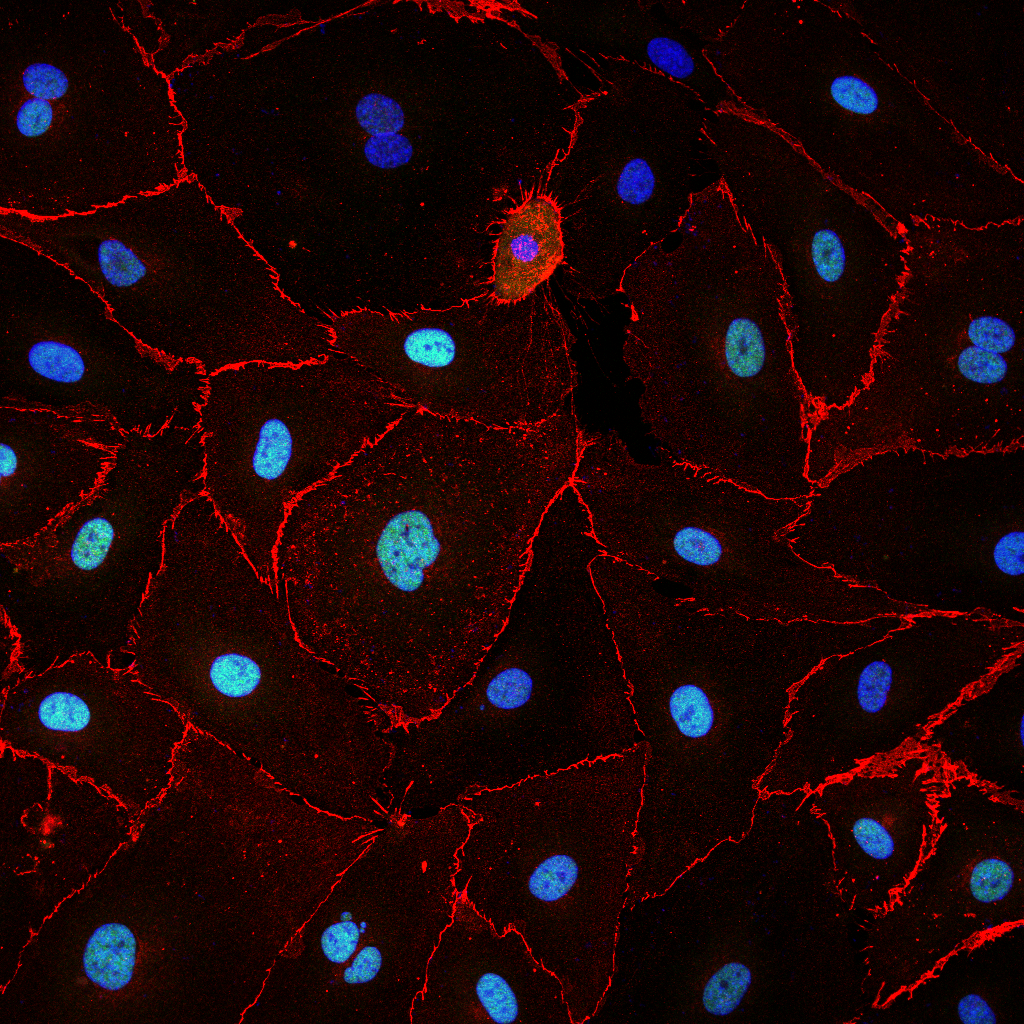

Supplement: Supplementary file 11 — Source Data for Figure 8 [file EMMM-14-e15619-s007.zip › EMM-2021-15619-V3-Figure_8_Source_Data/Fig. 8/8C/TEKL914F VM08.tif]

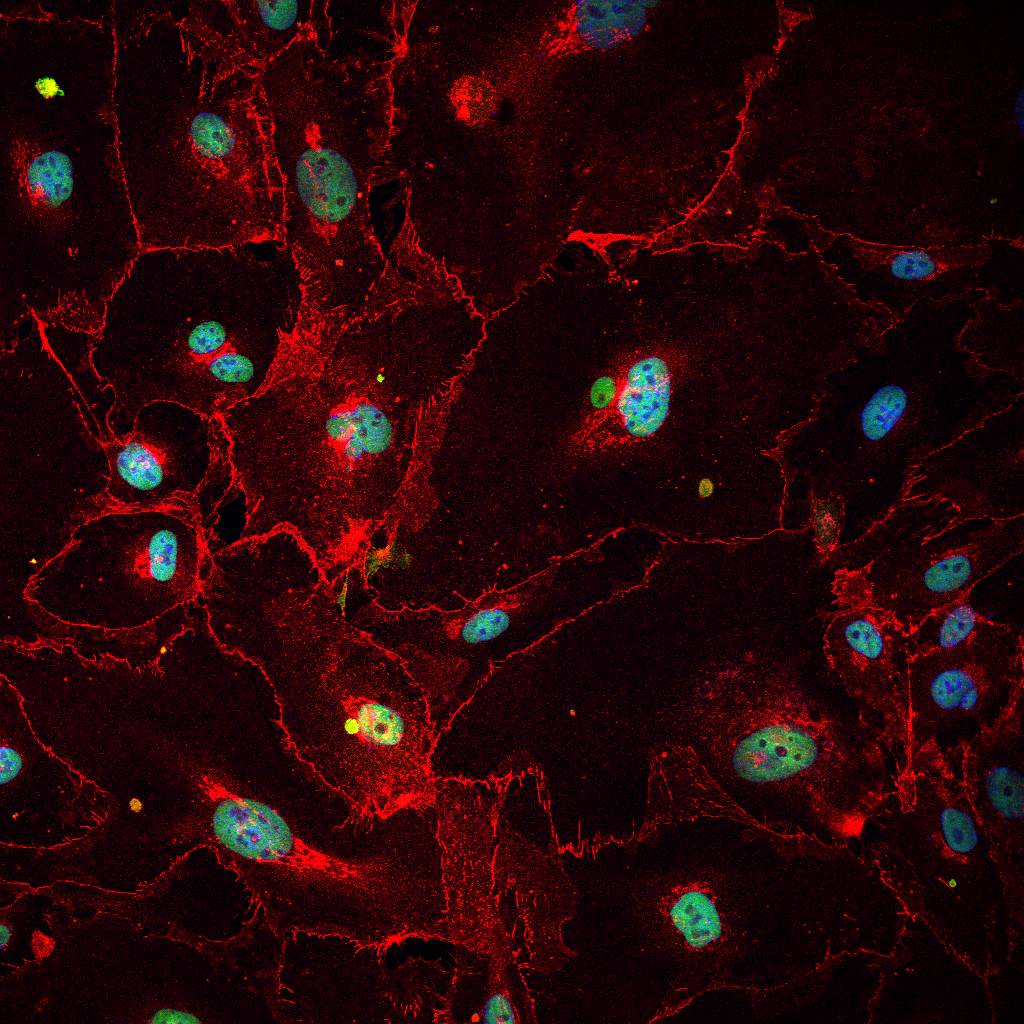

Supplement: Supplementary file 11 — Source Data for Figure 8 [file EMMM-14-e15619-s007.zip › EMM-2021-15619-V3-Figure_8_Source_Data/Fig. 8/8C/PIK3CAH1047R VM06.tif]

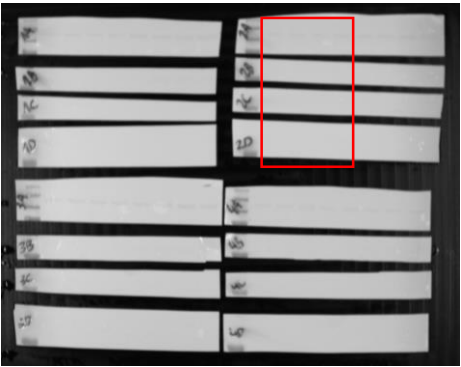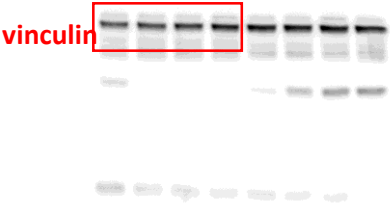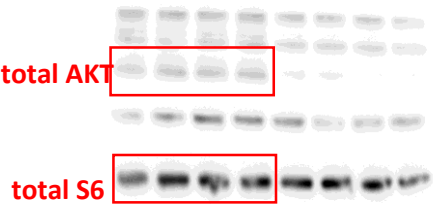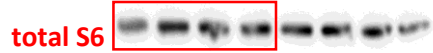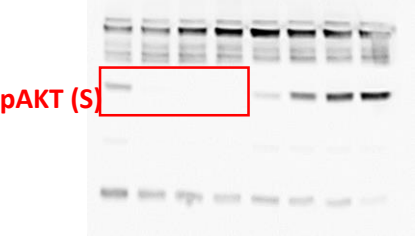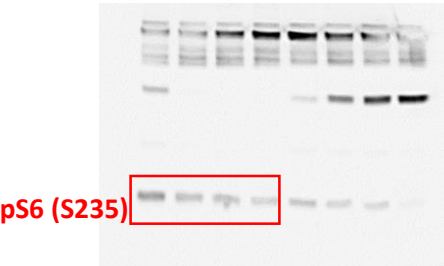

Supplement: Supplementary file 11 — Source Data for Figure 8 [file EMMM-14-e15619-s007.zip › EMM-2021-15619-V3-Figure_8_Source_Data/WB Fig 8F.pdf]

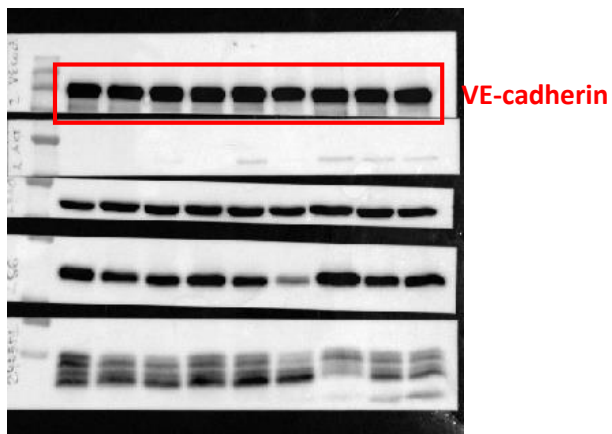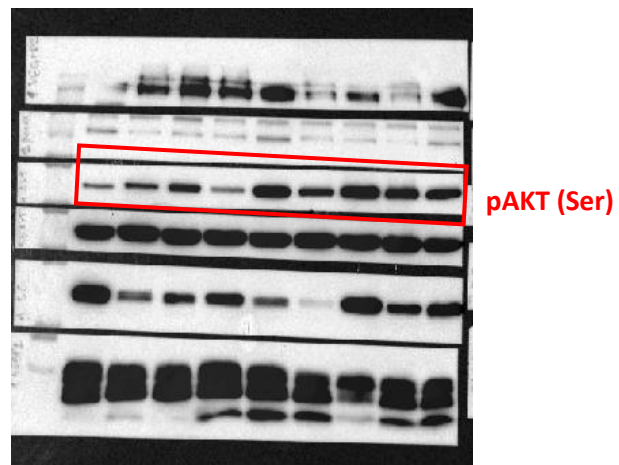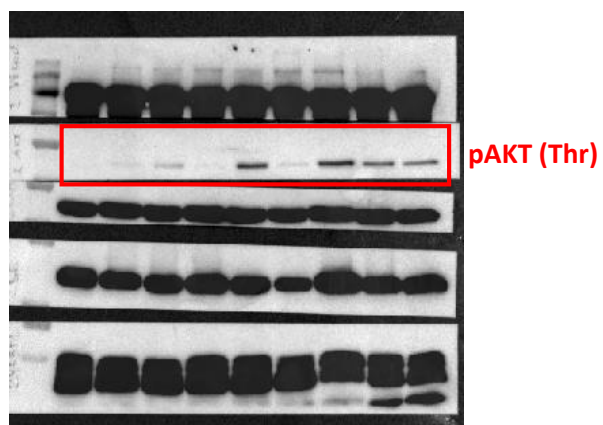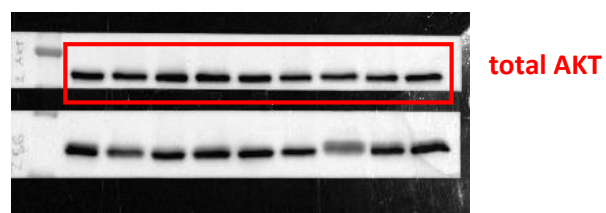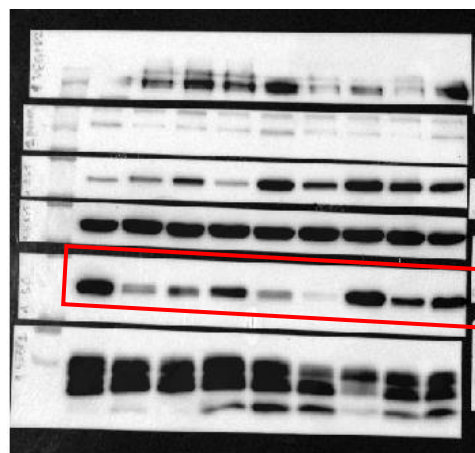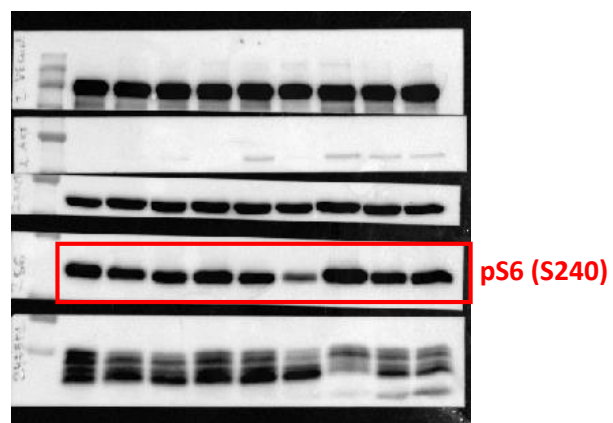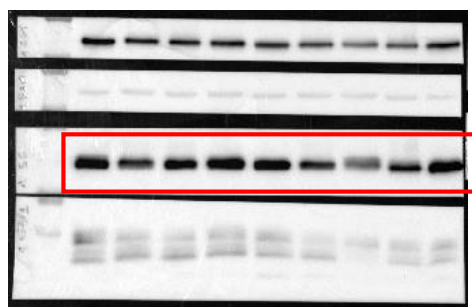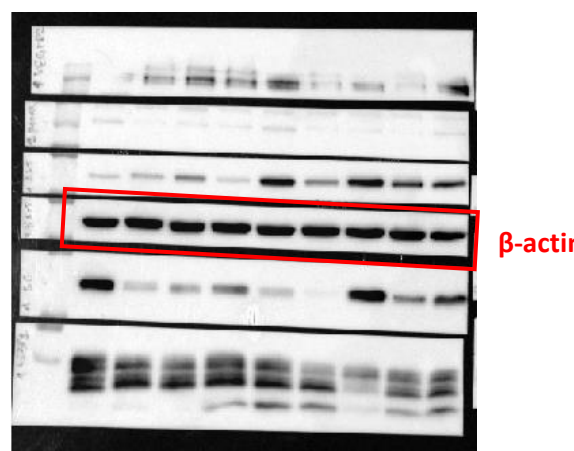

Supplement: Supplementary file 11 — Source Data for Figure 8 [file EMMM-14-e15619-s007.zip › EMM-2021-15619-V3-Figure_8_Source_Data/WB Fig 8D.pdf]

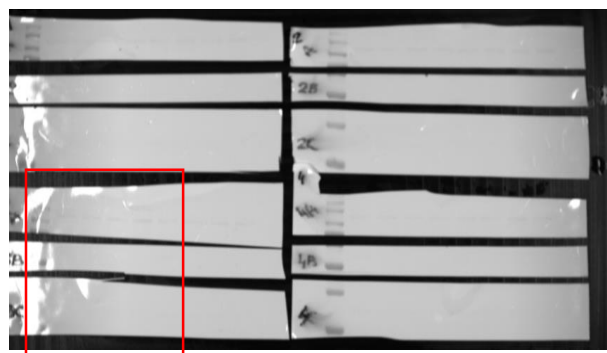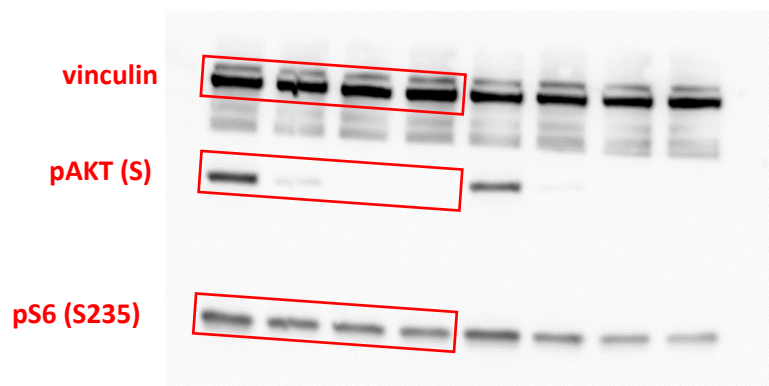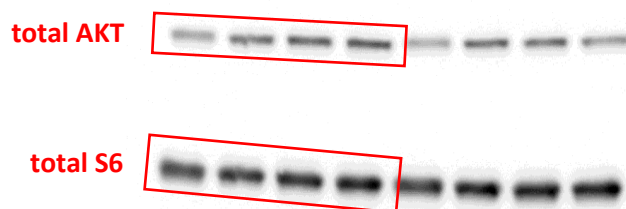

Supplement: Supplementary file 11 — Source Data for Figure 8 [file EMMM-14-e15619-s007.zip › EMM-2021-15619-V3-Figure_8_Source_Data/WB Fig 8E.pdf]

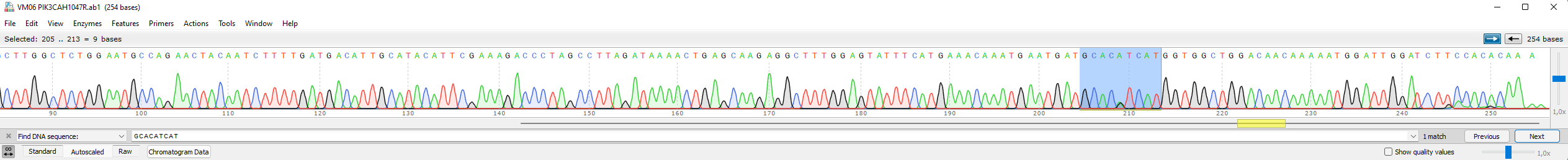

Supplement: Supplementary file 11 — Source Data for Figure 8 [file EMMM-14-e15619-s007.zip › EMM-2021-15619-V3-Figure_8_Source_Data/Fig 8B/VM06.tif]

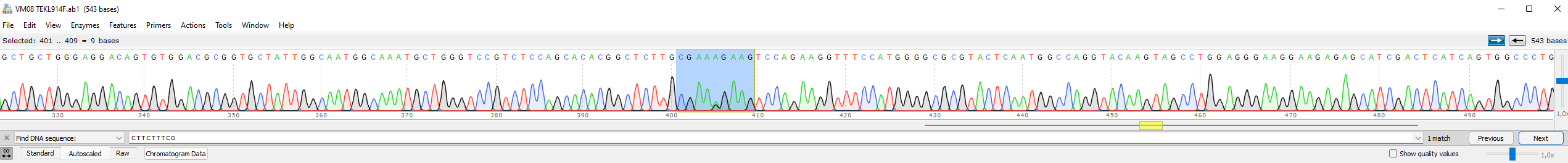

Supplement: Supplementary file 11 — Source Data for Figure 8 [file EMMM-14-e15619-s007.zip › EMM-2021-15619-V3-Figure_8_Source_Data/Fig 8B/VM08.tif]

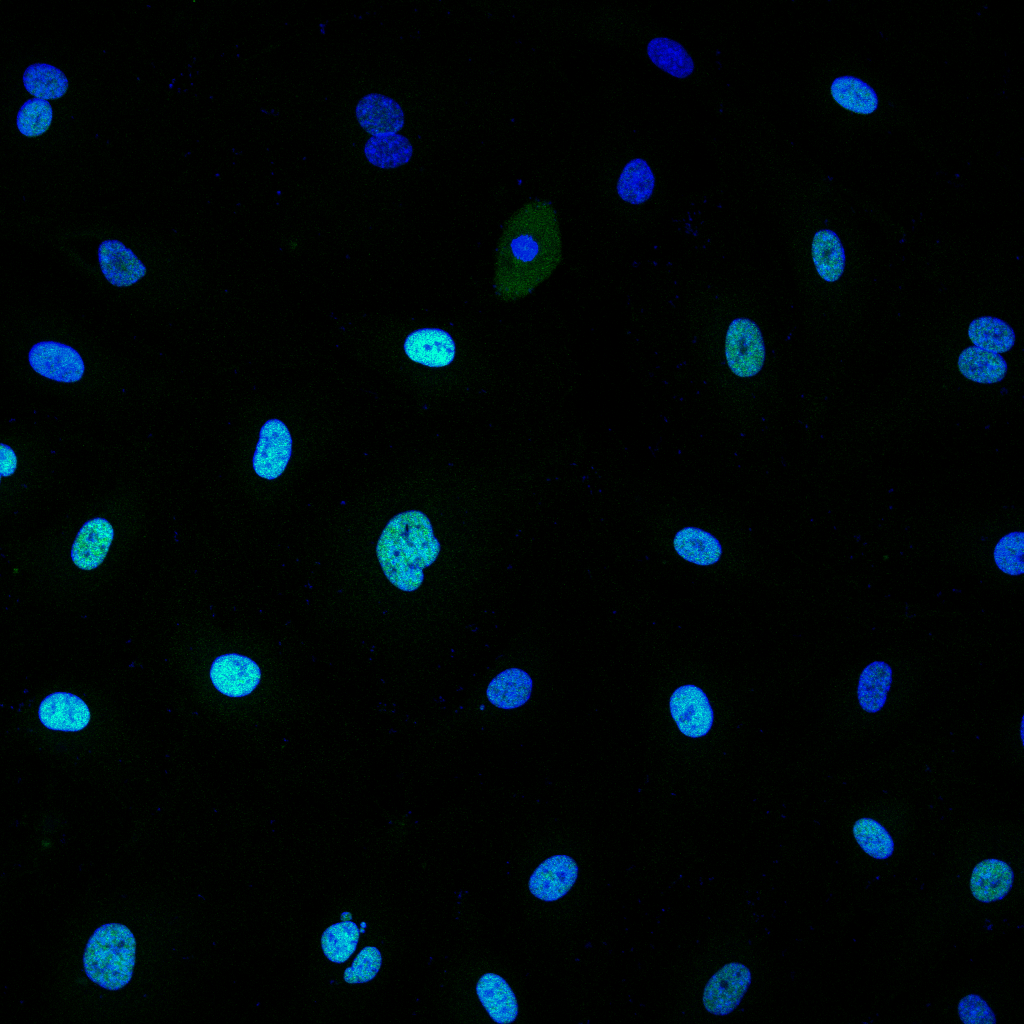

Supplement: Supplementary file 11 — Source Data for Figure 8 [file EMMM-14-e15619-s007.zip › EMM-2021-15619-V3-Figure_8_Source_Data/Fig 8C/TEKL914F VM08_ERG_DAPI.png]

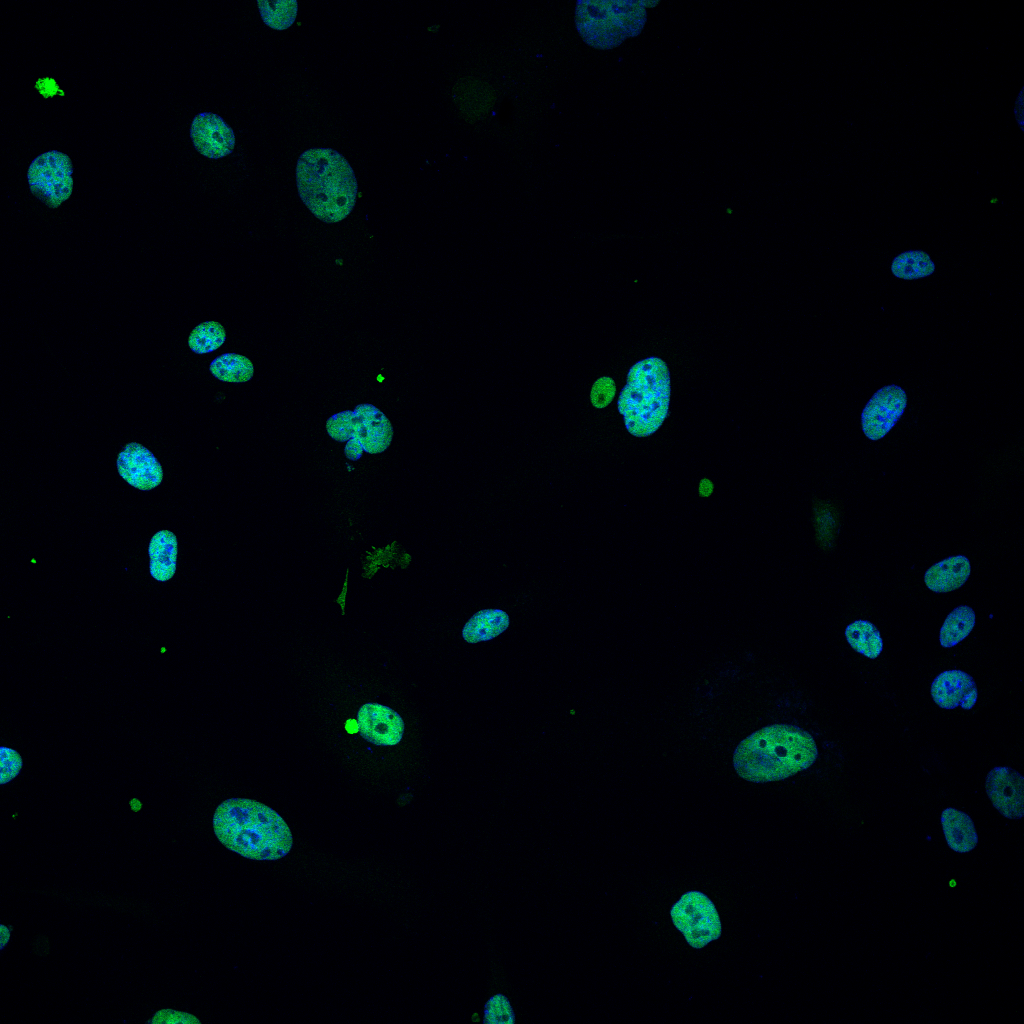

Supplement: Supplementary file 11 — Source Data for Figure 8 [file EMMM-14-e15619-s007.zip › EMM-2021-15619-V3-Figure_8_Source_Data/Fig 8C/PIK3CAH1047R VM06_ERG_DAPI.png]

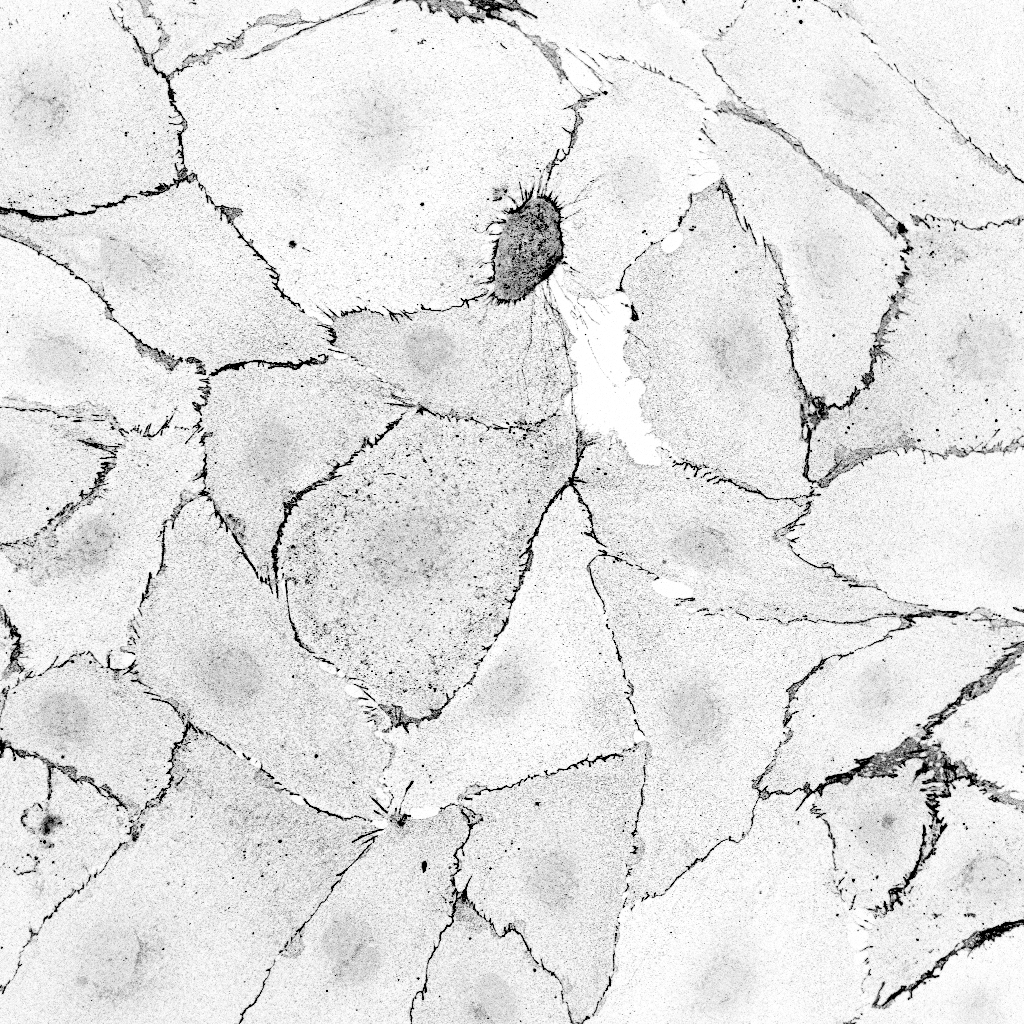

Supplement: Supplementary file 11 — Source Data for Figure 8 [file EMMM-14-e15619-s007.zip › EMM-2021-15619-V3-Figure_8_Source_Data/Fig 8C/TEKL914F VM08_VECADH.png]

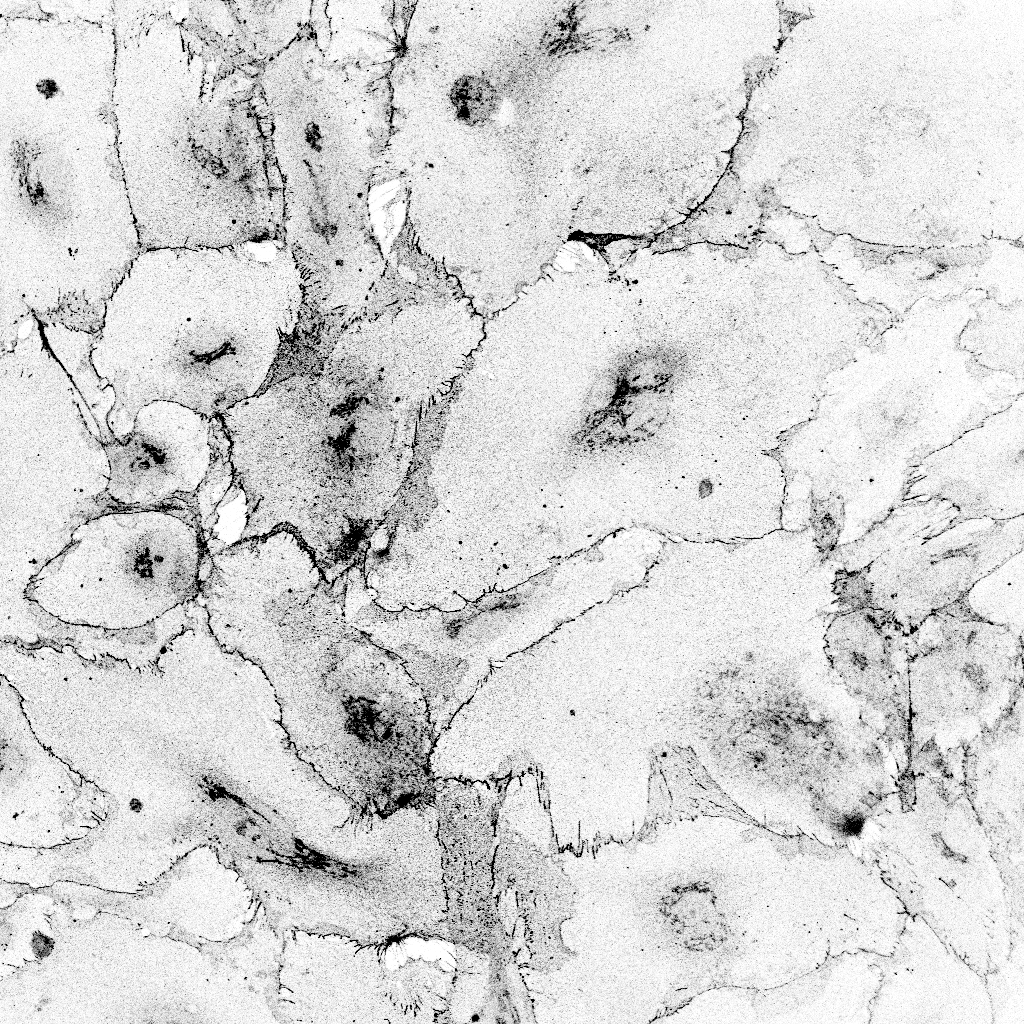

Supplement: Supplementary file 11 — Source Data for Figure 8 [file EMMM-14-e15619-s007.zip › EMM-2021-15619-V3-Figure_8_Source_Data/Fig 8C/PIK3CAH1047R VM06_VECADH.png]
