# Supplementary material for: The onset of PI3K‐related vascular malformations occurs during angiogenesis and is prevented by the AKT inhibitor miransertib
Source: EMBO Mol Med. 2022 Jun 13;14(7):e15619. doi: 10.15252/emmm.202115619 (PMC9260211; doi:10.15252/emmm.202115619)
Supplement: Supplementary file 12 [file EMMM-14-e15619-s008.pdf]

EMBO Molecular Medicine

The onset of PI3K-related vascular malformations occurs during angiogenesis

Piotr Kobialka, Helena Sabata, Odena Vilalta, Leonor Gouveia, Ana Angulo-Urarte, Laia Muixi, Jasmina Zanoncello, Oscar Muñoz-Aznar, Nagore Olaciregui, Lucia Fanlo, Anna Esteve-Codina, Cinzia Lavarino, Biola Javierre, Veronica Celis, Carlota Rovira, Susana López-Fernández, Eulalia Baselga, Jaume Mora, Sandra Castillo, and Mariona Graupera Garcia-Mila
DOI: 10.15252/emmm.202115619

Corresponding authors: Sandra Castillo (scastillo@carrerasresearch.org) , Mariona Graupera Garcia-Mila (mgraupera@carrerasresearch.org)

Review Timeline:

Submission Date:	4th Jan 22
Editorial Decision:	27th Jan 22
Revision Received:	21st Apr 22
Editorial Decision:	11th May 22
Revision Received:	19th May 22
Accepted:	20th May 22

Editor: Lise Roth

Transaction Report:

(Note: With the exception of the correction of typographical or spelling errors that could be a source of ambiguity, letters and reports are not edited. Depending on transfer agreements, referee reports obtained elsewhere may or may not be included in this compilation. Referee reports are anonymous unless the Referee chooses to sign their reports.)

27th Jan 2022

Dear Dr. Castillo,

Thank you for the submission of your manuscript to EMBO Molecular Medicine. We have now received feedback from the three reviewers who agreed to evaluate your manuscript. As you will see from the reports below, the referees acknowledge the interest of the study and are overall supporting publication of your work pending appropriate revisions.

However, while the reviewers acknowledge the interest of the study, they also raise a number of issues. In particular, referee #3 mentions two major concerns related to the models that does not recapitulate the patients' clinical manifestations, and to the treatment that might trigger serious adverse effects. As translational relevance is key for publication in EMBO Molecular Medicine, we discussed further these two points with the referees.
We concluded that even though the model does not fully recapitulate patients' phenotype, it still has translational value, particularly considering the consistent phenotype developed over a short-time frame. Therefore, in a revised version of the manuscript, we would like you to clearly discuss the limitations of the model.
The referees also agreed that evaluating the effects of miransertib on wild-type endothelial cells would answer their concern on potential side-effects of the drug.

Addressing these points and the other the reviewers' concerns in full will be necessary for further considering the manuscript in our journal, and acceptance of the manuscript will entail a second round of review. EMBO Molecular Medicine encourages a single round of revision only and therefore, acceptance or rejection of the manuscript will depend on the completeness of your responses included in the next, final version of the manuscript. For this reason, and to save you from any frustrations in the end, I would strongly advise against returning an incomplete revision.

Revised manuscripts should be submitted within three months of a request for revision; they will otherwise be treated as new submissions, except under exceptional circumstances in which a short extension is obtained from the editor.

When submitting your revised manuscript, please carefully review the instructions that follow below. We perform an initial quality control of all revised manuscripts before re-review; failure to include requested items will delay the evaluation of your revision.

We require:

1) A .docx formatted version of the manuscript text (including legends for main figures, EV figures and tables). Please make sure that the changes are highlighted to be clearly visible.

2) Individual production quality figure files as .eps, .tif, .jpg (one file per figure). For guidance, download the 'Figure Guide PDF' (https://www.embopress.org/page/journal/17574684/authorguide#figureformat).

3) A .docx formatted letter INCLUDING the reviewers' reports and your detailed point-by-point responses to their comments. As part of the EMBO Press transparent editorial process, the point-by-point response is part of the Review Process File (RPF), which will be published alongside your paper.

4) A complete author checklist, which you can download from our author guidelines (https://www.embopress.org/page/journal/17574684/authorguide#submissionofrevisions). Please insert information in the checklist that is also reflected in the manuscript. The completed author checklist will also be part of the RPF.

5) Please note that all corresponding authors are required to supply an ORCID ID for their name upon submission of a revised manuscript.

6) It is mandatory to include a 'Data Availability' section after the Materials and Methods. Before submitting your revision, primary datasets produced in this study need to be deposited in an appropriate public database, and the accession numbers and database listed under 'Data Availability'. Please remember to provide a reviewer password if the datasets are not yet public (see https://www.embopress.org/page/journal/17574684/authorguide#dataavailability).
In case you have no data that requires deposition in a public database, please state so in this section. Note that the Data Availability Section is restricted to new primary data that are part of this study.

7) For data quantification: please specify the name of the statistical test used to generate error bars and P values, the number (n) of independent experiments (specify technical or biological replicates) underlying each data point and the test used to calculate p-values in each figure legend. The figure legends should contain a basic description of n, P and the test applied. Graphs must include a description of the bars and the error bars (s.d., s.e.m.). Exact p values (not a range) are required.

8) We would also encourage you to include the source data for figure panels that show essential data. Numerical data should be provided as individual .xls or .csv files (including a tab describing the data). For blots or microscopy, uncropped images should be submitted (using a zip archive if multiple images need to be supplied for one panel). Additional information on source data and instruction on how to label the files are available at
.

9) Our journal encourages inclusion of *data citations in the reference list* to directly cite datasets that were re-used and obtained from public databases. Data citations in the article text are distinct from normal bibliographical citations and should directly link to the database records from which the data can be accessed. In the main text, data citations are formatted as follows: "Data ref: Smith et al, 2001" or "Data ref: NCBI Sequence Read Archive PRJNA342805, 2017". In the Reference list, data citations must be labeled with "[DATASET]". A data reference must provide the database name, accession number/identifiers and a resolvable link to the landing page from which the data can be accessed at the end of the reference. Further instructions are available at .

10) We replaced Supplementary Information with Expanded View (EV) Figures and Tables that are collapsible/expandable online. A maximum of 5 EV Figures can be typeset. EV Figures should be cited as 'Figure EV1, Figure EV2" etc... in the text and their respective legends should be included in the main text after the legends of regular figures.
- For the figures that you do NOT wish to display as Expanded View figures, they should be bundled together with their legends in a single PDF file called *Appendix*, which should start with a short Table of Content. Appendix figures should be referred to in the main text as: "Appendix Figure S1, Appendix Figure S2" etc.
- Additional Tables/Datasets should be labeled and referred to as Table EV1, Dataset EV1, etc. Legends have to be provided in a separate tab in case of .xls files. Alternatively, the legend can be supplied as a separate text file (README) and zipped together with the Table/Dataset file.
See detailed instructions here:
.

11) Conflict of interest: We updated our journal's competing interests policy in January 2022 and request authors to consider both actual and perceived competing interests. Please review the policy https://www.embopress.org/competing-interests and update your competing interests if necessary.

12) Every published paper now includes a 'Synopsis' to further enhance discoverability. Synopses are displayed on the journal webpage and are freely accessible to all readers. They include a short stand first (maximum of 300 characters, including space) as well as 2-5 one-sentences bullet points that summarizes the paper. Please write the bullet points to summarize the key NEW findings. They should be designed to be complementary to the abstract - i.e. not repeat the same text. We encourage inclusion of key acronyms and quantitative information (maximum of 30 words / bullet point). Please use the passive voice. Please attach these in a separate file or send them by email, we will incorporate them accordingly.
Please also suggest a striking image or visual abstract to illustrate your article as a PNG file 550 px wide x 300-600 px high.

13) As part of the EMBO Publications transparent editorial process initiative (see our Editorial at http://embomolmed.embopress.org/content/2/9/329), EMBO Molecular Medicine will publish online a Review Process File (RPF) to accompany accepted manuscripts.
In the event of acceptance, this file will be published in conjunction with your paper and will include the anonymous referee reports, your point-by-point response and all pertinent correspondence relating to the manuscript. Let us know whether you agree with the publication of the RPF and as here, if you want to remove or not any figures from it prior to publication.
Please note that the Authors checklist will be published at the end of the RPF.

EMBO Molecular Medicine has a "scooping protection" policy, whereby similar findings that are published by others during review or revision are not a criterion for rejection. Should you decide to submit a revised version, I do ask that you get in touch after three months if you have not completed it, to update us on the status.

I look forward to receiving your revised manuscript.

With kind regards,

Lise Roth

Lise Roth, PhD
Editor
EMBO Molecular Medicine

***** Reviewer's comments *****

Referee #1 (Comments on Novelty/Model System for Author):

The study described in this manuscript was well designed and executed. The results were presented in a clear and concise manner with both qualitative and quantitative analyses reported. The conclusions drawn were valid. The model system described recapitulates the vascular malformations seen in human disease with mosaic expression of PIK3CA and TEK pathogenic variants. Developing models for mosaic diseases is extremely challenging and often lead to heterogeneous phenotypes. This system has a consistent phenotype that develops over a short time frame and can be easily assessed, making it very attractive for use in testing therapeutics.

Referee #1 (Remarks for Author):

The paper by Kobialka, et. al. entitled "The onset of PI3K-related vascular malformations occurs during angiogenesis and is prevented by the AKT inhibitor miransertib", uses both cell culture and an in vivo retinal model to investigate the role of PIK3CAH1047R in vascular development and the efficacy of the AKT inhibitor, miransertib, for ameliorating the vascular malformations associated with this variant. The authors showed that murine vascular endothelial cells expressing Pik3caH1047R had enhanced cell cycle progression compared to wild type cells in the presence of serum. Using the murine retinal model, they showed that activation of Pik3caH1047R early in vascular development led to more extensive vascular malformations than when the variant was activated later in the remodeling process and concluded that active angiogenesis is necessary for malformations to occur. Next, they demonstrated that mosaic expression of Pik3caH1047R with low numbers of variant-positive cells was sufficient to develop malformations and showed that the resulting malformations demonstrated several characteristics of human low-flow vascular malformations. They then showed that both high and low doses of miransertib could reduce formation of vascular malformations when administered early, and cause regression of malformations when administered later in retinal vascular development. Finally, the authors showed that miransertib could reduce PI3K/AKT signaling and viability in endothelial cells isolated from human vascular malformations with both PIK3CA and TEK mutations. The authors conclude that Pik3caH1047R murine retinal model is a robust system for testing efficacy of treatment strategies for low-flow vascular malformations and that miransertib is a good candidate for future clinical trials in patients with PIK3CA or TEK vascular malformations.
This is a very well-designed and executed study. The experiments were clearly described and conclusions valid. The development of this model system will be extremely useful not only for assessing therapeutic strategies for PI3K-driven vascular malformations, but may also be an important tool for studying the mechanism by which Pik3caH1047R causes these malformations to develop. I have only a few minor comments for the authors.

1. In lines 183-184, the authors state that with low dose 4-OHT activation, Pik3caH1047R only targeted veins and capillaries. How were the vessels identified? Was it just by morphology or were specific markers used to distinguish vessel types?
2. In figure 8G, the authors showed decreased viability with increasing concentration of miransertib in human cell lines with a PIK3CA or TEK variant, but did not show what effect miransertib had on wild type endothelial cells. Since these malformations occur in patients with mosaic expression of these variants and as the authors suggest, patients may require long term treatment, it would be helpful to know what impact miransertib has on the viability of wild type cells.
3. The extended version of the figures are mis-labeled and need to be corrected. EV3, should be EV4, EV4 should be EV5 and EV6 should be EV7.
4. References on lines 627 and 630 are not in alphabetical order
5. The nomenclature for Pik3caH1047R is inconsistent. It is sometimes italicized, but other times it is not. If the authors did not italicize it because they are referring to the protein with the H1047R variant, then PIK3CA should be capitalized.
6. The sentence on lines 277-281 could be stated more clearly.
7. Line 283, the word "it" is missing.
8. The sentence on lines 294-297 is not clear and needs to be re-worded.
9. Line 396, trypsinized is mis-spelled.

Referee #2 (Comments on Novelty/Model System for Author):

This is an excellent study involving a new preclinical mouse model (postnatal retinal ECs carrying a PIK3CA gain-of function mutation). The study also uses human EC cells carrying PIK3CA and TEK gain of function mutations.

Referee #2 (Remarks for Author):

The manuscript by Kobialka et al. summarizes work on low-flow vascular malformations that are caused by an activation of the PI3K/AKT pathway. They use a novel mouse model based on a well-established PI3KCAH1047R mutation that is induced in the postnatal mouse retina. Using this model, the authors perform a suppression study using the AKT inhibitor miransertib, which prevents both the formation and induces the regression of PI3KCA-dependent vascular malformations. This is also confirmed in human endothelial cells harboring the same gain of function mutation in PI3KCA. The manuscript is beautifully written and the

logic is clearly transparent. The figure panels are mostly of superb quality. I have only a few general comments to the manuscript as detailed below.

Major comments

1) How good is the specificity of miransertib, which is known to affect not only AKT1-3 but also other targets including αMβ2 integrin function and glycoprotein Ib/IX/V-mediated agglutination in platelets? It would be good to confirm this beneficial effect with another independent AKT inhibitor to verify the specificity of miransertib described in this study. This could be done for instance in human PIK3CA and TEK EC models.

2) Fig 2E shows images of EC-mTmG mouse retinas isolated at indicated time points stained with IB4 and GFP. For the P15 stage, there is a lot of background staining. For clarity of the data this should be improved (page 37).

3) Figs. 4D, 5D, 6D, and 7D have quantifications of EdU positive cells (incl. upon miransertib treatment). Here, the authors show the total number of ECs per 104 µm2 , which is about 2.5 fold higher in PI3KCA mutants. Then they also determine the EdU+ ECs per 104 µm2 , which are also increased. Here, they should determine the ratio of Edu+ ECs/total number of ECs. This would be a more meaningful representation of the proliferation rate.

4) Fig. 8G summarizes the IC50 effect of miransertib on TEK and PI3KCA mutant cells. How does miransertib affect viability of WT ECs in comparison ?

5) In Figure EV4C: Please, indicate whether there is a significant difference between the miransertib-treated PI3KCA mutant PC coverage.

Referee #3 (Remarks for Author):

The article from Kobialka et al describe a new mouse model of low flow vascular malformations due to PI3KCA gain of function mutation. Low flow vascular malformations are mostly related to PIK3CA or TEK mutations. Currently there is no satisfying mouse model of venous malformation and no drugs approved in this indication.

The manuscript is well written, easy to read, the figures are particularly clear and the approach is logic. The link between postnatal date and severity of disease is interesting.

However, I have two major concerns. The first one is related to the model and the second to the treatment.
While I really appreciate the effort of the team to develop a satisfying preclinical model of vascular malformations, the one used here is not a model of vascular malformation.
The mouse model is not recapitulating the phenotype of patients with vascular malformations limiting the significance of the manuscript. The vascular malformations seem to be only localized to the retina. In addition to visible superficial or deep malformation, patients usually present with thrombosis, hemorrhages, inflammatory flares or infection that are not described in this model.
The second concern is about the treatment. It is expected that AKT inhibitors improve the malformations but these drugs are associated with severe adverse events including hyperglycemia. The development of these drugs is heavily disputed in oncology. I was wondering why the authors have not used rapamycin to compare with a drug already approved in clinic for other indication?
I have also additional questions:

- Do the authors have explored mice with aging? Do these mice develop vascular malformation elsewhere? Pdgfr is not the most appropriate Cre since it expressed in other tissues than endothelial cells.
- Do the authors have tried to induce the Cre recombinase during embryo development?
- I was surprised to read that primary culture of endothelial cells carry 50% VAF. Does this mean that the mutation is not somatic but heterozygous?

Point-by-point response

Referee #1 (Comments on Novelty/Model System for Author):

The study described in this manuscript was well designed and executed. The results were presented in a clear and concise manner with both qualitative and quantitative analyses reported. The conclusions drawn were valid. The model system described recapitulates the vascular malformations seen in human disease with mosaic expression of PIK3CA and TEK pathogenic variants. Developing models for mosaic diseases is extremely challenging and often lead to heterogeneous phenotypes. This system has a consistent phenotype that develops over a short time frame and can be easily assessed, making it very attractive for use in testing therapeutics.

Referee #1 (Remarks for Author):

The paper by Kobialka, et. al. entitled "The onset of PI3K-related vascular malformations occurs during angiogenesis and is prevented by the AKT inhibitor miransertib", uses both cell culture and an in vivo retinal model to investigate the role of PIK3CAH1047R in vascular development and the efficacy of the AKT inhibitor, miransertib, for ameliorating the vascular malformations associated with this variant. The authors showed that murine vascular endothelial cells expressing Pik3caH1047R had enhanced cell cycle progression compared to wild type cells in the presence of serum. Using the murine retinal model, they showed that activation of Pik3caH1047R early in vascular development led to more extensive vascular malformations than when the variant was activated later in the remodeling process and concluded that active angiogenesis is necessary for malformations to occur. Next, they demonstrated that mosaic expression of Pik3caH1047R with low numbers of variant-positive cells was sufficient to develop malformations and showed that the resulting malformations demonstrated several characteristics of human low-flow vascular malformations. They then showed that both high and low doses of miransertib could reduce formation of vascular malformations when administered early, and cause regression of malformations when administered later in retinal vascular development. Finally, the authors showed that miransertib could reduce PI3K/AKT signaling and viability in endothelial cells isolated from human vascular malformations with both PIK3CA and TEK mutations. The authors conclude that Pik3caH1047R murine retinal model is a robust system for testing efficacy of treatment strategies for low-flow vascular malformations and that miransertib is a good candidate for future clinical trials in patients with PIK3CA or TEK vascular malformations.

This is a very well-designed and executed study. The experiments were clearly described and conclusions valid. The development of this model system will be extremely useful not only for assessing therapeutic strategies for PI3K-driven vascular malformations, but may also be an important tool for studying the mechanism by which Pik3caH1047R causes these malformations to develop. I have only a few minor comments for the authors.

We thank this Reviewer for the thorough and constructive review of our manuscript. We have now included her/his comments on the revised version of the manuscript and specified them below.

1. In lines 183-184, the authors state that with low dose 4-OHT activation, Pik3caH1047R only targeted veins and capillaries. How were the vessels identified? Was it just by morphology or were specific markers used to distinguish vessel types?

We thank this reviewer for this comment. The mouse retina is a unique and very powerful experimental model for the analysis of blood vessel growth during angiogenesis. During the angiogenic process, arteries, veins and capillaries are nicely distinguished (Pitulescu *et al*, 2010); being the former an area with low EC proliferation, low (surrounding) capillary network and a prominent coverage by smooth muscle cells (SMCs, which are positive for α-smooth muscle actin, αSMA). Instead, veins are composed of ECs which are proliferative, exhibit a dense capillary network in close proximity and are poorly covered by αSMC. Some of these features can be observed in the Figure R1, a representative image of a wild-type postnatal day (P) 7 mouse retina stained for αSMA. Indeed, mural cells (stained with αSMA) are present in arteries (yellow arrowheads) whereas veins (yellow asterisks) present a scarce SMC coverage and αSMA staining. Mural cells (αSMA) are absent in capillaries. We have used these parameters to analyze *Pik3ca*^{H1047R} postnatal retinas; thus, allowing to conclude that expression of *Pik3ca*^{H1047R} in ECs leads to abnormal growth in veins and capillaries but not in arteries.

We have clarified this in the Materials and Methods (page 20) of the revised manuscript.

2. In figure 8G, the authors showed decreased viability with increasing concentration of miransertib in human cell lines with a PIK3CA or TEK variant, but did not show what effect miransertib had on wild type endothelial cells. Since these malformations occur in patients with mosaic expression of these variants and as the authors suggest, patients may require long term treatment, it would be helpful to know what impact miransertib has on the viability of wild type cells.

As this Reviewer points out, long-term treatment may be required to treat these diseases and so it is very important to avoid undesired side effects in normal vasculature. As suggested by this Reviewer, we have now included new data assessing the impact of miransertib in PI3K/AKT signalling (Appendix Fig S3D) and cell viability (Figure 8G) in wild-type endothelial cells (HUVECs, human umbilical vein endothelial cells).

We show that miransertib also blocks PI3K/AKT signalling and reduces cell viability in a dose-dependent manner in wild type endothelial cells. However, the sensibility of wild type endothelial cells to miransertib treatment is reduced compared to *PIK3CA* and *TEK*-mutant endothelial cells (although not statistically significant: *PIK3CA*-mutant vs. HUVECs $p = 0.0815$; *TEK*-mutant vs. HUVECs: $p = 0.4085$; statistical analysis has been done using the extra sum-of-squares F test for the comparison of best-fit values; this analysis has been included in figure legend of Figure 8G).

Nevertheless, in vitro studies have some limitations that are counteracted by in vivo analysis. Our results using our in vivo model show that the impact of miransertib treatment on wild-type (control) mice have no detrimental effect on normal vasculature. In the revised version of the manuscript we have included statistical analysis of the comparisons on miransertib treatment on control mouse retinas (Figures 5, 6, and 7).

Our data suggest that miransertib treatment (especially low doses) might not alter the normal vasculature. However, we would like to acknowledge that future clinical studies should consider monitoring for this potential impact on non-malformed and development vasculature in humans.

3. The extended version of the figures are mis-labeled and need to be corrected. EV3, should be EV4, EV4 should be EV5 and EV6 should be EV7.

We thank this reviewer for pointing this out. This has now been corrected.

4. References on lines 627 and 630 are not in alphabetical order

This has now been corrected.

5. The nomenclature for Pik3caH1047R is inconsistent. It is sometimes italicized, but other times it is not. If the authors did not italicize it because they are referring to the protein with the H1047R variant, then PIK3CA should be capitalized.

Nomenclature for *Pik3ca*/PIK3CA has been carefully revised and modified accordingly. *Pik3ca* has been used when referring to the mouse gene. Instead, *PIK3CA* is used when referring to the human gene. PIK3CA when referring the protein.

6. The sentence on lines 277-281 could be stated more clearly.

As suggested by this reviewer, we have now replaced the original sentence:

from: "*Nevertheless, our results showing that pathological expansion of Pik3ca*^{H1047R} *ECs primary occurs in the presence of growth factors, and pS6, a downstream effector of AKT/mTOR, is chiefly activated in the presence of growth factors, suggest that the AKT/mTOR axis orchestrates the onset of Pik3ca*^{H1047R} *vascular malformations.*"

To: "*Nevertheless, our results show that pathological expansion of Pik3ca*^{H1047R} *ECs primary occurs in the presence of growth factors. Given that pS6, a downstream effector of AKT/mTOR,*

is selectively activated in the presence of growth factors in mutant cells, it suggests that the AKT/mTOR axis orchestrates the onset of Pik3ca^{H1047R} vascular malformations.”

7. Line 283, the word "it" is missing.

We are sorry but we are not sure where the “it” word should be included; instead, we have included a comma in this sentence.

“*Using the postnatal mouse retina as a model of vascular malformations in combination with a tamoxifen-induced strategy, which allows to mosaically express the activating H1047R Pik3ca mutation at different developmental times, we demonstrate that active angiogenesis is required for Pik3ca mutants to generate vascular malformations.”*

8. The sentence on lines 294-297 is not clear and needs to be re-worded.

We have now modified this sentence to make it more clear: “*However, despite of being considered as non-proliferative lesions at the time of diagnosis, acute production of extrinsic inputs such as hormonal changes, injury, or wound healing, can reactivate malformed vasculature (Pang et al, 2020); thereby being a key aspect to mind for preventive approaches*.”

to “*This also explains why low-flow vascular malformations are histopathologically characterized by a low rate of EC proliferation. However, acute production of extrinsic inputs such as hormonal changes, injury, and wound healing can reactivate proliferation in the malformed vasculature (Pang et al, 2020); being a key aspect to mind for preventive approaches*.”

9. Line 396, trypsinized is mis-spelled.

This has now been corrected.

Referee #2 (Comments on Novelty/Model System for Author):
This is an excellent study involving a new preclinical mouse model (postnatal retinal ECs carrying a PIK3CA gain-of function mutation). The study also uses human EC cells carrying PIK3CA and TEK gain of function mutations.

Referee #2 (Remarks for Author):
The manuscript by Kobialka et al. summarizes work on low-flow vascular malformations that are caused by an activation of the PI3K/AKT pathway. They use a novel mouse model based on a well-established PI3KCAH1047R mutation that is induced in the postnatal mouse retina. Using this model, the authors perform a suppression study using the AKT inhibitor miransertib, which prevents both the formation and induces the regression of PI3KCA-dependent vascular malformations. This is also confirmed in human endothelial cells harboring the same gain of function mutation in PI3KCA. The manuscript is beautifully written and the logic is clearly transparent. The figure panels are mostly of superb quality. I have only a few general comments to the manuscript as detailed below.

We thank this Reviewer for the thorough and constructive review of our manuscript. We have now included her/his comments on the revised version of the manuscript and specified them below.

Major comments

1) How good is the specificity of miransertib, which is known to affect not only AKT1-3 but also other targets including αMβ2 integrin function and glycoprotein Ib/IX/V-mediated agglutination in platelets? It would be good to confirm this beneficial effect with another independent AKT inhibitor to verify the specificity of miransertib described in this study. This could be done for instance in human PIK3CA and TEK EC models.

This is an interesting comment. The laboratory of Jaehyun Cho previously showed that miransertib blocks αMβ2 integrin function and glycoprotein Ib/IX/V-mediated agglutination in platelets and propose miransertib treatment for sickle cell disease (SCD) (Kim *et al*, 2017). In that study, they proposed that this effect is a result of AKT inhibition, instead of a direct targeting of miransertib on αMβ2 integrin function; these authors claim: "*These results suggest that specific AKT inhibition by ARQ 092 attenuates the membrane translocation and ligand-binding function of αMβ2 integrin in stimulated neutrophils isolated from SCD patients*". In line with these data, a previous study from this laboratory (Li *et al*, 2014) using Akt-isoforms knock-out mouse models, found that neutrophil Akt2 was critical for membrane translocation of αMβ2 integrin. Here, they observed similar inhibition on αMβ2 integrin function by using the AKT2-specific inhibitor Akti XII.

To further validate the impact of AKT inhibition in vascular malformations, we have performed a cell viability assay assessing the effect of the AKT inhibitor MK2206 (also commonly used for in

vitro studies) in wild type (HUVECs), *PIK3CA* and *TEK*-mutant primary endothelial cells from vascular malformations (Appendix Fig S3E). We demonstrate that MK2206 inhibitor also impairs cell viability in a dose-dependent manner in all genotypes. Of note, miransertib affected cell viability more effectively than MK2206 inhibitor (miransertib vs. MK2206: PIK3CA-mutant: $p < 0.0001$; TEK-mutant: $p = 0.0017$; wild type (HUVECs): $p = 0.0654$). Another interesting observation is that wild-type endothelial cells are slightly more sensitive (not statistically significant) to MK2206 inhibitor than *PIK3CA* and *TEK*-mutant endothelial cells.

Collectively, our data support the notion that mirasentib, by inhibiting AKT, is a promising therapeutic approach for the treatment of vascular malformation. We have now included these data in the revised manuscript (Appendix Fig S3E).

2) Fig 2E shows images of EC-mTmG mouse retinas isolated at indicated time points stained with IB4 and GFP. For the P15 stage, there is a lot of background staining. For clarity of the data this should be improved (page 37).

We have now changed this representative image to avoid background staining.

3) Figs. 4D, 5D, 6D, and 7D have quantifications of EdU positive cells (incl. upon miransertib treatment). Here, the authors show the total number of ECs per 10^4 µm2, which is about 2.5 fold higher in PI3KCA mutants. Then they also determine the EdU+ ECs per 10^4 µm2, which are also increased. Here, they should determine the ratio of Edu+ ECs/total number of ECs. This would be a more meaningful representation of the proliferation rate.

The reviewer raises an important point. We would like to clarify that these assays were performed by injecting EdU only 2h prior to the isolation of the retinas. The rationale behind this strategy was to get an accurate snapshot of the number of ECs which were proliferating in a specific time point. Hence, we believe that the correct way to represent our data is by expressing number of EdU+ ECs (as included in our first submission). The representation of EdU+ ECs/total number of ECs (when EdU is added only for 2h) does not result in differences between control and mutant retinas (there are many more EC in mutant retinas, hence the ratio is compensated). However, this is not unexpected given that many more ECs get accumulated over time in the Pik3ca mutant retinas. Instead, if EdU is administrated 24h prior to the sacrifice of the mice, *Pik3ca* mutant retinas exhibit a prominent and significant difference compared to control retinas when representing EdU+ ECs/total number of ECs (Figure R2). This supports the notion that expression of Pik3ca^{H1047R} leads to enhanced EC proliferation; and many more ECs accumulate through longer times.

We have now clarified this information in the revised manuscript (page 9).

4) Fig. 8G summarizes the IC50 effect of miransertib on TEK and PI3KCA mutant cells. How does miransertib affect viability of WT ECs in comparison?

This comment is similar to comment #2 of Reviewer 1.

We have now included new data assessing the impact of miransertib in PI3K/AKT signalling (Appendix Fig S3D) and cell viability (Figure 8G) in wild-type endothelial cells (HUVECs, human umbilical vein endothelial cells).

We show that miransertib also blocks PI3K/AKT signalling and reduces cell viability in a dose-dependent manner in wild type endothelial cells. However, the sensibility of wild type endothelial cells to miransertib treatment is reduced compared to *PIK3CA* and *TEK*-mutant endothelial cells (although not statistically significant: *PIK3CA*-mutant vs. HUVECs p = 0.0815; *TEK*-mutant vs. HUVECs: p = 0.4085; statistical analysis has been done using the extra sum-of-squares F test for the comparison of best-fit values; this analysis has been included in figure legend of Figure 8G).

Nevertheless, in vitro studies have some limitations that are counteracted by in vivo analysis. Our results using our in vivo model show that the impact of miransertib treatment on wild-type (control) mice have no detrimental effect on normal vasculature. In the revised version of the manuscript we have included statistical analysis of the comparisons on miransertib treatment on control mouse retinas (Figures 5, 6, and 7).

Our we suggest that miransertib treatment might not impact in normal vasculature; however, future clinical studies may consider monitoring for this potential impact on non-malformed and development vasculature.

5) In Figure EV4C: Please, indicate whether there is a significant difference between the miransertib-treated PI3KCA mutant PC coverage.

For this, we have included statistical analysis from the comparison of miransertib-treated EC-Pik3ca^{H1047R} mice and vehicle-treated control mice. This has shown that pericyte coverage in mutant retinas upon miransertib treatment is not fully prevented at P6; however, it is significantly increased compared to vehicle-treated EC-Pik3ca^{H1047R} mice.

We have also commented this in the manuscript text: "*Also, the treatment partially prevented the loss of NG2-positive mural cell coverage (Fig EV5)*"

Referee #3 ***(Remarks for Author):***

The article from Kobialka et al describe a new mouse model of low flow vascular malformations due to PI3KCA gain of function mutation. Low flow vascular malformations are mostly related to PIK3CA or TEK mutations. Currently there is no satisfying mouse model of venous malformation and no drugs approved in this indication.

The manuscript is well written, easy to read, the figures are particularly clear and the approach is logic. The link between postnatal date and severity of disease is interesting. However, I have two major concerns. The first one is related to the model and the second to the treatment. While I really appreciate the effort of the team to develop a satisfying preclinical model of vascular malformations, the one used here is not a model of vascular malformation.

We thank this Reviewer for the thorough and constructive review of our manuscript. We have now included her/his comments on the revised version of the manuscript and specified them below.

1. The mouse model is not recapitulating the phenotype of patients with vascular malformations limiting the significance of the manuscript. The vascular malformations seem to be only localized to the retina. In addition to visible superficial or deep malformation, patients usually present with thrombosis, hemorrhages, inflammatory flares or infection that are not described in this model.

We agree with Referee that our preclinical model using the mouse postnatal retina is not a systemic model of vascular malformations. However, it recapitulates the main hallmarks of the pathogenesis of PI3K-driven vascular malformations: (i) *PIK3CA* mutation is present in endothelial cells leading to enhanced PI3K/AKT/mTOR signalling, ii) vascular malformations occur specifically in veins and capillaries, iii) vascular malformations arise as a consequence of endothelial cell hyperproliferation. Besides, one of the main advantages of the postnatal retina is that it allows the development of vascular malformations in a very short time frame (one week) allowing the rapid and robust assessment of therapeutic responses. In contrast, this short time frame does not allow the development of overt systemic vascular malformations in other locations in our system.

We previously developed a mouse model for systemic vascular malformations by inducing the expression of *Pik3ca*^{H1047R} in the embryonic mesoderm which also developed other knock-on effects such as localized and internal hemorrhages and phlebectasia of major veins (Castillo *et al*, 2016). Yet, this mouse model presented important limitations for its use as a preclinical system; i) these mice showed high morbidity and mortality, ii) the variable penetrance of the mutation causing the disease, iii) the high heterogeneity of the phenotypes, and iv) the long time-frame to show therapeutic response to molecular therapies. In fact, these limitations inspired us to generate and use the retina model. Indeed, our data have proven that posnatal retinas are not only robust, rapid, and reliable for preclinical studies, but they are also instrumental for the understanding on how these lesions appear.

We have now included and discussed the limitations of the retinal model in the Discussion section of the revised version of the manuscript:

"However, this is not a systemic model and thus, it lacks some important aspects of the disease such as thrombosis, hemorrhages, phlebectasia or infection (Pang et al, 2020). We have previously modelled systemic vascular malformations by embryonic expression of Pik3ca^{H1047R} mutation in mice recapitulating some of these knock-on effects (Castillo et al, 2016a). This preclinical model showed that therapeutic reduction of vascular malformation may also eliminate and relieve some of these clinical manifestations such as hemorrhages or phlebectasia of main internal veins (Castillo et al, 2016a). Nevertheless, the high heterogeneity of this model and the long-time frame needed to assess therapeutic effectivity are important drawbacks for preclinical studies of molecular therapies."

2. The second concern is about the treatment. It is expected that AKT inhibitors improve the malformations but these drugs are associated with severe adverse events including hyperglycemia. The development of these drugs is heavily disputed in oncology. I was wondering why the authors have not used rapamycin to compare with a drug already approved in clinic for other indication?

We agree with the Referee that AKT inhibitors have shown adverse effects in oncology. Similarly, other inhibitors of the PI3K/AKT/mTOR signalling pathway, including PI3K inhibitors as well as rapamycin and rapalogues, have also shown adverse effect in oncology trials. These common adverse effects include hyperglycemia, rash, stomatitis and diarrhea; they are reversible with drug interruption and usually lessen or disappear upon dose reduction (Nunnery and Mayer, 2019). However, this did not stop the use of these inhibitors for compassionate use or are under clinical trials for congenital syndromes and vascular anomalies (Queisser *et al*., 2021). Importantly, it has now been approved the use of the PIK3CA inhibitor alpelisib/VIJOICE for PROS syndromes (https://www.fda.gov/drugs/resources-information-approved-drugs/fda-approves-alpelisib-pik3ca-related-overgrowth-spectrum).

To overcome reported side effects of inhibitors of the PI3K/AKT/mTOR signalling pathway described in oncology setting, these inhibitors are used at lower doses as compared with the doses used in oncology. Indeed, this led to less or no side effects. In the same line, miransertib has been used for compassionate use in a reduced number of patients with Proteus and PROS syndromes and was well tolerated with no significant toxicities reported or resolved spontaneously (Forde *et al*., 2021; Biesecker *et al*., 2020) indicating that miransertib may not show more adverse effects than other PI3K pathway inhibitors. While we are still learning from these pharmacotherapy studies, it is now clear that PI3K inhibitors (and derivates) have become essential tools to treat vascular malformations.

We would also like to clarify that we have not compared miransertib therapeutic effect with other inhibitors because the aim of this study was not to compare drug efficacy among inhibitors but to add new therapeutic opportunities for the treatment of PI3K-driven vascular malformations. In line with this, not all patients benefit or response similarly to current PI3K pathway inhibitors; also, they may have different adverse effects or may need to alternate therapeutic strategies; thus, we provide preclinical data to include miransertib as a promising therapeutic strategy for these diseases.

3. Do the authors have explored mice with aging? Do these mice develop vascular malformation elsewhere?

This Reviewer raises an interesting point. We and others have previously demonstrated that expression of Pik3ca^{H1047R} ubiquitously or in the embryonic mesoderm using other Cre mouse lines leads to lethality or morbidity due vascular abnormalities (Hare *et al*. 2015; Castillo *et al*. 2016). Thus, indicating the unique sensitivity of endothelial cells to activating Pik3ca mutations. We have also analyzed EC (Pdgfb-iCreER)-Pik3ca^{H1047R} mice 10 weeks after 4-hydroxytamoxifen administration (Figure R3). These mice develop vascular malformations in the uterus, mesentery, subcutaneously and in the muscle without showing any apparent phenotype involving other tissues. While these data support the strategy employed to generate mice with Pik3ca-related blood vascular malformations (expression of Pik3ca^{H1047R} in ECs by using the Pdgfb-iCreER line), their characterization are beyond the scope of this manuscript.

4. Pdgfr is not the most appropriate Cre since it expressed in other tissues than endothelial cells.

We would like to clarify that the CreER mouse model used is Pdgfb-CreiER, and not Pdgfrb-CreERT2. We are sorry that this information was not clear in the manuscript.

We agree that Pdgfb is expressed in other cell types different from endothelial cells such as macrophages, megakaryocytes, adipocytes, pancreatic endocrine cells, and langerhans cells (https://www.proteinatlas.org/). Yet, Pdgfb-iCreER mice have shown Cre activity in other cell types only under specific experimental contexts (Claxton *et al*. 2008; Zhang *et al*. 2020; Monelli *et al*. 2022; http://www.informatics.jax.org/allele/MGI:3793852). Importantly, in postnatal retinas, Cre activity in Pdgfb-iCreER mice has shown activity exclusively in endothelial cells (Claxton et al. 2008). This explain why the Pdgfb-iCreER inducible mouse line is widely used for the study of vascular phenotypes.

5. Do the authors have tried to induce the Cre recombinase during embryo development?

Several approaches have been pursuit to study the impact of *Pik3ca*^{H1047R} during embryonic development. On one hand, non-inducible lines (including ubiquitous and endothelial expression of Cre) have shown that generalized expression of *Pik3ca*^{H1047R} leads to embryonic development due to vascular defects (Hare *et al*. 2015; Berenjeno *et al*. 2017). On the other hand, mosaic expression of *Pik3ca*^{H1047R} mutation during embryo development (E7.5) in the lateral mesoderm results in a broad range of blood and lymphatic vascular malformations (Castillo *et al*. 2016). While these models have provided important biological insights into the relevant role of *Pik3ca* in endothelial cells, they are insufficient for preclinical studies, either because of their lethality or because they are not robust enough. As discussed above, we strongly believe that some of these limitations are overcome by the retina model; hence stressing its enormous potential for preclinical studies.

6. I was surprised to read that primary culture of endothelial cells carry 50% VAF. Does this mean that the mutation is not somatic but heterozygous?

Our data (endothelial cells carrying 50% VAF) indicate that the culture is pure (largely composed by heterozygous mutant endothelial cells). Also, it indicates that in vascular malformation the mutation is present in the endothelial cells, but not in the non-endothelial cell population. However, many studies have demonstrated that patients are mosaic, with *PIK3CA* or *TEK* largely presented in a somatic fashion. Yet, it is not clear whether all endothelial cells within these lesions are mutant or a mixed mutant/wild type endothelial cells are present in the malformed vasculature. Our observation that cell cultures of endothelial cells derived from vascular malformations´ biopsies carry 50% VAF suggests that the former scenario is more plausible. However, here we also show that *TEK* and *PIK3CA* activating mutations provide a proliferative advantage that may result in an enriched population in vitro. Both scenarios would be compatible with a cell culture composed by heterozygous mutant endothelial cells for signalling and cell viability comparison studies.

References

Berenjeno IM, Piñeiro R, Castillo SD, Pearce W, McGranahan N, Dewhurst SM, Meniel V, Birkbak NJ, Lau E, Sansregret L, Morelli D, Kanu N, Srinivas S, Graupera M, Parker VER, Montgomery KG, Moniz LS, Scudamore CL, Phillips WA, Semple RK, Clarke A, Swanton C, Vanhaesebroeck B.Oncogenic PIK3CA induces centrosome amplification and tolerance to genome doubling. Nat Commun. 2017 Nov 24;8(1):1773.

Biesecker LG, Edwards M, O'Donnell S, Doherty P, MacDougall T, Tith K, Kazakin J, and Schwartz B. Clinical report: one year of treatment of Proteus syndrome with miransertib (ARQ 092). Cold Spring Harb Mol Case Stud. 2020; 6(1) a004549.

Castillo SD, Baselga E & Graupera M (2019) PIK3CA mutations in vascular malformations. Curr Opin Hematol 26: 170–178.

Claxton S, Kostourou V, Jadeja S, Chambon P, Hodivala-Dilke K, Fruttiger M. Efficient, inducible Cre-recombinase activation in vascular endothelium. Genesis 2008. Feb;46(2):74-80.

Forde K, Resta N, Ranieri C, Rea D, Kubassova O, Hinton M, Andrews KA, Semple R, Irvine AD, Dvorakova V. Clinical experience with the AKT1 inhibitor miransertib in two children with PIK3CA-related overgrowth syndrome. Orphanet J Rare Dis. 2021 Feb 27;16(1):109.

Hare LM, Schwarz Q, Wiszniak S, Gurung R, Montgomery KG, Mitchell CA, Phillips WA. Heterozygous expression of the oncogenic Pik3ca(H1047R) mutation during murine development results in fatal embryonic and extraembryonic defects. Dev Biol. 2015 Aug 1;404(1):14-26.

Kim K, Li J, Barazia A, Tseng A, Youn SW, Abbadessa G, Yu Y, Schwartz B, Andrews RK, Gordeuk VR, et al (2017) ARQ 092, an orally-available, selective AKT inhibitor, attenuates neutrophil-platelet interactions in sickle cell disease. Haematologica 102: 246–259

Li J, Kim K, Hahm E, Molokie R, Hay N, Gordeuk VR, Du X & Cho J (2014) Neutrophil AKT2 regulates heterotypic cell-cell interactions during vascular inflammation. J Clin Invest 124: 1483–1496.

Nunnery SE and Mayer IA. Management of toxicity to isoform α-specific PI3K inhibitors. Ann Oncol 2019;30(Suppl_10):21-26.

Pitulescu ME, Schmidt I, Benedito R & Adams RH (2010) Inducible gene targeting in the neonatal vasculature and analysis of retinal angiogenesis in mice. Nat Protoc 2010 59 5: 1518–1534.

Queisser A, Seront E, Boon LM and Vikkula M. Genetic Basis and Therapies for Vascular Anomalies. Circ Res 2021; 129(1):155-173.

Zhang J, Muri J, Fitzgerald G, Gorski T, Gianni-Barrera R, Masschelein E, D'Hulst G, Gilardoni P, Turiel G, Fan Z, Wang T, Planque M, Carmeliet P, Pellerin L, Wolfrum C, Fendt SM, Banfi A, Stockmann C, Soro-Arnáiz I, Kopf M, De Bock K. Endothelial Lactate Controls Muscle Regeneration from Ischemia by Inducing M2-like Macrophage Polarization. Cell Metab. 2020 Jun 2;31(6):1136-1153.e7.

Figures

Figure R1. Identification of arteries, veins and capillaries in mouse retina. Representative image of WT P7 retina immunostained for blood vessels (IB4), EC nuclei marker (Erg) and alpha smooth muscle actin (αSMA), a marker specific for arteries. Yellow asterisks show veins and yellow arrowheads point at arteries. Scale bars: 150 μm.

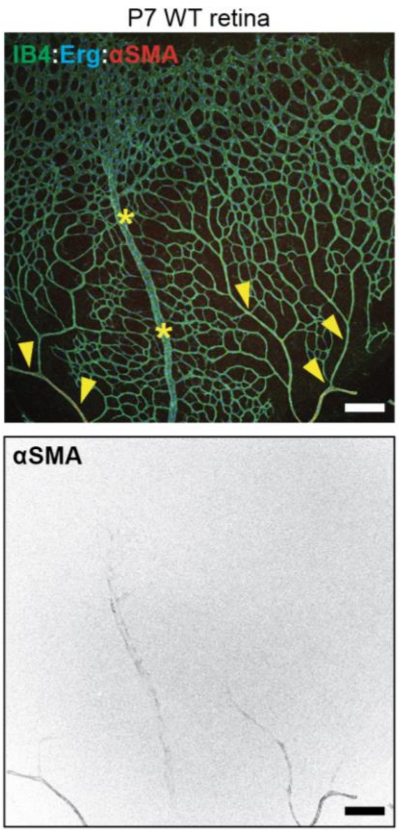

Figure R2. Impact of *Pik3ca* activation on EC proliferation during retinal angiogenesis. (**A**) Experimental setup showing 4-OHT and EdU dosing. (**B**) Representative confocal images of P6 retinas isolated from control and EC-*Pik3ca*$^{H1047R/WT}$ mouse littermates immunostained for blood vessels (IB4), EC nuclei marker (Erg) and proliferative cells (EdU). Scale bars: 150 µm. (**C**) High magnification images of representative areas showing EC nuclei (Erg) and proliferative cells (EdU) as well as blood vessels (IB4). Yellow circles point proliferative ECs (both Erg and EdU positive). Scale bars: 30 µm. (**D**) Quantification of EdU positive ECs per analysed field (10^4 um^2). (**E**) Quantification of proliferative ECs presented as a ration of EdU positive ECs per total number of EC in analysed field. n ≥ 4 retinas per genotype. Statistical analysis was performed by nonparametric Mann–Whitney test. *p < 0.05 was considered statistically significant.

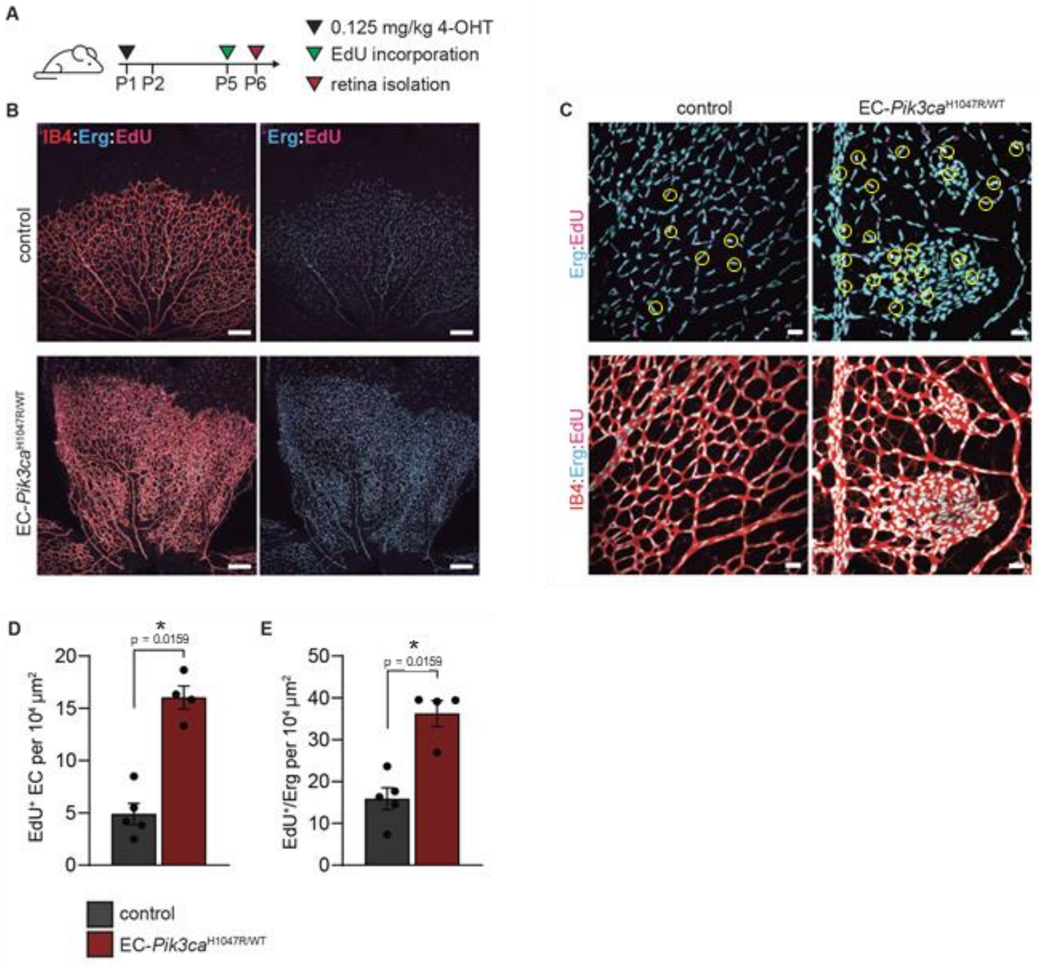

Figure R3. Systemic effect of EC-specific *Pik3ca*^{H1047R} expression. (A) Experimental setup showing 4-OHT and sacrificing time point. (**B**) Description of phenotypes found in adult EC-*Pik3ca*$^{H1047R/WT}$ animals. (**C**) Macro- and microscopic (H&E staining) images of depicted tissues obtained from *Pik3ca*$^{H1047R/WT}$ animals described. Yellow arrowheads point vascular malformations and orange asterisks show enlarged lumen of the endometrium.

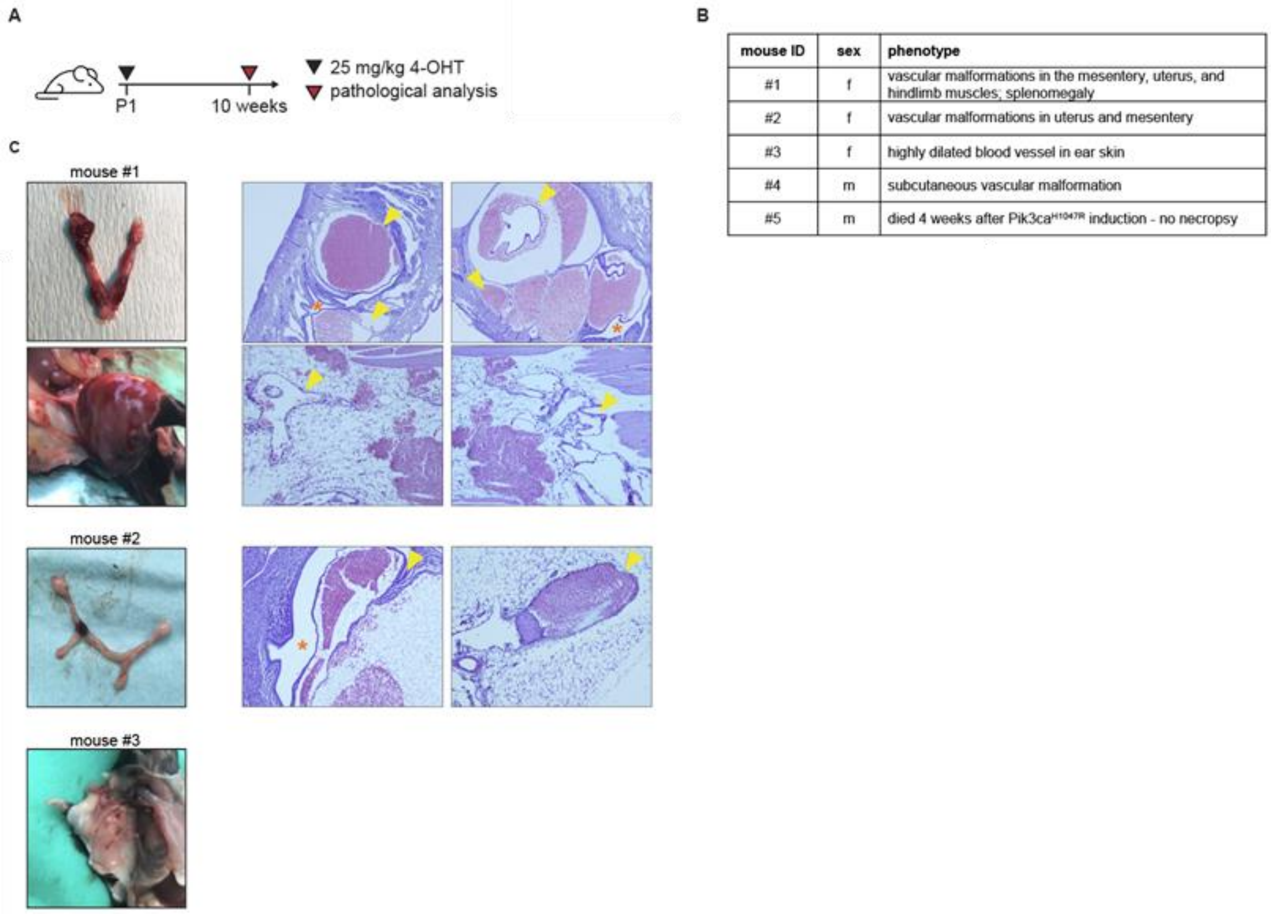

mouse ID	sex	phenotype
#1	f	vascular malformations in the mesentery, uterus, and hindlimb muscles; splenomegaly
#2	f	vascular malformations in uterus and mesentery
#3	f	highly dilated blood vessel in ear skin
#4	m	subcutaneous vascular malformation
#5	m	died 4 weeks after Pik3ca^{H1047R} induction - no necropsy

11th May 2022

Dear Dr. Castillo,

Thank you for the submission of your manuscript to EMBO Molecular Medicine. We have now received the enclosed reports from the two referees who re-reviewed your manuscript. As you will see, while referee #2 is mostly satisfied with the revisions, referee #3 raises remaining concerns. We discussed further with the referees the necessity (or not) to perform additional in vivo experiments, and we concluded that at this point, addressing the remaining issues by discussing them in the text would be sufficient.
I am therefore pleased to inform you that we will be able to accept your manuscript once the following points will be addressed:

1/ Please discuss the remaining concerns raised by referees #2 and #3.

2/ Main manuscript text:
- Please accept the changes and remove the red/highlighted text. Only keep in track changes mode any new modification.
- Material and Methods:
o Please indicate the origin and gender of the mice.
o Please indicate whether the cells were tested for mycoplasma contamination
o For the clinical samples, please include a statement that informed consent was obtained from all subjects and that the experiments conformed to the principles set out in the WMA Declaration of Helsinki and the Department of Health and Human Services Belmont Report.

2/ Checklist:
Thank you for providing the completed checklist. Please carefully check the indicated page numbers.

3/ Appendix:
Please also provide in the appendix figures the exact p values, not a range.

4/ Source Data:
Thank you for providing source data supporting your figures. Please double-check the following:
- labeling of source data for EV figures
- blots seem to be missing for SD Fig 1D
- uncropped images for Fig S3C
- if possible, please provide source data for Fig 8B and S3B in a different format

5/ Thank you for providing The Paper Explained. I added minor modifications, please amend as you see fit:
PROBLEM: Low-flow vascular malformations are congenital diseases caused by a focal overgrowth of vessels. They may cause pain, bleeding, infections, and obstruction of organs. While their genetic causes, leading to hyperactivation of PI3K signaling, have been known for a few years, no targeted molecular therapy has been approved so far.
RESULTS: Pik3ca mutation in ECs activates transcriptomic changes, leading to enhanced cell cycle progression in the presence of growth factors. We have generated a robust and fast preclinical in vivo model that allows for testing of targeted drugs. With this model, we demonstrated that PI3K-driven vascular malformations rely on active angiogenesis. Our preclinical studies show that AKT inhibition using miransertib prevents the disease and fully regresses established vascular malformations.
IMPACT: Our new in vivo model of PI3K-driven vascular malformations constitutes a reliable and fast preclinical setting to test new or repurposed targeted drugs. Our studies support that Pik3ca-mutant ECs cause vascular malformations upon growth stimuli, highlighting the importance of preventive therapeutic approaches after invasive treatments. We provide proof of concept for the use of the AKT inhibitor miransertib in PI3K-driven vascular malformations, which opens a new window for targeted therapeutic intervention for these diseases. We further demonstrate in vitro that this targeted therapy is similarly effective in both PIK3CA and TEK-mutant vascular malformations.

6/ Thank you for providing a nice synopsis picture. Please upload it as a PNG/JPEG/TIFF file 550 px wide x 300-600 px high and make sure that the text remains legible.

7/ As part of the EMBO Publications transparent editorial process initiative (see our Editorial at http://embomolmed.embopress.org/content/2/9/329), EMBO Molecular Medicine will publish online a Review Process File (RPF) to accompany accepted manuscripts.
This file will be published in conjunction with your paper and will include the anonymous referee reports, your point-by-point response and all pertinent correspondence relating to the manuscript. Let us know whether you agree with the publication of the RPF and as here, if you want to remove or not any figures from it prior to publication.

I look forward to receiving your revised manuscript.

With my best wishes,

Lise

Lise Roth, Ph.D
Senior Editor
EMBO Molecular Medicine

To submit your manuscript, please follow this link:
Link Not Available

*Additional important information regarding Figures

Each figure should be given in a separate file and should have the following resolution:
Graphs 800-1,200 DPI
Photos 400-800 DPI
Colour (only CMYK) 300-400 DPI"

Figures are not edited by the production team. All lettering should be the same size and style; figure panels should be indicated by capital letters (A, B, C etc). Gridlines are not allowed except for log plots. Figures should be numbered in the order of their appearance in the text with Arabic numerals. Each Figure must have a separate legend and a caption is needed for each panel.

*Additional important information regarding figures and illustrations can be found at https://bit.ly/EMBOPressFigurePreparationGuideline. See also figure legend preparation guidelines: https://www.embopress.org/page/journal/17574684/authorguide#figureformat

The system will prompt you to fill in your funding and payment information. This will allow Wiley to send you a quote for the article processing charge (APC) in case of acceptance. This quote takes into account any reduction or fee waivers that you may be eligible for. Authors do not need to pay any fees before their manuscript is accepted and transferred to our publisher.

***** Reviewer's comments *****

Referee #2 (Remarks for Author):

The manuscript by Kobialka and colleagues is substantially improved and of high quality. Also, the authors have addressed most of my and the other reviewers' comments. With the exception of one comment that needs further clarification, the manuscript appears to be of sufficient quality for publication with EMM (pending the clarification of that issue).

I had previously stated the following comment: "[In Fig. 4...] the authors show the total number of ECs per 104 µm2, which is about 2.5 fold higher in PI3KCA mutants. Then they also determine the EdU+ ECs per 104 µm2, which are also increased. Here, they should determine the ratio of Edu+ ECs/total number of ECs. This would be a more meaningful representation of the proliferation rate."

The authors responded with the following summary: "The reviewer raises an important point. We would like to clarify that these assays were performed by injecting EdU only 2h prior to the isolation of the retinas. The rationale behind this strategy was to get an accurate snapshot of the number of ECs which were proliferating in a specific time point. Hence, we believe that the correct way to represent our data is by expressing number of EdU+ ECs (as included in our first submission). The representation of EdU+ ECs/total number of ECs (when EdU is added only for 2h) does not result in differences between control and mutant retinas (there are many more EC in mutant retinas, hence the ratio is compensated)."

This means, contrary to their statement, the proliferation rate at that time is not increased.

"However, this is not unexpected given that many more ECs get accumulated over time in the Pik3ca mutant retinas. Instead, if EdU is administrated 24h prior to the sacrifice of the mice, Pik3ca mutant retinas exhibit a prominent and significant difference

compared to control retinas when representing EdU+ ECs/total number of ECs (Figure R2). This supports the notion that expression of Pik3caH1047R leads to enhanced EC proliferation; and many more ECs accumulate through longer times. We have now clarified this information in the revised manuscript (page 9)"

Within the revised manuscript, the authors write: "A 2 hours snapshot of the number of ECs in S-phase unveiled that focal PI3K signalling activation resulted in enhanced EC proliferation (Fig 4C, E). In turn, this led to an accumulation of ECs which explains the overall enhanced vascularity (Fig 4C, F, G)."

However, there is no evidence of an enhanced proliferation based on this data. This data does not provide a mitotic index that would be required to support this statement. Based on their data, the larger number of PIK3CA+ cells results in more proliferative events. But the data provided is not in support of a higher proliferation rate, which is comparable or even lower than in WT. Multiple alternative scenarios could explain why there is an increased cell count in that PIK3CA condition (different time window of proliferation, less cell apoptosis, different cell sizes, closer arrangement of cells within vessels, etc.).

I would suggest the authors rephrase this conclusion. Also, they could discuss the potential roles of miransertib in affecting these malformations. It is plausible that other mechanisms such as cell morphology, cell accumulation, morphology of the vessels etc may be impacted by this compound.

Otherwise, the authors have substantially improved the quality of the manuscript. They have now further supported their pharmaceutical study with another AKT inhibitor (MK2206), which substantiates the notion that targeting AKT is a promising approach in PIK3CA-driven vascular malformations. Also, the quality of some images has been improved (i.e. Fig. 2E). The effect of miransertib on WT endothelial cells has been tested. This latter data suggests that the compound also impacts the survival of WT cells in almost the same way as that of mutant cells.

Referee #3 (Remarks for Author):

The authors have well addressed the remarks but I am still not fully convinced that this model can be considered as a model of venous malformations.
In addition, as the authors mentioned in their rebuttal letter, Alpelisib was recently approved by the US FDA for patients with PROS. I strongly believe that additional experiments are indeed mandatory to compare the efficacy of Alpelisib vs. Miransertib in this model.

Point-by-point response

Referee #2 (Remarks for Author):

The manuscript by Kobialka and colleagues is substantially improved and of high quality. Also, the authors have addressed most of my and the other reviewers' comments. With the exception of one comment that needs further clarification, the manuscript appears to be of sufficient quality for publication with EMM (pending the clarification of that issue).

I had previously stated the following comment: "[In Fig. 4...] the authors show the total number of ECs per 104 µm2, which is about 2.5 fold higher in PI3KCA mutants. Then they also determine the EdU+ ECs per 104 µm2, which are also increased. Here, they should determine the ratio of Edu+ ECs/total number of ECs. This would be a more meaningful representation of the proliferation rate."

The authors responded with the following summary: "The reviewer raises an important point. We would like to clarify that these assays were performed by injecting EdU only 2h prior to the isolation of the retinas. The rationale behind this strategy was to get an accurate snapshot of the number of ECs which were proliferating in a specific time point. Hence, we believe that the correct way to represent our data is by expressing number of EdU+ ECs (as included in our first submission). The representation of EdU+ ECs/total number of ECs (when EdU is added only for 2h) does not result in differences between control and mutant retinas (there are many more EC in mutant retinas, hence the ratio is compensated)."

This means, contrary to their statement, the proliferation rate at that time is not increased.

"However, this is not unexpected given that many more ECs get accumulated over time in the Pik3ca mutant retinas. Instead, if EdU is administrated 24h prior to the sacrifice of the mice, Pik3ca mutant retinas exhibit a prominent and significant difference compared to control retinas when representing EdU+ ECs/total number of ECs (Figure R2). This supports the notion that expression of Pik3caH1047R leads to enhanced EC proliferation; and many more ECs accumulate through longer times. We have now clarified this information in the revised manuscript (page9)"

Within the revised manuscript, the authors write: "A 2 hours snapshot of the number of ECs in S-phase unveiled that focal PI3K signalling activation resulted in enhanced EC proliferation (Fig 4C, E). In turn, this led to an accumulation of ECs which explains the overall enhanced vascularity (Fig4C,F,G)."

However, there is no evidence of an enhanced proliferation based on this data. This data does not provide a mitotic index that would be required to support this statement. Based on their data, the larger number of PIK3CA+ cells results in more proliferative events. But the data provided is

not in support of a higher proliferation rate, which is comparable or even lower than in WT. Multiple alternative scenarios could explain why there is an increased cell count in that PIK3CA condition (different time window of proliferation, less cell apoptosis, different cell sizes, closer arrangement of cells within vessels, etc.).

I would suggest the authors rephrase this conclusion. Also, they could discuss the potential roles of miransertib in affecting these malformations. It is plausible that other mechanisms such as cell morphology, cell accumulation, morphology of the vessels etc may be impacted by this compound.

Otherwise, the authors have substantially improved the quality of the manuscript. They have now further supported their pharmaceutical study with another AKT inhibitor (MK2206), which substantiates the notion that targeting AKT is a promising approach in PIK3CA-driven vascular malformations. Also, the quality of some images has been improved (i.e. Fig. 2E). The effect of miransertib on WT endothelial cells has been tested. This latter data suggests that the compound also impacts the survival of WT cells in almost the same way as that of mutant cells.

We thank this reviewer for raising this point. First, we would like to apologize as this reviewer is right that our previous answer did not adequately address the reviewer's concern that "the proliferation rate of ECs is increased upon expression of *Pik3ca*^{H1047R} in the retinal vasculature at the studied time point". Indeed, we agree with this reviewer that if there are more ECs, the probability to capture more EdU positive cells in a short window of time is increased. We also agree with this reviewer that other alternative scenarios such as cell apoptosis come into play in our phenotype. However, we would like to clarify that apoptosis does not largely occur in the mouse postnatal retina (Franco *et al*, 2015). Although changes in cell size or cell arrangements may be impacted by PI3K activation in ECs, but we do not have any evidence for this mechanism in our model and believe that these would not be unique in such strong phenotype.

Hence, in order to accurately demonstrate this point, we have now calculated the mitotic index at P4 (Fig EV2). We have chosen this time point as by then mutant retinas exhibit emerging vascular malformations without accumulation of too many ECs. Upon performing an EdU-incorporation assay at P4, we show that expression of *Pik3ca*^{H1047R} induces a substantial increase in the number of ECs in S-phase. While these data were partially included in our previous version of the manuscript, we have decided to discuss it earlier in the new revised version of the manuscript.

Reference:

Franco CA, Jones ML, Bernabeu MO, Geudens I, Mathivet T, Rosa A, Lopes FM, Lima AP, Ragab A, Collins RT, et al. (2015) Dynamic endothelial cell rearrangements drive developmental vessel regression. PLoS Biol. 13(4):e1002125.

Referee #3 (Remarks for Author):

The authors have well addressed the remarks but I am still not fully convinced that this model can be considered as a model of venous malformations.
In addition, as the authors mentioned in their rebuttal letter, Alpelisib was recently approved by the US FDA for patients with PROS. I strongly believe that additional experiments are indeed mandatory to compare the efficacy of Alpelisib vs. Miransertib in this model.

We agree with this referee that this is not a systemic model of venous malformations. However, as stated previously, it recapitulates the main hallmarks of the pathogenesis of PI3K-driven vascular malformations in a very short time frame (one week) allowing the rapid and robust assessment of therapeutic responses.

We believe that the approval of alpelisib by the US FDA for patients with PROS highlights the importance of the development of preclinical models for PI3K-related congenital syndromes. We also agree that comparison of PI3K signaling pathway inhibitors may help to refine therapeutic strategies for these diseases. However, these are early days for patient stratification for PI3K-related disorders; therefore, preclinical assessment of new molecular drugs is critical to maximize therapeutic opportunities for these patients. In line with this, clinical evidence has shown that PI3K-related disorders may require chronic treatment of low dose of these inhibitors; thus, alternation of therapies may be required to avoid side effects and the development of drug resistance.

We have included this in the Discussion section as follows:
"In addition, comparison of different PI3K signalling pathway inhibitors may contribute to the refinement of therapeutic strategies for these diseases. Since these are early days for patient stratification for PI3K-related disorders, preclinical assessment of new molecular drugs is critical to maximize therapeutic opportunities for these patients. Indeed, these patients may need long-term or even chronic treatment; thus alternation therapies over time using effective low dose of inhibitor may be required to avoid side effects and the development of drug resistance."

20th May 2022

Dear Dr. Castillo,

Thank you for submitting the revised files. I am pleased to inform you that your manuscript is accepted for publication and is now being sent to our publisher to be included in the next available issue of EMBO Molecular Medicine!

Congratulations on your interesting work!

With kind regards,

Lise

Lise Roth, Ph.D
Senior Editor
EMBO Molecular Medicine

Follow us on Twitter @EmboMolMed
Sign up for eTOCs at embopress.org/alertsfeeds

EMBO Press Author Checklist

Corresponding Author Name: Mariona Graupera, Sandra D. Castillo
Journal Submitted to: EMBO Molecular Medicine
Manuscript Number: EMM-2021-15619

USEFUL LINKS FOR COMPLETING THIS FORM
The EMBO Journal - Author Guidelines
EMBO Reports - Author Guidelines
Molecular Systems Biology - Author Guidelines
EMBO Molecular Medicine - Author Guidelines

Reporting Checklist for Life Science Articles (updated January 2022)
This checklist is adapted from Materials Design Analysis Reporting (MDAR) Checklist for Authors. MDAR establishes a minimum set of requirements in transparent reporting in the life sciences (see Statement of Task: 10.31222/osf.io/9sm4x). Please follow the journal's guidelines in preparing your manuscript.
Please note that a copy of this checklist will be published alongside your article.

Abridged guidelines for figures
1. Data
The data shown in figures should satisfy the following conditions:
- the data were obtained and processed according to the field's best practice and are presented to reflect the results of the experiments in an accurate and unbiased manner.
- ideally, figure panels should include only measurements that are directly comparable to each other and obtained with the same assay.
- plots include clearly labeled error bars for independent experiments and sample sizes. Unless justified, error bars should not be shown for technical replicates.
- if n<5, the individual data points from each experiment should be plotted. Any statistical test employed should be justified.
- Source Data should be included to report the data underlying figures according to the guidelines set out in the authorship guidelines on Data Presentation.

2. Captions
Each figure caption should contain the following information, for each panel where they are relevant:
- a specification of the experimental system investigated (eg cell line, species name).
- the assay(s) and method(s) used to carry out the reported observations and measurements.
- an explicit mention of the biological and chemical entity(ies) that are being measured.
- an explicit mention of the biological and chemical entity(ies) that are altered/varied/perturbed in a controlled manner.
- the exact sample size (n) for each experimental group/condition, given as a number, not a range;
- a description of the sample collection allowing the reader to understand whether the samples represent technical or biological replicates (including how many animals, litters, cultures, etc.).
- a statement of how many times the experiment shown was independently replicated in the laboratory.
- definitions of statistical methods and measures:
  - common tests, such as t-test (please specify whether paired vs. unpaired), simple χ2 tests, Wilcoxon and Mann-Whitney tests, can be unambiguously identified by name only, but more complex techniques should be described in the methods section;
  - are tests one-sided or two-sided?
  - are there adjustments for multiple comparisons?
  - exact statistical test results, e.g., P values = x but not P values < x;
  - definition of 'center values' as median or average;
  - definition of error bars as s.d. or s.e.m.

Please complete ALL of the questions below.
Select "Not Applicable" only when the requested information is not relevant for your study.

Materials

Newly Created Materials	Information included in the manuscript?	In which section is the information available? (Reagents and Tools Table, Materials and Methods, Figures, Data Availability Section)
New materials and reagents need to be available; do any restrictions apply?	Not Applicable	

Antibodies	Information included in the manuscript?	In which section is the information available? (Reagents and Tools Table, Materials and Methods, Figures, Data Availability Section)
For **antibodies** provide the following information:<br>- Commercial antibodies: RRID (if possible) or supplier name, catalogue number and or/clone number<br>- Non-commercial: RRID or citation	Yes	Materials and Methods: pages 18, 20, 22. 23

DNA and RNA sequences	Information included in the manuscript?	In which section is the information available? (Reagents and Tools Table, Materials and Methods, Figures, Data Availability Section)
**Short novel DNA or RNA including primers, probes**: provide the sequences.	Yes	Materials and Methods: pages 22, 23; Appendix Table S3

Cell materials	Information included in the manuscript?	In which section is the information available? (Reagents and Tools Table, Materials and Methods, Figures, Data Availability Section)
**Cell lines:** Provide species information, strain. Provide accession number in repository **OR** supplier name, catalog number, clone number, and/**OR** RRID.	Not Applicable	
**Primary cultures:** Provide species, strain, sex of origin, genetic modification status.	Yes	Materials and Methods: pages 17, 18, 22
Report if the cell lines were recently **authenticated** (e.g., by STR profiling) and tested for mycoplasma contamination.	Not Applicable	

Experimental animals	Information included in the manuscript?	In which section is the information available? (Reagents and Tools Table, Materials and Methods, Figures, Data Availability Section)
**Laboratory animals or Model organisms:** Provide species, strain, sex, age, genetic modification status. Provide accession number in repository **OR** supplier name, catalog number, clone number, **OR** RRID.	Yes	Materials and Methods: page 17
**Animal observed in or captured from the field:** Provide species, sex, and age where possible.	Not Applicable	
Please detail **housing and husbandry conditions**.	Yes	Materials and Methods: page 17

Plants and microbes	Information included in the manuscript?	In which section is the information available? (Reagents and Tools Table, Materials and Methods, Figures, Data Availability Section)
**Plants:** provide species and strain, ecotype and cultivar where relevant, unique accession number if available, and source (including location for collected wild specimens).	Not Applicable	
**Microbes:** provide species and strain, unique accession number if available, and source.	Not Applicable	

Human research participants	Information included in the manuscript?	In which section is the information available? (Reagents and Tools Table, Materials and Methods, Figures, Data Availability Section)
If collected and within the bounds of privacy constraints report on age, sex and gender or ethnicity for all study participants.	Yes	Materials and Methods: page 22, Appendix Table S2

Core facilities	Information included in the manuscript?	In which section is the information available? (Reagents and Tools Table, Materials and Methods, Figures, Data Availability Section)
If your work benefited from core facilities, was their service mentioned in the acknowledgments section?	Yes	Materials and Methods: page 17

Design

Study protocol	Information included in the manuscript?	In which section is the information available? (Reagents and Tools Table, Materials and Methods, Figures, Data Availability Section)
If study protocol has been **pre-registered, provide DOI in the manuscript**. For clinical trials, provide the trial registration number **OR** cite DOI.	Not Applicable	
Report the **clinical trial registration number** (at ClinicalTrials.gov or equivalent), where applicable.	Not Applicable	

Laboratory protocol	Information included in the manuscript?	In which section is the information available? (Reagents and Tools Table, Materials and Methods, Figures, Data Availability Section)
Provide DOI OR other citation details if **external detailed step-by-step protocols** are available.	Not Applicable	

Experimental study design and statistics	Information included in the manuscript?	In which section is the information available? (Reagents and Tools Table, Materials and Methods, Figures, Data Availability Section)
Include a statement about **sample size** estimate even if no statistical methods were used.	Yes	Each scientific question in which statistical analysis was performed, was addressed in a pilot experiment using n=3 per group or based on similar previously published experiments. With these results, power analysis on these [illegible]
Were any steps taken to minimize the effects of subjective bias when allocating animals/samples to treatment (e.g. **randomization procedure**)? If yes, have they been described?	Not Applicable	Genotypes were not known at the time of treatment and immunostaining. Image acquisition and quantification were done in an automated way.
Include a statement about **blinding** even if no blinding was done.	Not Applicable	No blinding was performed. Genotypes were not known at the time of treatment and immunostaining.
Describe **inclusion/exclusion criteria** if samples or animals were excluded from the analysis. Were the criteria pre-established?<br><br>If sample or data points were omitted from analysis, report if this was due to attrition or intentional exclusion and provide justification.	Not Applicable	No data were excluded from the analysis.
For every figure, are **statistical tests** justified as appropriate? Do the data meet the assumptions of the tests (e.g., normal distribution)? Describe any methods used to assess it. Is there an estimate of variation within each group of data? Is the variance similar between the groups that are being statistically compared?	Yes	All statistical tests used are included in figure legends. For in vitro (Fig 1, S1) and in vivo studies (Fig. 2, 3, 4, 5, 6, and 7) given that n<10, non-parametric tests have been applied. Data between two groups were compared with unpaired two-tailed Mann–Whitney test. Statistical analysis of Fig S2D was done using one-tail t test (hypothesis-driven) assuming equal variances. For IC50 comparison (Fig. 8G) statistical analysis was performed by comparison of [illegible]

Sample definition and in-laboratory replication	Information included in the manuscript?	In which section is the information available? (Reagents and Tools Table, Materials and Methods, Figures, Data Availability Section)
In the figure legends: state number of times the experiment was **replicated** in laboratory.	Yes	Figure legends: page 35-39; Appendix Figure legends: pages 3, 4 (Appendix)
In the figure legends: define whether data describe **technical or biological replicates**.	Yes	Figure legends: page 35-39; Appendix Figure legends: pages 3, 4 (Appendix)

Ethics

Ethics	Information included in the manuscript?	In which section is the information available? (Reagents and Tools Table, Materials and Methods, Figures, Data Availability Section)
Studies involving **human participants**: State details of **authority granting ethics approval** (IRB or equivalent committee(s), provide reference number for approval.	Yes	Materials and Methods: page 22
Studies involving **human participants**: Include a statement confirming that **informed consent** was obtained from all subjects and that the experiments conformed to the principles set out in the WMA Declaration of Helsinki and the Department of Health and Human Services Belmont Report.	Yes	Materials and Methods: page 22
Studies involving **human participants:** For publication of **patient photos**, include a statement confirming that consent to publish was obtained.	Not Applicable	
Studies involving experimental **animals**: State details of **authority granting ethics approval** (IRB or equivalent committee(s), provide reference number for approval. Include a statement of compliance with ethical regulations.	Yes	Materials and Methods: page 17
Studies involving **specimen and field samples:** State if relevant **permits** obtained, provide details of authority approving study; if none were required, explain why.	Not Applicable	

Dual Use Research of Concern (DURC)	Information included in the manuscript?	In which section is the information available? (Reagents and Tools Table, Materials and Methods, Figures, Data Availability Section)
Could your study fall under dual use research restrictions? Please check biosecurity documents and list of **select agents and toxins** (CDC): https://www.selectagents.gov/sat/list.htm	Not Applicable	
If you used a select agent, is the security level of the lab appropriate and reported in the manuscript?	Not Applicable	
If a study is subject to dual use research of concern regulations, is the name of the **authority granting approval and reference number** for the regulatory approval provided in the manuscript?	Not Applicable	

Reporting

The MDAR framework recommends adoption of discipline-specific guidelines, established and endorsed through community initiatives. Journals have their own policy about requiring specific guidelines and recommendations to complement MDAR.

Adherence to community standards	Information included in the manuscript?	In which section is the information available? (Reagents and Tools Table, Materials and Methods, Figures, Data Availability Section)
State if relevant guidelines or checklists (e.g., **ICMJE, MIBBI, ARRIVE, PRISMA**) have been followed or provided.	Not Applicable	
For **tumor marker prognostic studies,** we recommend that you follow the **REMARK** reporting guidelines (see link list at top right). See author guidelines, under 'Reporting Guidelines'. Please confirm you have followed these guidelines.	Not Applicable	
For **phase II and III randomized controlled trials**, please refer to the **CONSORT** flow diagram (see link list at top right) and submit the CONSORT checklist (see link list at top right) with your submission. See author guidelines, under 'Reporting Guidelines'. Please confirm you have submitted this list.	Not Applicable	

Data Availability

Data availability	Information included in the manuscript?	In which section is the information available? (Reagents and Tools Table, Materials and Methods, Figures, Data Availability Section)
Have **primary datasets** been deposited according to the journal's guidelines (see 'Data Deposition' section) and the respective accession numbers provided in the Data Availability Section?	Yes	Data Availability: page 24
Were **human clinical and genomic datasets** deposited in a public access-controlled repository in accordance to ethical obligations to the patients and to the applicable consent agreement?	Not Applicable	
Are **computational models** that are central and integral to a study available without restrictions in a machine-readable form? Were the relevant accession numbers or links provided?	Not Applicable	
If publicly available data were reused, provide the respective **data citations in the reference list**.	Not Applicable	
