# Supplementary material for: The onset of PI3K‐related vascular malformations occurs during angiogenesis and is prevented by the AKT inhibitor miransertib
Source: EMBO Mol Med. 2022 Jun 13;14(7):e15619. doi: 10.15252/emmm.202115619 (PMC9260211; doi:10.15252/emmm.202115619)
Supplement: Supplementary file 3 — Source Data for Expanded View and Appendix [file EMMM-14-e15619-s003.zip › Fig_S3.zip!/Fig S3/WB Fig S3D.pdf]

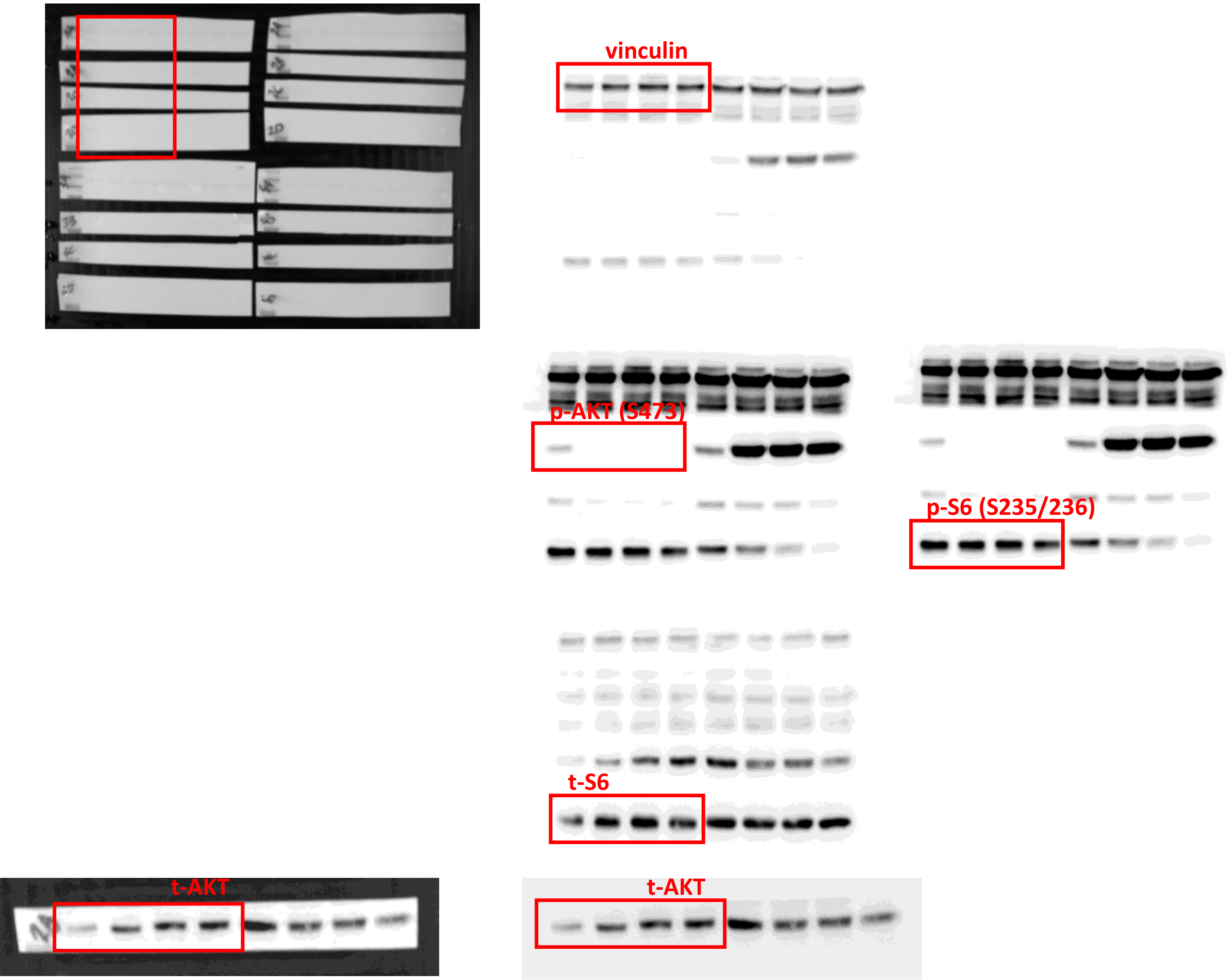

vinculin
p-AKT (S473)
p-S6 (S235/236)
t-S6
t-AKT
t-AKT
