# Supplementary material for: The onset of PI3K‐related vascular malformations occurs during angiogenesis and is prevented by the AKT inhibitor miransertib
Source: EMBO Mol Med. 2022 Jun 13;14(7):e15619. doi: 10.15252/emmm.202115619 (PMC9260211; doi:10.15252/emmm.202115619)
Supplement: Supplementary file 3 — Source Data for Expanded View and Appendix [file EMMM-14-e15619-s003.zip › Fig_S2.zip!/Fig S2/WB Fig S2A.pdf]

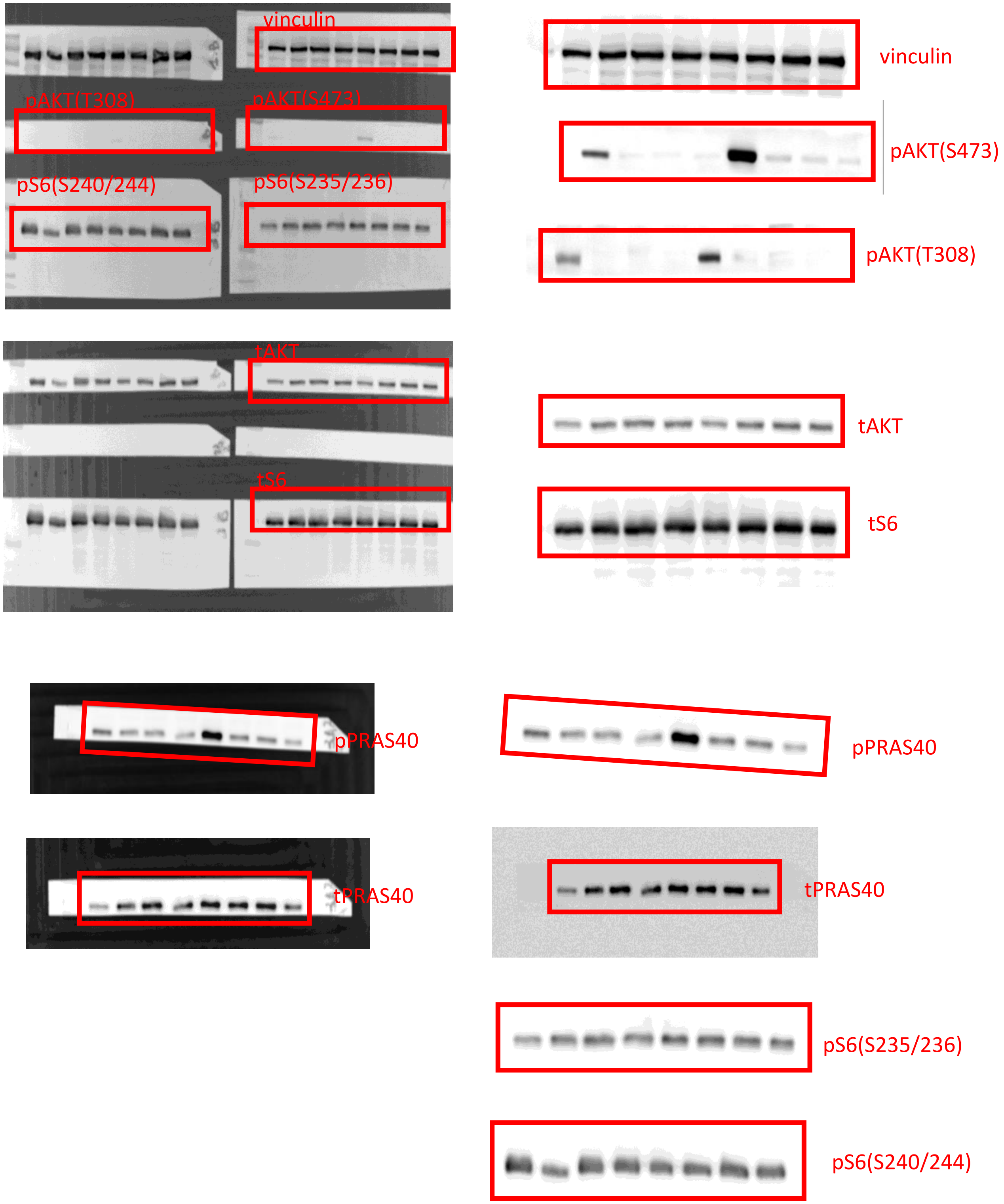
vinculin
pAKT(T308)
pAKT(S473)
pS6(S240/244)
pS6(S235/236)
tAKT
tS6
pPRAS40
tPRAS40
vinculin
pAKT(S473)
pAKT(T308)
tAKT
tS6
pPRAS40
tPRAS40
pS6(S235/236)
pS6(S240/244)
